# A general approach to stereospecific Pd-catalyzed cross-coupling reactions of benzylic stereocenters†

**DOI:** 10.1039/d3sc04519f

**Published:** 2023-11-24

**Authors:** Meruyert Binayeva, Xinghua Ma, Pejman Ghaemimohammadi, Mark R. Biscoe

**Affiliations:** a Department of Chemistry and Biochemistry, The City College of New York (CCNY) New York NY 10031 USA mbiscoe@ccny.cuny.edu; b The Graduate Center of the City University of New York (CUNY) 365 Fifth Avenue New York NY 10016 USA

## Abstract

We have developed a general process for the formation of enantioenriched benzylic stereocenters *via* stereospecific Pd-catalyzed cross-coupling reactions of enantioenriched benzylic tricyclohexyltin nucleophiles. This process proceeds with excellent stereospecificity for a remarkably broad scope of electrophilic coupling partners including aryl and heteroaryl halides and triflates, acid chlorides, thioesters, chloroformates, and carbamoyl chlorides. Thus, enantioenriched 1,1-diarylalkanes as well as formal products of asymmetric enolate arylation are readily accessed using this approach. We additionally provide the first demonstration of a Sn-selective cross-coupling reaction using a vicinal alkylborylstannane nucleophile. In these reactions, the presence of cyclohexyl spectator ligands on tin is essential to ensure selective transfer of the secondary benzylic unit from tin to palladium.

## Abstract 2

Stereospecific Pd-catalyzed cross-coupling reactions using enantioenriched benzylic tricyclohexyltin nucleophiles enable broad access to enantioenriched benzylic stereocenters.

## Graphical abstract

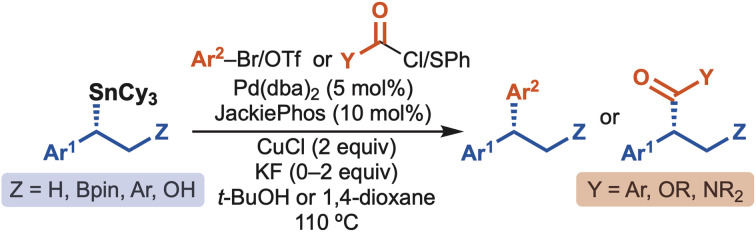

Aryl-substituted stereocenters are ubiquitous structural components of biologically active molecules.^1^ Of potential aryl-containing stereocenters, 1,1-diaryl stereocenters and stereocenters formed from α-arylated carbonyl groups are particularly common structural motifs found in compounds emerging from the drug-discovery process (Scheme 1).^1–3^ In most instances, a single enantiomer of a racemic mixture constitutes the therapeutically active species, while the opposite stereoisomer may be inactive, reduce the efficacy of the active species, or result in deleterious side effects.^4^ Therefore, synthetic strategies to rapidly and reliably introduce or manipulate aryl-substituted stereocenters with precise control of absolute stereochemistry are of significant medicinal importance.

**Scheme 1:**
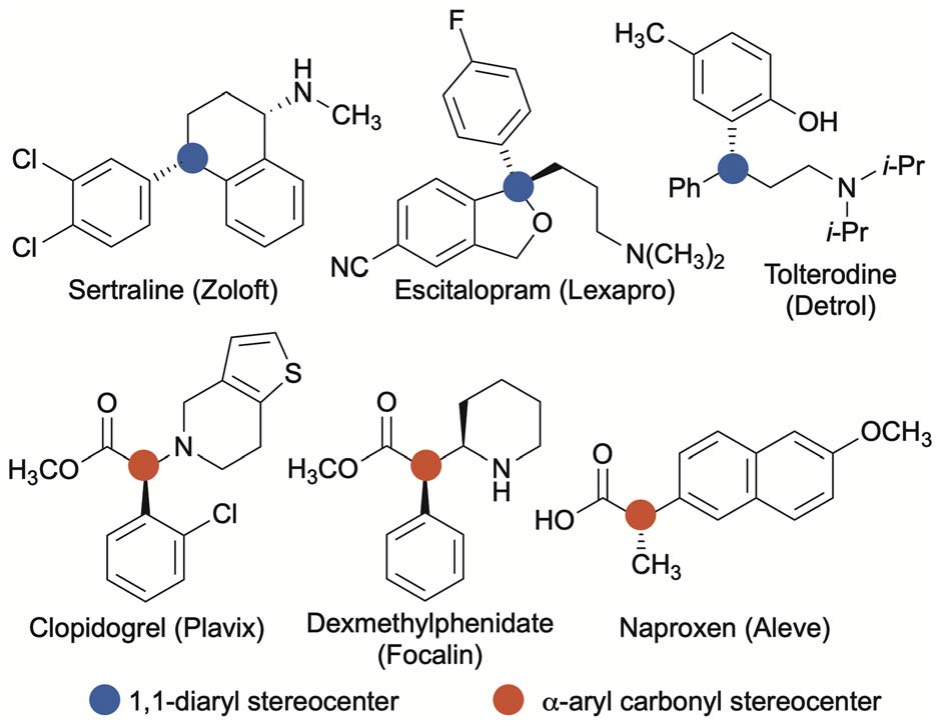
Examples of benzylic stereocenters in small-molecule drugs.

Stereospecific transition metal-catalyzed cross-coupling reactions employing configurationally stable enantioenriched nucleophiles (1) have emerged as a powerful alternative approach to asymmetric synthesis (Scheme 2).^5,6^ Though this approach requires the preparation of an enantioenriched main group organometallic nucleophile, stereochemical information can often be reliably transferred *via* a stereoretentive or stereoinvertive transmetallation event in ensuing cross-coupling processes.^5*e*^ Previous studies of Pd-catalyzed stereospecific Stille reactions suggest that the stereoretentive course of transmetallation is strongly preferred for enantioenriched alkyltin nucleophiles when Cu(i) is employed as a co-transmetallating agent, and is often independent of the electronic and steric properties of the nucleophilic and electrophilic coupling partners.^7^ Thus, stereospecific Stille cross-coupling reactions have tremendous potential as a general synthetic strategy to predictably modify three-dimensional molecular structure.

**Scheme 2:**
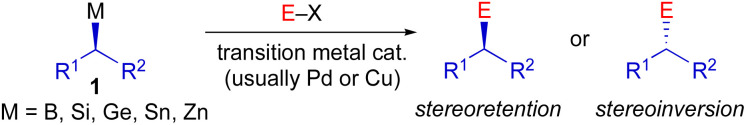
Stereospecific transition metal-catalyzed cross-coupling reactions using enantioenriched main group organometallic nucleophiles.

Because organostannanes used in cross-coupling reactions possess four organic units, each of which is theoretically capable of undergoing transmetallation to Pd, it is essential that selective transfer of a single unit be achieved during the cross-coupling process. The selective transfer of a C(sp) or C(sp^2^) group from an organostannane is commonly achieved by incorporation of three alkyl units (*e.g.*, Me or *n*-Bu) onto tin, which serve as spectator ligands due to the slow rate of alkyl transfer from tin (Scheme 3).^8^ Slow alkyl transfer from tin therefore complicates efforts to effect the selective transfer of a single alkyl unit from a tetraalkylstannane. We have demonstrated that incorporation of a carbastannatrane backbone^9^ into the alkylstannane enables the selective transfer of an otherwise unactivated secondary alkyl group in Pd-catalyzed cross-coupling reactions (Scheme 4a) when CuCl is employed as a co-transmetallating agent and JackiePhos^10^ (2) as the supporting phosphine ligand.^7*d*,*g*,*h*^ However, in the absence of a carbastannatrane activating group, significant electronic differentiation of single alkyl unit of the tetraalkylstannane has been necessary to effect selective alkyl transfer. Hoppe^11^ and Falck^7*a*^ have separately demonstrated the ability of highly activated secondary alkyl groups to undergo selective and stereospecific transfer to Pd in the presence of *n*-butyl ligands. In these instances, electronic activation (*i.e.*, inclusion of an α-heteroatom or α-C(sp^2^) substituent) in combination with a remote coordinating group was required to promote selective alkyl transfer (Scheme 4b and c). More recently, to effect selective transmetallation of less activated secondary alkyl units, we replaced the *n*-butyl spectator ligands of the enantioenriched tetraalkylstannane with cyclohexyl spectator ligands.^7*i*,12^ This modification enabled the selective and stereospecific transfer of nitrogen-containing stereocenters (Scheme 4d) to Pd, and was thereafter extended to use with racemic α-oxygenated secondary alkylstannanes not bearing a remote directing/activating oxygen-containing group.^13^ These results suggested that selective and stereospecific transfer of marginally activated secondary alkyl groups might be broadly achievable for other enantioenriched alkyltricyclohexylstannane nucleophiles. Herein, we describe the use of enantioenriched benzylic tricyclohexylstannanes in stereospecific Pd-catalyzed reactions with aryl and acyl electrophiles (Scheme 4e). In this system, incorporation of cyclohexyl ligands into the organostannane nucleophile is essential to achieve selective transfer of the benzylic unit to Pd. In line with previous stereospecific Stille couplings, this process proceeds with high stereospecificity while tolerating an extraordinarily broad scope of electrophilic coupling partners, including aryl and heteroaryl halides and triflates, acid chlorides, thioesters, chloroformates, and carbamoyl chlorides. Thus, in addition to products from stereospecific arylation reactions (*i.e.*, 1,1-diaryl stereocenters),^14^ formal products of asymmetric enolate arylation reactions can also be readily obtained using this approach.^15–19^ This process will therefore serve as a general synthetic strategy for the stereocontrolled formation of new benzylic stereocenters.

**Scheme 3:**
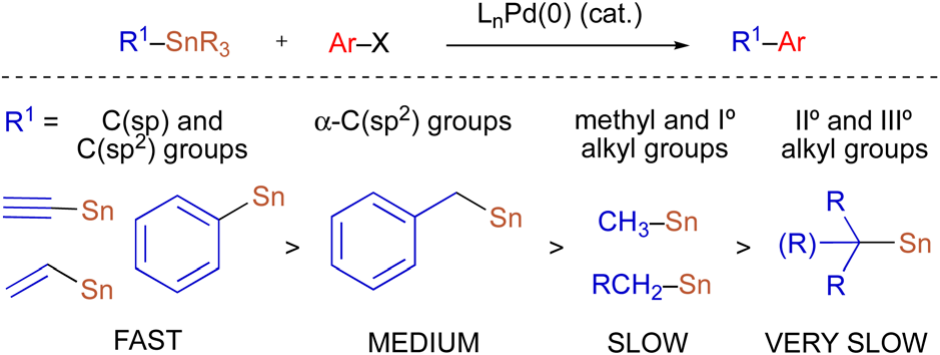
Relative rates of group transfer in Pd-catalyzed cross-coupling reactions of tetraorganostannanes.

**Scheme 4:**
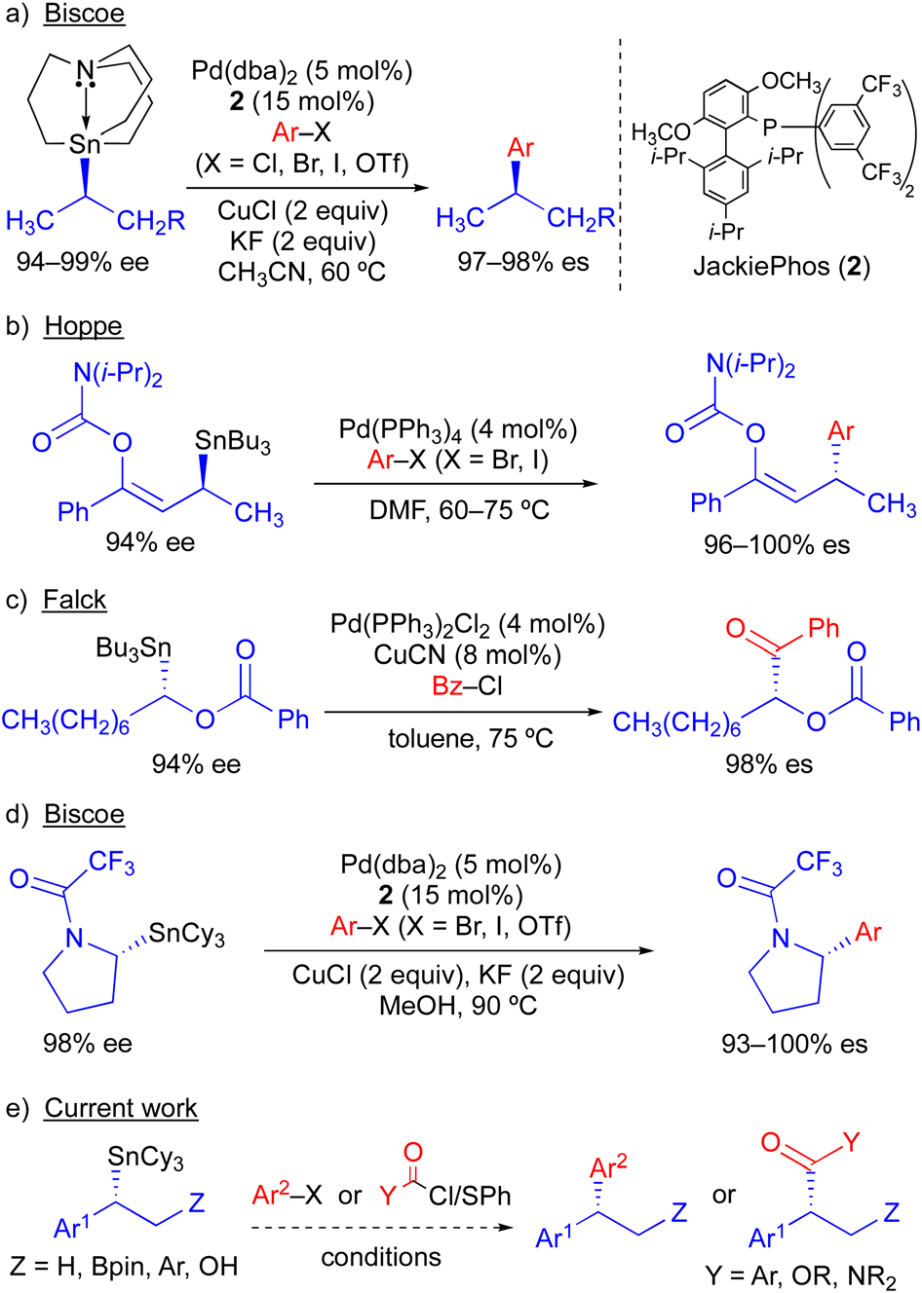
Stereospecific Pd-catalyzed Stille cross-coupling reactions using enantioenriched secondary alkylstannanes.

We have previously found that the use of JackiePhos (2) as a supporting phosphine ligand facilitates transmetallation of alkyl groups from organostannanes in Pd-catalyzed Stille cross-coupling reactions when CuCl is included as a co-transmetallating agent (see Scheme 4a and d).^7*d*,*g*–*i*^ Thus, 2 was a logical choice of supporting phosphine ligand for use in exploratory Pd-catalyzed cross-coupling studies using enantioenriched benzylic organostannanes. We initiated our investigations using secondary benzylic tributylstannane 3a in cross-coupling reactions with 4-bromoacetophenone (Table 1). Use of 3a as the nucleophilic coupling partner resulted in low yields of coupling product 4a and/or significant *n*-butyl transfer (5a) for all solvents examined. In contrast, when tricyclohexylstannane analogue 3b was employed, significantly improved yields of 4a were obtained alongside only nominal evidence (less than 2% by gas chromatography) of competitive cyclohexyl coupling (5b). We found that reactions conducted at 110 °C provided optimal reaction conversion across the range of conditions examined. These results indicate that although a secondary benzylic stereocenter is insufficiently activated to outcompete transfer of an unactivated primary alkyl group from tin, selective transfer can be achieved in the presence of an unactivated secondary alkyl unit. To investigate potential stereochemical transfer in these reactions, enantioenriched 3b was prepared using a stereoselective sulfoxide-directed metalation strategy previously reported by Ruano and Padwa.^20^ When enantioenriched 3b was employed under the optimized cross-coupling reaction conditions, exceptional stereofidelity was observed (Table 1, entries 9 and 10). Thus, a selective and enantiospecific Pd-catalyzed coupling was achieved using an enantioenriched secondary benzylic tricyclohexylstannane nucleophile.

**Table 1:** Effect of tin spectator ligand and solvent on the selectivity of Stille cross-coupling reactions using 2

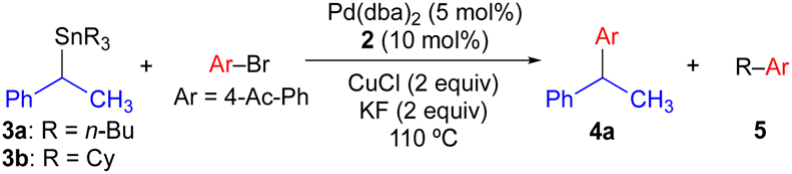				
Entry	R	Solvent	4a yield^a^ (%)	5 yield^a^ (%)
1	*n*-Bu (3a)	CH_3_CN	29	45 (5a)
2	*n*-Bu	Toluene	11	8
3	*n*-Bu	CH_3_OH	18	14
4	*n*-Bu	1,4-Dioxane	20	9
5	*n*-Bu	*t*-BuOH	26	19
6	Cy (3b)	CH_3_CN	69	<2 (5b)
7	Cy	Toluene	57	<2
8	Cy	CH_3_OH	32	<2
9	Cy	1,4-Dioxane	72 (99% es)^b^	<2
10	Cy	*t*-BuOH	82 (97% es)^b^	<2

a Calibrated GC or ^1^H NMR yields.

b Using (*S*)-3b (96% ee); % es = [% ee of product]/[% ee of starting material].

Using the optimized conditions of Table 1, we investigated the scope of electrophilic coupling partners tolerated in stereospecific cross-coupling reaction with enantioenriched organostannane 3b (Table 2). Electron-rich, electron-neutral, and electron-deficient aryl electrophiles all undergo efficient Pd-catalyzed cross-coupling reactions, generating 1,1-diaryl stereocenters (4) with high enantiospecificity. The use of heteroaryl electrophiles is also acceptable, as is the use of an aryl electrophile bearing an *ortho* substituent. Thus, enantiospecificity is largely independent of the steric and electronic properties of the electrophilic aryl coupling partner, which is consistent with our past observations for stereospecific Pd-catalyzed Stille reactions. Additionally, acyl electrophiles can be successfully employed using the same reaction conditions (6a–6e), generating formal products of asymmetric enolate arylation reactions. Asymmetric mono-arylation reactions that form tertiary α-keto stereocenters constitute a major synthetic challenge due to the potential for diarylation as well as the propensity of the arylated α-keto stereocenter to racemize under basic conditions.^16^ Our ability to access products 6a–6e indicates that formal products of the asymmetric enolate arylation of ketones, esters, and amides can be broadly achieved using a stereospecific benzylic coupling strategy, thus circumventing the problems commonly associated with asymmetric enolate arylation. In cases where an enantioenriched acyl electrophile is employed, high reagent-controlled diastereoselectivity is achieved (6d, 6e). For all cross-coupling reactions in Table 2, no evidence of cyclohexyl transfer was observed.

**Table 2:** Stereospecific Pd-catalyzed couplings of enantioenriched benzylic tricyclohexylstannane 3b^a^

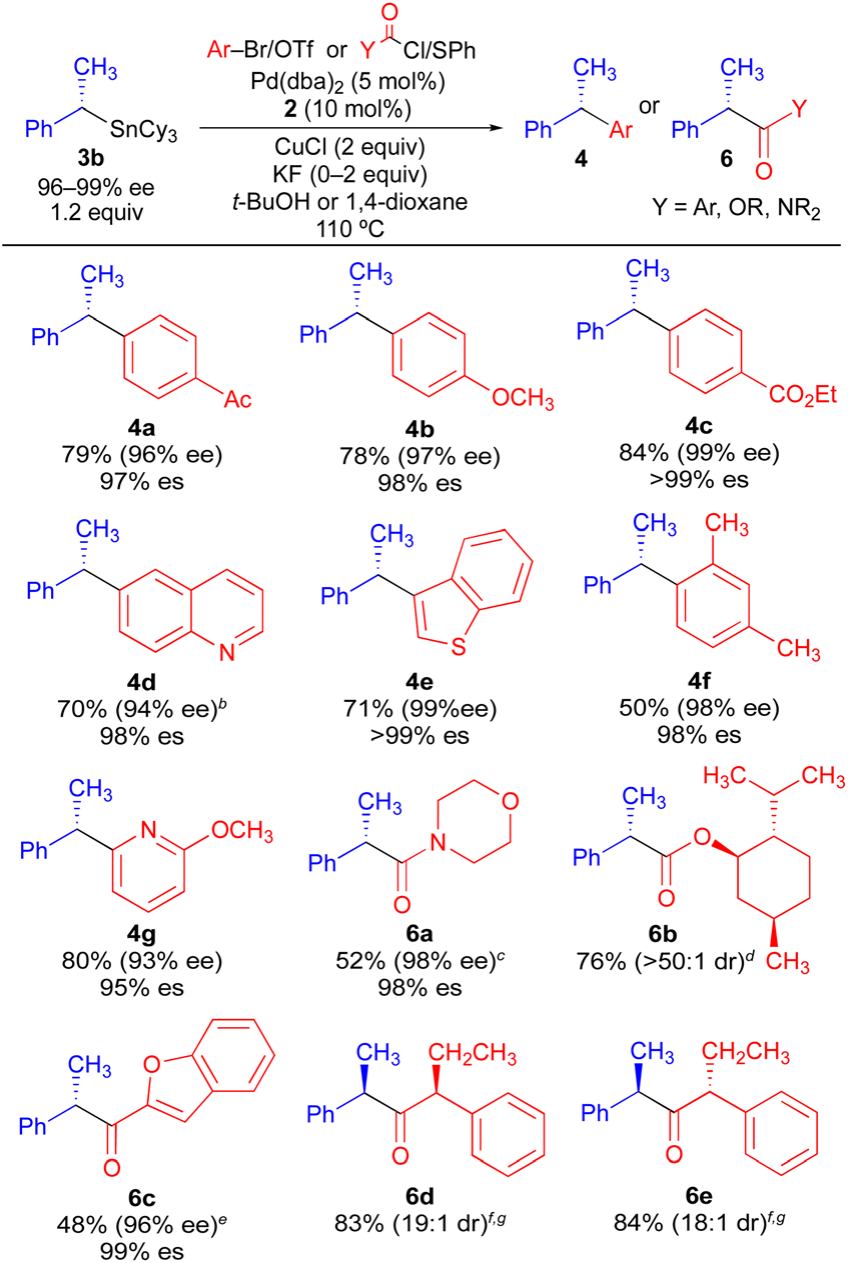

a Average isolated yields of duplicate runs; % es = [% ee of product]/[% ee of starting material].

b Using aryl triflate.

c Using carbamoyl chloride.

d Using chloroformate.

e Using acid chloride.

f Using thioester.

g Using (*R*)-3b.

As a more general approach to the preparation of enantioenriched benzylic stannanes, we adapted the asymmetric copper-catalyzed borylstannation of styrene developed by Liao using chiral sulfinylphosphine ligand 7.^21^ Using *t*-amyl alcohol as solvent and increasing the CuCl loading, we found that tricyclohexyltin could be incorporated into this process affording α-aryl-β-borylstannane 8 in high enantiomeric excess (Scheme 5). Styrene derivatives with aryl groups bearing an electron-donating group, electron-withdrawing group, and *ortho*-substituent could each be employed in this process. However, heteroaryl styrene derivatives appear to be incompatible with this transformation. Conceptually, enantioenriched 8 constitutes a powerful building block for use in stereospecific cross-coupling reactions, which could be employed in tin-selective or boron-selective cross-coupling processes. Using a known process for Pd-catalyzed Suzuki reactions of primary alkylboronate esters with 8a,^21^ we achieved a boron-selective cross-coupling reaction with no measurable erosion of enantioenrichment. Alternatively, when we employed our standard conditions from Table 2 with 8a, we successfully achieved a tin-selective cross-coupling reaction with efficient transfer of stereochemistry. Though Sn-selective cross-coupling reactions of vicinal borylstannyl alkenes have been demonstrated,^22^ to the best of our knowledge, there is no prior report of a Sn-selective cross-coupling reaction of a C(sp^3^)–Sn bond in the presence of a C(sp^3^)–B bond.^23^

**Scheme 5:**
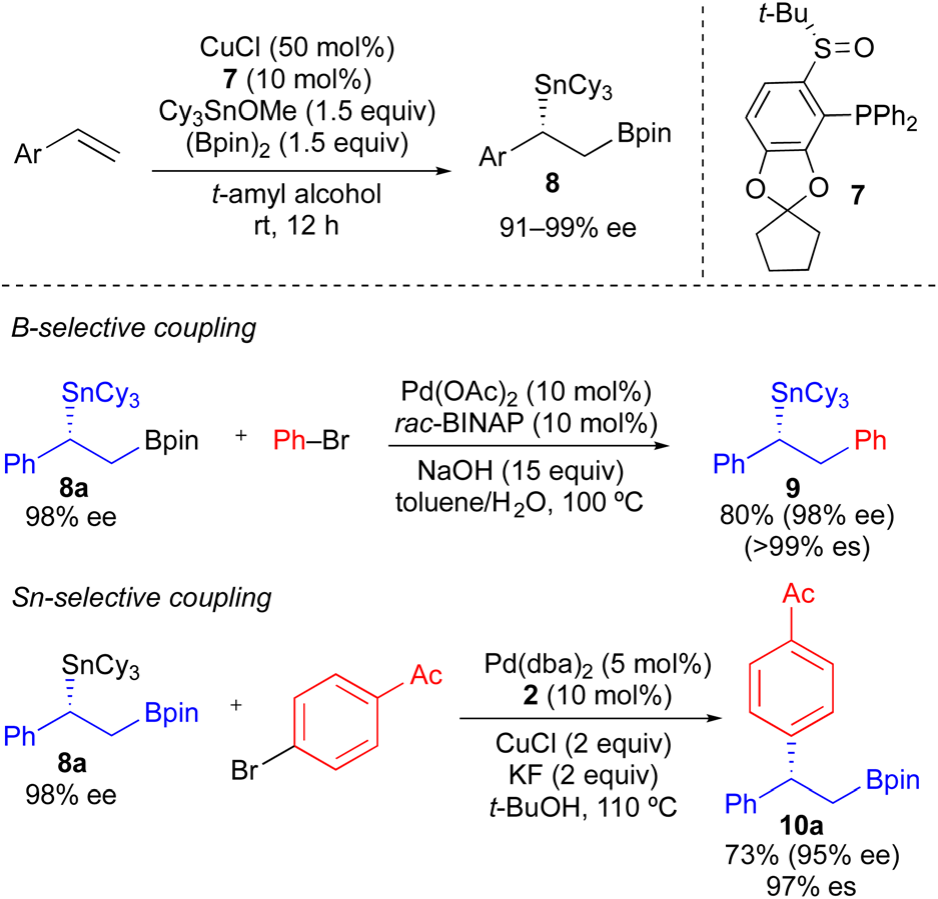
Formation of enantioenriched α-aryl-β-borylstannanes *via* asymmetric Cu-catalyzed borylstannation of styrenes, and their use in B-selective and Sn-selective cross-coupling reactions.

Our initial studies using borylstannane 8a (and aryl analogues thereof) focused on stereospecific Sn-selective couplings. Such a process leaves the primary Bpin group on the cross-coupling product, which could serve as a versatile functional handle for further structural modification.^24^ Using the Sn-selective stereospecific cross-coupling conditions, highly enantioenriched alkylboron products containing a 1,1-diaryl stereocenter could be readily prepared and isolated (Table 3, 10a, 10e). As we found that oxidation of the primary Bpin group facilitated chiral-phase HPLC analysis, we incorporated an optional oxidative workup into our general cross-coupling procedure and isolated the hydroxyl derivatives in the majority of our examples. Again, high enantiospecificities could be achieved in cross-coupling reactions using α-aryl-β-borylstannane nucleophiles regardless of the electronic or steric properties (electron-rich, electron-deficient, *ortho*-substituted) of the electrophilic coupling partner. Additionally, we found that α-aryl-β-borylstannane nucleophiles (8) derived from styrene precursors with sterically and electronically differentiated aryl rings could be efficiently employed in these stereospecific benzylation reactions. Identical reaction conditions to those of Table 2 were employed for cross-coupling reaction of 8, with no reoptimization of individual reactions. Thus, we have established a single set of reaction conditions that can be universally employed in these stereospecific benzylation reactions. Though these standard conditions do result in slightly diminished yields for products of Table 3 when compared to products of Table 2, we note that yields can be improved *ca.* 10–30% by increasing the loading of organostannane from 1.2 equiv. to 2.0 equiv. Finally, enantioenriched benzylic stannanes (11) prepared *via* boron-selective cross-coupling or oxidation reactions were employed in stereospecific Pd-catalyzed arylation and acylation reactions (Table 4). Again, high stereofidelity was observed in all of these reactions. It is particularly noteworthy that this cross-coupling reaction cleanly tolerates the presence of a free β-hydroxyl group (12b) on the enantioenriched organostannane.

**Table 3:** Stereospecific Pd-catalyzed Stille couplings of enantioenriched benzylic 1,2-borylstannanes (8)^a^

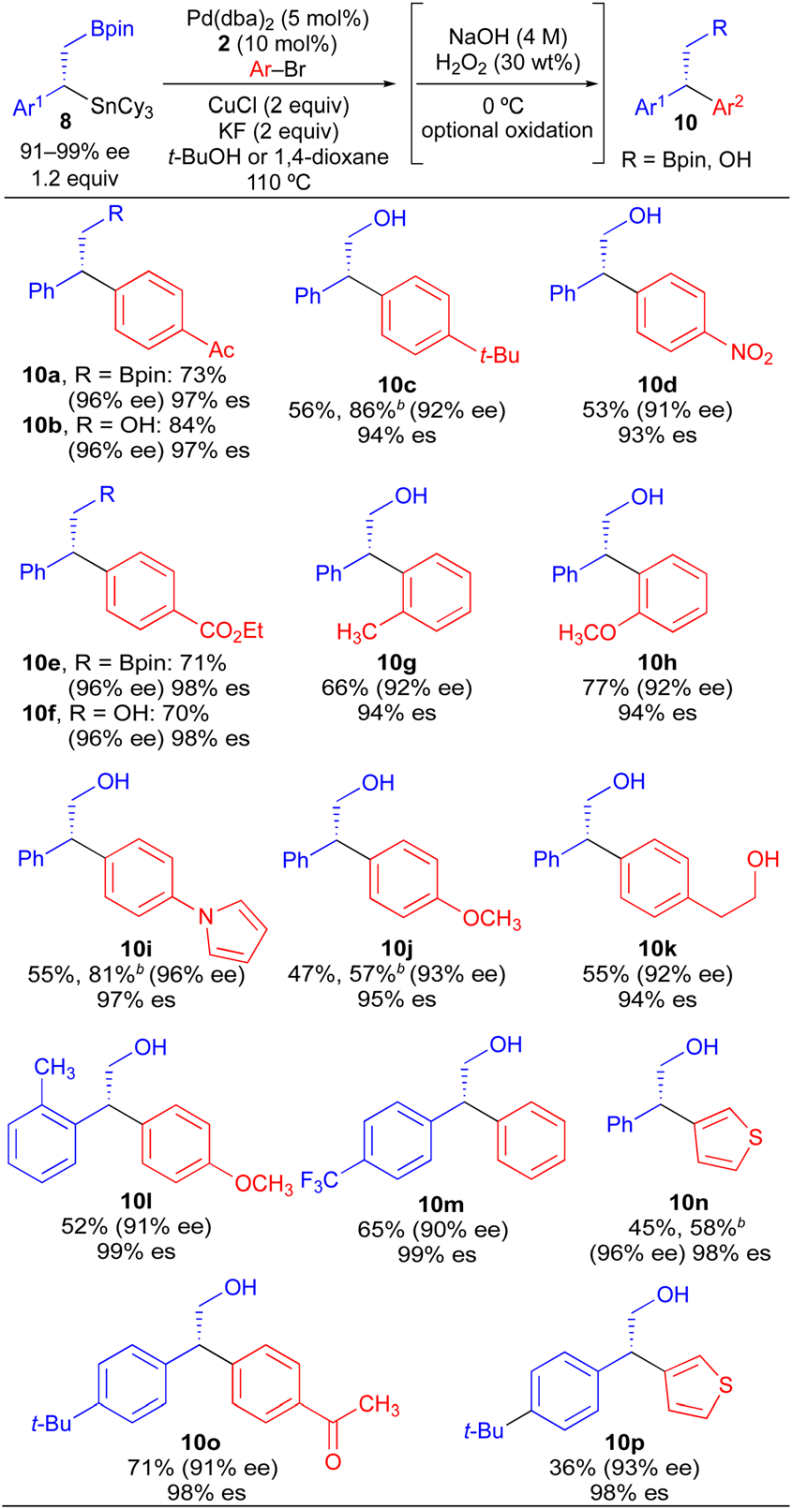

a Average isolated yields of duplicate runs; % es = [% ee of product]/[% ee of starting material].

b Using 2.0 equiv. of 8.

**Table 4:** Stereospecific Pd-catalyzed Stille couplings of enantioenriched benzylic tricyclohexylstannanes (11) prepared *via* B-selective functionalization of enantioenriched benzylic 1,2-borylstannanes^a^

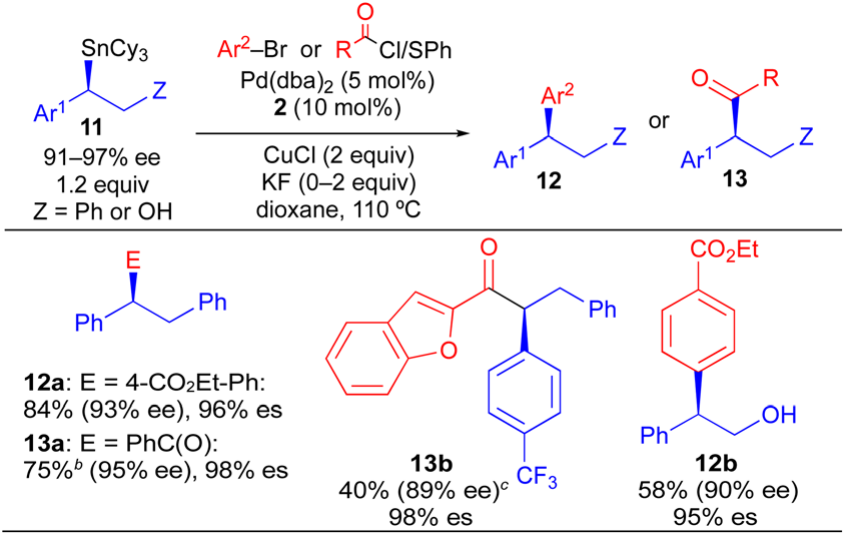

a Average isolated yields of duplicate runs; % es = [% ee of product]/[% ee of starting material].

b Using acid chloride.

c Using thioester.

This stereospecific benzylation process, in combination with our previous studies using enantioenriched secondary alkylstannane nucleophiles, suggests that the stereochemical course of alkyltin transmetallation is largely independent of the electronic and steric properties of the nucleophilic and electrophilic coupling partners when Pd/Cu are employed. The uniformity of reaction conditions (*i.e.*, Pd/Cu/JackiePhos) across diverse classes of alkylstannane nucleophiles (benzylic, nitrogen-containing,^7*i*^ oxygen-containing,^13^ and unactivated stereocenters^7*d*^) is a particularly significant feature of this chemistry, which will facilitate its future application to diversity-oriented synthesis. In contrast, the dominant stereochemical course of alkylboron transmetallation in analogous stereospecific Suzuki cross-coupling processes is dictated by subtle electronic and steric perturbations of the coupling partners.^5*e*^ Thus, it is uncommon for the reaction conditions for a stereospecific Suzuki transformation using a specific class of alkylboron nucleophile to be successfully extended to couplings that employ alternative, electronically differentiated alkylboron nucleophiles. In addition to facilitating selective alkyl transfer to copper and palladium, use of RSnCy_3_ compounds in place of RSnBu_3_ compounds offers important practical benefits. ClSnCy_3_ is crystalline and odorless, and exhibits significantly lower toxicity than commonly used ClSnBu_3_.^25^ RSnCy_3_ derivatives also tend to be highly crystalline, in contrast to RSnBu_3_ derivatives which tend to be oils. Thus, the crystallinity and solubility profile of tricyclohexyltin byproducts should enable tin to be readily purged from waste streams *via* solvent swaps and crystallization. As concerns regarding organotin toxicity and the removal of tin byproducts can heavily influence the decision to incorporate the Stille reaction into a synthetic sequence when SnBu_3_ (or SnMe_3_) nucleophiles are required, we expect that the use of SnCy_3_-based nucleophiles will extend beyond cross-coupling reactions of activated secondary alkyl groups and may find application in traditional C(sp^2^)–C(sp^2^) Stille cross-coupling reactions where aryl and vinyl SnBu_3_ derivatives are commonly employed.

## Conclusions

In summary, we have developed a general strategy for the formation of enantioenriched benzylic stereocenters *via* stereospecific Pd-catalyzed cross-coupling reactions of enantioenriched benzylic tricyclohexyltin nucleophiles. The use of cyclohexyl spectator ligands on the benzylic stannanes is vital to ensure selective stereospecific transfer of the secondary benzylic unit. In addition to aryl bromide and triflate electrophiles, chloroformates, carbamoyl chlorides, acid chlorides, and thioesters can be efficiently employed in these stereospecific cross-coupling reactions. Thus, enantioenriched 1,1-diarylalkanes as well as formal products of asymmetric enolate arylation are readily accessed using this approach. For all electrophiles and nucleophiles employed, excellent enantiospecificity or diasterospecificity was observed, indicating that the stereochemical transfer is widely independent of the electronic and steric properties of both the electrophilic and nucleophilic coupling partners employed. This work further illustrates the broad synthetic advantages achievable by incorporating cyclohexyl spectator ligands into organostannanes used in Stille cross-coupling reactions. Based on these advantages, we feel that applications of organotricyclohexylstannanes (RSnCy_3_) may transcend C(sp^2^)–C(sp^3^) cross-coupling reactions and find utility in C(sp^2^)–C(sp^2^) cross-coupling reactions where RSnBu_3_ or RSnMe_3_ nucleophiles are more commonly employed but less desirable.

## Data availability

All data have been included in the ESI.†

## Author contributions

Xinghua Ma initiated this project and developed the standard reaction conditions. Xinghua Ma, Meruyert Binayeva, and Pejman Ghaemimohammadi all contributed to cross-coupling reactions of Table 2. Meruyert Binayeva conducted all experiments shown in other schemes and tables.

## Conflicts of interest

There are no conflicts to declare.

## Supplementary Material

SC-014-D3SC04519F-s001

SC-014-D3SC04519F-s002
