# Supplementary material for: A general approach to stereospecific Pd-catalyzed cross-coupling reactions of benzylic stereocenters
Source: Chem Sci. 2023 Nov 24;14(48):14124–30. doi: 10.1039/d3sc04519f (PMC10717501; doi:10.1039/d3sc04519f)
Supplement: SC-014-D3SC04519F-s001 [file SC-014-D3SC04519F-s001.pdf]

Electronic Supplementary Material (ESI) for Chemical Science.
This journal is © The Royal Society of Chemistry 2023

A General Approach to Stereospecific Pd-Catalyzed Cross-Coupling Reactions of Benzylic Stereocenters

Meruyert Binayeva, Xinghua Ma, Pejman Ghaemimohammadi, Mark R. Biscoe*

Supporting Information

Table of Contents

1. General Reagent and Analytical Information……S2
2. Optimization of Asymmetric Borylstannylation……S3
3. Procedural Information……S4
   - *Procedure for preparation of sulfinylphosphine ligand 7*……S4
   - *Procedure for preparation of enantioenriched **3b***……S6
   - *General procedure for preparation of enantioenriched 1,2-borylstannanes **8***……S8
   - *General procedure for oxidation of C–B bond*……S11
   - *General procedure for Suzuki cross-couplings of 1,2-borylstannanes*……S14
   - *General procedures for Stille cross-couplings of alkylstannanes*……S16
4. Cross-Coupling Product Characterization Data……S18
5. X-ray Crystal Structure of **8a**……S33
6. References……S33
7. Chiral-Phase HPLC Data……S35
8. ^{1}H, ^{13}C, ^{119}Sn NMR Spectra……S69

1. General Reagent and Analytical Information

Commercially available reagents were purchased from Sigma-Aldrich, Alfa-Aesar, or Acros, and used as received unless otherwise noted. BDH brand ethyl ether was purchased from VWR. EMD brand Omnisolv THF (unstabilized) was purchased from Fisher. These solvents were transferred to separate 20 L solvent-delivery kegs and vigorously purged with argon for 2 h. The solvents were further purified by passing them under argon pressure through two packed columns of neutral alumina. Other anhydrous solvents (Sigma-Aldrich, SureSeal) were purged with argon prior to use. Water used for Suzuki reactions was degassed prior to use. Tricyclohexyltin chloride was purchased from Gelest Inc., or prepared from tricyclohexyltin hydroxide (SageChem) according to reference.[1] Raney Ni (W.R. Grace and Co. Raney® 2400) was purchased from Sigma-Aldrich. Flash chromatography was performed using Silicycle silica gel (ultra-pure grade). Solvents used for flash chromatography (ACS grade) were purchased from Fisher Scientific, and used as received. All reactions were performed in oven-dried glassware under Ar atmosphere, unless otherwise noted. Reaction mixtures were monitored by thin layer chromatography (TLC) with visualization by fluorescence quenching at 254 nm, or by gas chromatography (GC). TLC plates were stained using potassium permanganate solution or I_2.

Compounds are characterized by ^{1}H NMR, ^{13}C NMR, and ^{119}Sn NMR. All NMR spectra were obtained on a Bruker 300 (300 MHz for ^{1}H, 75 MHz for ^{13}C, and 112 MHz for ^{119}Sn). Calibrated ^{1}H NMR yields were obtained using mesitylene as an internal standard. All ^{1}H NMR experiments are reported in δ units, parts per million (ppm), and are measured relative to the signals for residual chloroform (7.26 ppm), unless otherwise noted. The following abbreviations are used to express the multiplicities: s = singlet; d = doublet; t = triplet; m = multiplet; br = broad, app = apparent. All ^{13}C NMR spectra are reported in ppm relative to deuterochloroform (77.23 ppm), and were obtained with ^{1}H decoupling. All previously unreported compounds are additionally characterized by high resolution MS. High resolution MS analyses were performed on a Bruker Maxis-II ETD ESI-QTOF instrument. All GC analyses were performed on a Shimadzu GC-2030 gas chromatograph. GC yields were obtained using dodecane as an internal standard. Chiral-phase HPLC analyses were performed using a Shimadzu Prominence HPLC system with binary mobile phase pumps and UV-vis detector (LC-20AB, SPD-20A) using an OD-RH (dimensions: 4.6 mm

x 150 mm; particle size: 5 μm) chiral-phase column (Daicel), an IA (dimensions: 4.6 mm x 150 mm; particle size: 5 μm) chiral-phase column (Daicel), IC3 (dimensions: 4.6 mm x 250 mm; particle size: 3 μm) chiral-phase column (Daicel), or OJ-RH (dimensions: 4.6 mm x 150 mm; particle size: 5 μm) chiral-phase column (Daicel).

2. Optimization of Asymmetric Borylstannylation

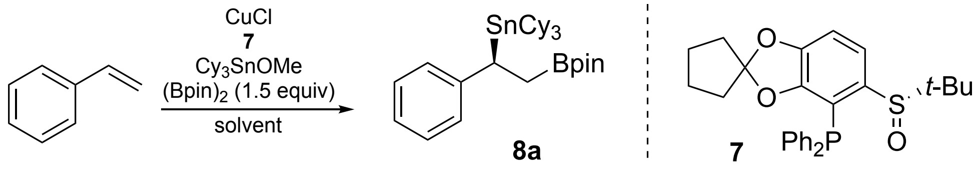

Entry	Solvent	Temp. (ºC)	CuCl (equiv)	**7** (equiv)	Cy_3SnOMe (equiv)	Yield (%)	% ee
1	*t*-BuOH	25	0.5	0.1	1.5	51%	93
2	dibutyl ether	25	0.5	0.1	1.5	61%	74
3	*N,N*-dimethyl acetamide	25	0.5	0.1	1.5	4%	n/a
4	*t*-BuOH	25	0.7	0.1	1.5	19%	88.3
5	*t*-BuOH	25	0.2	0.1	1.5	48%	91.7
6	*t*-BuOH	25	0.1	0.1	1.5	30%	87.4
7	*t*-BuOH	25	0.1	0.05	1.5	54%	88.5
8	MeCN	25	0.5	0.1	1.5	15%	78
9	*sec*-BuOH	25	0.5	0.1	1.5	39%	91.5
10	2-propanol	25	0.5	0.1	1.5	49%	89.8
11	EtOH	25	0.5	0.1	1.5	25%	89.5
12	*sec*-BuOH	4	0.5	0.1	1.5	43%	93.7
13	*sec*-BuOH	4	0.5	0.1	3	56%	93.2
14	*sec*-BuOH	–78	0.5	0.1	1.5	35%	91.2
15	toluene	25	0.5	0.1	1.5	37%	90.1
16	MTBE	25	0.5	0.1	1.5	35%	88.2
17	1-propanol	25	0.5	0.1	1.5	>5	n/a
18	DMSO	25	0.5	0.1	1.5	13%	14.4
19	*t*-amyl alcohol	25	0.5	0.1	1.5	56%	98.5
20	THF	25	0.1	0.1	1.5	70%	91

Figure S1. Effect of solvent, temperature and CuCl equivalents on 1,2-borylstannane synthesis. Enantiomeric excess was determined using product of B-selective Suzuki reaction (see page S16).

3. Procedural Information

Procedure for preparation of sulfinylphosphine ligand 7.[2]

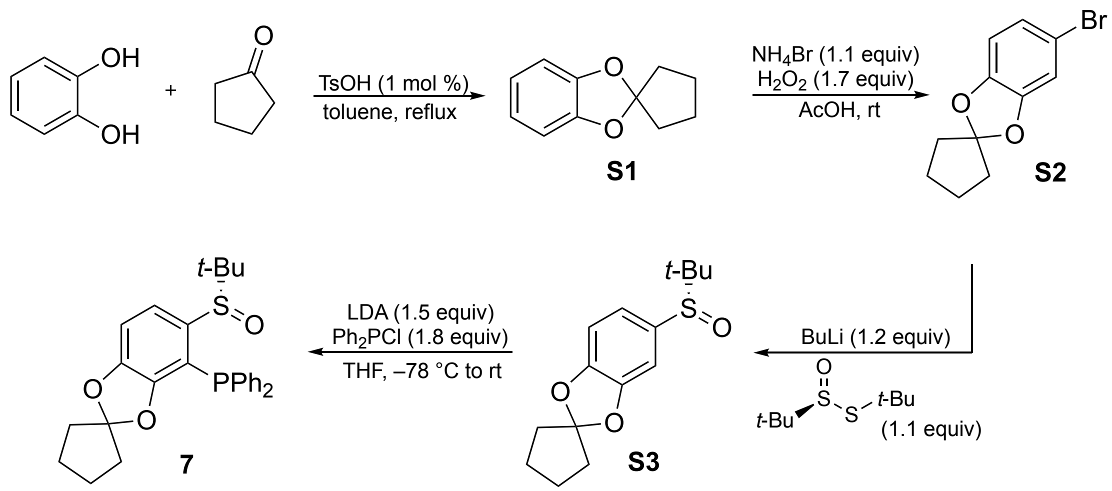

Figure S2. Reaction scheme for preparation of **7**.

To an oven-dried round bottom flask equipped with a stirbar under atmosphere of Argon, catechol (100 mmol), cyclopentanone (100 mmol) and TsOH (0.1 mmol) werer added. Toluene (150 mL) was then added and the resulting mixture was refluxed using a Dean-Stark trap at 130 ºC for 24 h. The reaction mixture was poured into a separatory funnel and washed with aqueous NaOH (2 x 50 mL, 4 M). The organic layer was separated, washed with water (50 mL) and brine (50 mL), dried over Na_2SO_4, filtered. Solvent was removed under reduced pressure, **S1** (6.6 g, 38% yield) was isolated by flash column chromatography (10:90 ethyl acetate:hexanes). On the bench top, **S1** (30.8 mmol), NH_4Br (33.8 mmol) and AcOH (49 mL) were added to an oven-dried round bottom flask equipped with a stirbar. After stirring for 10 min at rt, H_2O_2 (5.5 mL, 30 wt%) was added dropwise at 0 ºC to the reaction vessel. The reaction mixture was allowed to stir for 12 h. The resulting solution was poured into separatory funnel containing saturated aqueous $NaHCO_3$ (100 mL). The organic layer was extracted with DCM, washed sequentially with water and brine, dried over Na_2SO_4, and filtered. Solvent was removed under reduced pressure, **S2** was isolated by flash column chromatography (10:90 ethyl acetate:hexanes) as a bright yellow oil (4.33 g, 55% yield).

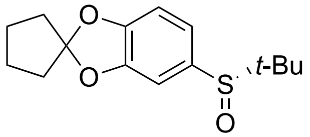

S3

(*S*)-5-(*tert*-Butylsulfinyl)spiro[benzo[*d*][1,3]dioxole-2,1'-cyclopentane] (S3). **S2** (4 mmol) was added to the oven-dried two-neck round bottom flask equipped with a stirbar. The round bottom flask was sealed with a septum and electrical tape. Using a needle attached to a vacuum manifold, the reaction vessel was evacuated (ca. 100 mtorr) and backfilled with argon 3 times. THF (8 mL) was then added via syringe. After stirring for 10 min at rt, reaction mixture was cooled to –78 ºC. *n*BuLi (4.8 mmol, 2.5 M) was added dropwise, and stirred for 30 min at –60 ºC. The reaction was cooled to –78 ºC, and a solution of (*S*)-S-*tert*-butyl 2-methylpropane-2-sulfinothioate (4.4 mmol) in THF (2 mL) was added slowly. The reaction mixture was stirred for 12 h at rt, and quenched with saturated aqueous NH_4Cl. After extraction with ethyl acetate (3 x 30 mL), combined organic phases were washed sequentially with water and brine, and dried over Na_2SO_4. The mixture was filtered, concentrated under reduced pressure, and purified by column chromatography (30:70 ethyl acetate:hexanes) to afford **S3** as a white solid (0.585 g, 52% yield). ^{1}H NMR (300 MHz, $CDCl_3$): δ 7.00 (m, 2H), 6.79 (d, *J* = 8.1 Hz, 1H), 2.16 (m, 4H), 1.88 (m, 4H), 1.16 (s, 9H) ppm. ^{13}C NMR (75 MHz, $CDCl_3$): δ 150.4, 148.2, 132.3, 129.1, 120.8, 107.7, 106.0, 55.9, 37.4 (d, *J* = 9.75 Hz), 23.4, 23.0 ppm. HRMS (ES$^+$) m/z: [M+H]$^+$ Calcd for $C_{15}H_{21}O_3S$ 281.1206; Found 281.1210.

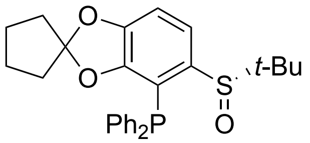

7

(*S*)-(5-(*tert*-Butylsulfinyl)spiro[benzo[*d*][1,3]dioxole-2,1'-cyclopentan]-4-yl) diphenyl phosphane (7): To the oven-dried two-neck round bottom flask equipped with a stirbar **S3** (3.1 mmol) was added. The round bottom flask was sealed with a septum and electrical tape. Using a needle attached to a vacuum manifold, the reaction vessel was evacuated (ca. 100 mtorr) and backfilled with argon 3 times. THF (6 mL) was then added via syringe. The resulting solution was cooled to –78 ºC, LDA (4.6 mmol, 2 M in THF/heptane/ethylbenzene) was added dropwise via syringe. The mixture was allowed to stir for 30 min at –40 ºC, and was cooled to –78 ºC. To the cooled vessel, chlorodiphenylphosphine (5.5 mmol) in THF (2 mL) was added slowly. The mixture was allowed to slowly warm to rt, and stirred overnight. The reaction mixture was quenched with saturated aqueous NH_4Cl, and poured into separatory funnel. The organic layer was extracted with ethyl acetate (3 x 30 mL), washed sequentially with water and brine, dried over Na_2SO_4, and filtered. Solvent was removed under reduced pressure, and **7** was isolated via flash

column chromatography (40:60 ethyl acetate:hexanes) as a white solid (0.945 g, 66% yield). Enantiomeric excess (99.0% ee) was determined by HPLC analysis (IA column, 65:35 CH_3CN:H_2O as eluent). ^{1}H NMR (300 MHz, $CDCl_3$): δ 7.52 (dd, J = 5.4, 2.7 Hz, 1H), 7.41 (m, 7H), 7.25 (m, 3H), 6.96 (d, J = 8.4 Hz, 1H), 1.98 (m, 1H), 1.77 (m, 2H), 1.62 (m, 2H), 1.48 (m, 3H), 1.23 (s, 9H) ppm. ^{13}C NMR (75 MHz, $CDCl_3$): δ 150.6 (d, J = 2.25 Hz), 149.8, 139.0 (d, J = 27 Hz), 136.4 (d, J = 11.25 Hz), 134.4 (d, J = 12 Hz), 133.2, 133.0, 132.8, 128.5, 128.4, 128.1, 127.9, 121.2 (d, J = 7.5 Hz), 117.0 (d, J = 23.25 Hz), 109.9, 58.0, 36.8, 23.7 (d, J = 4.5 Hz), 22.9 (d, J = 2.25 Hz) ppm. ^{31}P NMR (121 MHz, $CDCl_3$) δ -22.60 ppm. HRMS (ES$^+$) m/z: [M+H]$^+$ Calcd for $C_{27}H_{30}O_3PS$ 465.1648; Found 465.1648.

*Procedure for preparation of enantioenriched **3b***

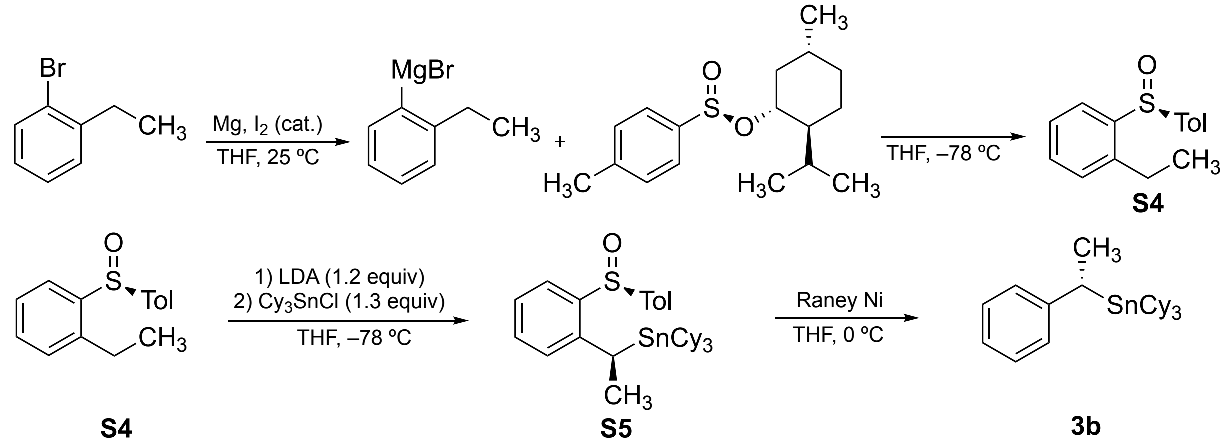

Figure S3. Synthetic sequence for preparation of enantiomerically enriched (*S*)-tricyclohexyl (1-phenylethyl)stannane **3b**.

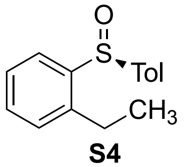

(*S*)-1-Ethyl-2-(*p*-tolylsulfinyl)benzene (S4). On the bench top, Mg turnings (128 mmol, 12.8 equiv) were added to a 100 mL oven-dried round bottom flask, equipped with a stir bar. The flask was sealed with a rubber septum and backfilled 3 times with argon (ca. 100 mtorr). Freshly distilled THF (40 mL) and catalytic amount of I_2 was added to the reaction flask. The reaction flask was cooled to 0 °C and allowed to stir for 10 min. 1-Bromo-2-ethylbenzene (16 mmol) was added dropwise to the reaction solution. The reaction mixture was stirred for 30 min, removed from the ice bath, and allowed to stir at rt for another 2 h. Grignard reagent formation was confirmed by quenching a sample of the reaction

mixture with water and analyzing using gas chromatography. (1*R*,2*S*,5*R*)-(-)-Menthyl (*S*)-*p*-toluenesulfinate (10 mmol) was added to an oven-dried 200 mL round bottom flask with a stirbar. The flask was sealed with a rubber septum and backfilled 3 times with argon (ca. 100 mtorr). Freshly distilled THF (20 mL) was added to the flask, and the reaction mixture was stirred for 5 min. The reaction flask was cooled to –78 ºC. Generated Grignard reagent was added dropwise to the reaction mixture. The flask was kept at –78 ºC for 3 h, and slowly warmed to rt overnight. The reaction solution was quenched with saturated NH_4Cl (40 mL), and extracted with ethyl acetate (3 x 50 mL). The combined organic layers were washed with saturated NaCl, dried over Na_2SO_4, and filtered. Solvent was removed under reduced pressure, and the crude product was purified by column chromatography (15:85 ethyl acetate:hexanes) to afford a pure product (1.99 g, 81% yield). ^1H NMR (300 MHz, $CDCl_3$): δ 7.92 (m, 1H), 7.45 (d, *J* = 8.1 Hz, 2H), 7.35 (m, 2H), 7.18 (m, 3H), 2.84 (m, 1H), 2.64 (m, 1H), 2.28 (s, 3H), 1.08 (t, *J* = 7.5 Hz, 3H) ppm. ^{13}C NMR (75 MHz, $CDCl_3$): δ 142.8, 142.1, 141.9, 141.7, 131.2, 130.0, 129.0, 127.2, 126.2, 124.8, 24.9, 21.5, 14.9 ppm.

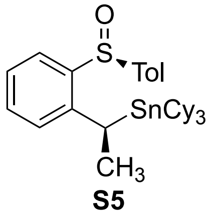

S5

Tricyclohexyl((*S*)-1-(2-((*S*)-*p*-tolylsulfinyl)phenyl)ethyl)stannane (S5).[3] An oven-dried round bottom flask with a stir bar was sealed with a septum and backfilled with argon 3 times. Diisopropylamine (1.7 mL, 12 mmol) was added to the reaction flask followed by freshly distilled THF (24 mL). The reaction was cooled to –78 ºC. *n*BuLi solution (4.3 mL, 10.8 mmol, 2.5 M in hexanes) was added dropwise to the reaction mixture. The reaction was stirred at –78 ºC for 15 min to complete formation of LDA (10.8 mmol). (*S*)-1-Ethyl-2-(*p*-tolylsulfinyl)benzene (**S4**) (8.1 mmol) was (8.1 mmol) was dissolved in THF (5 mL). This solution was added dropwise to the reaction flask containing LDA over 5 min. The reaction turns deep purple upon formation of benzylic anion. The reaction solution was stirred for another 30 min at –78 ºC. Cy_3SnCl (10.5 mmol) was dissolved in THF (10 mL), and this solution was added dropwise to the benzylic anion over 5 min. The reaction mixture turned dark orange, and was allowed to stir for another 30 min. Saturated NH_4Cl (20 mL) was added to the reaction mixture, and the resulting solution was then extracted with ethyl acetate (3 x 20 mL). The combined organic washes were dried over Na_2SO_4, filtered, and concentrated under reduced pressure. The product was purified by recrystallization from MeCN/MeOH to afford a white powder (3.95 g, 60%; 85:15 d.r.). Diastereomers were further separated using a silica column (2:98 ethyl acetate:toluene). ^1H NMR (300 MHz, $CDCl_3$): δ 7.99 (dd, *J* = 6.3 Hz, 1.5 Hz, 1H), 7.47 (d,

J = 8.1 Hz, 2H), 7.40 (m, 1H), 7.28 (m, 4H), 2.82 (q, J = 7.8 Hz, 1H), 2.36 (s, 3H), 1.85 (m, 3H), 1.69 (m, 12H), 1.53 (m, 9H), 1.33 (m, 12H) ppm. ^{13}C NMR (75 MHz, $CDCl_3$): δ 147.5, 143.0, 141.8, 139.8, 130.9, 130.2, 127.7, 126.5, 124.7, 123.9, 32.5, 29.5, 28.3, 27.4, 23.7, 21.6, 18.4 ppm.

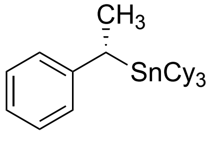

3b

(*S*)-Tricyclohexyl(1-phenylethyl)stannane (3b). Raney Ni (3.3 g, W.R. Grace and Co. Raney® 2400) was transferred to a round bottom flask and slurried in THF (8 mL). The mixture was cooled 0 °C. Raney Ni was allowed to settle and THF was removed with a syringe. This THF wash sequence was performed two more times to remove water from Raney Ni. Finally, THF (8 mL) was added to the Raney Ni. *p*-Tolyl (*S*)-2-((*S*)-1-(tricyclohexylstannyl) ethyl)benzenesulfinate (**S5**) (0.5 mmol) was dissolved in THF (6 mL) and added dropwise over 10 min to the RBF containing Raney Ni. The reaction was allowed to stir overnight. The reaction mixture was filtered through wet celite, and celite was washed 4 times with DCM. The organic layers were combined, and solvent removed under reduced pressure. The crude product was purified by column chromatography (100% hexanes). Enantiomeric excess (97.6% ee) was determined by HPLC analysis (OD-RH column, 75:25 CH_3CN:H_2O as eluent). Over several preparations of **3b**, the enantiomeric excess ranged from 96% ee to 99% ee. ^{1}H NMR (300 MHz, $CDCl_3$): δ 7.24 (m, 2H), 7.11 (d, J = 7.2 Hz, 2H), 7.02 (t, J = 7.5 Hz, 1H), 2.83 (q, J = 8.7 Hz, 1H), 1.82 (m, 6H), 1.65 (m, 11H), 1.53 (m, 9H), 1.31 (m, 10H) ppm. ^{13}C NMR (75 MHz, $CDCl_3$): δ 149.7, 128.3, 126.4, 123.4, 32.5, 29.6, 27.6, 27.4, 26.8, 18.6 ppm. ^{119}Sn (111 MHz, $CDCl_3$): –100.19 ppm. HRMS: Masses for charged ions were not observed under ESI-MS. However, characteristic fragment ions with a loss of a single cyclohexyl group were observed. HRMS (ES$^+$) m/z: $[M - C_6H_{11}]^+$ Calcd for $C_{20}H_{31}Sn$ 391.1446; Found 391.1447. Optical rotation of enantioenriched product $[\alpha]^{25}_D$ (c 0.12, $CHCl_3$) = +14.5 °.

*General procedure for preparation of enantioenriched 1,2-borylstannanes **8**.*

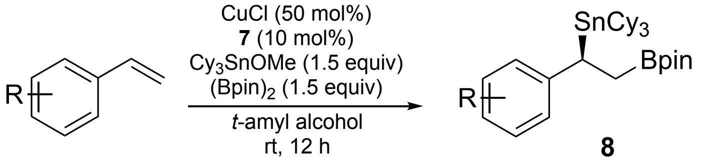

Figure S4. Reaction scheme for the preparation of 1,2-borylstannanes **8**.

On the benchtop, Cy_3SnCl (0.5 mmol) was added to an oven-dried 8 mL screw-top test tube equipped with a stirbar. The test tube was sealed with a screw-top septum and electrical tape. The reaction vessel was evacuated (ca. 100 mtorr) and backfilled with argon 3 times. NaOMe (5 mmol, 25 wt. % in MeOH) was added via syringe at this point. The septum was covered with electrical tape, and the reaction vessel was heated for 12 h at 90 ºC using a heating block. After cooling to rt, the reaction mixture was extracted with hexanes (3 x 10 mL), and the combined hexane layers were concentrated under reduced pressure. The product (white solid) was analyzed by ^{119}Sn NMR ($CDCl_3$) to confirm formation of Cy_3SnOMe (–4.52 ppm), and disappearance of Cy_3SnCl (60.88 ppm). Cy_3SnOMe was used directly in the next step. In the glove box, CuCl (0.16 mmol) and **7** (0.03 mmol) was added to an oven-dried test tube equipped with a stirbar, followed by *t*-amyl alcohol (1.6 mL). The resulting solution was allowed to stir for 30 min at rt inside glove box. After indicated time, $(Bpin)_2$ (0.5 mmol) and Cy_3SnOMe (0.5 mmol) in *t*-amyl alcohol (1.6 mL) was added, the test tube was sealed with septum and parafilm, and taken out of glove box. Styrene derivative (0.33 mmol) was added to the reaction vessel via syringe. The septum was covered with parafilm, and the vessel was allowed to stir at rt for 12 h. The reaction mixture was filtered through celite, and concentrated under reduced pressure. The crude reaction was purified by flash column chromatography (5:95 ethyl acetate:hexanes) to afford pure product.

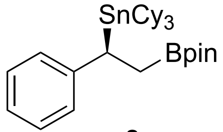

8a

(*S*)-Tricyclohexyl-(1-phenyl-2-(4,4,5,5-tetramethyl-1,3,2-dioxaborolan-2-yl)ethyl)stannane (8a). The general procedure for the synthesis of enantioselective 1,2-borylstannane compounds was employed using styrene (0.33 mmol), $(Bpin)_2$ (0.5 mmol), Cy_3SnOMe (0.5 mmol), CuCl (0.16 mmol) and **7** (0.03 mmol) in *t*-amyl alcohol (3.3 mL) at rt for 12 h. A white solid (110.6 mg, 56%) was isolated via column chromatography (5:95 ethyl acetate:hexanes) as a product. Enantiomeric excess (98.7% ee) was determined by HPLC analysis of β-hydroxystannane (**11c**) obtained by oxidation (NaOH, H_2O_2) of enantiomerically enriched **8a** (see page S12 below). ^1H NMR (300 MHz, $CDCl_3$): δ 7.17 (m, 4H), 6.96 (m, 1H), 2.94 (m, 1H), 1.79 (m, 6H), 1.69 (m, 10H), 1.53 (m, 9H), 1.27 (m, 10H), 1.06 (s, 6H), 0.98 (s, 6H) ppm. ^{13}C NMR (75 MHz, $CDCl_3$): δ 149.3, 128.0, 126,9, 123.3, 83.2, 32.5, 29.7, 27.9, 27.5, 25.1, 24.6. ^{11}B NMR (96 MHz, $CDCl_3$): δ 32.67 ppm. ^{119}Sn (111 MHz, $CDCl_3$): –101.53 ppm. HRMS (ES^+) m/z: $[M + NH_4]^+$ Calcd for $C_{32}H_{57}BO_2NSn$ 618.3510 Found 618.3517. Optical rotation of enantioenriched product $[\alpha]^{25}_D$ (c 0.4, $CHCl_3$) = –8.5 º.

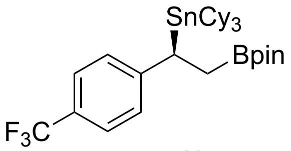
8b

(*S*)-Tricyclohexyl-(2-(4,4,5,5-tetramethyl-1,3,2-dioxaborolan-2-yl)-1-(4-(trifluoromethyl)phenyl)ethyl)stannane (8b). The general procedure for the synthesis of enantioselective 1,2-borylstannane compounds was employed using 4-(trifluoromethyl)styrene (0.33 mmol), $(Bpin)_2$ (0.5 mmol), Cy_3SnOMe (0.5 mmol), CuCl (0.16 mmol) and **7** (0.03 mmol) in *t*-amyl alcohol (3.3 mL) at rt for 12 h. A white solid (91.3 mg, 41% yield) was isolated via column chromatography (5:95 ethyl acetate:hexanes) as a product. Enantiomeric excess (89.8% ee) determined by HPLC analysis of β-hydroxystannane (**S6**) obtained by oxidation (NaOH, H_2O_2) of enantiomerically enriched **8b** (see page S12 below). ^{1}H NMR (300 MHz, $CDCl_3$): δ 7.43 (d, *J* = 7.8 Hz, 2H), 7.20 (d, *J* = 7.2 Hz, 2H), 3.01 (m, 1H), 1.78 (m, 6H), 1.65 (m, 10H), 1.48 (m, 10H), 1.29 (m, 9H), 1.07 (s, 6H), 0.99 (s, 6H) ppm. ^{13}C NMR (75 MHz, $CDCl_3$): δ 154.30, 130.30 (q, *J* = 269 Hz), 126.78, 126.00 (q, *J* = 30.75 Hz), 125.08 (q, *J* = 4 Hz), 83.34, 32.56 (d, *J* = 3 Hz), 29.59, 28.21, 27.37, 25.05, 24.55 ppm. ^{11}B NMR (96 MHz, $CDCl_3$): δ 32.59 ppm. ^{119}Sn (111 MHz, $CDCl_3$): –99.77 ppm. HRMS: Masses for charged ions were not observed under ESI-MS. However, characteristic fragment ions with a loss of a single cyclohexyl group were observed. HRMS (ES^+) m/z: $[M - C_6H_{11}]^+$ Calcd for $C_{27}H_{41}BF_3O_2Sn$ 585.2178; Found 585.2184. Optical rotation of enantioenriched product $[\alpha]^{25}_D$ (c 0.16, $CHCl_3$) = +8.8 º.

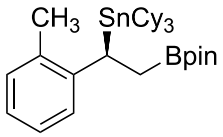
8c

(*S*)-Tricyclohexyl-(2-(4,4,5,5-tetramethyl-1,3,2-dioxaborolan-2-yl)-1-(*o*-tolyl)ethyl)stannane (8c). The general procedure for the synthesis of enantioselective 1,2-borylstannane compounds was employed using 2-methylstyrene (0.33 mmol), $(Bpin)_2$ (0.5 mmol), Cy_3SnOMe (0.5 mmol), CuCl (0.16 mmol) and **7** (0.03 mmol) in *t*-amyl alcohol (3.3 mL) at rt for 12 h. A white solid (85.4 mg, 42% yield) was isolated via column chromatography (5:95 ethyl acetate:hexanes) as a product. Enantiomeric excess (91.0% ee) determined by HPLC analysis of β-hydroxystannane (**S7**) obtained by oxidation (NaOH, H_2O_2) of enantiomerically enriched **8c** (see page S13 below). ^{1}H NMR (300 MHz, $CDCl_3$): δ 7.17 (m, 1H), 7.08 (m, 2H), 6.86 (m, 1H), 3.01 (m, 1H), 2.29 (s, 3H), 1.78 (m, 6H), 1.67 (m, 9H), 1.51 (m, 10H), 1.28 (m, 10H), 1.02 (s, 6H), 0.94 (s, 6H) ppm. ^{13}C NMR (75 MHz, $CDCl_3$): δ 147.6, 133.6, 129.7, 126.9, 126.1, 123.2, 83.0, 32.5, 29.7, 28.2, 27.4,

25.0, 24.5, 20.7 ppm. ^{11}B NMR (96 MHz, $CDCl_3$): δ 32.50 ppm. ^{119}Sn (111 MHz, $CDCl_3$): –97.80 ppm. HRMS: Masses for charged ions were not observed under ESI-MS. However, characteristic fragment ions with a loss of a single cyclohexyl group were observed. HRMS (ES^+) m/z: $[M - C_6H_{11}]^+$ Calcd for $C_{27}H_{44}BO_2Sn$ 531.2460; Found:531.2461. Optical rotation of enantioenriched product $[\alpha]^{25}_D$ (c 0.1, $CHCl_3$) = –14.2 º.

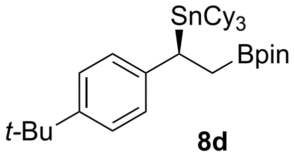

8d

(*S*)-(1-(4-(*tert*-Butyl)phenyl)-2-(4,4,5,5-tetramethyl-1,3,2-dioxaborolan-2-yl)ethyl) tricyclohexylstannane (8d). The general procedure for the synthesis of enantioselective 1,2-borylstannane compounds was employed using 4-*tert*-butylstyrene (0.33 mmol), $(Bpin)_2$ (0.5 mmol), Cy_3SnOMe (0.5 mmol), CuCl (0.16 mmol) and **7** (0.03 mmol) in *t*-amyl alcohol (3.3 mL) at rt for 12 h. A white solid (71.5 mg, 33% yield) was isolated via column chromatography (5:95 ethyl acetate:hexanes) as a product. Enantiomeric excess (92.9% ee) determined by HPLC analysis of β-hydroxystannane (**S8**) obtained by oxidation (NaOH, H_2O_2) of enantiomerically enriched **8d** (see page S13 below). ^1H NMR (300 MHz, $CDCl_3$): δ 7.17 (d, J = 8.7 Hz, 2H), 7.03 (d, J = 8.4 Hz, 2H), 2.90 (m, 1H), 1.75 (m, 6H), 1.66 (m, 10H), 1.51 (m, 11H), 1.25 (s, 9H), 1.20 (m, 8H), 1.06 (s, 6H), 1.00 (s, 6H) ppm. ^{13}C NMR (75 MHz, $CDCl_3$): δ 146.2, 146.1, 126.6, 124.8, 83.1, 34.3, 32.4, 31.7, 29.7, 27.9, 27.4, 24.9, 24.6 ppm. ^{11}B NMR (96 MHz, $CDCl_3$): δ 32.26 ppm. ^{119}Sn (111 MHz, $CDCl_3$): –102.79 ppm. HRMS: Masses for charged ions were not observed under ESI-MS. However, characteristic fragment ions with a loss of a single cyclohexyl group were observed. HRMS (ES^+) m/z: $[M - C_6H_{11}]^+$ Calcd for $C_{30}H_{50}BO_2Sn$ 573.2931; Found: 573.2941. Optical rotation of enantioenriched product $[\alpha]^{25}_D$ (c 0.12, $CHCl_3$) = –26.2 º.

General procedure for oxidation of C–B bond.

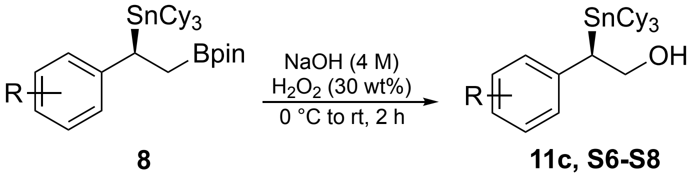

Figure S5. Reaction scheme for oxidation of C–B bond.

On the benchtop, 1,2-borylstannane **8** (0.2 mmol) was dissolved in THF (1 mL). After stirring at rt for 10 min, NaOH (1 mL, 4 M) was added via syringe. The reaction vessel was cooled to 0 ºC, and H_2O_2 (1 mL, 30 wt%) was added dropwise using syringe. The reaction mixture was stirred for 2 h at rt, and quenched with saturated $Na_2S_2O_3$ (aqueous) at 0 ºC. The mixture was extracted with ethyl acetate (3 x 10 mL) and the combined organic layers were dried over Na_2SO_4. After filtration, concentration, and column chromatography (10:90 ethyl acetate:hexanes), pure product was isolated as a bright yellow or transparent oil.

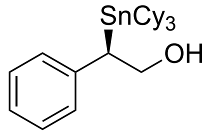

11c

(*S*)-2-Phenyl-2-(tricyclohexylstannyl)ethan-1-ol (11c). General procedure for oxidation of C–B bond was employed using NaOH (1.6 mL, 4 M), H_2O_2 (1.6 mL, 30 wt%), borylstannane **8a** (0.27 mmol) in THF (1.6 mL). A bright yellow oil **11c** (116 mg, 88% yield) was isolated by column chromatography (10:90 ethyl acetate:hexanes). Enantiomeric excess (98.7% ee) was determined by HPLC analysis (IA column, 75:25 CH_3CN:H_2O as eluent). ^1H NMR (300 MHz, $CDCl_3$): δ 7.28 (m, 2H), 7.17 (m, 2H), 7.08 (m, 1H), 4.45 (m, 1H), 4.06 (m, 1H), 3.06 (m, 1H), 1.78 (m, 6H), 1.66 (m, 9H), 1.48 (m, 8H), 1.29 (m, 10H) ppm. ^{13}C NMR (75 MHz, $CDCl_3$): δ 143.5, 128.9, 127.6, 124.6, 64.9, 38.8, 32.5, 29.6, 27.9, 27.3 ppm. ^{119}Sn (111 MHz, $CDCl_3$): –103.83 ppm. HRMS: Masses for charged ions were not observed under ESI-MS. However, characteristic fragment ions with a loss of a single cyclohexyl group were observed. HRMS (ES^+) m/z: $[M - C_6H_{11}]^+$ Calcd for $C_{20}H_{31}OSn$ 407.1395; Found: 407.1370.

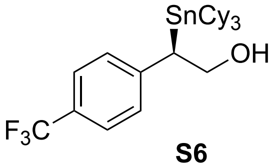

S6

(*S*)-2-(Tricyclohexylstannyl)-2-(4-(trifluoromethyl)phenyl)ethan-1-ol (S6). General procedure for oxidation of C–B bond was employed using NaOH (1.6 mL, 4 M), H_2O_2 (1.6 mL, 30 wt%), borylstannane **8b** (0.27 mmol) in THF (1.6 mL). A light yellow oil **S6** (77.9 mg, 52% yield) was isolated by column chromatography (10:90 ethyl acetate:hexanes). Enantiomeric excess (89.8% ee) was determined by HPLC analysis (OD-RH column, 65:35 CH_3CN:H_2O as eluent). ^1H NMR (300 MHz, $CDCl_3$): δ 7.52 (d, J = 8.1 Hz, 2H), 7.27 (d, J = 5.7 Hz, 2H), 4.46 (m, 1H), 4.15 (m, 1H), 3.13 (m, 1H), 1.75 (m, 6H), 1.67 (m, 9H), 1.52 (m, 9H), 1.30 (m, 9H) ppm. ^{13}C NMR (75 MHz, $CDCl_3$): δ 148.82, 130.03 (q, J = 270.75 Hz), 127.32, 127.15 (q, J = 32 Hz), 125.79 (q, J = 4.5 Hz, 3 Hz), 64.26, 38.95, 32.47, 29.46, 28.18, 27.24 ppm. ^{119}Sn (111 MHz, $CDCl_3$): –101.94

ppm. HRMS: Masses for charged ions were not observed under ESI-MS. However, characteristic fragment ions with a loss of a single cyclohexyl group were observed. HRMS (ES^+) m/z: $[M - C_6H_{11}]^+$ Calcd for $C_{21}H_{30}F_3OSn$ 476.1347; Found: 476.1468.

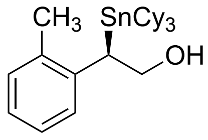
S7

(*S*)-2-(*o*-Tolyl)-2-(tricyclohexylstannyl)ethan-1-ol (S7). General procedure for oxidation of C–B bond was employed using NaOH (1.6 mL, 4 M), H_2O_2 (1.6 mL, 30 wt%), borylstannane **8c** (0.28 mmol) in THF (1.6 mL). A light yellow oil **S7** (70 mg, 50% yield) was isolated by column chromatography (10:90 ethyl acetate:hexanes). Enantiomeric excess (91.0% ee) was determined by HPLC analysis (OD-RH column, 65:35 CH_3CN:H_2O as eluent). ^1H NMR (300 MHz, $CDCl_3$): δ 7.22 (m, 3H), 6.99 (m, 1H), 4.48 (m, 1H), 4.08 (m, 1H), 3.23 (m, 1H), 2.31 (s, 3H), 1.78 (m, 4H), 1.69 (m, 10H), 1.54 (m, 10H), 1.29 (m, 9H) ppm. ^{13}C NMR (75 MHz, $CDCl_3$): δ 141.6, 135.4, 130.8, 126.6, 126.3, 124.4, 65.5, 34.6, 32.5, 29.6, 28.1, 27.4, 20.8 ppm. ^{119}Sn (111 MHz, $CDCl_3$): –101.22 ppm. HRMS (ES^+) m/z: $[M + nNa]^+$ Calcd for $C_{27}H_{44}OSnNa$ 527.2311; Found 527.2429. Optical rotation of enantioenriched product $[\alpha]^{25}_D$ (c 0.14, $CHCl_3$) = +9.2 °.

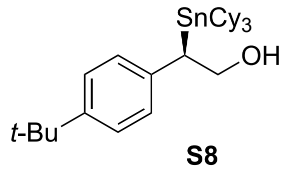
S8

(*S*)–2-(4-(*tert*-Butyl)phenyl)-2-(tricyclohexylstannyl)ethan-1-ol (S8). General procedure for oxidation of C–B bond was employed using NaOH (0.6 mL, 4M), H_2O_2 (0.6 mL, 30 wt%), borylstannane **8d** (0.1 mmol) in THF (0.6 mL). A light yellow oil **S8** (27 mg, 50% yield) was isolated by column chromatography (5:95 ethyl acetate:hexanes). Enantiomeric excess (92.9% ee) was determined by HPLC analysis (OJ-RH column, 65:35 CH_3CN:H_2O as eluent). ^1H NMR (300 MHz, $CDCl_3$): δ 7.28 (m, 1H), 7.25 (m, 1H), 7.09 (d, J = 8.1 Hz, 2H), 4.47 (m, 1H), 4.05 (m, 1H), 3.05 (m, 1H), 1.72 (m, 6H), 1.65 (m, 11H), 1.49 (m, 9H), 1.29 (s, 9H), 1.25 (m, 8H) ppm. ^{13}C NMR (75 MHz, $CDCl_3$): δ 147.5, 139.9, 127.1, 125.7, 64.8, 38.2, 34.4, 32.3, 31.6, 29.6, 27.8, 27.3 ppm. ^{119}Sn (111 MHz, $CDCl_3$): –104.57 ppm. HRMS: Masses for charged ions were not observed under ESI-MS. However, characteristic fragment ions with a loss of a single cyclohexyl group were observed. HRMS (ES^+) m/z: $[M - C_6H_{11}]^+$ Calcd for $C_{24}H_{39}OSn$ 463.2022; Found: 463.2080. Optical rotation of enantioenriched product $[\alpha]^{25}_D$ (c 0.4, $CHCl_3$) = –4.0 °.

General procedure for Suzuki cross-couplings of 1,2-borylstannanes.

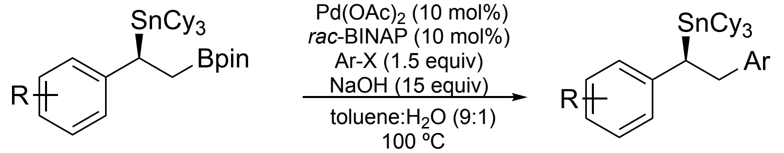

Figure S6. Reaction scheme for Suzuki cross-coupling reactions of **8**.

In an inert-atmosphere glovebox, $Pd(OAc)_2$ (0.01 mmol) and *rac*-BINAP (0.01 mmol) were added to an oven-dried Schlenk tube equipped with a stirbar. Solvent (toluene, 0.5 mL) was then added via syringe. The resulting solution was allowed to stir for 30 min at rt inside the glovebox. 1,2-borylstannane **8** (0.1 mmol), NaOH (1.5 mmol) and toluene (1.3 mL) were then added. The Schlenk tube was sealed with a screw-top Teflon valve and removed from the glovebox. With the sidearm of the Schlenk tube connected to a Schlenk line under a positive pressure of argon, the screw-top Teflon valve was replaced with a rubber septum. Bromobenzene (0.15 mmol) was added to the reaction vessel via a microsyringe at this point, followed by degassed H_2O (0.2 mL). The rubber septum was again replaced with a screw-top Teflon valve and the reaction vessel sealed. The reaction vessel was heated for 12 h at 100 ºC using an oil bath. The reaction mixture was cooled to rt, transferred to a separatory funnel, diluted with water, and extracted with ethyl acetate (3 x 10 mL). The combined organic layers were combined and dried over Na_2SO_4. The solution was filtered and concentrated to provide the crude product. Pure Suzuki cross-coupled product was isolated by column chromatography.

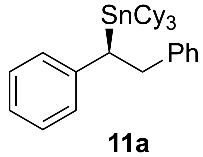

11a

(*S*)-Tricyclohexyl(1,2-diphenylethyl)stannane (**11a**). The general procedure for Suzuki cross-coupling reactions of 1,2-borylstannanes (**8**) was employed using $Pd(OAc)_2$ (0.01 mmol), rac-BINAP (0.01 mmol), bromobenzene (0.15 mmol), borylstannane **8a** (0.1 mmol), NaOH (1.5 mmol) in toluene:H_2O (1.8:0.2 mL). A white solid **11a** (44 mg, 80% yield) was isolated by column chromatography (1:99 ethyl acetate:hexanes). Enantiomeric excess (94.1% ee) was determined by HPLC analysis (OJ-RH column, 75:25 CH_3CN:H_2O as eluent). ^1H NMR (300 MHz, $CDCl_3$): δ 7.20 (m, 9H), 6.98 (m, 1H), 3.48 (m, 1H), 3.29 (dd, J =9.6 Hz, 4.8 Hz, 1H), 3.02 (m, 1H), 1.83 (m, 4H), 1.67 (m, 11H), 1.55 (m, 2H),1.45 (m, 7H), 1.31 (m, 9H) ppm. ^{13}C NMR (75 MHz, $CDCl_3$): δ 146.6, 143.0, 128.6, 128.4, 128.2,

127.7, 125.7, 123.6, 39.3, 35.6, 32.6, 29.7, 28.1, 27.4 ppm. ^{119}Sn (111 MHz, $CDCl_3$): –101.32 ppm. HRMS (ES^+) m/z: $[M+K]^+$ Calcd for $C_{32}H_{46}SnK$ 589.2258; Found 589.2275. Optical rotation of enantioenriched product $[\alpha]^{25}_D$ (c 1.2, $CHCl_3$) = –15.5 º.

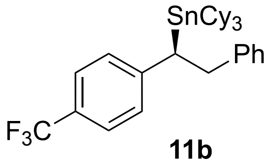

11b

(S)-Tricyclohexyl(2-phenyl-1-(4-(trifluoromethyl)phenyl)ethyl)stannane (**11b**). The general procedure for Suzuki cross-coupling reactions of 1,2-borylstannanes (**8**) was employed using $Pd(OAc)_2$ (0.01 mmol), rac-BINAP (0.01 mmol), bromobenzene (0.15 mmol), borylstannane **8b** (0.1 mmol), NaOH (1.5 mmol) in toluene:H_2O (1.8:0.2 mL). A white solid **11b** (49 mg, 80% yield) was isolated by column chromatography (0.5:99.5 ethyl acetate:hexanes). Enantiomeric excess (90.5% ee) was determined by HPLC analysis (OD-RH column, 65:35 CH_3CN:H_2O as eluent). ^{1}H NMR (300 MHz, $CDCl_3$): δ 7.42 (d, J = 8.1 Hz, 2H), 7.21 (m, 7H), 3.49 (dd, J = 12.0 Hz, 2.7 Hz, 1H), 3.31 (dd, J = 10.2 Hz, 4.2 Hz, 1H), 3.12 (dd, J = 7.2 Hz, 3.9 Hz, 1H), 1.82 (m, 15H), 1.55 (m, 9H), 1.30 (m, 9H) ppm. ^{13}C NMR (75 MHz, $CDCl_3$): δ 151.27, 142.34, 130.18 (q, J = 269.25 Hz), 128.43, 127.38, 126.34 (q, J = 32 Hz), 126.08, 125.33 (q, J = 3.75 Hz), 38.47, 35.60, 32.58 (m), 29.55, 28.32, 27.33 ppm. ^{119}Sn (111 MHz, $CDCl_3$): –99.76 ppm. HRMS: Masses for charged ions were not observed under ESI-MS. However, characteristic fragment ions with a loss of a single cyclohexyl group were observed. HRMS (ES^+) m/z: $[M - C_6H_{11}]^+$ Calcd for $C_{27}H_{34}F_3Sn$ 535.1634; Found: 535.1631. Optical rotation of enantioenriched product $[\alpha]^{25}_D$ (c 0.42, $CHCl_3$) = –14.1 º.

*For characterization data of **11c**, see page S12 above.*

General procedures for Stille cross-coupling reactions of alkylstannanes.

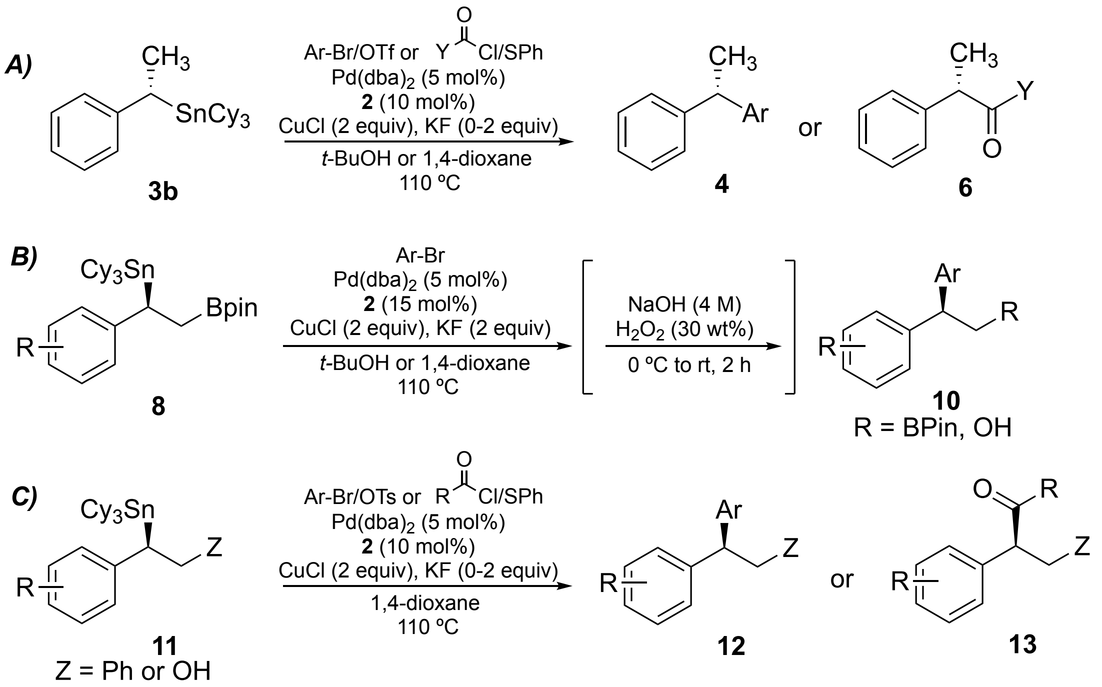

Figure S7. A) Stereospecific Pd-catalyzed Stille cross-coupling reactions using **3b**. B) Stereospecific Pd-catalyzed Stille cross-coupling reactions using 1,2-borylstannanes (**8**). C) Stereospecific Pd-catalyzed Stille cross-coupling reactions of B-derivatized organostannanes (**11**).

General Cross-Coupling Procedure A** – for cross-coupling reactions using **3b: On the benchtop, the electrophile (0.10–0.15 mmol) (if solid), 1,2-borylstannane (0.12–0.18 mmol), $Pd(dba)_2$ (0.0050–0.0075 mmol), JackiePhos (**2**) (0.010–0.015 mmol), CuCl (0.20–0.30 mmol), and anhydrous KF (0.20–0.30 mmol) (only for aryl electrophiles) were added to an oven-dried 8 mL screw-top test tube equipped with a stirbar. The test tube was sealed with a screw-top septum and electrical tape. Using a needle attached to a vacuum manifold, the reaction vessel was evacuated (ca. 100 mtorr) and backfilled with argon 3 times. If the electrophile was liquid, it was added to the reaction vessel using a microsyringe at this point. *t*-Butanol or 1,4-dioxane (1.0–1.5 mL) was then added via syringe. The septum was covered with electrical tape, and the reaction vessel was heated at 110 ºC for 18 h using a heating block. Following completion of the reaction, the cooled reaction mixture was diluted with water (5 mL) and extracted with ethyl acetate (3 x 10 mL). The combined organic layers were washed with brine (10 mL) and dried over Na_2SO_4. The solution

was filtered, analyzed by GC (if applicable), concentrated under reduced pressure, and purified by column chromatography.

***General Cross-Coupling Procedure B** – for cross-coupling reactions using 1,2-borylstannanes (**8**):* On the benchtop, the aryl electrophile (0.13 mmol) (if solid), 1,2-borylstannane **8** (0.15 mmol), $Pd(dba)_2$ (0.0065 mmol), JackiePhos (**2**) (0.013 mmol), CuCl (0.26 mmol), and *anhydrous* KF (0.26 mmol) were added to an oven-dried Schlenk tube equipped with a stirbar. The Schlenk tube was sealed with a rubber septum, and the sidearm was connected to Schlenk line. The reaction vessel was evacuated (ca. 100 mtorr) and backfilled with argon 3 times. If the electrophile was liquid, it was added to the reaction vessel via a microsyringe at this point. *t*-Butanol or 1,4-dioxane (1.3 mL) was then added using syringe. Under a positive pressure of argon, the rubber septum was replaced with a screw-top Teflon valve, and the Schlenk tube was sealed. The reaction vessel was heated for 12 h at 110 °C using an oil bath. The reaction mixture was cooled to rt and transferred to a separatory funnel. The reaction mixture was diluted with water and extracted with ethyl acetate (3 x 10 mL). The organics layers were combined and dried over Na_2SO_4. If the organoborane product was desired, the solution was filtered, concentrated, and purified using column chromatography to provide the desired product. If the alcohol from oxidation of the C–B bond was desired, an ^{1}H NMR yield of the crude 1,2-borylstannane cross-coupled product was obtained at this point using mesitylene as internal standard. This was followed by the general procedure for oxidation of C–B bond, and purification of the compound.

***General Cross-Coupling Procedure C** – for cross-coupling reactions using B-derivatized organostannanes (**11**):* On the benchtop, the electrophile (aryl bromide/acyl chloride 0.13 mmol) (if solid), enantioenriched benzylic 1,2-borylstannane (**11**) (0.15 mmol), $Pd(dba)_2$ (0.0065 mmol), JackiePhos (**2**) (0.013 mmol), CuCl (0.26 mmol), and *anhydrous* KF (0.26 mmol) (only for aryl electrophiles) were added to an oven-dried Schlenk tube equipped with a stirbar. The Schlenk tube was sealed with a rubber septum, and the sidearm was connected to Schlenk line. The reaction vessel was evacuated (ca. 100 mtorr) and backfilled with argon 3 times. If the electrophile was liquid, it was added to the reaction vessel via microsyringe at this point. 1,4-Dioxane (1.3 mL) was then added via syringe. Under a positive pressure of argon, the rubber septum was replaced with a screw-top Teflon valve, and the Schlenk tube was sealed. The reaction vessel was heated for 12 h

at 110 °C using an oil bath. The reaction mixture was cooled to rt and transferred to a separatory funnel. The reaction mixture was diluted with water and extracted with ethyl acetate (3 x 10 mL). The organic layers were combined and dried over Na_2SO_4. The solution was filtered, concentrated, and purified using column chromatography to provide the desired product.

4. Cross-Coupling Product Characterization Data

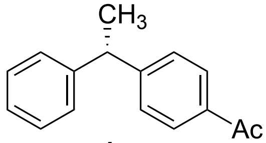

4a

(*S*)-1-(4-(1-Phenylethyl)phenyl)ethan-1-one (**4a**).[4] General cross-coupling procedure **A** was employed using 4-bromoacetophenone (0.15 mmol), benzylic tricyclohexylstannane **3b** (0.18 mmol, 91% ee), $Pd(dba)_2$ (0.0075 mmol), JackiePhos (0.015 mmol), CuCl (0.3 mmol), KF (0.3 mmol) in *t*-BuOH (1.5 mL). A transparent oil **4a** (26.5 mg, 80% yield) was isolated by column chromatography (5:95 ethyl acetate:hexanes). Enantiomeric excess (88.3% ee; 97% es) was determined by HPLC analysis [OJ-RH column, 90:10 (5% CH_3CN in MeOH):H_2O as eluent]. ^1H NMR (300 MHz, $CDCl_3$): δ 7.90 (d, *J* = 8.1 Hz, 2H), 7.33 (m, 4H), 7.22 (m, 3H), 4.25 (q, *J* = 7.2 Hz, 1H), 2.57 (s, 3H), 1.68 (d, *J* = 6.9 Hz, 3H) ppm. ^{13}C NMR (75 MHz, $CDCl_3$): δ 197.9, 152.2, 145.5, 135.4, 128.8, 128.7, 128.0, 127.8, 126.6, 45.0, 26.8, 21.8 ppm. Optical rotation of enantioenriched product $[\alpha]^{25}_D$ (c 0.2, $CHCl_3$) = –3.0 °.

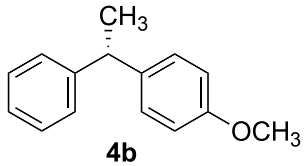

4b

(*S*)-1-Methoxy-4-(1-phenylethyl)benzene (**4b**).[5] General cross-coupling procedure **A** was employed using 4-bromoanisole (0.1 mmol), benzylic tricyclohexylstannane **3b** (0.12 mmol, 99% ee), $Pd(dba)_2$ (0.005 mmol), JackiePhos (0.01 mmol), CuCl (0.2 mmol), KF (0.2 mmol) in 1,4-dioxane (1 mL). A transparent oil **4b** (16 mg, 78% yield) was isolated by column chromatography (1:99 ethyl acetate:hexanes). Enantiomeric excess (97% ee; 98% es) was determined by HPLC analysis [IA column, 85:15 (5% CH_3CN in MeOH):H_2O as eluent]. ^1H NMR (300 MHz, $CDCl_3$): δ 7.32 (m, 2H), 7.23 (m, 5H), 6.87 (d, *J* = 8.7 Hz, 3.0 Hz, 2H), 4.16 (q, *J* = 7.2 Hz, 1H), 3.79 (s, 3H), 1.64 (d, *J* = 7.2 Hz, 3H) ppm. ^{13}C NMR (75 MHz, $CDCl_3$): δ 158.1, 146.9, 138.8, 128.7, 128.5, 127.7, 126.1, 113.9, 55.5, 44.1, 22.3 ppm. Optical rotation of enantioenriched product $[\alpha]^{25}_D$ (c 0.1, $CHCl_3$) = –21.8 °.

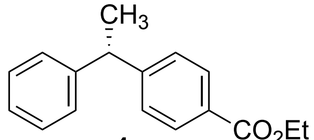

4c

Ethyl (*S*)-4-(1-phenylethyl)benzoate (**4c**).[6] General cross-coupling procedure **A** was employed using ethyl 4-bromobenzoate (0.1 mmol), benzylic tricyclohexylstannane **3b** (0.12 mmol, 99% ee), Pd(dba)$_2$ (0.005 mmol), JackiePhos (0.01 mmol), CuCl (0.2 mmol), KF (0.2 mmol) in 1,4-dioxane (1 mL). A transparent oil **4c** (21.7 mg, 84% yield) was isolated by column chromatography (2:98 ethyl acetate:hexanes). Enantiomeric excess (98% ee; 99% es) was determined by HPLC [OJ-RH column, 80:20 (5% CH_3CN in MeOH):H_2O as eluent]. ^{1}H NMR (300 MHz, $CDCl_3$): δ 7.98 (d, *J* = 8.1 Hz, 2H), 7.32 (m, 4H), 7.22 (m, 3H), 4.40 (q, *J* = 6 Hz, 2H), 4.24 (q, *J* = 6 Hz, 1H), 1.67 (d, *J* = 7.2 Hz, 3H), 1.40 (t, *J* = 7.2 Hz, 3H) ppm. ^{13}C NMR (75 MHz, $CDCl_3$): δ 166.8, 151.8, 145.7, 129.9, 128.7, 127.8, 127.8, 126.5, 60.9, 45.1, 21.9, 14.6 ppm. Optical rotation of enantioenriched product $[\alpha]^{25}_D$ (c 0.12, $CHCl_3$) = –12.1 º.

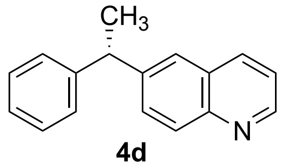

4d

(*S*)-6-(1-Phenylethyl)quinoline (**4d**).[7] General cross-coupling procedure **A** was employed using 5-quinolyl trifluoromethanesulfonate (0.1 mmol), benzylic tricyclohexylstannane **3b** (0.12 mmol, 96% ee), Pd(dba)$_2$ (0.005 mmol), JackiePhos (0.01 mmol), CuCl (0.2 mmol), KF (0.2 mmol) in 1,4-dioxane (1 mL). A transparent oil **4d** (16.3 mg, 70% yield) was isolated by column chromatography (25:75 ethyl acetate:hexanes). Enantiomeric excess (94% ee; 98% es) was determined by HPLC analysis [OJ-RH column, 80:20 (5% CH_3CN in MeOH):H_2O as eluent]. ^{1}H NMR (300 MHz, $CDCl_3$): δ 8.87 (s, 1H), 8.12 (d, *J* = 8.4 Hz, 1H), 8.02 (d, *J* = 8.7 Hz, 1H), 7.66 (s, 1H), 7.58 (d, *J* = 7.8 Hz, 1H), 7.40 (m, 1H), 7.28 (m, 3H), 7.24 (m, 2H), 4.38 (q, J = 7.2 Hz, 1H), 1.76 (d, J = 7.2 Hz, 3H) ppm. ^{13}C NMR (75 MHz, $CDCl_3$): δ 150.1, 147.4, 145.9, 144.9, 136.1, 130.6, 129.6, 129.0, 128.7, 127.9, 126.5, 125.3, 121.3, 44.9, 21.9 ppm. Optical rotation of enantioenriched product $[\alpha]^{25}_D$ (c 0.5, $CHCl_3$) = +22.6 º.

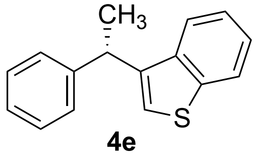

4e

(*S*)-3-(1-Phenylethyl)benzo[*b*]thiophene (**4e**).[8] General cross-coupling procedure **A** was employed using 3-bromobenzothiophene (0.1 mmol), benzylic tricyclohexylstannane **3b** (0.12 mmol, 99% ee), Pd(dba)$_2$ (0.005 mmol), JackiePhos (0.01 mmol), CuCl (0.2 mmol), KF (0.2 mmol) in *t*-BuOH (1 mL). A transparent oil **4e** (16.9 mg, 71% yield) was isolated by column chromatography (0.5:99.5 ethyl acetate:hexanes). Enantiomeric excess (99% ee; >99% es) was determined by HPLC analysis [OJ-

RH column, 85:15 (5% CH_3CN in MeOH):H_2O as eluent]. ^{1}H NMR (300 MHz, $CDCl_3$): δ 7.86 (d, *J* = 7.8 Hz, 1H), 7.58 (d, *J* = 7.2 Hz, 1H), 7.32 (m, 8H), 4.50 (q, *J* = 7.2 Hz, 1H), 1.76 (d, *J* = 7.2 Hz, 3H) ppm. ^{13}C NMR (75 MHz, $CDCl_3$): δ 145.6, 140.8, 140.6, 138.7, 128.7, 127.5, 126.4, 124.3, 123.9, 122.9, 122.7, 121.6, 39.7, 22.6 ppm. Optical rotation of enantioenriched product $[\alpha]^{25}_D$ (c 0.4, $CHCl_3$) = +48.6 °.

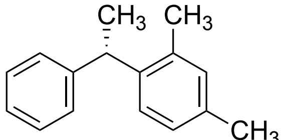

4f

(*S*)-2,4-Dimethyl-1-(1-phenylethyl)benzene (4f).[9] General cross-coupling procedure **A** was employed using 1-bromo-2,4-dimethylbenzene (0.1 mmol), benzylic tricyclohexylstannane **3b** (0.12 mmol, 99% ee), $Pd(dba)_2$ (0.005 mmol), JackiePhos (0.01 mmol), CuCl (0.2 mmol), KF (0.2 mmol) in 1,4-dioxane (1 mL). A transparent oil **4f** (10.5 mg, 50% yield) was isolated by column chromatography (1:99 ethyl acetate:hexanes). Enantiomeric excess (98% ee; 98% es) was determined by HPLC analysis [IA column, 75:25 (5% CH_3CN in MeOH):H_2O as eluent]. ^{1}H NMR (300 MHz, $CDCl_3$): δ 7.30 (m, 2H), 7.17 (m, 4H), 7.03 (m, 1H), 6.96 (m, 1H), 4.32 (q, *J* = 7.2 Hz, 1H), 2.30 (s, 3H), 2.20 (s, 3H), 1.61 (d, *J* = 7.2 Hz, 3H) ppm. ^{13}C NMR (75 MHz, $CDCl_3$): δ 146.7, 141.2, 136.1, 135.7, 131.5, 128.5, 127.8, 126.9, 125.9, 40.9, 22.4, 21.1, 19.9 ppm. Optical rotation of enantioenriched product $[\alpha]^{25}_D$ (c 0.1, $CHCl_3$) = +17.3 °.

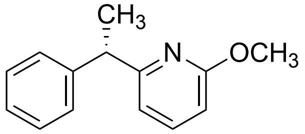

4g

(*S*)-2-Methoxy-6-(1-phenylethyl)pyridine (4g).[10] General cross-coupling procedure **A** was employed using 2-bromo-6-methoxypyridine (0.1 mmol), benzylic tricyclohexylstannane **3b** (0.12 mmol, 98% ee), $Pd(dba)_2$ (0.005 mmol), JackiePhos (0.01 mmol), CuCl (0.2 mmol), KF (0.2 mmol) in *t*-BuOH (1 mL). A transparent oil **4g** (17 mg, 80% yield) was isolated by column chromatography (40:60 ethyl acetate:hexanes). Enantiomeric excess (93% ee; 95% es) was determined by HPLC analysis [OJ-RH column, 85:15 (5% CH_3CN in MeOH):H_2O as eluent]. ^{1}H NMR (300 MHz, $CDCl_3$): δ 7.46 (m, 1H), 7.38 (m, 2H), 7.33 (m, 2H), 7.23 (m, 1H), 6.70 (d, *J* = 6.6 Hz, 1H), 6.55 (d, *J* = 8.4 Hz, 1H), 4.20 (q, *J* = 6.9 Hz 1H), 3.95 (s, 3H), 1.71 (d, *J* = 7.5 Hz, 3H) ppm. ^{13}C NMR (75 MHz, $CDCl_3$): δ 163.7, 162.8, 145.6, 138.9, 128.4, 127.9, 126.4, 114.6,

107.8, 53.3, 47.3, 21.0 ppm. Optical rotation of enantioenriched product $[\alpha]^{25}_D$ (c 0.2, $CHCl_3$) = +23.8 º.

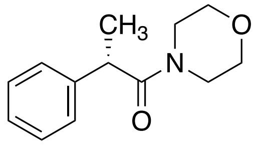
6a

(*S*)-1-Morpholino-2-phenylpropan-1-one (**6a**).[11] General cross-coupling procedure **A** was employed using 4-morpholinecarbonyl chloride (0.1 mmol), benzylic tricyclohexylstannane **3b** (0.12 mmol, 99% ee), $Pd(dba)_2$ (0.005 mmol), JackiePhos (0.01 mmol), CuCl (0.2 mmol) in 1,4-dioxane (1 mL). A transparent oil **6a** (11.38 mg, 52% yield) was isolated by column chromatography (40:60 ethyl acetate:hexanes). Enantiomeric excess (97.5% ee; 98% es) was determined by HPLC analysis [IA column, 70:30 (5% CH_3CN in MeOH):H_2O as eluent]. ^{1}H NMR (300 MHz, $CDCl_3$): δ 7.34 (m, 2H), 7.25 (m, 3H), 3.87 (m, 2H), 3.68 (m, 1H), 3.56 (m, 5H), 3.12 (m, 1H), 1.46 (d, *J* = 7.2 Hz, 3H) ppm. ^{13}C NMR (75 MHz, $CDCl_3$): δ 172.3, 142.0, 129.2, 127.3, 127.1, 67.0, 66.4, 46.1, 43.5, 42.5, 20.9 ppm. Optical rotation of enantioenriched product $[\alpha]^{25}_D$ (c 0.5, $CHCl_3$) = +69.8 º.

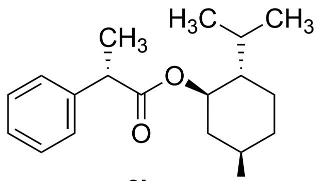
6b

(1*R*,2*S*,5*R*)-2-Isopropyl-5-methylcyclohexyl (*S*)-2-phenylpropanoate (**6b**). General Stille cross-coupling procedure **A** was employed using (1*R*)-(-)-menthyl chloroformate (0.15 mmol), benzylic tricyclohexylstannane **3b** (0.18 mmol, 98% ee), $Pd(dba)_2$ (0.0075 mmol), JackiePhos (0.015 mmol), CuCl (0.3 mmol) in toluene (1.5 mL). *This reaction was heated to 130 ºC instead of 110 ºC.* A transparent oil **6b** (26.5 mg, 80% yield) was isolated by column chromatography (15:85 ethyl acetate:hexanes). The dr value was determined using ^{1}H NMR. ^{1}H NMR (300 MHz, C_6D_6): δ 7.28 (d, *J* = 7.5 Hz, 2H), 7.14 (m, 2H), 7.04 (m, 1H), 4.84 (m, 1H), 3.64 (q, *J* = 3 Hz, 0.02H from other diastereomer), 3.59 (q, *J* = 7.2 Hz, 1H), 2.10 (m, 1H), 1.58 (m, 1H), 1.47 (d, *J* = 7.5 Hz, 3H), 1.42 (m, 2H), 1.26 (m, 5H), 0.76 (d, *J* = 6.6 Hz, 3H), 0.71 (d, *J* = 6.9 Hz, 3H), 0.64 (d, *J* = 6.9 Hz, 3H) ppm. ^{13}C NMR (75 MHz, C_6D_6): δ 173.6, 141.7, 128.8, 127.9, 127.2, 74.2, 47.6, 46.3, 41.4, 34.6, 31.5, 25.9, 23.6, 22.1, 20.9, 18.7, 16.2 ppm. HRMS (ES$^+$) m/z: $[M+H]^+$ Calcd for $C_{19}H_{28}O_2$ 289.2162; Found 289.2162.

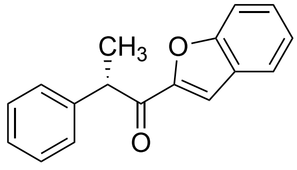

6c

(*S*)-1-(Benzofuran-2-yl)-2-phenylpropan-1-one (**6c**). General Stille cross-coupling procedure **A** was employed using phenyl benzofuran-2-carbothioate (0.15 mmol), benzylic tricyclohexylstannane **3b** (0.18 mmol, 97% ee), Pd(dba)$_2$ (0.0075 mmol), JackiePhos (0.015 mmol), CuCl (0.3 mmol, KF (0.3 mmol) in *t*-BuOH (1.5 mL). A transparent oil **6c** (17.6 mg, 47% yield) was isolated by column chromatography (5:95 ethyl acetate:hexanes). Enantiomeric excess (96% ee; 99% es) was determined by HPLC analysis [OJ-RH column, 85:15 (5% CH_3CN in MeOH):H_2O as eluent]. ^{1}H NMR (300 MHz, $CDCl_3$): δ 7.66 (d, *J* = 8.1 Hz, 1H), 7.56 (m, 8H), 7.24 (m, 1H), 4.68 (q, *J* = 7.2 Hz, 1H), 1.60 (d, *J* = 6.6 Hz, 3H) ppm. ^{13}C NMR (75 MHz, $CDCl_3$): δ 191.5, 155.7, 152.3, 140.8, 135.3, 129.1, 128.4, 128.2, 127.4, 124.0, 123.4, 113.8, 112.6, 48.6, 18.6 ppm. HRMS (ES$^+$) m/z: [M+H]$^+$ Calcd for $C_{17}H_{14}O_2$ 251.1067; Found 251.1071. Optical rotation of enantioenriched product $[\alpha]^{25}_D$ (c 0.25, $CHCl_3$) = –28.0 º.

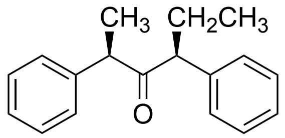

6d

(2R,4S)-2,4-diphenylhexan-3-one (6d). General cross-coupling procedure B was employed using (*S*)-S-phenyl-2-phenylbutanethioate (0.2 mmol), benzylic tricyclohexylstannane (*R*)-**3b** (0.3 mmol), Pd$_2$dba$_3$ (10 mol %), JackiePhos (24 mol %), CuCl (0.4 mmol), KF (0.4 mmol) in toluene (1 mL). A colorless oil **6d** (41.9 mg, 83% yield) was isolated by column chromatography (5:95 ethyl acetate:hexanes). Diastereomeric ratio (19:1 dr) was determined by ^{1}H NMR analysis. ^{1}H NMR (300 MHz, $CDCl_3$): δ 7.32 (m, 6H), 7.17 (m, 4H), 3.74 (q, *J* = 6.9 Hz, 1H), 3.53 (t, *J* = 7.3 Hz), 1.93 (m, 1H), 1.63 (m, 1H), 1.25 (d, *J* = 6.9 Hz, 3H), 0.57 (t, *J* = 7.4 Hz, 3H) ppm. ^{13}C NMR (75 MHz, $CDCl_3$): δ 209.8, 140.7, 139.5, 129.10, 129.05, 128.9, 128.6, 128.3, 127.3, 58.7, 51.5, 25.6, 18.1, 11.9 ppm. HRMS (ES$^+$) m/z: [M+H]$^+$ Calcd for $C_{18}H_{20}O$ 253.1592; Found 253.1593.

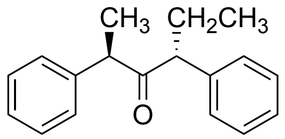

6e

(2R,4R)-2,4-diphenylhexan-3-one (6e). General cross-coupling procedure B was employed using (*R*)-S-phenyl-2-phenylbutane thioate (0.2 mmol), benzylic tricyclohexylstannane (*R*)-**3b** (0.3 mmol), Pd$_2$dba$_3$ (10 mol %), JackiePhos (24 mol %), CuCl (0.4 mmol), KF (0.4 mmol) in toluene (1 mL). A colorless oil **6e** (42.4 mg, 84% yield) was isolated by column chromatography (5:95 ethyl acetate:hexanes). Diastereomeric ratio (18:1 d.r.) was determined by ^{1}H NMR analysis.

^{1}H NMR (300 MHz, $CDCl_3$): δ 7.16 (m, 6H), 6.96 (m, 4H), 3.83 (q, J = 6.9 Hz, 1H), 3.64 (t, J = 7.5 Hz), 2.01 (m, 1H), 1.68 (m, 1H), 1.37 (d, J = 7.1 Hz, 3H), 0.79 (t, J = 7.3 Hz, 3H) ppm. ^{13}C NMR (75 MHz, $CDCl_3$): δ 211.4, 140.0, 138.9, 128.57, 128.53, 128.45, 128.35, 127.0, 126.9, 59.5, 53.0, 26.6, 18.0, 12.4 ppm.

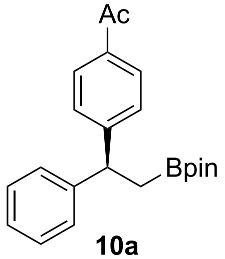

10a

(*S*)-1-(4-(1-phenyl-2-(4,4,5,5-tetramethyl-1,3,2-dioxaborolan-2-yl)ethyl)-phenyl)ethan-1-one (**10a**). General cross-coupling procedure **B** was employed using 4-bromoacetophenone (0.1 mmol), 1,2-borylstannane **8a** (0.12 mmol, 98% ee), $Pd(dba)_2$ (0.005 mmol), JackiePhos (0.015 mmol), CuCl (0.2 mmol), KF (0.2 mmol) in 1,4-dioxane (1 mL) at 110 ºC. A bright yellow oil (**10a**) (25 mg, 73% yield) was isolated by column chromatography (10:90 to 20:80 ethyl acetate:hexanes). Enantiomeric excess (95.6% ee, 97% es) determined by HPLC analysis of alcohol **10b** obtained by oxidation (NaOH, H_2O_2) of enantiomerically enriched **10a**. ^{1}H NMR (300 MHz, $CDCl_3$): d 7.87 (d, J = 8.1 Hz, 2H), 7.37 (d, J = 8.1 Hz, 2H), 7.24 (m, 4H), 7.17 (m, 1H), 4.37 (t, J = 8.4 Hz, 1H), 2.55 (s, 3H), 1.62 (d, J = 8.1 Hz, 2H), 1.06 (s, 12H) ppm. ^{13}C NMR (75 MHz, $CDCl_3$): d 198.1, 152.6, 145.8, 135.3, 128.7, 128.6, 128.1, 127.9, 126.5, 83.5, 46.7, 26.8, 24.8 ppm. ^{11}B NMR (96 MHz, $CDCl_3$): d 32.09 ppm. Optical rotation of enantioenriched product $[\alpha]^{25}_D$ (c 1.0, $CHCl_3$) = +15.0 º.

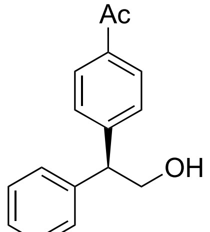

10b

(*S*)-1-(4-(2-Hydroxy-1-phenylethyl)phenyl)ethan-1-one (**10b**). General Stille cross-coupling procedure **B** was employed using 4-bromoacetophenone (0.13 mmol), 1,2-borylstannane **8a** (0.15 mmol, 98% ee), $Pd(dba)_2$ (0.0065 mmol), JackiePhos (0.019 mmol), CuCl (0.26 mmol), KF (0.26 mmol) in 1,4-dioxane (1.3 mL) at 110 ºC. Crude product was obtained [82% yield by ^{1}H NMR]. General procedure for oxidation of C–B bond was employed using NaOH (0.65 mL, 4M), H_2O_2 (0.65 mL, 30 wt%) in THF (0.65 mL). A transparent oil **10b** (27.3 mg, 87% yield) was isolated by column chromatography (20:80 to 50:50 ethyl acetate:hexanes). Enantiomeric excess (95.6% ee; 97% es) was determined by HPLC analysis (IA column, 55:45 CH_3CN:H_2O as eluent). ^{1}H NMR (300 MHz, $CDCl_3$): δ 7.93 (d, J = 8.4 Hz, 2H), 7.39 (m, 4H), 7.27 (m, 3H), 4.30 (m, 3H), 2.57 (s, 3H), 1.63 (s, 1H, OH) ppm. ^{13}C NMR (75 MHz, $CDCl_3$): δ 197.9, 147.3, 140.8, 135.9, 129.1, 128.9, 128.8, 128.5, 127.4, 65.9, 53.7, 26.8 ppm. HRMS (ES^+) m/z: $[M+H]^+$ Calcd for $C_{16}H_{17}O_2$

241.1223; Found: 241.1223. Optical rotation of enantioenriched product $[\alpha]^{25}_{D}$ (c 0.1, $CHCl_3$) = +6 º.

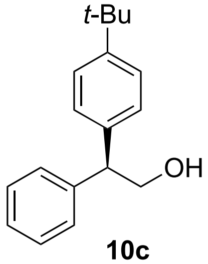

10c

(*S*)-2-(4-(*tert*-Butyl)phenyl)-2-phenylethan-1-ol (**10c**). General Stille cross-coupling procedure **B** was employed using 1-bromo-4-*tert*–butylbenzene (0.1 mmol), 1,2-borylstannane **8a** (0.12 mmol, 98% ee), $Pd(dba)_2$ (0.005 mmol), JackiePhos (0.015 mmol), CuCl (0.2 mmol), KF (0.2 mmol) in 1,4-dioxane (1 mL) at 110 ºC. Crude product was obtained [72% yield by ^1H NMR]. General procedure for oxidation of C–B bond was employed using NaOH (0.65 mL, 4 M), H_2O_2 (0.65 mL, 30 wt%) in THF (0.65 mL). A white solid **10c** (15.1 mg, 52% yield) was isolated by column chromatography (30:70 ethyl acetate:hexanes). Enantiomeric excess (91.9% ee; 94% es) was determined by HPLC analysis (OJ-RH column, 60:40 CH_3CN:H_2O as eluent). ^1H NMR (300 MHz, $CDCl_3$): δ 7.35 (m, 6H), 7.23 (m, 3H), 4.19 (m, 3H), 1.49 (s, 1H, OH), 1.30 (s, 9H) ppm. ^{13}C NMR (75 MHz, $CDCl_3$): δ 149.9, 141.8, 138.4, 128.9, 128.5, 128.1, 127.0, 125.9, 66.4, 53.5, 34.6, 31.5 ppm. HRMS: Masses for charged ions were not observed under ESI-MS. However, characteristic fragment ions with a loss of a hydroxyl group were observed. HRMS (ES^+) m/z: $[M-OH]^+$ Calcd for $C_{18}H_{21}$ 237.1638; Found: 237.1638. Optical rotation of enantioenriched product $[\alpha]^{25}_{D}$ (c 0.2, $CHCl_3$) = +12.0 º.

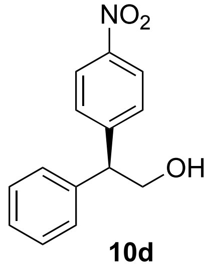

10d

(*S*)-2-(4-Nitrophenyl)-2-phenylethan-1-ol (**10d**). General Stille cross-coupling procedure **B** was employed using 1-bromo-4-nitrobenzene (0.14 mmol), 1,2-borylstannane **8a** (0.17 mmol, 98% ee), $Pd(dba)_2$ (0.007 mmol), JackiePhos (0.021 mmol), CuCl (0.28 mmol), KF (0.28 mmol) in 1,4-dioxane (1.4 mL) at 110 ºC. Crude product was obtained [61% yield by ^1H NMR]. General procedure for oxidation of C–B bond was employed using NaOH (0.65 mL, 4 M), H_2O_2 (0.65 mL, 30 wt%) in THF (0.65 mL). A reddish yellow oil **10d** (17.6 mg, 52% yield) was isolated by column chromatography (15:85 to 40:60 ethyl acetate:hexanes). Enantiomeric excess (91.3% ee; 93% es) was determined by HPLC analysis (OJ-RH column, 40:60 CH_3CN:H_2O with 0.1% phosphoric acid as eluent). ^1H NMR (300 MHz, $CDCl_3$): δ 8.17 (m, 2H), 7.45 (d, J = 8.4 Hz, 2H), 7.36 (m, 3H), 7.23 (m, 2H), 4.33 (m, 1H), 4.21 (m, 2H), 1.57 (s, 1H, OH) ppm. ^{13}C NMR (75 MHz, $CDCl_3$): δ 149.6, 147.0, 140.1, 129.5, 129.3, 128.5, 127.7, 124.0, 65.8, 53.5 ppm. HRMS (ES^+)

m/z: $[M+H]^+$ Calcd for $C_{14}H_{14}NO_3$ 244.0968; Found: 244.0968. Optical rotation of enantioenriched product $[\alpha]^{25}_D$ (c 0.8, $CHCl_3$) = +8.0 º.

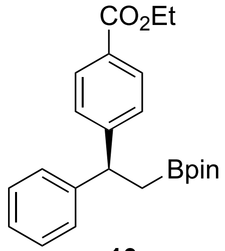

10e

Ethyl (*S*)-4-(1-phenyl-2-(4,4,5,5-tetramethyl-1,3,2-dioxaborolan-2-yl)ethyl)benzoate (**10e**). General Stille cross-coupling procedure **C** was employed using ethyl 4-bromobenzoate (0.1 mmol), 1,2-borylstannane **8a** (0.12 mmol, 98% ee), $Pd(dba)_2$ (0.005 mmol), JackiePhos (0.015 mmol), CuCl (0.2 mmol), KF (0.2 mmol) in 1,4-dioxane (1 mL) at 110 ºC. A transparent oil **10e** (27 mg, 71% yield) was isolated by column chromatography (10:90 ethyl acetate:hexanes). Enantiomeric excess (96.3% ee; 98% es) determined by HPLC analysis of alcohol **10f** obtained by oxidation (NaOH, H_2O_2) of enantiomerically enriched **10e**. ^1H NMR (300 MHz, $CDCl_3$): δ 7.97 (d, *J* = 8.4 Hz, 2H), 7.37 (d, *J* = 8.4 Hz, 2H), 7.28 (m, 5H), 4.40 (m, 3H), 1.64 (d, *J* = 7.8 Hz, 2H), 1.41 (t, *J* = 7.5 Hz, 3H), 1.08 (s, 12H) ppm. ^{13}C NMR (75 MHz, $CDCl_3$): δ 166.9, 152.1, 145.9, 129.8, 128.6, 128.4, 127.9, 126.4, 83.5, 60.9, 46.7, 24.8, 14.5 ppm. HRMS (ES^+) m/z: $[M+H]^+$ Calcd for $C_{23}H_{30}BO_4$ 381.2236; Found: 381.2237. Optical rotation of enantioenriched product $[\alpha]^{25}_D$ (c 0.12, $CHCl_3$) = +13.3 º.

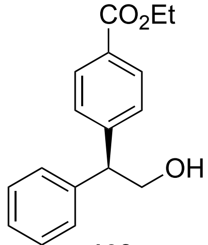

10f

Ethyl (*S*)-4-(2-hydroxy-1-phenylethyl)benzoate (**10f**). General Stille cross-coupling procedure **B** was employed using ethyl 4-bromobenzoate (0.13 mmol), 1,2-borylstannane **8a** (0.15 mmol, 98% ee), $Pd(dba)_2$ (0.0065 mmol), JackiePhos (0.019 mmol), CuCl (0.26 mmol), KF (0.26 mmol) in 1,4-dioxane (1.3 mL) at 110 ºC. Crude product was obtained [75% yield by ^1H NMR]. General procedure for oxidation of C–B bond was employed using NaOH (0.65 mL, 4 M), H_2O_2 (0.65 mL, 30 wt%) in THF (0.65 mL). A transparent oil **10f** (23 mg, 65% yield) was isolated by column chromatography (15:85 to 40:60 ethyl acetate:hexanes). Enantiomeric excess (96.3% ee; 98% es) was determined by HPLC analysis (OJ-RH column, 35:65 CH_3CN:H_2O as eluent). ^1H NMR (300 MHz, $CDCl_3$): δ 8.01 (d, *J* = 8.4 Hz, 2H), 7.36 (m, 4H), 7.27 (m, 3H), 4.40 (q, *J* = 7.2 Hz, 2H), 4.30 (m, 3H), 1.62 (s, 1H, OH), 1.40 (t, *J* = 6.9 Hz, 3H) ppm. ^{13}C NMR (75 MHz, $CDCl_3$): δ 166.7, 146.9, 140.8, 130.1, 129.2, 129.0, 128.5, 127.2, 66.0, 61.1, 53.7, 14.5 ppm. HRMS (ES^+) m/z: $[M+H]^+$ Calcd for $C_{17}H_{19}O_3$ 271.1329; Found: 271.1330.

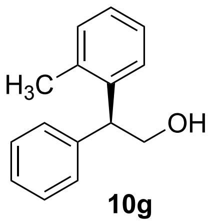

10g

(*S*)-2-Phenyl-2-(*o*-tolyl)ethan-1-ol (10g). General Stille cross-coupling procedure **B** was employed using 2-bromotoluene (0.14 mmol), 1,2-borylstannane **8a** (0.17 mmol, 98% ee), $Pd(dba)_2$ (0.007 mmol), JackiePhos (0.021 mmol), CuCl (0.28 mmol), KF (0.28 mmol) in 1,4-dioxane (1.4 mL) at 110 ºC. Crude product was obtained [70% yield by ^{1}H NMR]. General procedure for oxidation of C–B bond was employed using NaOH (0.65 mL, 4 M), H_2O_2 (0.65 mL, 30 wt%) in THF (0.65 mL). A bright yellow oil **10g** (16.3 mg, 55% yield) was isolated by column chromatography (20:80 ethyl acetate:hexanes). Enantiomeric excess (92.4% ee; 94% es) was determined by HPLC analysis (OJ-RH column, 75:25 CH_3CN:H_2O with 0.1% phosphoric acid as eluent). ^{1}H NMR (300 MHz, $CDCl_3$): δ 7.35 (m, 3H), 7.23 (m, 6H), 4.42 (t, J = 6.9 Hz, 1H), 4.22 (m, 2H), 2.26 (s, 3H), 1.56 (s, 1H, OH) ppm. ^{13}C NMR (75 MHz, $CDCl_3$): δ 141.2, 139.4, 137.4, 131.2, 128.9 (d, J = 7.5 Hz), 126.9 (d, J = 3.8 Hz), 126.7, 126.4, 66.3, 49.9, 20.0 ppm. HRMS: Masses for charged ions were not observed under ESI-MS. However, characteristic fragment ions with a loss of a hydroxyl group were observed. HRMS (ES^+) m/z: $[M-OH]^+$ Calcd for $C_{15}H_{15}$ 195.1168; Found: 195.117. Optical rotation of enantioenriched product $[\alpha]^{25}_D$ (c 0.28, $CHCl_3$) = +59.6 º.

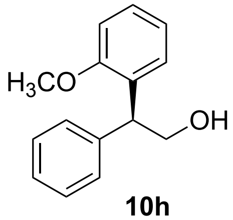

10h

(*S*)-2-(2-Methoxyphenyl)-2-phenylethan-1-ol (10h). General Stille cross-coupling procedure **B** was employed using 2-bromoanisole (0.13 mmol), 1,2-borylstannane **8a** (0.15 mmol, 98% ee), $Pd(dba)_2$ (0.0065 mmol), JackiePhos (0.019 mmol), CuCl (0.26 mmol), KF (0.26 mmol) in 1,4-dioxane (1.3 mL) at 110 ºC. Crude product was obtained [67% yield by ^{1}H NMR]. General procedure for oxidation of C–B bond was employed using NaOH (0.65 mL, 4 M), H_2O_2 (0.65 mL, 30 wt%) in THF (0.65 mL). A dark red oil **10h** (27 mg, 85% yield) was isolated by column chromatography (20:80 ethyl acetate:hexanes). Enantiomeric excess (91.6% ee; 93% es) was determined by HPLC analysis (OJ-RH column, 50:50 CH_3CN:H_2O as eluent). ^{1}H NMR (300 MHz, $CDCl_3$): δ 7.28 (m, 4H), 7.22 (m, 3H), 6.92 (m, 2H), 4.66 (t, J = 6.3 Hz, 1H), 4.14 (d, J = 7.2 Hz, 2H), 3.76 (s, 3H), 1.57 (s, 1H, OH) ppm. ^{13}C NMR (75 MHz, $CDCl_3$): δ 157.6, 141.7, 129.9, 128.8, 128.7, 128.5, 128.0, 126.7, 120.9, 111.1, 65.6, 55.7, 46.8 ppm. HRMS: Masses for charged ions were not observed under ESI-MS. However, characteristic fragment ions with a loss of a hydroxyl

group were observed. HRMS (ES^+) m/z: $[M-OH]^+$ Calcd for $C_{15}H_{15}O$ 211.1117; Found: 211.1116. Optical rotation of enantioenriched product $[\alpha]^{25}_D$ (c 0.2, $CHCl_3$) = –32.0 º.

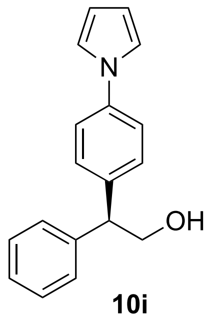

10i

(*S*)-2-(4-(1*H*-Pyrrol-1-yl)phenyl)-2-phenylethan-1-ol (**10i**). General Stille cross-coupling procedure **B** was employed using 1-(4-bromophenyl)-1H-pyrrole (0.1 mmol), 1,2-borylstannane **8a** (0.12 mmol, 98% ee), $Pd(dba)_2$ (0.005 mmol), JackiePhos (0.015 mmol), CuCl (0.2 mmol), KF (0.2 mmol) in 1,4-dioxane (1 mL) at 110 ºC. Crude product was obtained [58% yield by ^1H NMR]. General procedure for oxidation of C–B bond was employed using NaOH (0.65 mL, 4 M), H_2O_2 (0.65 mL, 30 wt%) in THF (0.65 mL). A white solid **10i** (14 mg, 54% yield) was isolated by column chromatography (30:70 ethyl acetate:hexanes). Enantiomeric excess (95.5% ee; 97% es) was determined by HPLC analysis (OJ-RH column, 65:35 CH_3CN:H_2O as eluent). ^1H NMR (300 MHz, $CDCl_3$): δ 7.38 (m, 9H), 7.07 (t, *J* = 2.1 Hz, 2H), 6.35 (t, *J* = 2.4 Hz, 2H), 4.28 (m, 3H), 1.60 (s, 1H, OH) ppm. ^{13}C NMR (75 MHz, $CDCl_3$): δ 141.4, 139.7, 139.2, 129.6, 129.0, 128.5, 127.2, 120.9, 119.5, 110.6, 66.3, 53.2 ppm. HRMS (ES^+) m/z: $[M+H]^+$ Calcd for $C_{18}H_{18}NO$ 264.1383; Found: 264.1384. Optical rotation of enantioenriched product $[\alpha]^{25}_D$ (c 0.4, $CHCl_3$) = +7.8 º.

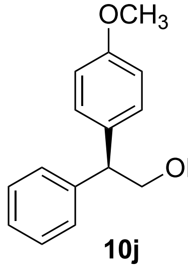

10j

(*S*)-2-(4-Methoxyphenyl)-2-phenylethan-1-ol (**10j**). General Stille cross-coupling procedure **B** was employed using 4-bromoanisole (0.14 mmol), 1,2-borylstannane **8a** (0.17 mmol, 98% ee), $Pd(dba)_2$ (0.007 mmol), JackiePhos (0.021 mmol), CuCl (0.28 mmol), KF (0.28 mmol) in 1,4-dioxane (1.4 mL) at 110 ºC. Crude product was obtained [52% yield by ^1H NMR]. General procedure for oxidation of C–B bond was employed using NaOH (0.65 mL, 4 M), H_2O_2 (0.65 mL, 30 wt%) in THF (0.65 mL). A bright yellow oil **10j** (12.9 mg, 40% yield) was isolated by column chromatography (20:80 ethyl acetate:hexanes). Enantiomeric excess (92.9% ee; 95% es) was determined by HPLC analysis (OJ-RH column, 60:40 CH_3CN:H_2O as eluent). ^1H NMR (300 MHz, $CDCl_3$): δ 7.34 (m, 7H), 6.88 (d, *J* = 8.7 Hz, 2H), 4.16 (m, 3H), 3.78 (s, 3H), 1.50 (s, 1H, OH) ppm. ^{13}C NMR (75 MHz, $CDCl_3$): δ 158.6, 141.9, 133.6, 129.5, 128.9, 128.4, 126.9, 114.3, 66.5, 55.5, 53.0 ppm. HRMS: Masses for charged ions were not observed under ESI-MS. However, characteristic fragment ions with a loss of a hydroxyl group were observed. HRMS (ES^+) m/z: [M-

OH]$^+$ Calcd for $C_{15}H_{15}O$ 211.1117; Found: 211.1115. Optical rotation of enantioenriched product $[\alpha]^{25}_D$ (c 0.12, $CHCl_3$) = +16.7 º.

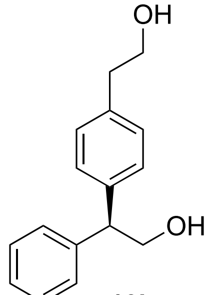

10k

(*S*)-2-(4-(2-Hydroxyethyl)phenyl)-2-phenylethan-1-ol (10k). General Stille cross-coupling procedure **B** was employed using 2-(4-bromophenyl)ethanol (0.1 mmol), 1,2-borylstannane **8a** (0.12 mmol, 98% ee), $Pd(dba)_2$ (0.005 mmol), JackiePhos (0.015 mmol), CuCl (0.2 mmol), KF (0.2 mmol) in 1,4-dioxane (1 mL) at 110 ºC. Crude product was obtained [62% yield by ^{1}H NMR]. General procedure for oxidation of C–B bond was employed using NaOH (0.65 mL, 4 M), H_2O_2 (0.65 mL, 30 wt%) in THF (0.65 mL). A transparent oil **10k** (14.1 mg, 51% yield) was isolated by column chromatography (30:70 ethyl acetate:hexanes). Enantiomeric excess (92.3% ee; 94% es) was determined by HPLC analysis (OJ-RH column, 30:70 CH_3CN:H_2O as eluent). ^{1}H NMR (300 MHz, $CDCl_3$): δ 7.35 (m, 2H), 7.27 (m, 1.3H), 7.23 (m, 5.6H), 4.22 (m, 3H), 3.84 (t, *J* = 6.3 Hz, 2H), 2.84 (t, *J* = 6.3 Hz, 2H), 1.48 (s, 2H, OH) ppm. ^{13}C NMR (75 MHz, $CDCl_3$): δ 141.6, 139.8, 137.2, 129.6, 128.9, 128.7, 128.5, 127.0, 66.4, 63.8, 53.6, 38.9 ppm. HRMS: Masses for charged ions were not observed under ESI-MS. However, characteristic fragment ions with a loss of a hydroxyl group were observed. HRMS (ES$^+$) m/z: [M-OH]$^+$ Calcd for $C_{16}H_{17}O$ 225.1274; Found: 225.1273. Optical rotation of enantioenriched product $[\alpha]^{25}_D$ (c 0.1, $CHCl_3$) = 2.0 º.

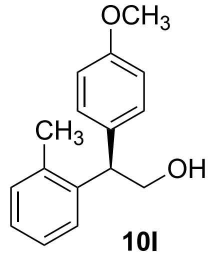

10l

(*S*)-2-(4-Methoxyphenyl)-2-(*o*-tolyl)ethan-1-ol (10l). General Stille cross-coupling procedure **B** was employed using 4-bromoanisole (0.13 mmol), 1,2-borylstannane **8c** (0.15 mmol, 92% ee), $Pd(dba)_2$ (0.0065 mmol), JackiePhos (0.019 mmol), CuCl (0.26 mmol), KF (0.26 mmol) in 1,4-dioxane (1.3 mL) at 110 ºC. Crude product was obtained [60% yield by ^{1}H NMR]. General procedure for oxidation of C–B bond was employed using NaOH (0.65 mL, 4 M), H_2O_2 (0.65 mL, 30 wt%) in THF (0.65 mL). A transparent oil **10l** (15.7 mg, 50% yield) was isolated by column chromatography (15:85 ethyl acetate:hexanes). Enantiomeric excess (91.2% ee; 99% es) was determined by HPLC analysis (OJ-RH column, 45:55 CH_3CN:H_2O as eluent). ^{1}H NMR (300 MHz, $CDCl_3$): δ 7.33 (d, *J* = 6.9 Hz, 1H), 7.25 (m, 5H), 6.85 (d, *J* = 7.8 Hz, 2H), 4.34 (t, *J* = 7.2 Hz, 1H), 4.18 (m, 2H), 3.77 (s, 3H), 2.26 (s, 3H), 1.54 (s, 1H, OH) ppm. ^{13}C NMR (75 MHz, $CDCl_3$): δ 158.5, 139.7, 137.4, 133.2, 131.2, 129.7, 126.9, 126.6, 126.4, 114.3, 66.4, 55.5, 49.0, 19.9 ppm.

HRMS: Masses for charged ions were not observed under ESI-MS. However, characteristic fragment ions with a loss of a hydroxyl group were observed. HRMS (ES^+) m/z: $[M-OH]^+$ Calcd for $C_{16}H_{17}O$ 225.1274; Found: 225.1272. Optical rotation of enantioenriched product $[\alpha]^{25}_D$ (c 1.0, $CHCl_3$) = –18.4 º.

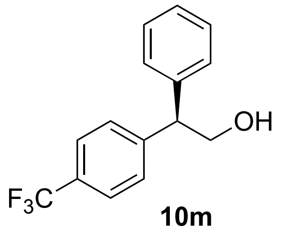
10m

(*S*)-2-Phenyl-2-(4-(trifluoromethyl)phenyl)ethan-1-ol (**10m**). General Stille cross-coupling procedure **B** was employed using bromobenzene (0.21 mmol), 1,2-borylstannane **8b** (0.25 mmol, 90.5% ee), $Pd(dba)_2$ (0.01 mmol), JackiePhos (0.03 mmol), CuCl (0.42 mmol), KF (0.42 mmol) in 1,4-dioxane (2.1 mL) at 110 ºC. Crude product was obtained [70% yield by ^1H NMR]. General procedure for oxidation of C–B bond was employed using NaOH (1 mL, 4 M), H_2O_2 (1 mL, 30 wt%) in THF (1 mL). A transparent oil **10m** (35 mg, 65% yield) was isolated by column chromatography (15:85 ethyl acetate:hexanes). Enantiomeric excess (89.6% ee; 99% es) was determined by HPLC analysis (IA column, 55:45 $CH_3CN:H_2O$ as eluent). ^1H NMR (300 MHz, $CDCl_3$): δ 7.60 (d, *J* = 8.1 Hz, 2H), 7.41 (m, 7H), 4.30 (m, 3H), 1.59 (s, 1H, OH) ppm. ^{13}C NMR (75 MHz, $CDCl_3$): δ 145.96, 140.74, 129.97 (q, *J* = 32 Hz), 129.82 (q, *J* = 270 Hz), 129.17, 128.94, 128.57, 127.43, 125.90 (q, *J* = 3.8 Hz), 66.06, 53.60 ppm. HRMS: Masses for charged ions were not observed under ESI-MS. However, characteristic fragment ions with a loss of a hydroxyl group were observed. HRMS (ES^+) m/z: $[M-OH]^+$ Calcd for $C_{15}H_{12}F_3$ 249.0886; Found: 249.0885. Optical rotation of enantioenriched product $[\alpha]^{25}_D$ (c 0.16, $CHCl_3$) = +6.9 º.

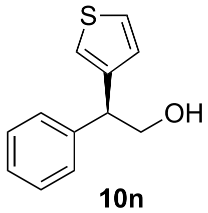
10n

(*S*)-2-Phenyl-2-(thiophen-3-yl)ethan-1-ol (**10n**). General Stille cross-coupling procedure **B** was employed using 3-bromothiophene (0.13 mmol), 1,2-borylstannane **8a** (0.15 mmol, 98% ee), $Pd(dba)_2$ (0.0065 mmol), JackiePhos (0.019 mmol), CuCl (0.26 mmol), KF (0.26 mmol) in 1,4-dioxane (1.3 mL) at 110 ºC. Crude product was obtained [58% yield by ^1H NMR]. General procedure for oxidation of C–B bond was employed using NaOH (0.65 mL, 4 M), H_2O_2 (0.65 mL, 30 wt%) in THF (0.65 mL). A bright yellow oil **10n** (11.7 mg, 45% yield) was isolated by column chromatography (20:80 ethyl acetate:hexanes). Enantiomeric excess (96.1% ee; 98% es) was determined by HPLC analysis (IA column, 65:35 $CH_3CN:H_2O$ as eluent). ^1H NMR (300 MHz, $CDCl_3$): δ 7.36 (m, 6H), 7.10 (d, *J* = 2.7 Hz, 1H), 6.98 (d, *J* = 4.8 Hz, 1H), 4.30 (m, 1H), 4.19 (m,

2H), 1.50 (s, 1H, OH) ppm. ^{13}C NMR (75 MHz, $CDCl_3$): δ 142.2, 141.3, 128.9, 128.5, 127.9, 127.2, 126.1, 121.6, 66.7, 49.7 ppm. HRMS: Masses for charged ions were not observed under ESI-MS. However, characteristic fragment ions with a loss of a hydroxyl group were observed. HRMS (ES^+) m/z: $[M\text{-}OH]^+$ Calcd for $C_{12}H_{11}S$ 187.0576; Found: 187.0579. Optical rotation of enantioenriched product $[\alpha]^{25}_D$ (c 1.6, $CHCl_3$) = +20.6 º.

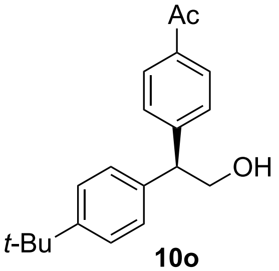

10o

(*S*)-1-(4-(1-(4-(*tert*-Butyl)phenyl)-2-hydroxyethyl)phenyl)ethan-1-one (**10o**). General Stille cross-coupling procedure **B** was employed using 4-bromoacetophenone (0.13 mmol), 1,2-borylstannane **8d** (0.15 mmol, 93% ee), $Pd(dba)_2$ (0.0065 mmol), JackiePhos (0.019 mmol), CuCl (0.26 mmol), KF (0.26 mmol) in 1,4-dioxane (1.3 mL) at 110 ºC. Crude product was obtained [70% ^{1}H NMR]. General procedure for oxidation of the C–B bond was employed using NaOH (0.65 mL, 4 M), H_2O_2 (0.65 mL, 30 wt%) in THF (0.65 mL). A transparent oil **10o** (25.9 mg, 67% yield) was isolated by column chromatography (30:70 ethyl acetate:hexanes). Enantiomeric excess (91.2% ee; 98% es) was determined by HPLC analysis (OJ-RH column, 50:50 CH_3CN:H_2O as eluent). ^{1}H NMR (300 MHz, $CDCl_3$): δ 7.93 (d, J = 8.1 Hz, 2H), 7.40 (dd, J = 8.4 Hz, 3.0 Hz, 4H), 7.18 (d, J = 8.1 Hz, 2H), 4.27 (m, 3H), 2.57 (s, 3H), 1.60 (s, 1H, OH), 1.30 (s, 9H) ppm. ^{13}C NMR (75 MHz, $CDCl_3$): δ 197.9, 150.2, 147.6, 137.6, 135.9, 128.9, 128.8, 128.1, 125.9, 66.1, 53.4, 34.7, 31.5, 26.8 ppm. HRMS (ES^+) m/z: $[M+H]^+$ Calcd for $C_{20}H_{25}O_2$ 297.1849; Found: 297.1850. Optical rotation of enantioenriched product $[\alpha]^{25}_D$ (c 0.12, $CHCl_3$) = +17.5 º.

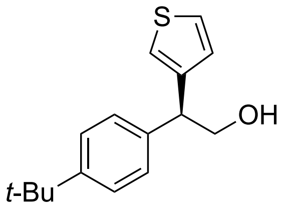

10p

(*S*)-2-(4-(*tert*-Butyl)phenyl)-2-(thiophen-3-yl)ethan-1-ol (**10p**). General Stille cross-coupling procedure **B** was employed using 3-bromothiophene (0.07 mmol), 1,2-borylstannane **8d** (0.084 mmol, 95% ee), $Pd(dba)_2$ (0.0035 mmol), JackiePhos (0.010 mmol), CuCl (0.14 mmol), KF (0.14 mmol) in 1,4-dioxane (0.7 mL) at 110 ºC. Crude product was obtained [46% yield by ^{1}H NMR]. General procedure for oxidation of the C–B bond was employed using NaOH (0.38 mL, 4 M), H_2O_2 (0.38 mL, 30 wt%) in THF (0.38 mL). A transparent oil **10p** (7 mg, 35% yield) was isolated by column chromatography (30:70 ethyl acetate:hexanes). Enantiomeric excess (93.0% ee; 98% es) was determined by HPLC analysis (OJ-RH column, 80:20 CH_3CN:H_2O as

eluent). ^{1}H NMR (300 MHz, $CDCl_3$): δ 7.36 (m, 2H), 7.30 (m, 1H), 7.19 (d, J = 7.8 Hz, 2H), 7.10 (m, 1H), 6.99 (dd, J = 3.9 Hz, 1.2 Hz, 1H), 4.27 (t, J = 6.9 Hz, 1H), 4.16 (m, 2H), 1.51 (s, 1H, OH), 1.31 (s, 9H) ppm. ^{13}C NMR (75 MHz, $CDCl_3$): δ 150.0, 142.5, 138.1, 128.1, 128.0, 126.1, 125.9, 121.5, 66.8, 49.3, 34.6, 31.5 ppm. HRMS (ES^+) m/z: $[M+H]^+$ Calcd for $C_{16}H_{21}OS$ 261.1308; Found: 261.1314. Optical rotation of enantioenriched product $[\alpha]^{25}_{D}$ (c 0.1, $CHCl_3$) = +25.5 º.

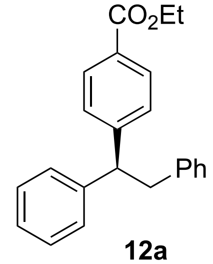

12a

Ethyl (*S*)-4-(1,2-diphenylethyl)benzoate (**12a**). General Stille cross-coupling procedure **C** was employed using ethyl 4-bromobenzoate (0.23 mmol), organostannane **11a** (0.27 mmol, 97% ee), $Pd(dba)_2$ (0.011 mmol), JackiePhos (0.034 mmol), CuCl (0.46 mmol), KF (0.46 mmol) in 1,4-dioxane (2.3 mL) at 110 ºC. A transparent oil **12a** (63.7 mg, 84% yield) was isolated by column chromatography (5:95 ethyl acetate:hexanes). Enantiomeric excess (93.1% ee; 96% es) was determined by HPLC analysis (OJ-RH column, 55:45 CH_3CN:H_2O as eluent). ^{1}H NMR (300 MHz, $CDCl_3$): δ 7.95 (d, J = 8.4 Hz, 2H), 7.31 (m, 10H), 7.03 (m, 2H), 4.39 (m, 3H), 3.41 (m, 2H), 1.40 (t, J = 7.2 Hz, 3H) ppm. ^{13}C NMR (75 MHz, $CDCl_3$): δ 166.7, 149.7, 143.9, 139.9, 129.8, 129.2, 128.7, 128.3, 128.3, 128.2, 126.7, 126.2, 60.9, 53.3, 41.9, 14.5 ppm. HRMS (ES^+) m/z: $[M+H]^+$ Calcd for $C_{23}H_{23}O_2$ 331.1693; Found: 331.1692. Optical rotation of enantioenriched product $[\alpha]^{25}_{D}$ (c 0.2, $CHCl_3$) = –53.0 º.

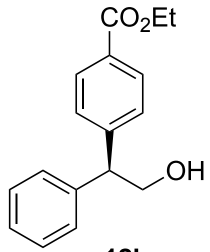

12b

Ethyl (*S*)-4-(2-hydroxy-1-phenylethyl)benzoate (**12b**) *[same compound as **10f**, but prepared using **11c** as nucleophile instead of **8a**]*. General Stille cross-coupling procedure **C** was employed using ethyl 4-bromobenzoate (0.05 mmol), organostannane **11c** (0.06 mmol, 94% ee), $Pd(dba)_2$ (0.0025 mmol), JackiePhos (0.0075 mmol), CuCl (0.1 mmol), KF (0.1 mmol) in 1,4-dioxane (0.5 mL) at 110 ºC. A transparent oil **12b** (7.4 mg, 58% yield) was isolated by column chromatography (30:70 ethyl acetate:hexanes). Enantiomeric excess (89.6% ee; 95% es) was determined by HPLC analysis. ^{1}H NMR (300 MHz, $CDCl_3$): δ 8.01 (d, J = 8.4 Hz, 2H), 7.36 (m, 4H), 7.27 (m, 3H), 4.40 (q, J = 7.2 Hz, 2H), 4.30 (m, 3H), 1.62 (s, 1H, OH), 1.40 (t, J = 6.9 Hz, 3H) ppm. ^{13}C NMR (75 MHz, $CDCl_3$): δ 166.7, 146.9, 140.8, 130.1, 129.2, 129.0, 128.5, 127.2, 66.0, 61.1, 53.7, 14.5 ppm. HRMS (ES^+) m/z: $[M+H]^+$ Calcd for $C_{17}H_{19}O_3$ 271.1329; Found: 271.1330.

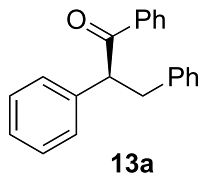

13a

(*S*)-1,2,3-triphenylpropan-1-one (**13a**). General cross-coupling procedure **C** was employed using S-phenyl benzothioate (0.2 mmol), organostannane **11a** (0.24 mmol, 97% ee), Pd(dba)$_2$ (0.01 mmol), JackiePhos (0.03 mmol), CuCl (0.4 mmol), KF (0.4 mmol) in 1,4-dioxane (2 mL) at 110 ºC. A white solid **13a** (41 mg, 75% yield) was isolated by column chromatography (1:99 ethyl acetate:hexanes). Enantiomeric excess (95.3% ee; 98% es) was determined by HPLC analysis (OJ-RH column, 50:50 CH_3CN:H_2O as eluent). ^{1}H NMR (300 MHz, $CDCl_3$): δ 7.91 (d, *J* = 6.9 Hz, 2H), 7.48 (m, 1H), 7.37 (m, 2H), 7.24 (m, 10H), 4.83 (t, *J* = 6.9 Hz, 1H), 3.60 (m, 1H), 3.10 (m, 1H) ppm. ^{13}C NMR (75 MHz, $CDCl_3$): δ 199.4, 140.0, 139.3, 136.9, 133.1, 129.3, 129.1, 128.9, 128.7, 128.5, 128.5, 127.4, 126.3, 56.2, 40.3 ppm. HRMS (ES$^+$) m/z: $[M+H]^+$ Calcd for $C_{21}H_{19}O$ 287.1430; Found: 287.1433. Optical rotation of enantioenriched product $[\alpha]^{25}_D$ (c 2.0, $CHCl_3$) = +9.6 º.

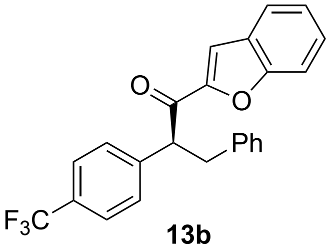

(S)-1-(benzofuran-2-yl)-3-phenyl-2-(4-(trifluoromethyl)phenyl)-propan-1-one (**13b**). General cross-coupling procedure **C** was employed using S-phenyl benzofuran-2-carbothioate (0.1 mmol), organostannane **11b** (0.12 mmol, 90.5% ee), Pd(dba)$_2$ (0.005 mmol), JackiePhos (0.015 mmol), CuCl (0.2 mmol), KF (0.2 mmol) in 1,4-dioxane (1 mL) at 110 ºC. A white solid **13b** (16.6 mg, 40% yield) was isolated by column chromatography (3:97 ethyl acetate:hexanes). Enantiomeric excess (89.0% ee; 98% es) was determined by HPLC analysis (OD-RH column, 75:25 CH_3CN:H_2O as eluent). ^{1}H NMR (300 MHz, $CDCl_3$): δ 7.65 (d, *J* = 7.8 Hz, 1H), 7.56 (m, 7H), 7.30 (m, 6H), 4.88 (t, *J* = 6.6 Hz, 1H), 3.66 (m, 1H), 3.15 (m, 1H) ppm. ^{13}C NMR (75 MHz, $CDCl_3$): δ 189.65, 155.86, 152.11, 142.48, 138.94, 130.50 (q, *J* = 30 Hz), 129.62 (q, *J* = 269 Hz), 129.25, 129.03, 128.73, 128.67, 127.11, 126.74, 126.04 (q, *J* = 3.8 Hz), 124.23, 123.56, 114.15, 112.67, 56.15, 39.28 ppm. HRMS (ES$^+$) m/z: $[M+H]^+$ Calcd for $C_{24}H_{18}F_3O$ 395.1253; Found: 395.1254. Optical rotation of enantioenriched product $[\alpha]^{25}_D$ (c 0.4, $CHCl_3$) = +8.8 º.

5. Single Crystal X-Ray Structure of Compound 8a

Experimental Description

X-ray diffraction data were collected on a Bruker D8 VENTURE diffractometer. The structure was solved using a dual-space method and standard difference map techniques, and was refined by full-matrix least-squares procedures on F^2 with SHELXTL (Version 2018/3). All hydrogen atoms were placed in calculated positions and refined with a riding model [U_{iso}(H) = 1.2–1.5U_{eq}(C)].

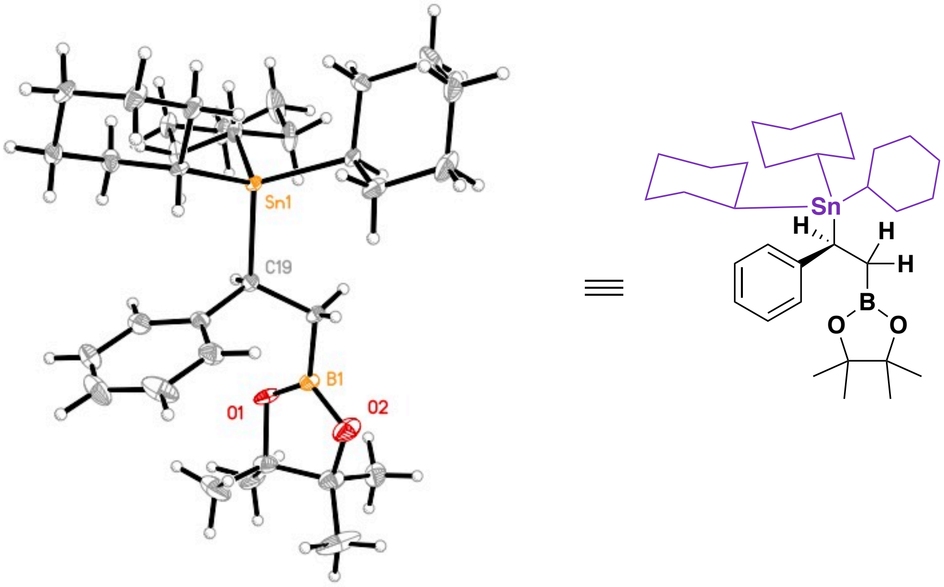

Figure S8. Single crystal X-ray structure of **8a**.

6. References

1. S. Aalla, G. Gilla, Y. Bojja, R. R. Anumula, P. R. Vummenthala, P. R. Padi, *Org. Process Res. Dev.* 2012, **16**, 682.
2. T. Jia, P. Cao, D. Wang, Y. Lou, J. Liao, *Chem. Eur. J.* 2015, **21**, 4918.
3. J. L. García Ruano, J. Alemán, M. T. Aranda, M. J. Arévalo, A. Padwa, *Org. Lett.* 2005, **7**, 19.
4. V. Saini, L. Liao, Q. Wang, R. Jana, M. S. Sigman, *Org. Lett.* 2013, **15**, 5008.
5. A. López-Pérez, J. Adrio, J. C. Carretero, *Org. Lett.* 2009, **11**, 5514.

6. M. Amatore, C. Gosmini, *Chem. Comm.* 2008, 5019.
7. X. Cheng, H. Lu, Z. Lu, *Nat. Commun.* 2019, **10**, 3549.
8. C. Cazorla, E. Métay, M. Lemaire, *Tetrahedron* 2011, **67**, 8615.
9. H.-B. Sun, B. Li, R. Hua, Y. Yin, *Eur. J. Org. Chem.* 2006, **2006**, 4231.
10. C. Dong, X. Wang, Z. Pei, R. Shen, *Org. Lett.* 2019, **21**, 4148.
11. A. M. Vasquez, J. A., Gurak, C. L. Joe, E. C. Cherney, K. M. Engle, *J. Am. Chem. Soc.* 2020, **142**, 10477.

7. Chiral-Phase HPLC Data

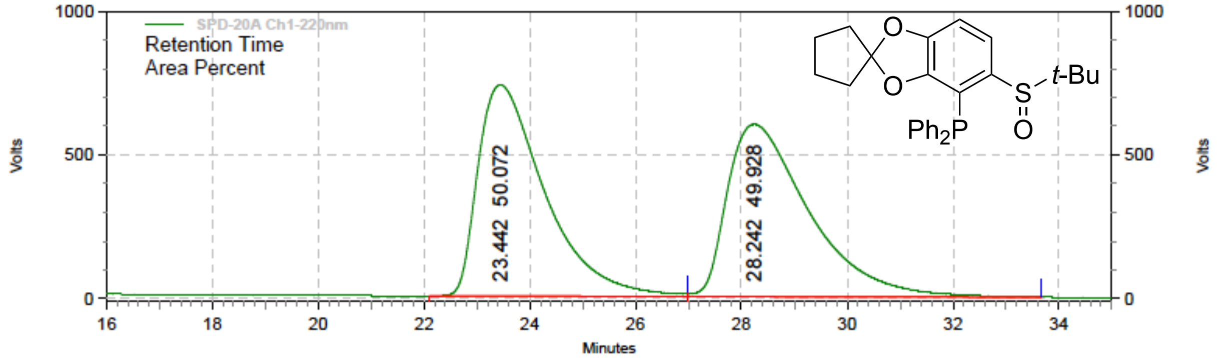

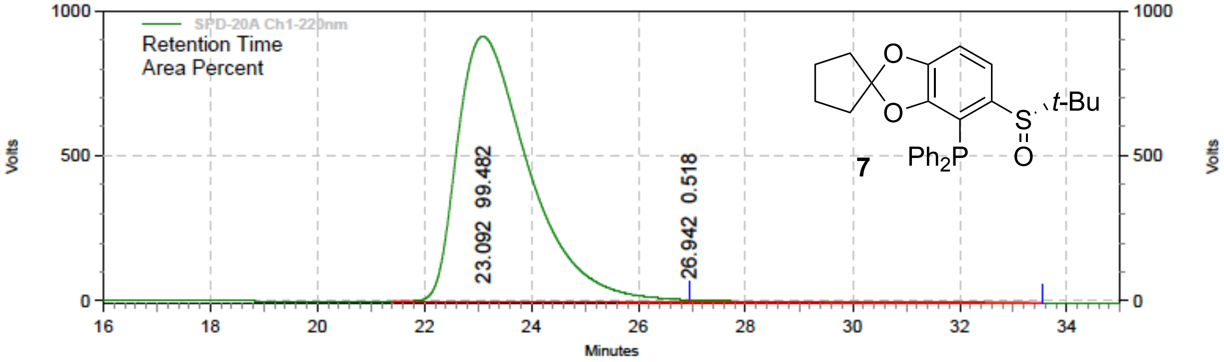

Conditions and results:

Column	IA
Mobile phase	65:35 = CH_3CN:H_2O
Flow	0.8 mL/min
Detector	220 nm
Temp	25 °C

Figure S9. HPLC traces of racemic and enantioenriched **7**.

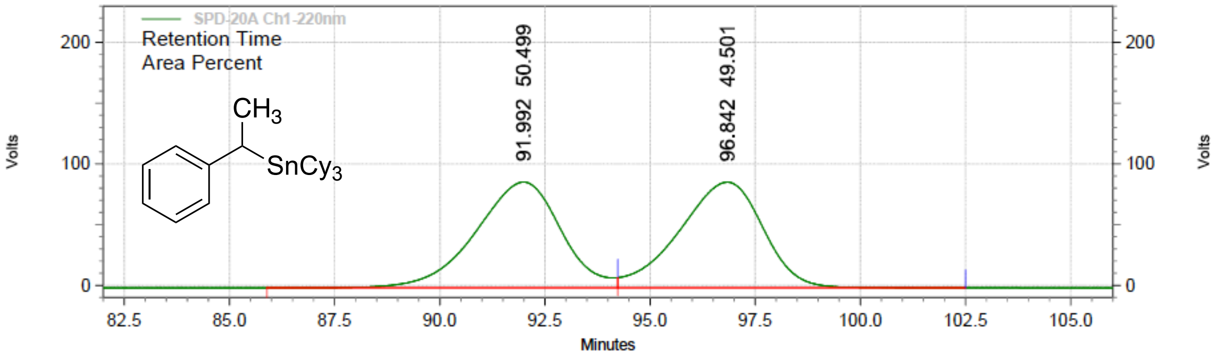

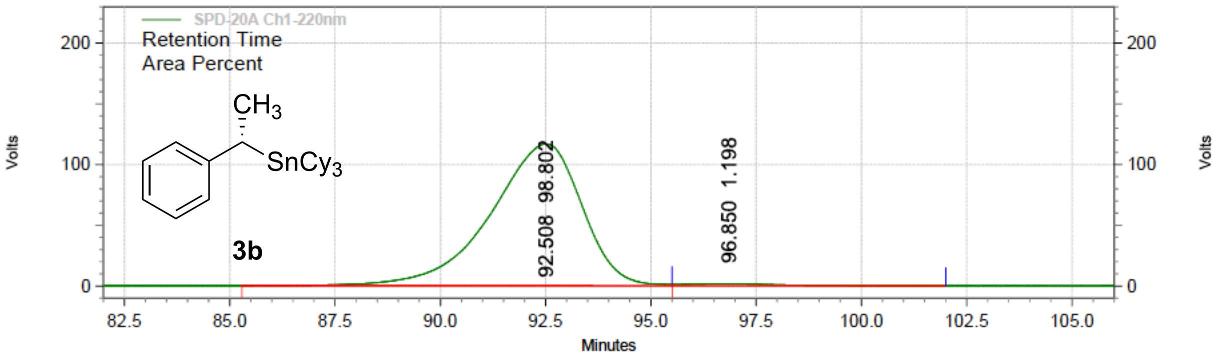

Conditions and results:

Column	OD-RH
Mobile phase	75:25 = CH_3CN:H_2O
Flow	0.4 mL/min
Detector	220 nm
Temp	25 ºC

Figure S10. HPLC traces of racemic and enantioenriched **3b**.

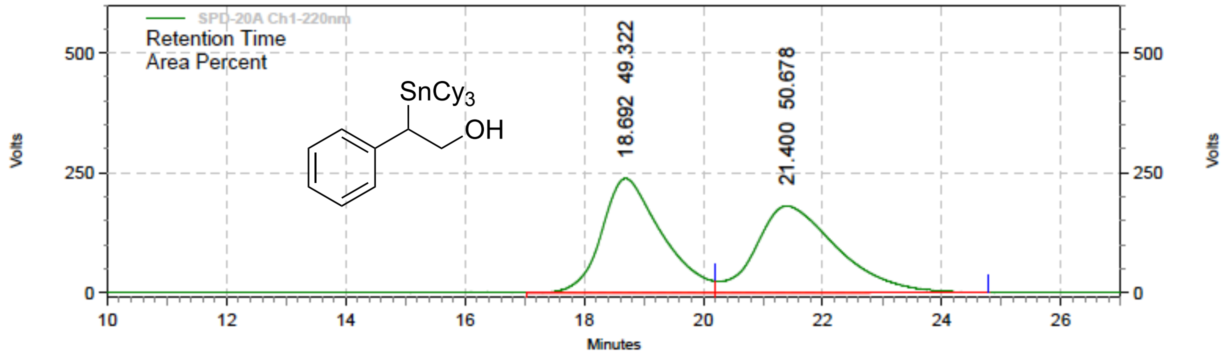

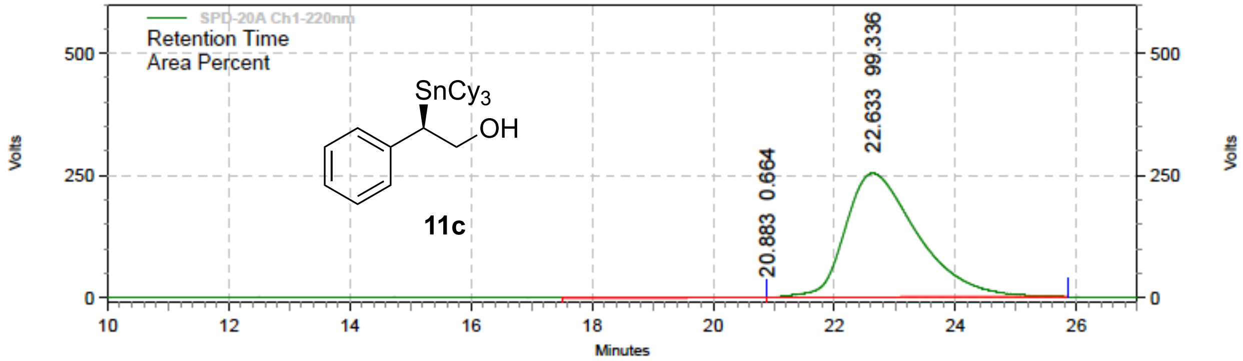

Conditions and results:

Column	IA
Mobile phase	75:25 = CH_3CN:H_2O
Flow	1 mL/min
Detector	220 nm
Temp	25 ºC

Figure S11. HPLC traces of racemic and enantioenriched **11c**.

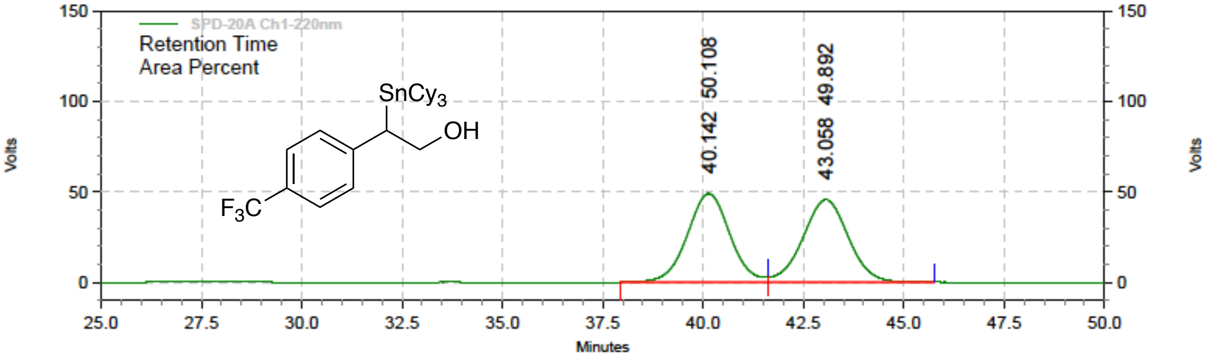

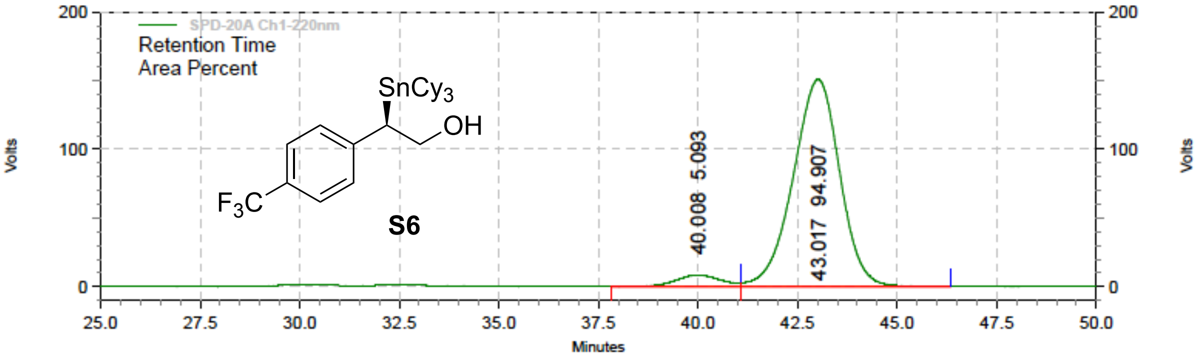

Conditions and results:

Column	OD-RH
Mobile phase	65:35 = CH_3CN:H_2O
Flow	0.8 mL/min
Detector	220 nm
Temp	25 ºC

Figure S12. HPLC traces of racemic and enantioenriched **S6**.

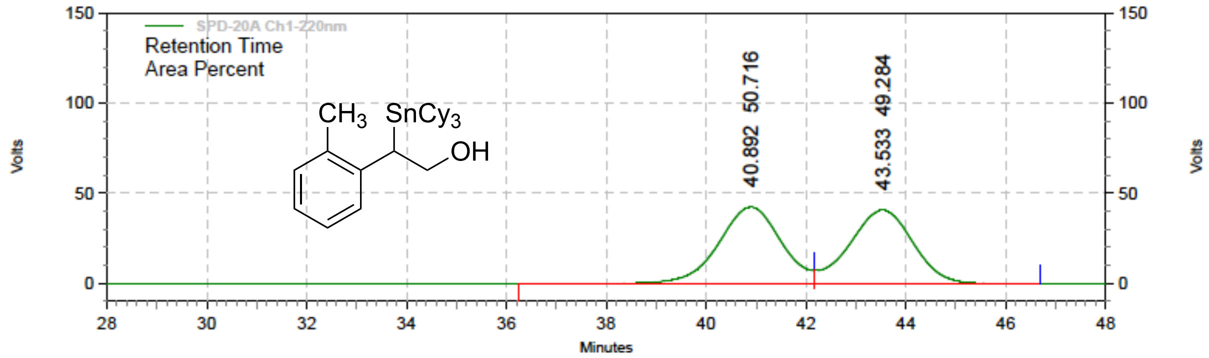

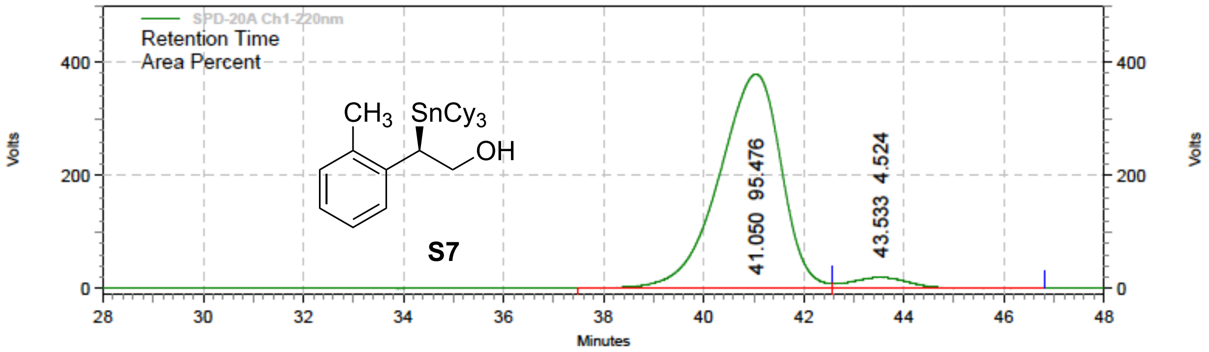

Conditions and results:

Column	OD-RH
Mobile phase	65:35 = CH_3CN:H_2O
Flow	1 mL/min
Detector	220 nm
Temp	25 ºC

Figure S13. HPLC traces of racemic and enantioenriched **S7**.

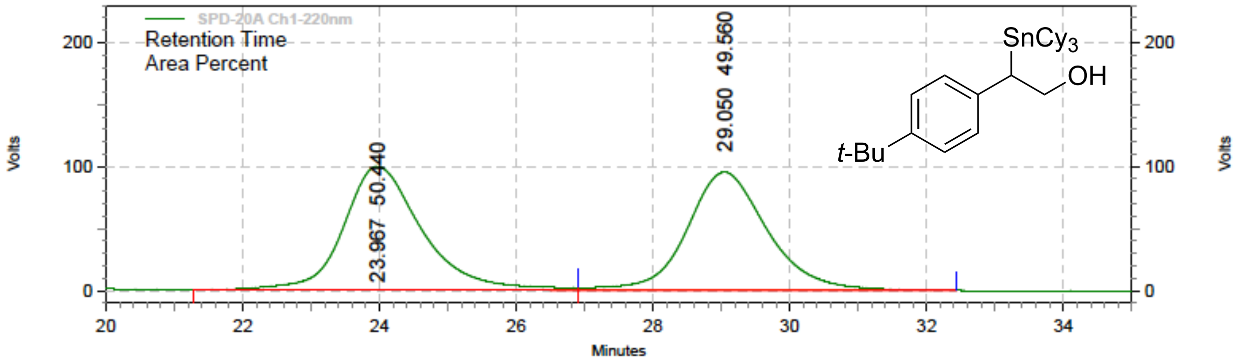

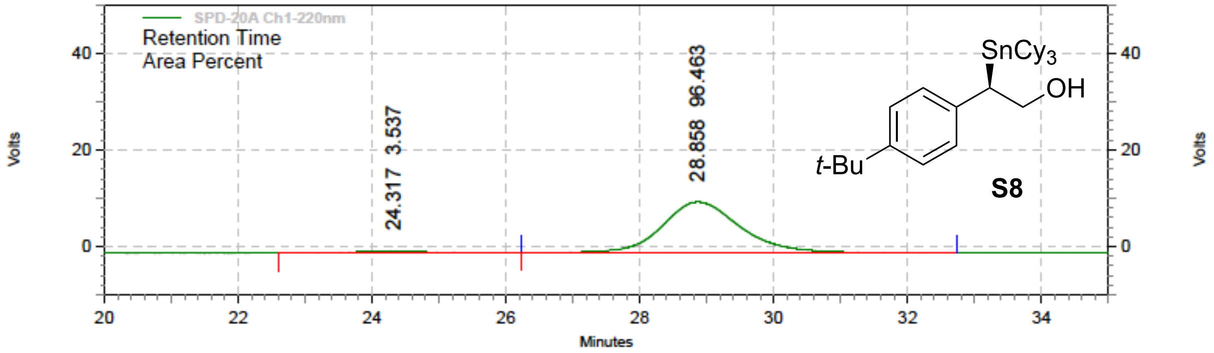

Conditions and results:

Column	OJ-RH
Mobile phase	65:35 = $CH_3CN:H_2O$
Flow	1 mL/min
Detector	220 nm
Temp	25 ºC

Figure S14. HPLC traces of racemic and enantioenriched **S8**.

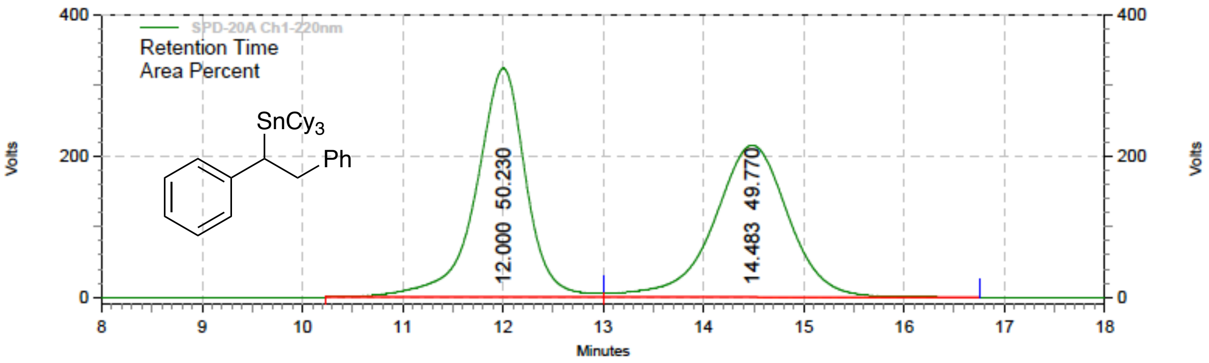

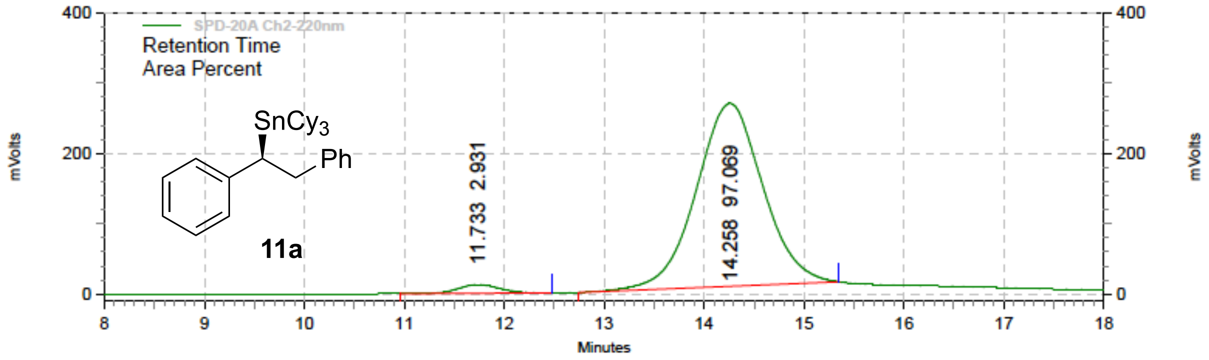

Conditions and results:

Column	OJ-RH
Mobile phase	75:25 = CH_3CN:H_2O
Flow	1.4 mL/min
Detector	220 nm
Temp	25 ºC

Figure S15. HPLC traces of racemic and enantioenriched **11a**.

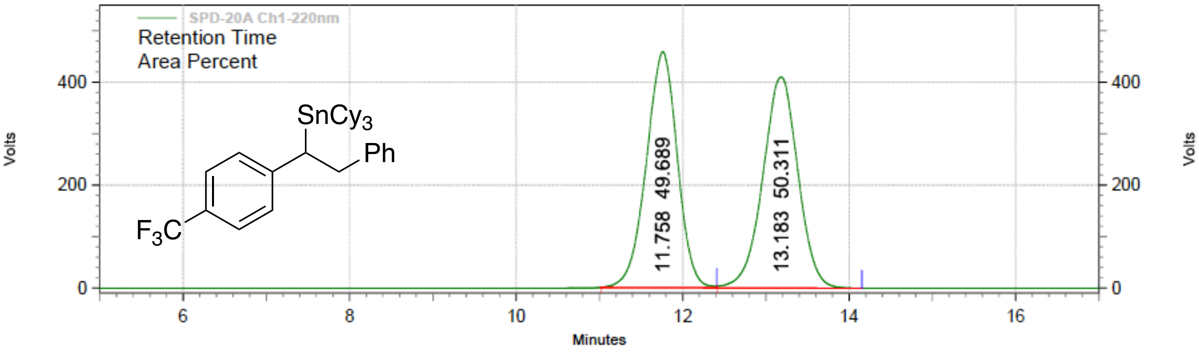

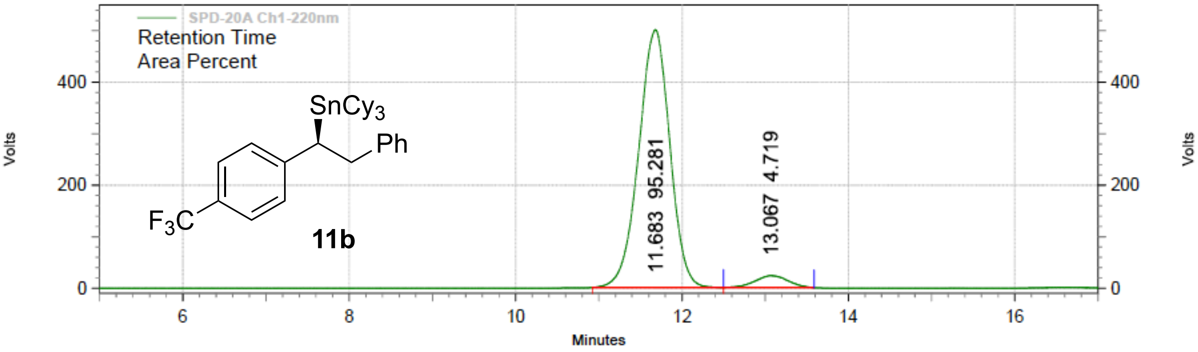

Conditions and results:

Column	OD-RH
Mobile phase	85:15 = CH_3CN:H_2O
Flow	1 mL/min
Detector	220 nm
Temp	25 ºC

Figure S16. HPLC traces of racemic and enantioenriched **11b**.

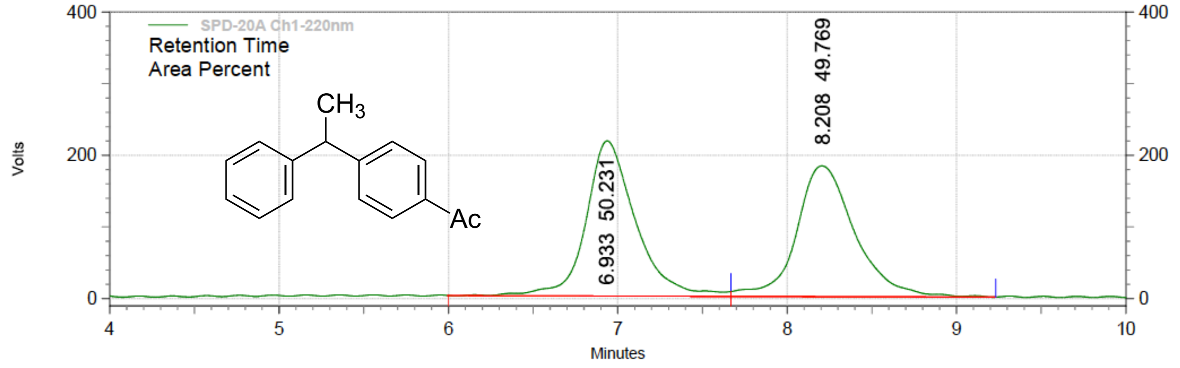

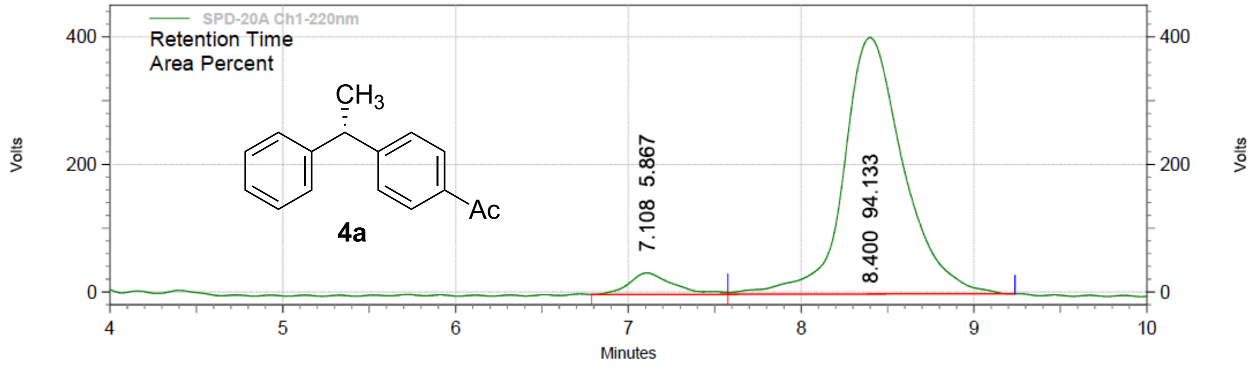

Conditions and results:

Column	OJ-RH
Mobile phase	90:10 = (5% CH_3CN in CH_3OH) : H_2O
Flow	1 mL/min
Detector	220 nm
Temp	25 ºC

Figure S17. HPLC traces of racemic and enantioenriched **4a**.

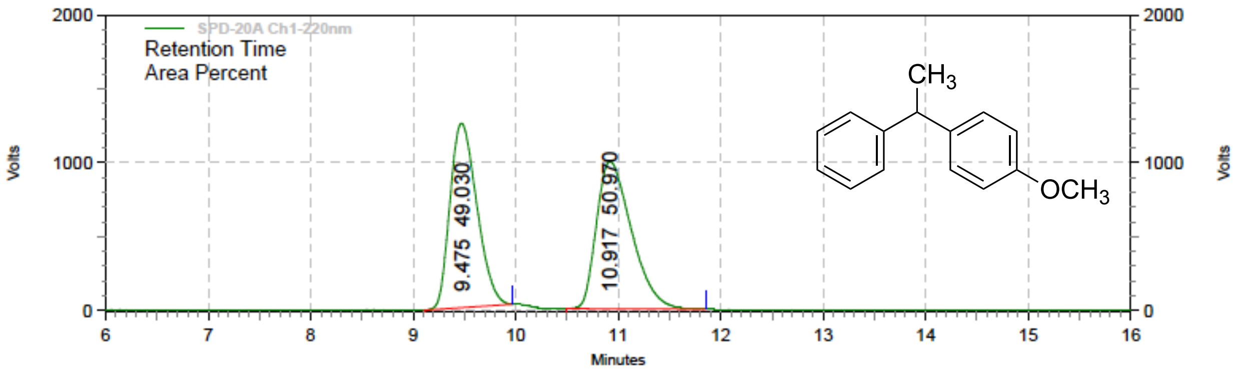

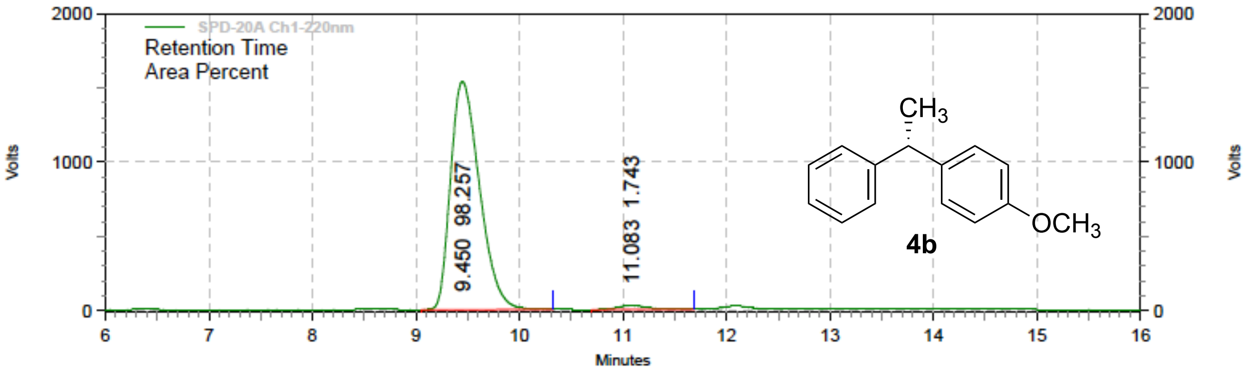

Conditions and results:

Column	IA
Mobile phase	85:15 = (5% CH_3CN in CH_3OH) : H_2O
Flow	1 mL/min
Detector	220 nm
Temp	25 ºC

Figure S18. HPLC traces of racemic and enantioenriched **4b**.

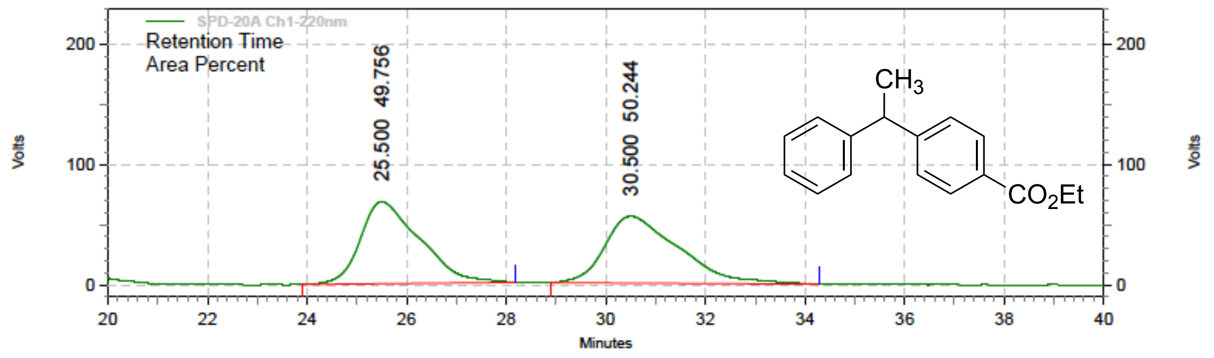

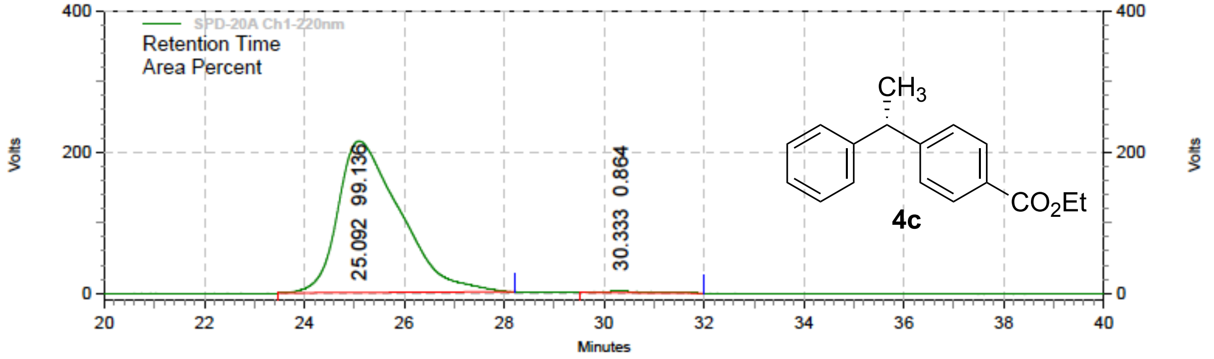

Conditions and results:

Column	OJ-RH
Mobile phase	80:20 = (5% CH_3CN in CH_3OH) : H_2O
Flow	0.8 mL/min
Detector	220 nm
Temp	25 ºC

Figure S19. HPLC traces of racemic and enantioenriched **4c**.

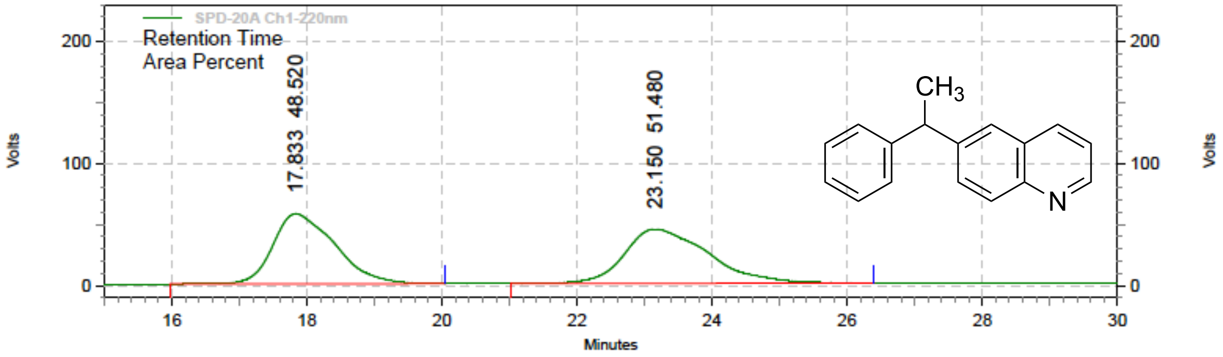

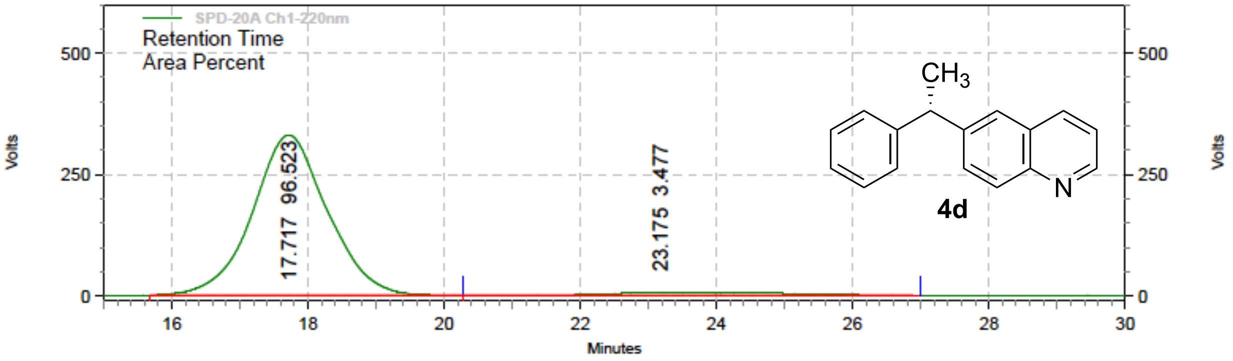

Conditions and results:

Column	OJ-RH
Mobile phase	80:20 = (5% CH_3CN in CH_3OH) : H_2O
Flow	0.6 mL/min
Detector	220 nm
Temp	25 ºC

Figure S20. HPLC traces of racemic and enantioenriched **4d**.

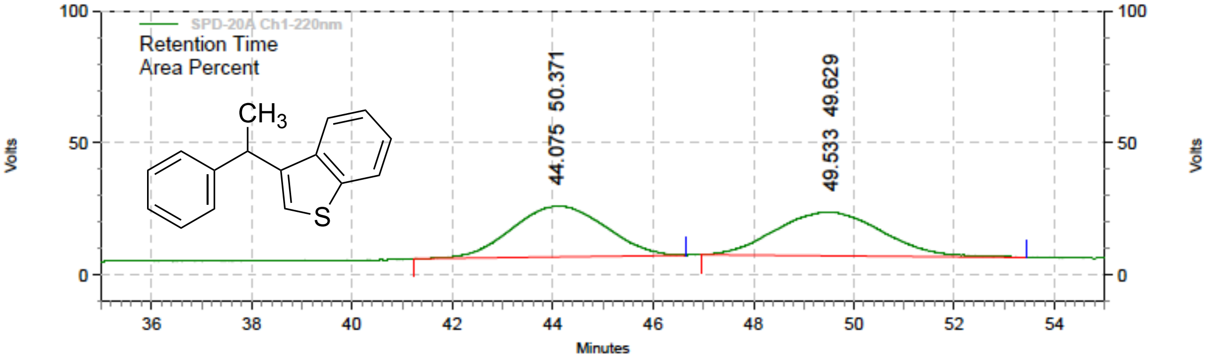

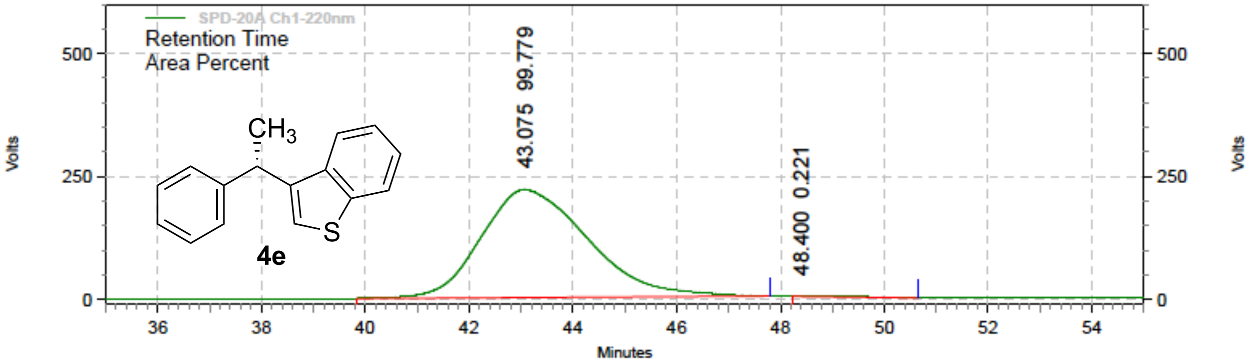

Conditions and results:

Column	OJ-RH
Mobile phase	85:15 = (5% CH_3CN in CH_3OH) : H_2O
Flow	0.8 mL/min
Detector	220 nm
Temp	25 ºC

Figure S21. HPLC traces of racemic and enantioenriched **4e**.

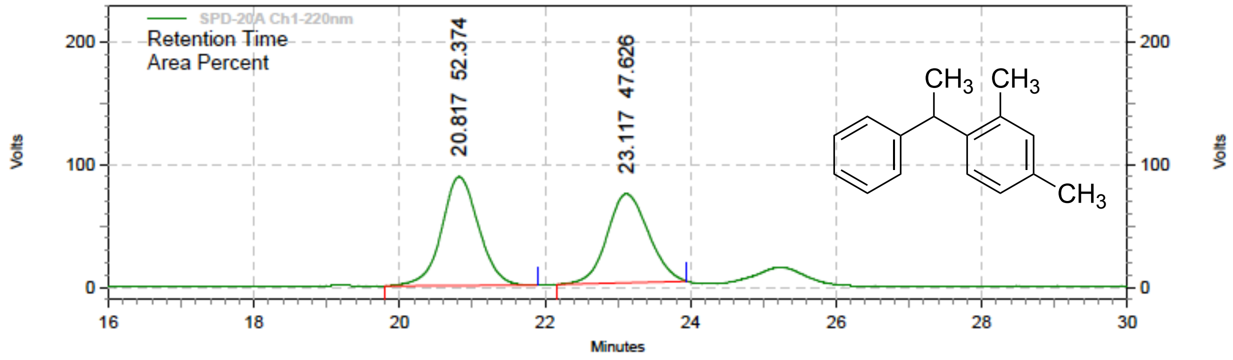

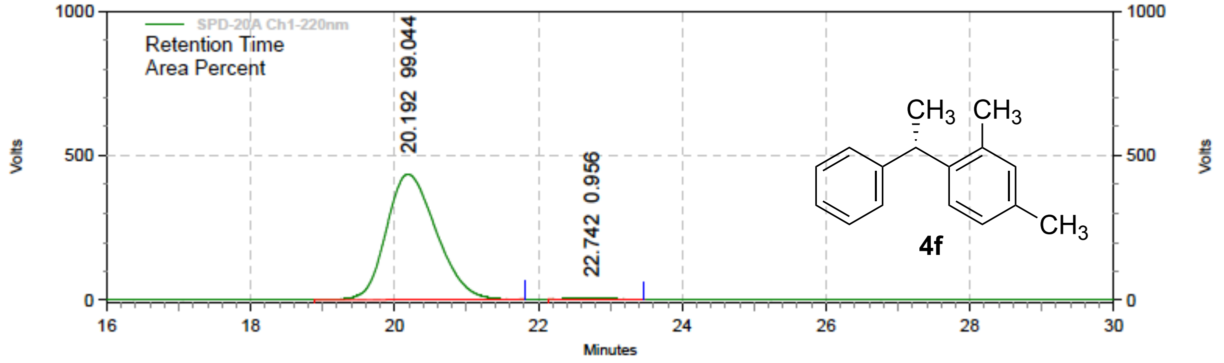

Conditions and results:

Column	IA
Mobile phase	75:25 = (5% CH_3CN in CH_3OH) : H_2O
Flow	1 mL/min
Detector	220 nm
Temp	25 ºC

Figure S22. HPLC traces of racemic and enantioenriched **4f**.

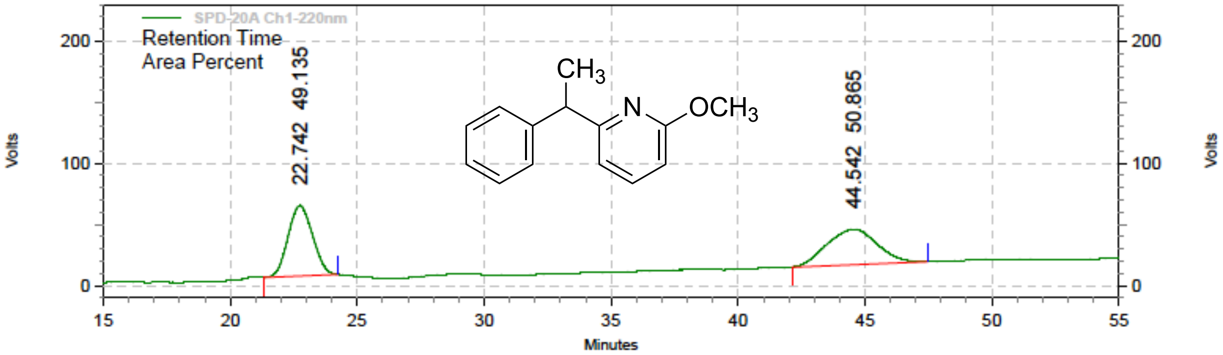

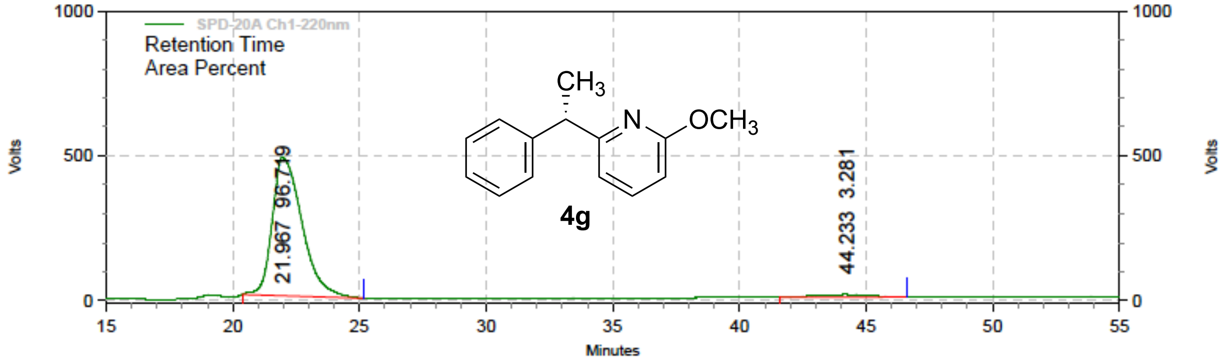

Conditions and results:

Column	OJ-RH
Mobile phase	85:15 = (5% CH_3CN in CH_3OH) : H_2O
Flow	0.8 mL/min
Detector	220 nm
Temp	25 ºC

Figure S23. HPLC traces of racemic and enantioenriched **4g**.

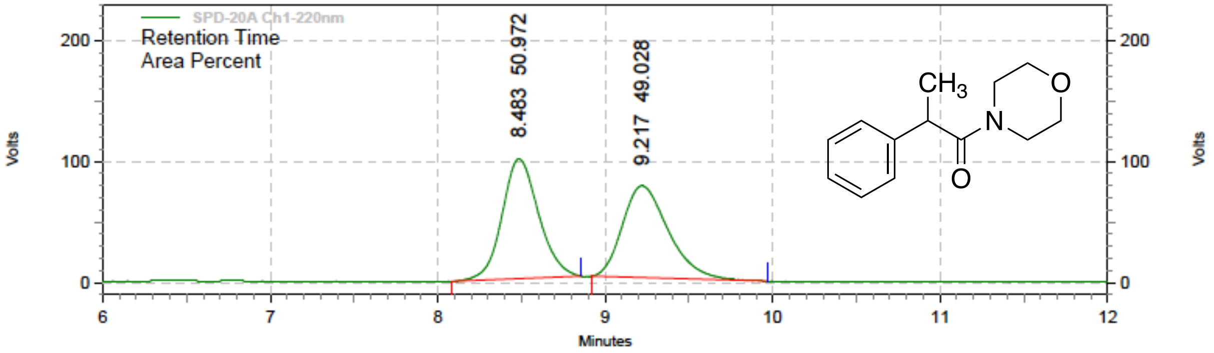

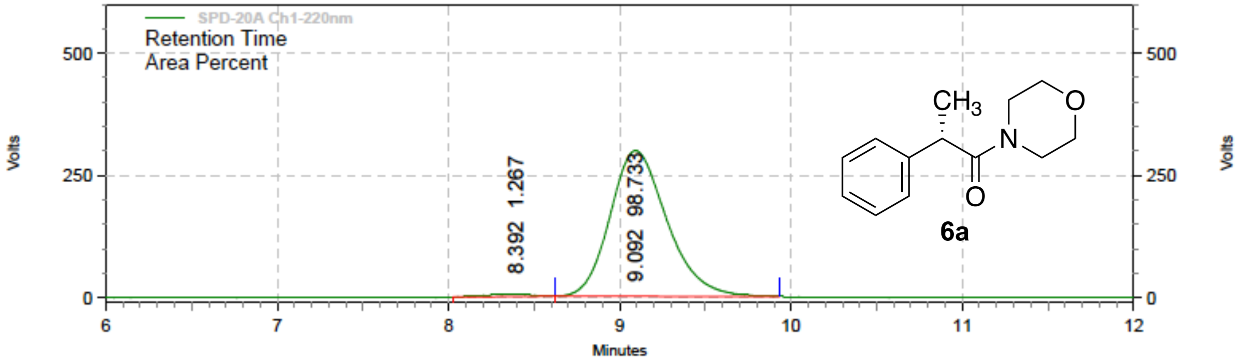

Conditions and results:

Column	IA
Mobile phase	70:30 = (5% CH_3CN in CH_3OH) : H_2O
Flow	0.8 mL/min
Detector	220 nm
Temp	25 ºC

Figure S24. HPLC traces of racemic and enantioenriched **6a**.

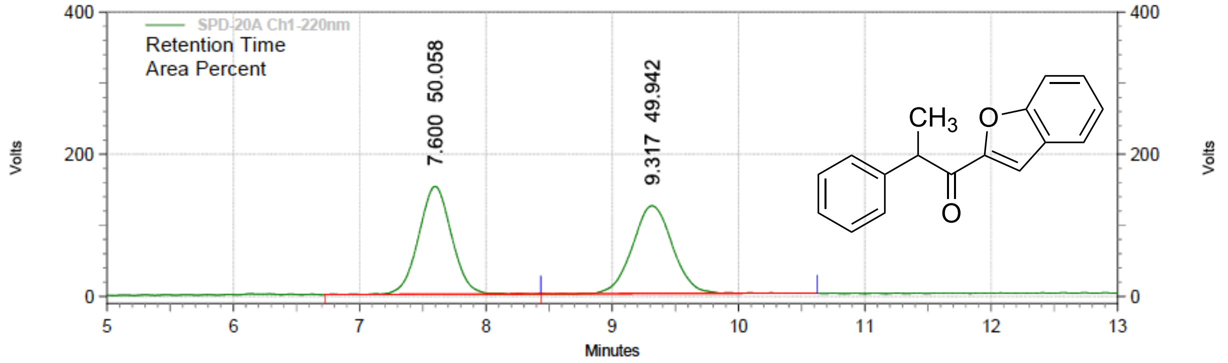

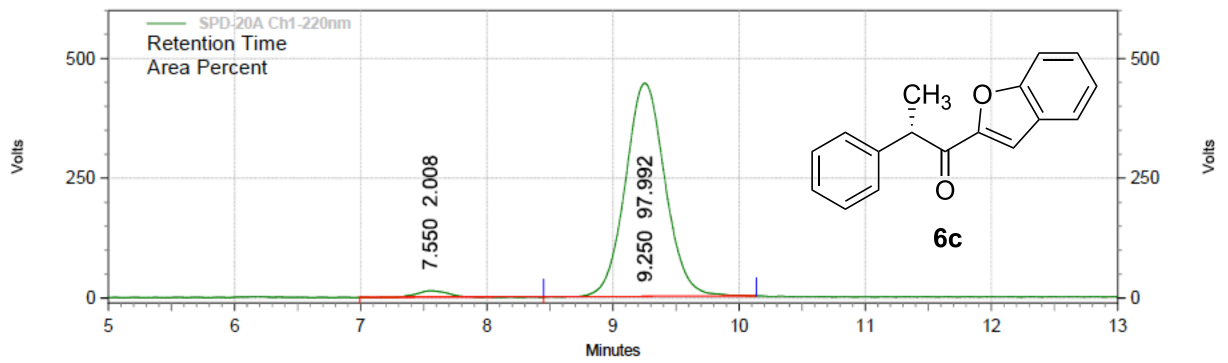

Conditions and results:

Column	OD-RH
Mobile phase	85:15 = (5% CH_3CN in CH_3OH) : H_2O
Flow	0.8 mL/min
Detector	220 nm
Temp	25 ºC

Figure S25. HPLC traces of racemic and enantioenriched **6c**.

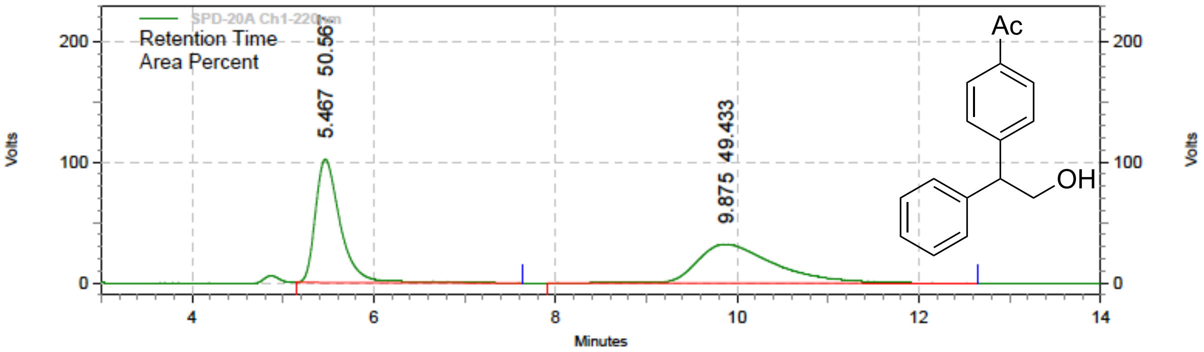

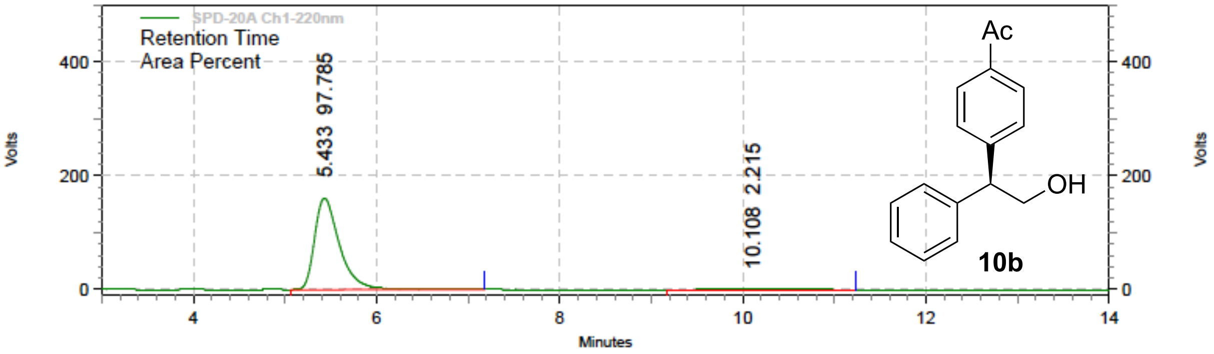

Conditions and results:

Column	IA
Mobile phase	55:45 = $CH_3CN:H_2O$
Flow	1.4 mL/min
Detector	220 nm
Temp	25 ºC

Figure S26. HPLC traces of racemic and enantioenriched **10b**.

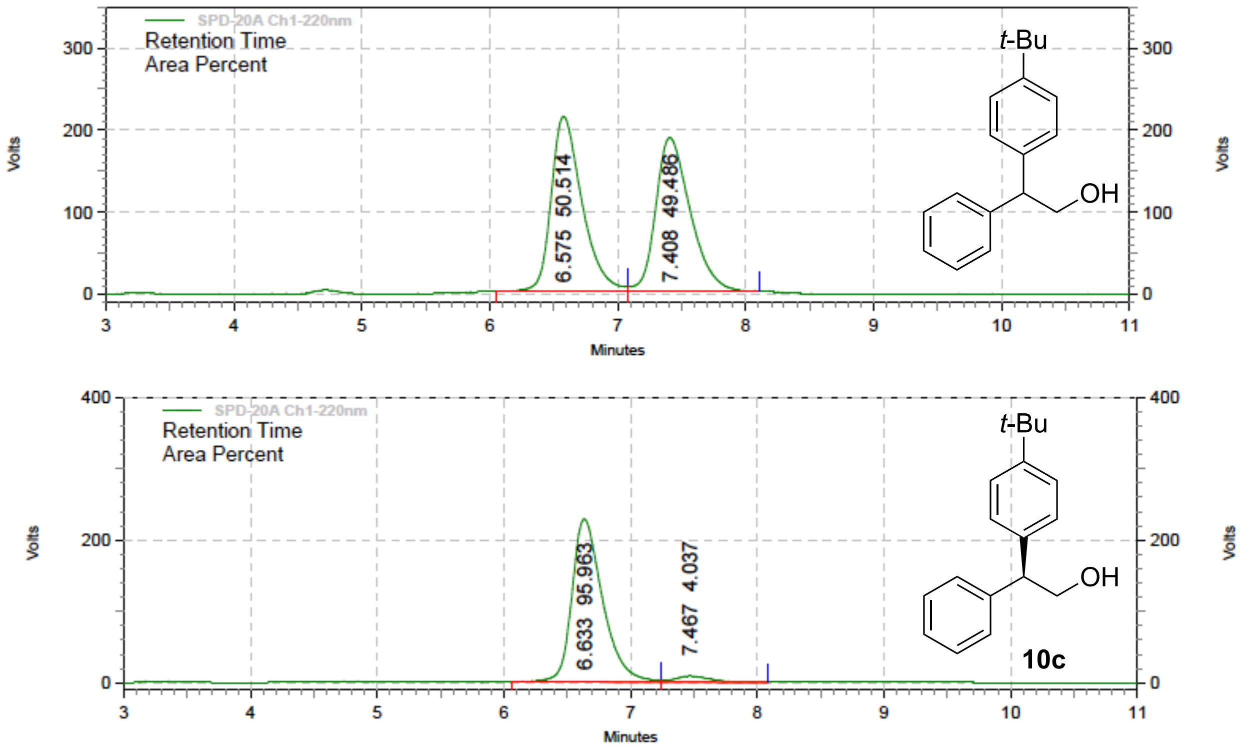

Conditions and results:

Column	OJ-RH
Mobile phase	60:40 = CH_3CN:H_2O
Flow	0.8 mL/min
Detector	220 nm
Temp	25 ºC

Figure S27. HPLC traces of racemic and enantioenriched **10c**.

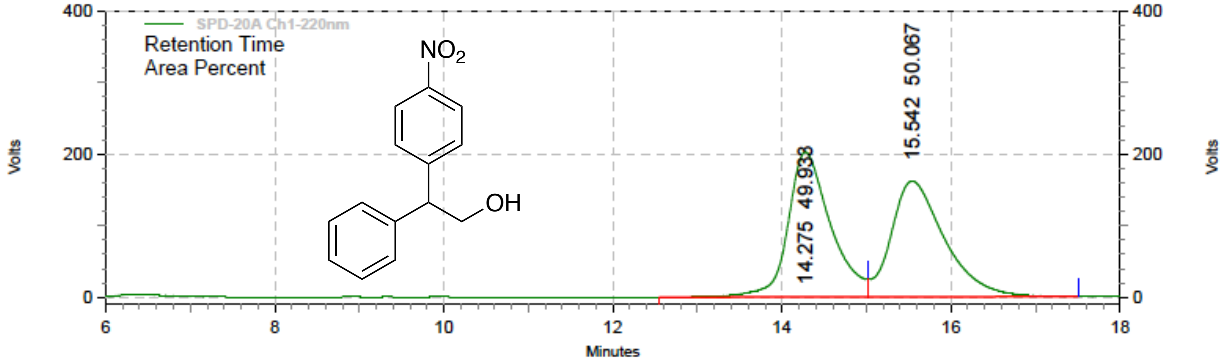

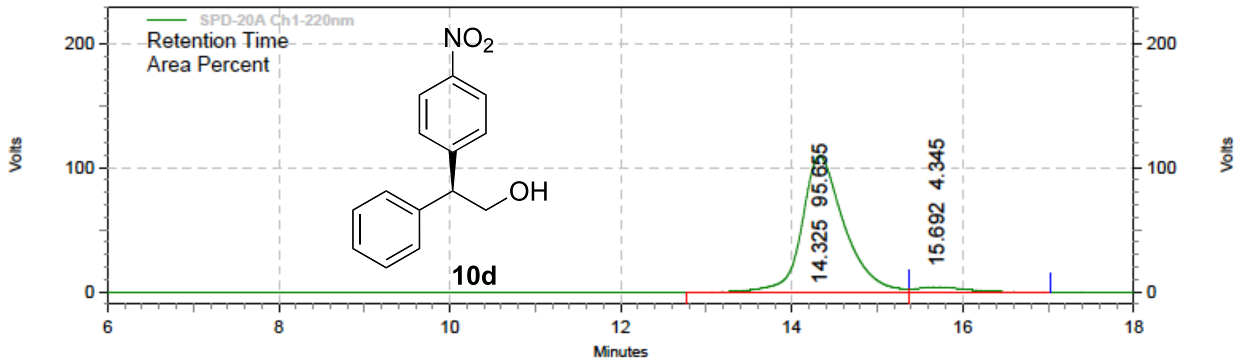

Conditions and results:

Column	OJ-RH
Mobile phase	40:60 = CH_3CN:H_2O (0.1% phosphoric acid buffer pH=3)
Flow	0.8 mL/min
Detector	220 nm
Temp	25 ºC

Figure S28. HPLC traces of racemic and enantioenriched **10d**.

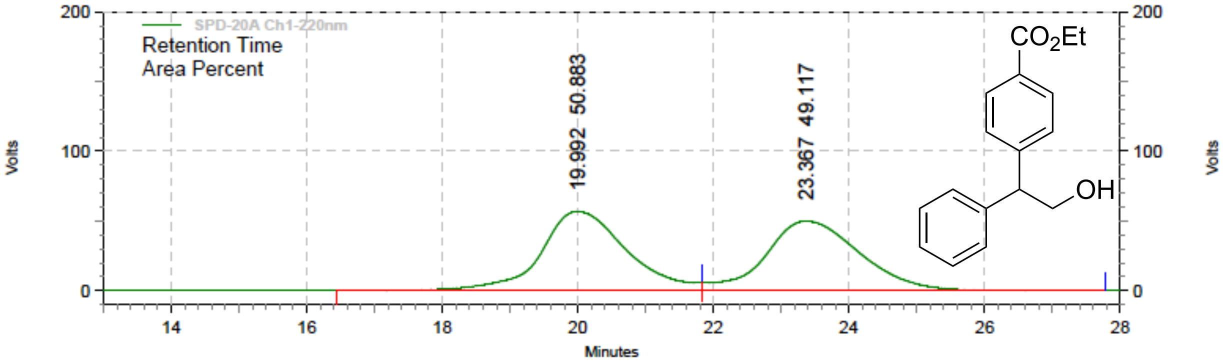

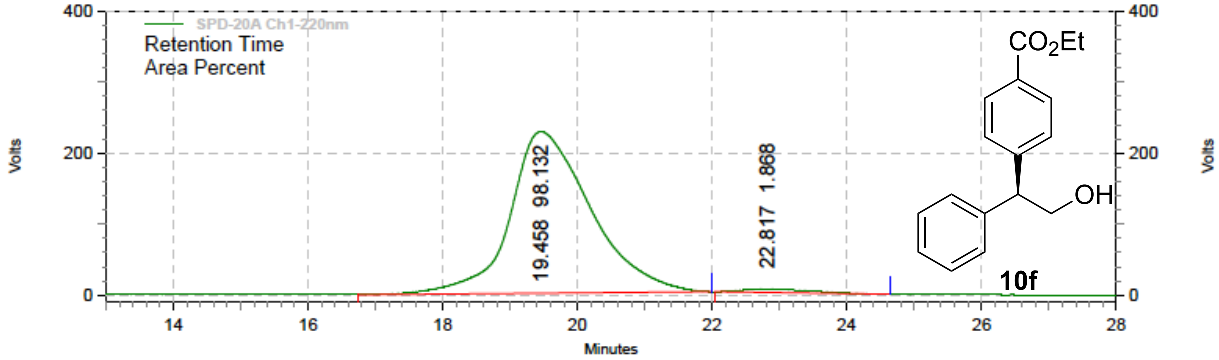

Conditions and results:

Column	OJ-RH
Mobile phase	35:65 = CH_3CN:H_2O
Flow	1 mL/min
Detector	220 nm
Temp	25 °C

Figure S29. HPLC traces of racemic and enantioenriched **10f**.

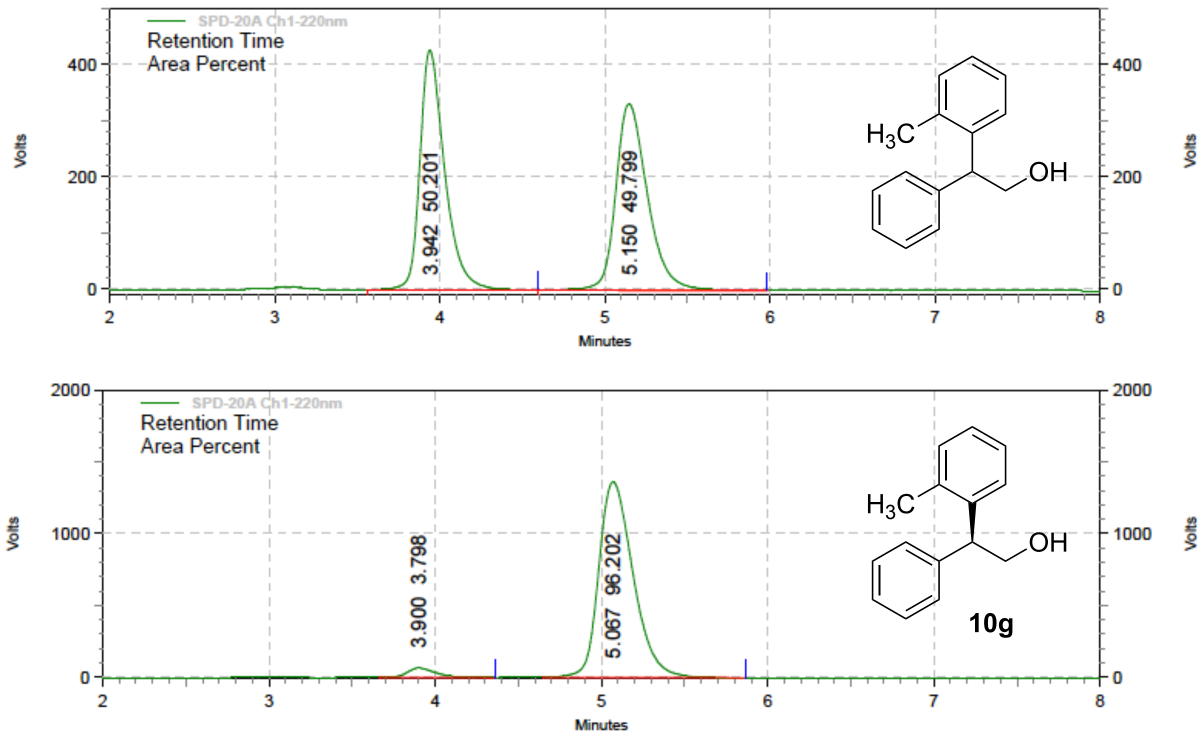

Conditions and results:

Column	OJ-RH
Mobile phase	75:25 = CH_3CN:H_2O (0.1% phosphoric acid buffer pH=3)
Flow	0.8 mL/min
Detector	220 nm
Temp	25 ºC

Figure S30. HPLC traces of racemic and enantioenriched **10g**.

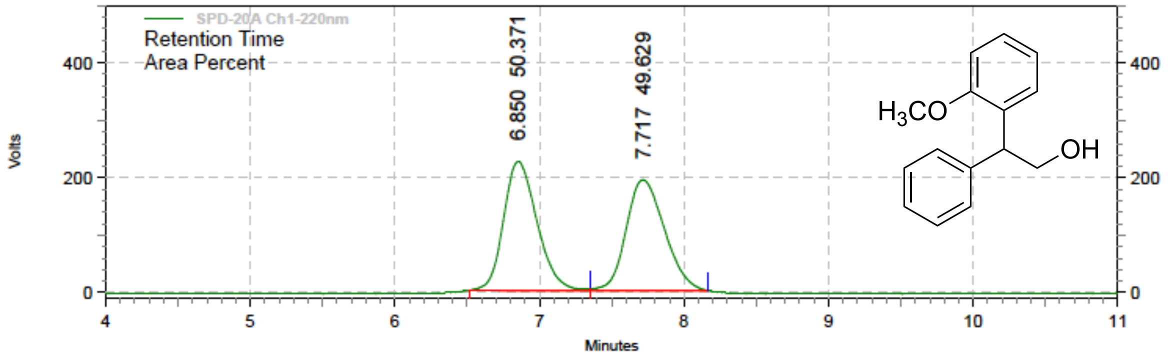

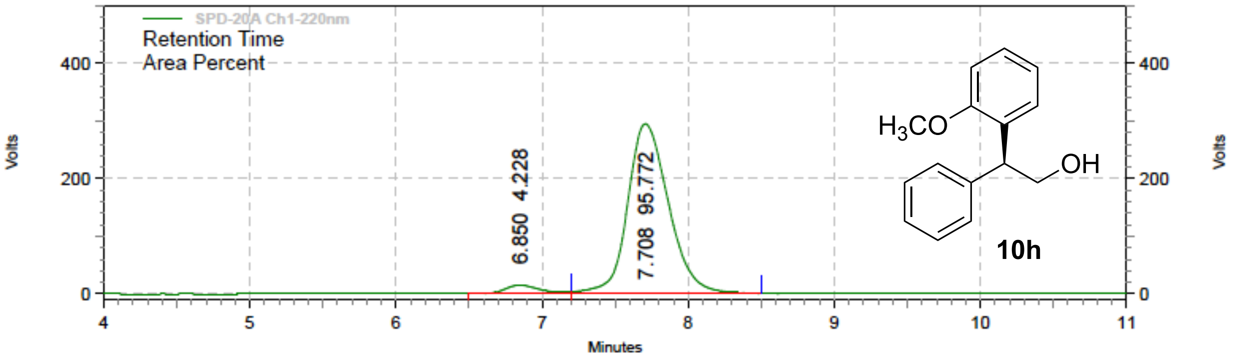

10h

Conditions and results:

Column	OJ-RH
Mobile phase	50:50 = CH_3CN:H_2O
Flow	0.8 mL/min
Detector	220 nm
Temp	25 ºC

Figure S31. HPLC traces of racemic and enantioenriched **10h**.

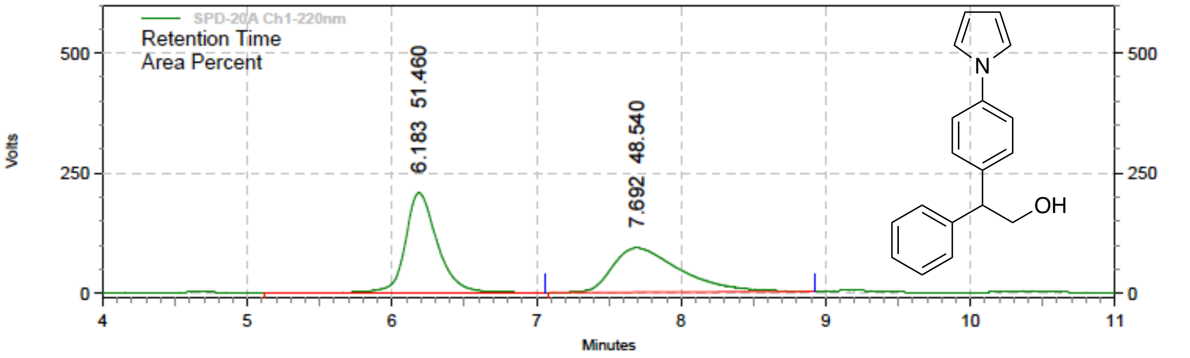

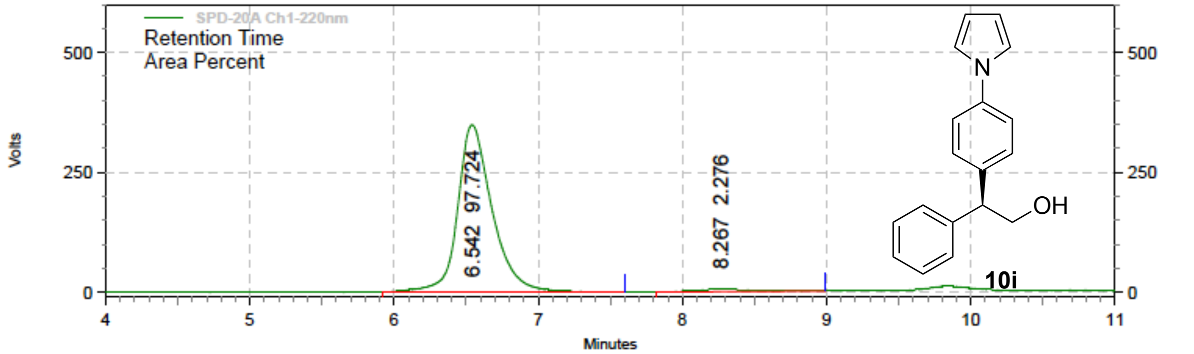

Conditions and results:

Column	OJ-RH
Mobile phase	65:35 = CH_3CN:H_2O
Flow	0.8 mL/min
Detector	220 nm
Temp	25 ºC

Figure S32. HPLC traces of racemic and enantioenriched **10i**.

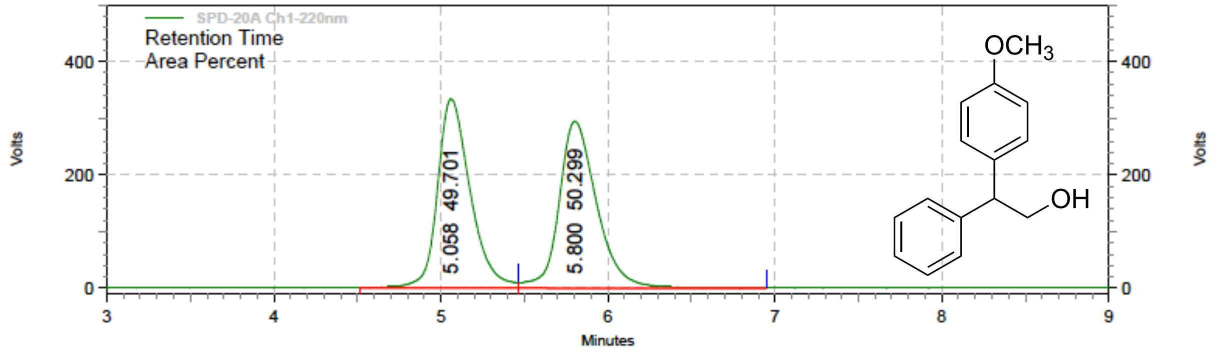

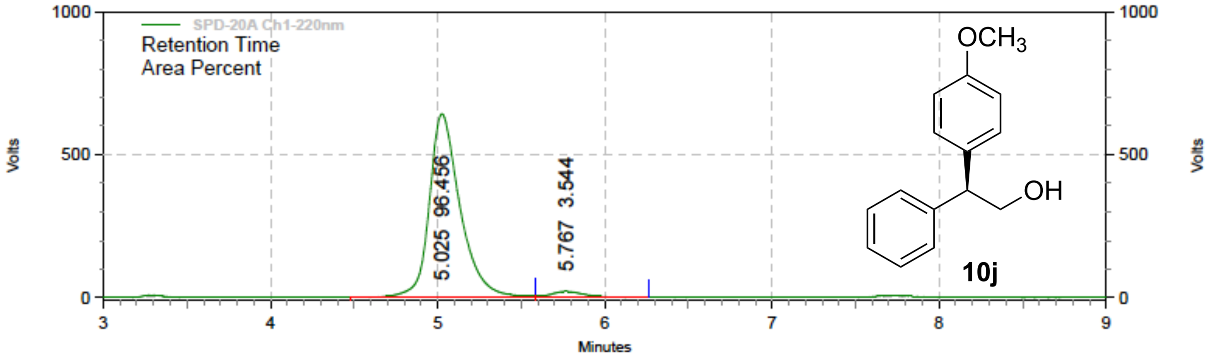

Conditions and results:

Column	OJ-RH
Mobile phase	60:40 = CH_3CN:H_2O
Flow	0.8 mL/min
Detector	220 nm
Temp	25 ºC

Figure S33. HPLC traces of racemic and enantioenriched **10j**.

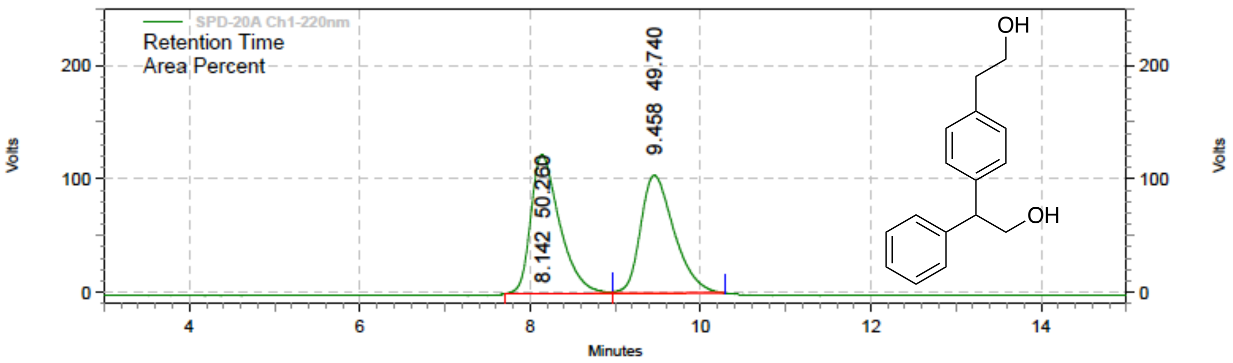

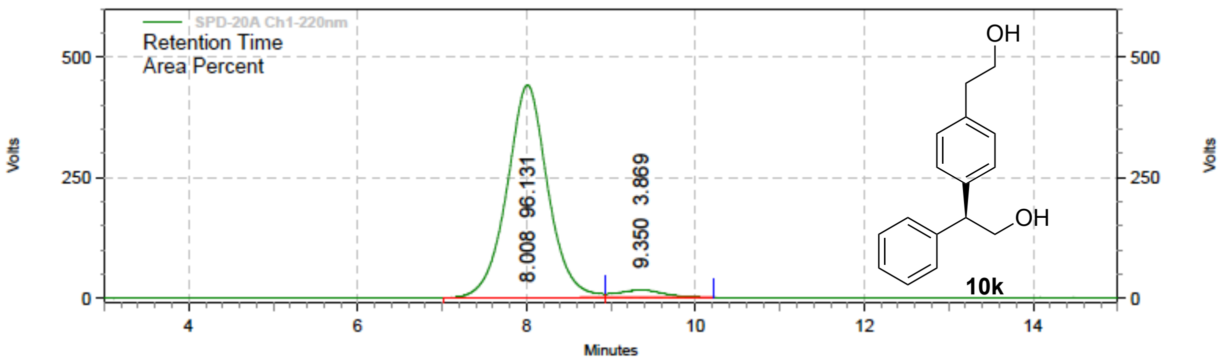

Conditions and results:

Column	OJ-RH
Mobile phase	30:70 = $CH_3CN:H_2O$
Flow	0.8 mL/min
Detector	220 nm
Temp	25 ºC

Figure S34. HPLC traces of racemic and enantioenriched **10k**.

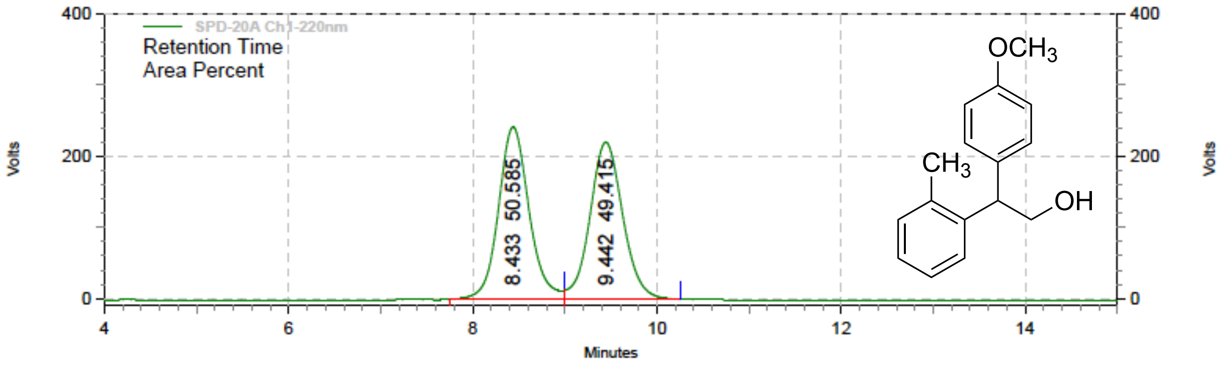

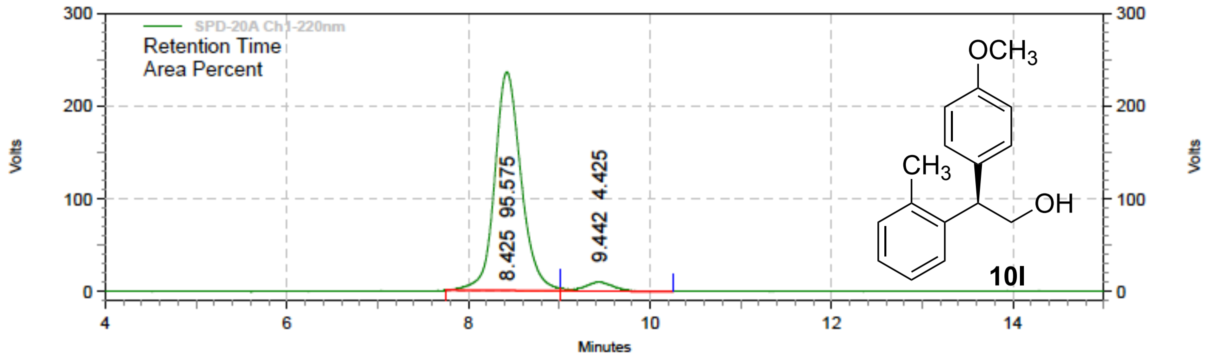

Conditions and results:

Column	OJ-RH
Mobile phase	45:55 = CH_3CN:H_2O
Flow	1 mL/min
Detector	220 nm
Temp	25 ºC

Figure S35. HPLC traces of racemic and enantioenriched **10l**.

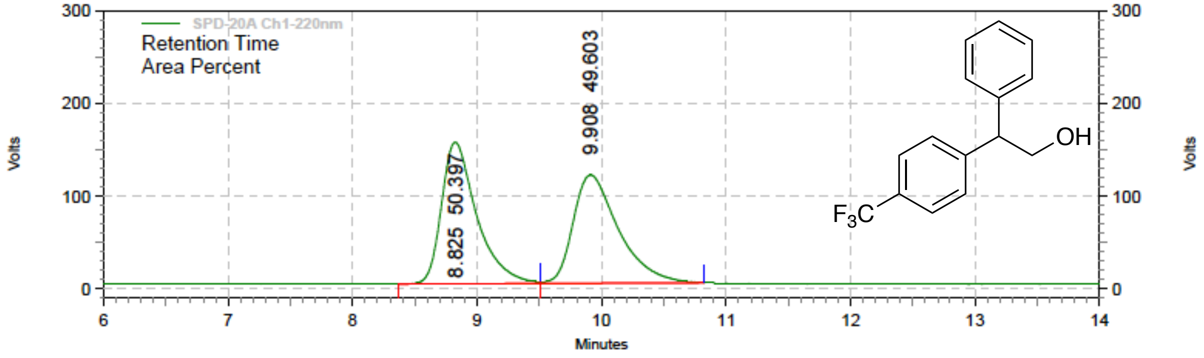

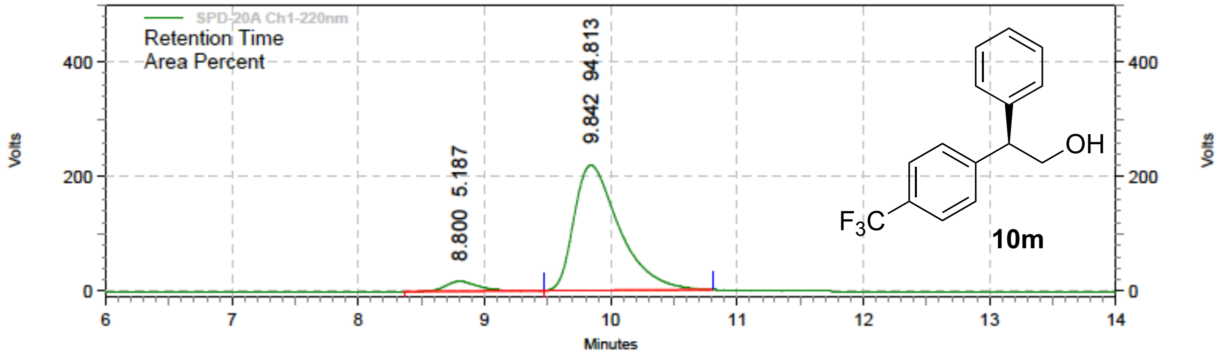

Conditions and results:

Column	IA
Mobile phase	55:45 = CH_3CN:H_2O
Flow	1 mL/min
Detector	220 nm
Temp	25 ºC

Figure S36. HPLC traces of racemic and enantioenriched **10m**.

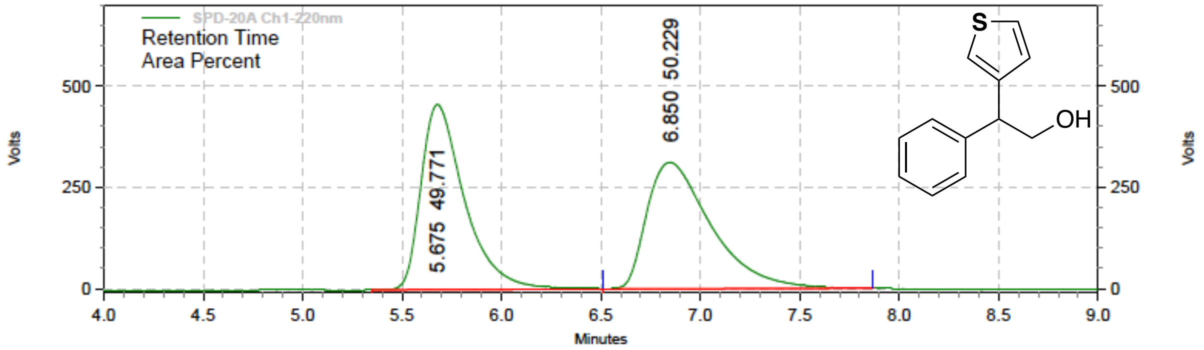

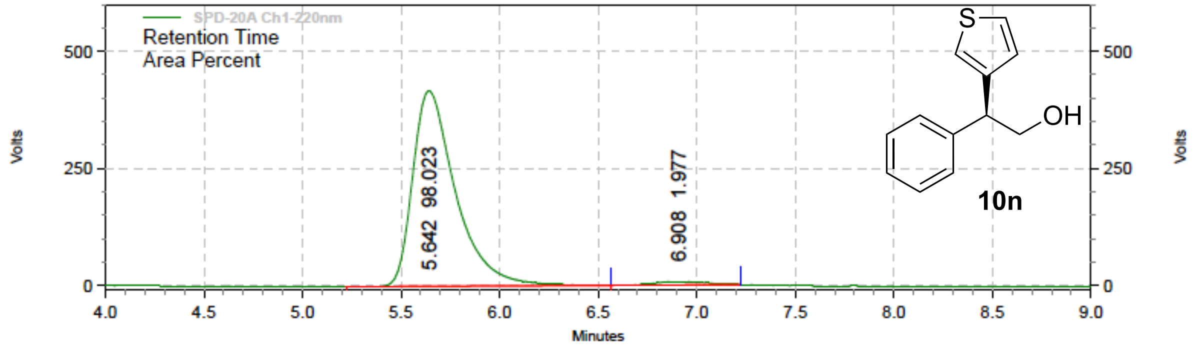

Conditions and results:

Column	IA
Mobile phase	65:35 = $CH_3CN:H_2O$
Flow	1 mL/min
Detector	220 nm
Temp	25 ºC

Figure S37. HPLC traces of racemic and enantioenriched **10n**.

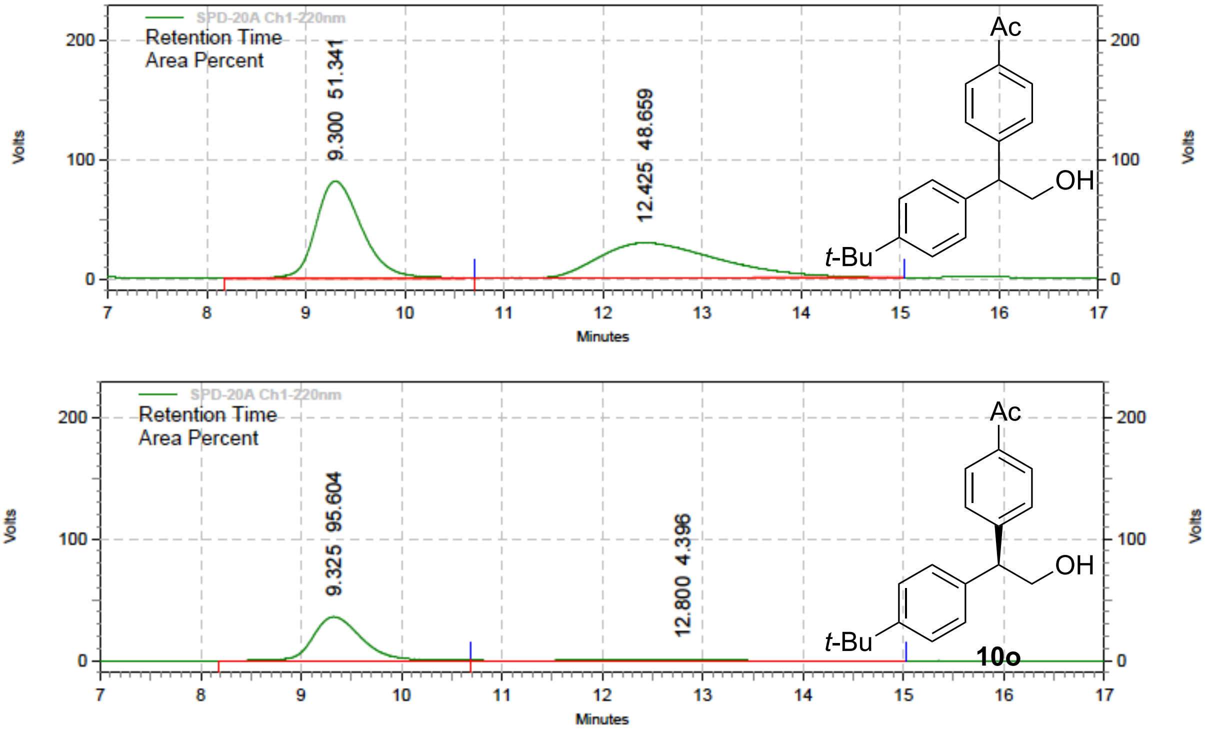

Conditions and results:

Column	OJ-RH
Mobile phase	50:50 = $CH_3CN:H_2O$
Flow	0.8 mL/min
Detector	220 nm
Temp	25 ºC

Figure S38. HPLC traces of racemic and enantioenriched **10o**.

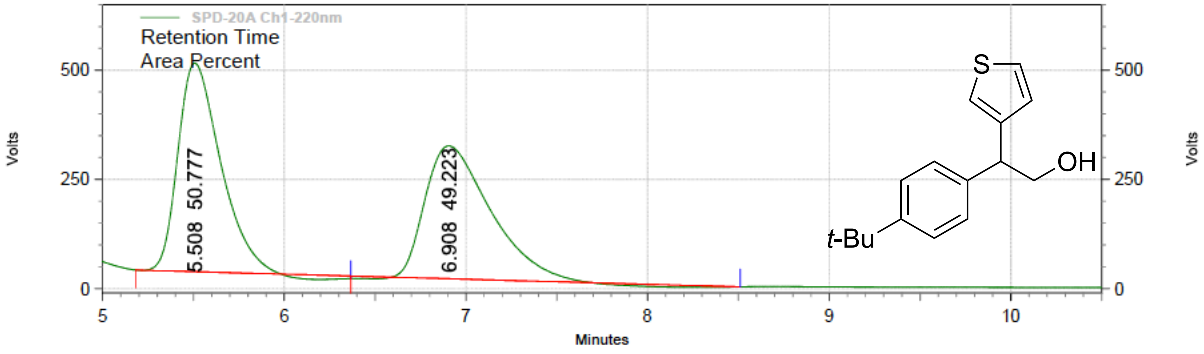

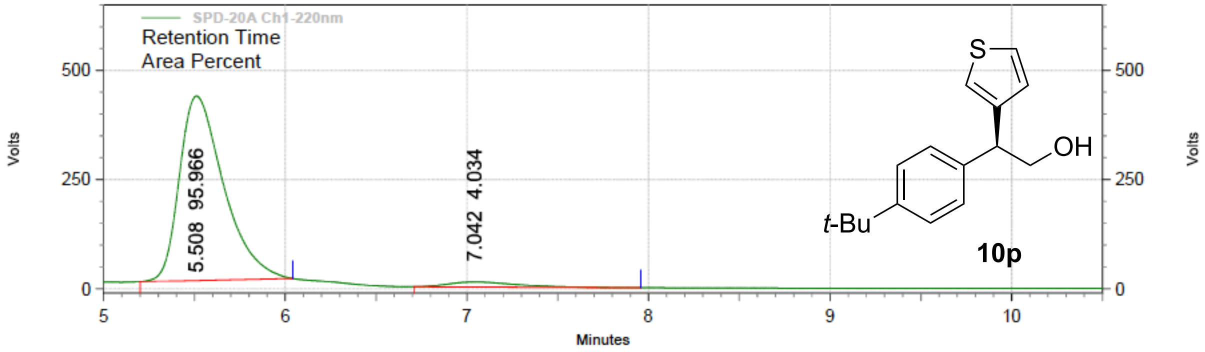

Conditions and results:

Column	IA
Mobile phase	80:20 = CH_3CN:H_2O
Flow	1 mL/min
Detector	220 nm
Temp	25 ºC

Figure S39. HPLC traces of racemic and enantioenriched **10p**.

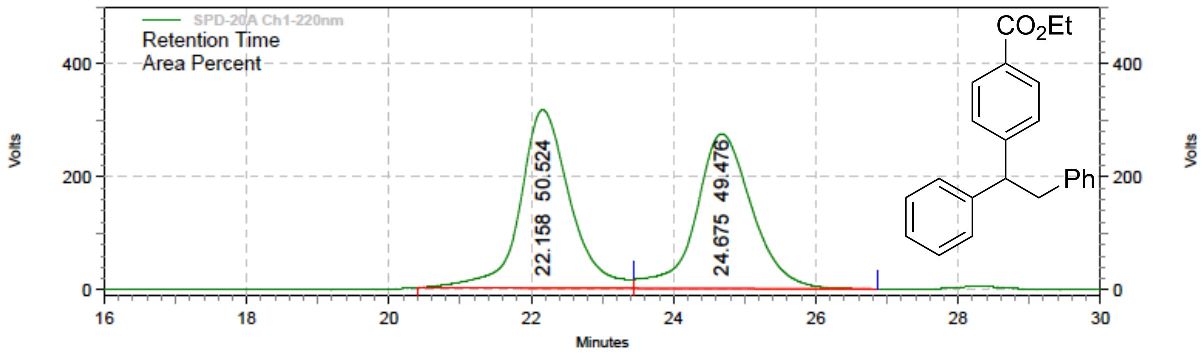

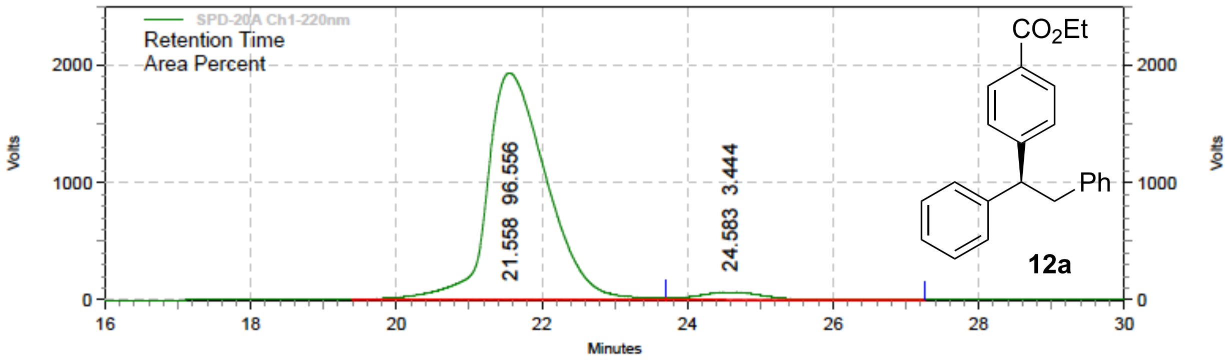

Conditions and results:

Column	OJ-RH
Mobile phase	55:45 = CH_3CN:H_2O
Flow	1 mL/min
Detector	220 nm
Temp	25 ºC

Figure S40. HPLC traces of racemic and enantioenriched **12a**.

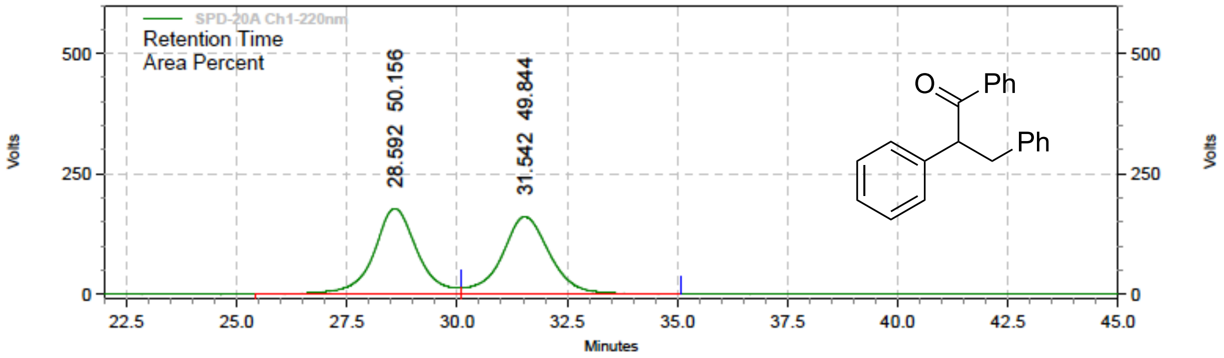

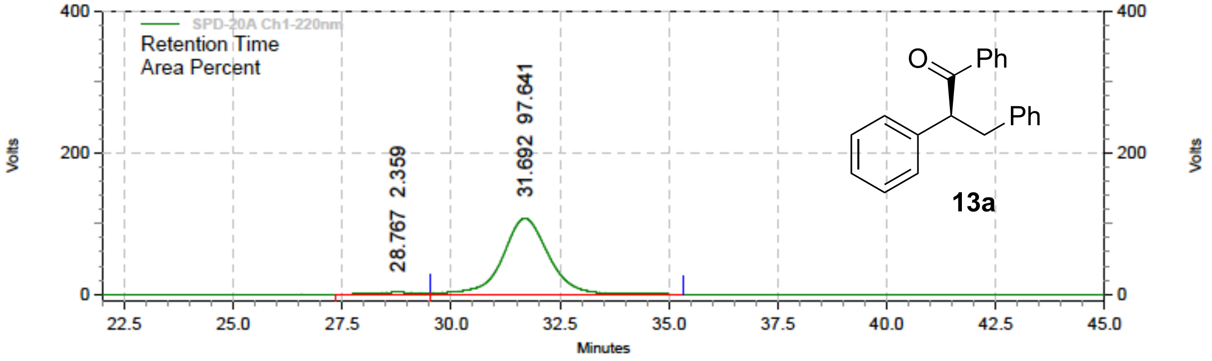

Conditions and results:

Column	OJ-RH
Mobile phase	50:50 = CH_3CN:H_2O
Flow	0.8 mL/min
Detector	220 nm
Temp	25 ºC

Figure S41. HPLC traces of racemic and enantioenriched **13a**.

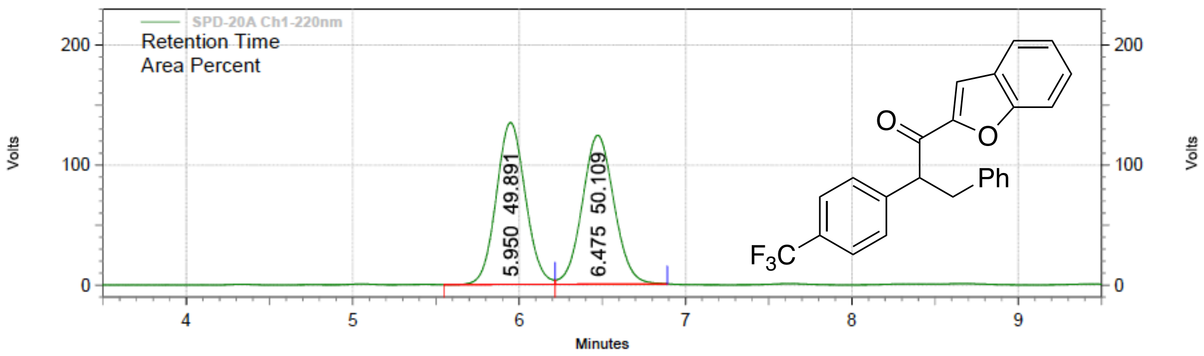

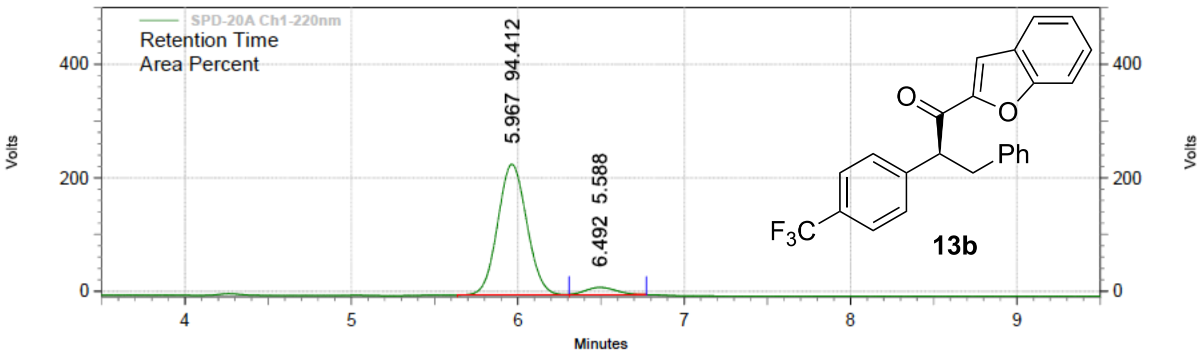

Conditions and results:

Column	OD-RH
Mobile phase	75:25 = CH_3CN:H_2O
Flow	1 mL/min
Detector	220 nm
Temp	25 ºC

Figure S42. HPLC traces of racemic and enantioenriched **13b**.

8. ^{1}H, ^{13}C, ^{119}Sn NMR Spectra

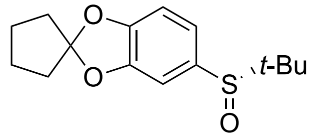

S3

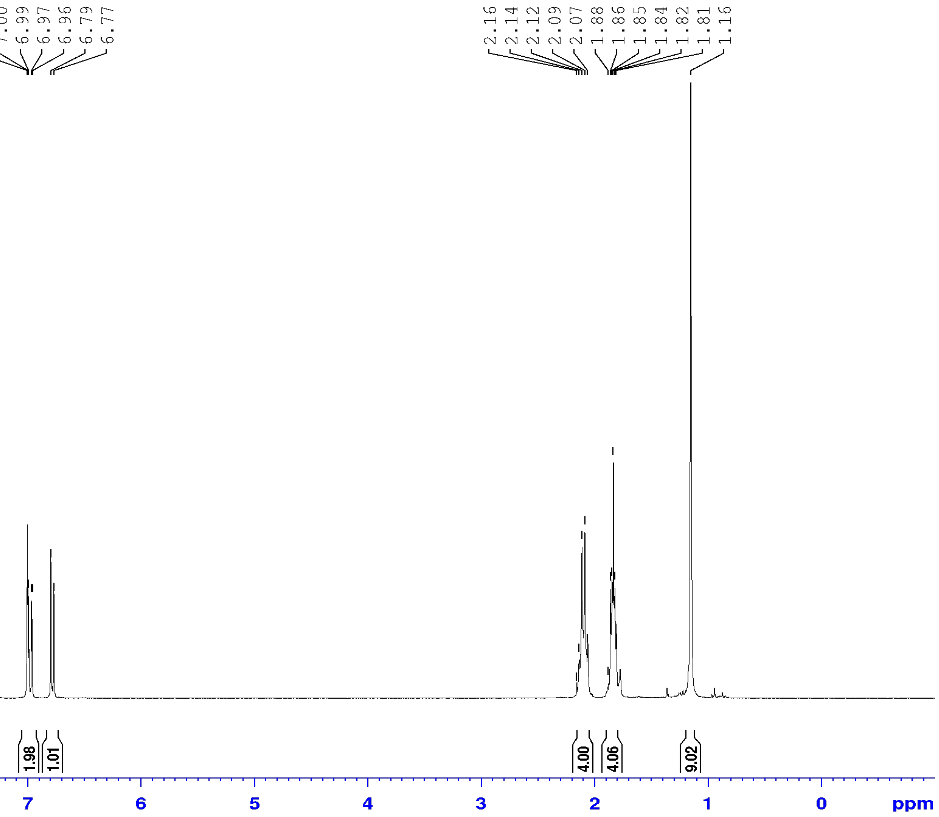

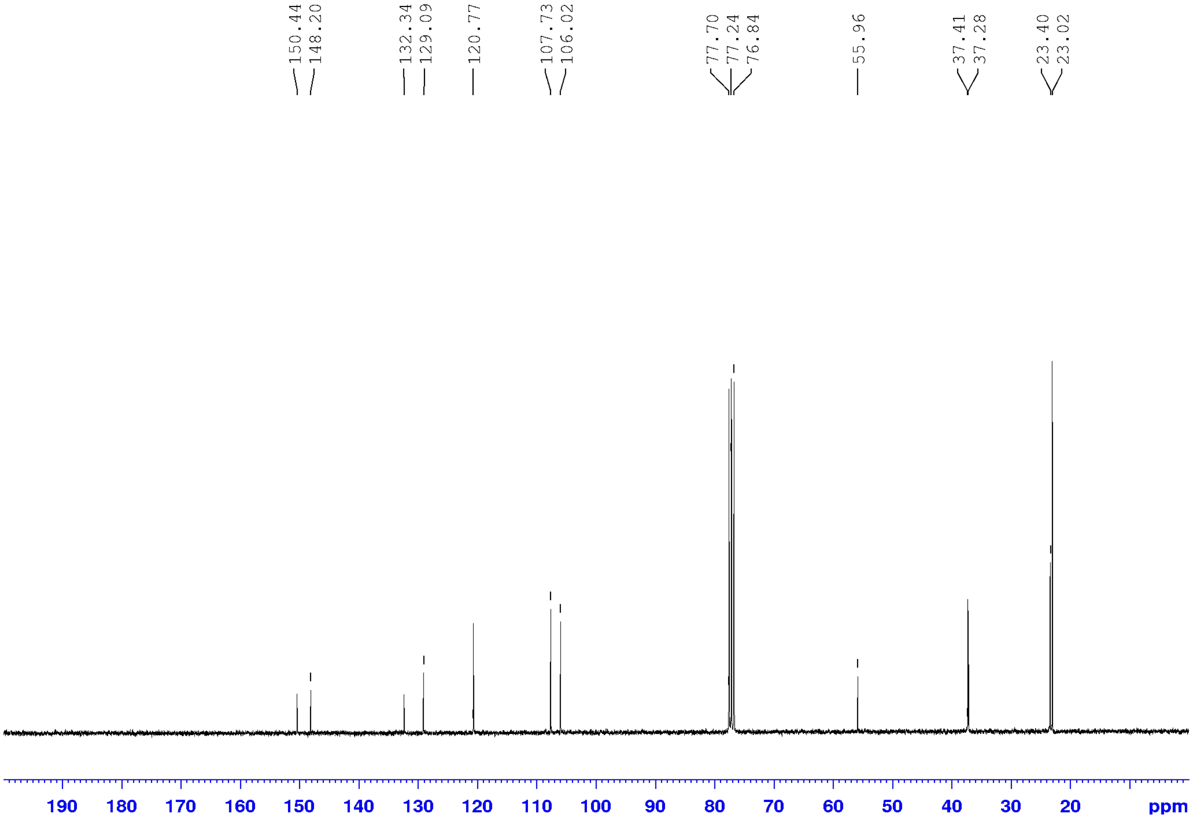

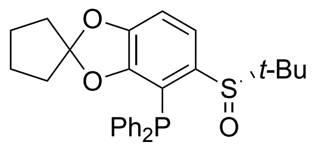
O
O
t-Bu
S
O
Ph2P

7

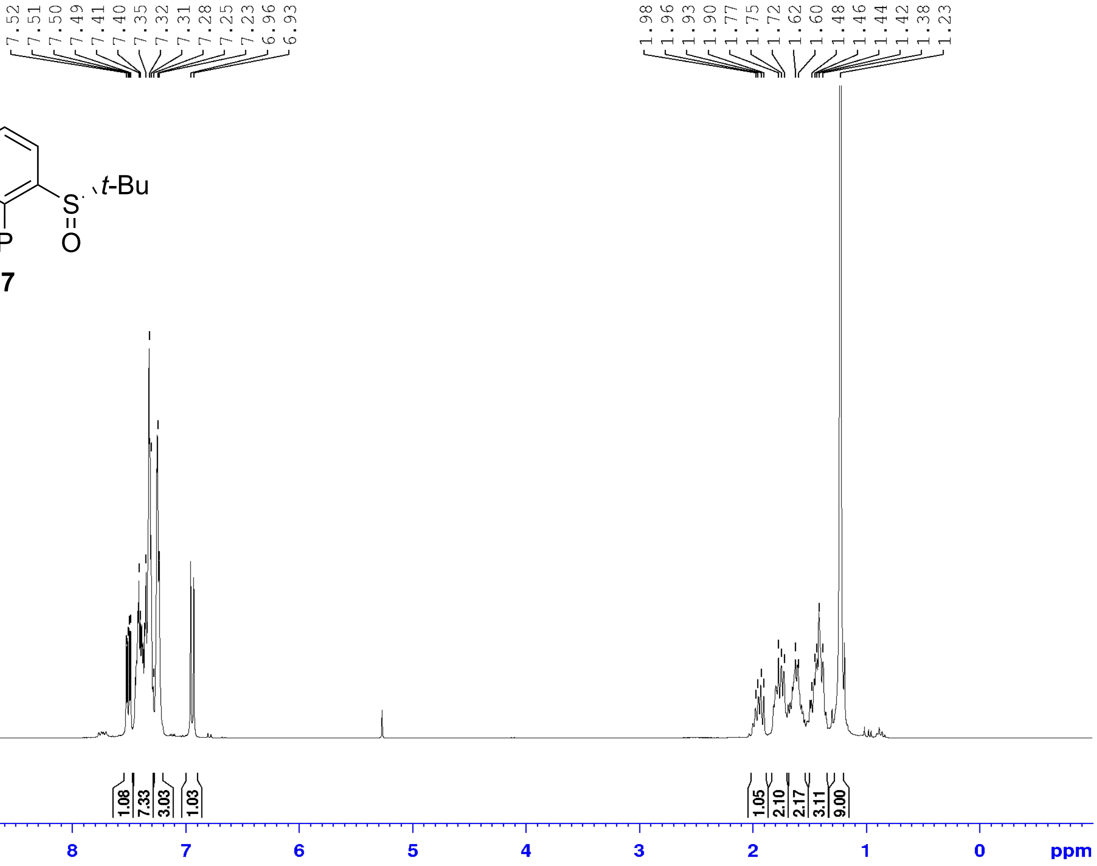
7.52
7.51
7.50
7.49
7.41
7.40
7.35
7.32
7.31
7.28
7.25
7.23
6.96
6.93
1.98
1.96
1.93
1.90
1.77
1.75
1.72
1.62
1.60
1.48
1.46
1.44
1.42
1.38
1.23
1.08
7.33
3.03
1.03
1.05
2.10
2.17
3.11
9.00
9
8
7
6
5
4
3
2
1
0
ppm

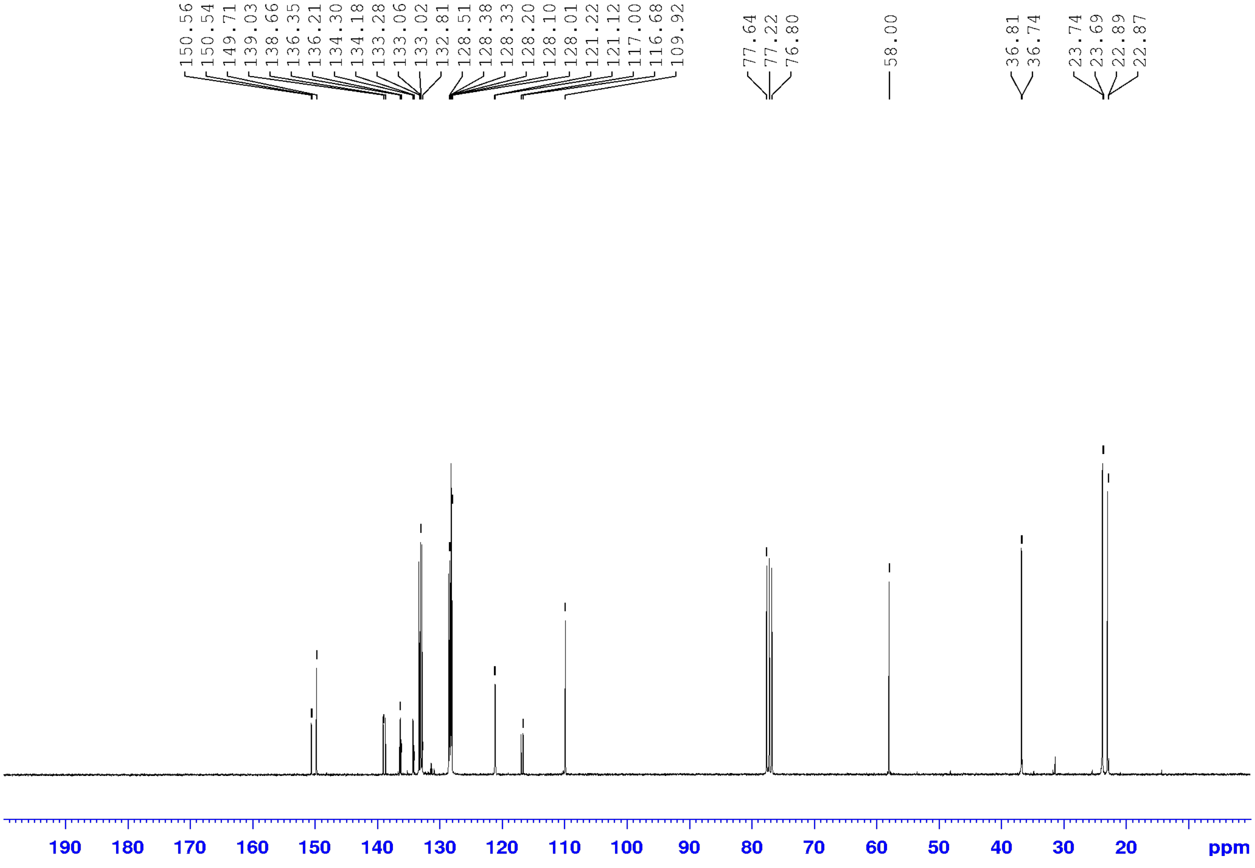
150.56
150.54
149.71
139.03
138.66
136.35
136.21
134.30
134.18
133.28
133.06
133.02
132.81
128.51
128.38
128.33
128.20
128.10
128.01
121.22
121.12
117.00
116.68
109.92
77.64
77.22
76.80
58.00
36.81
36.74
23.74
23.69
22.89
22.87
190
180
170
160
150
140
130
120
110
100
90
80
70
60
50
40
30
20
ppm

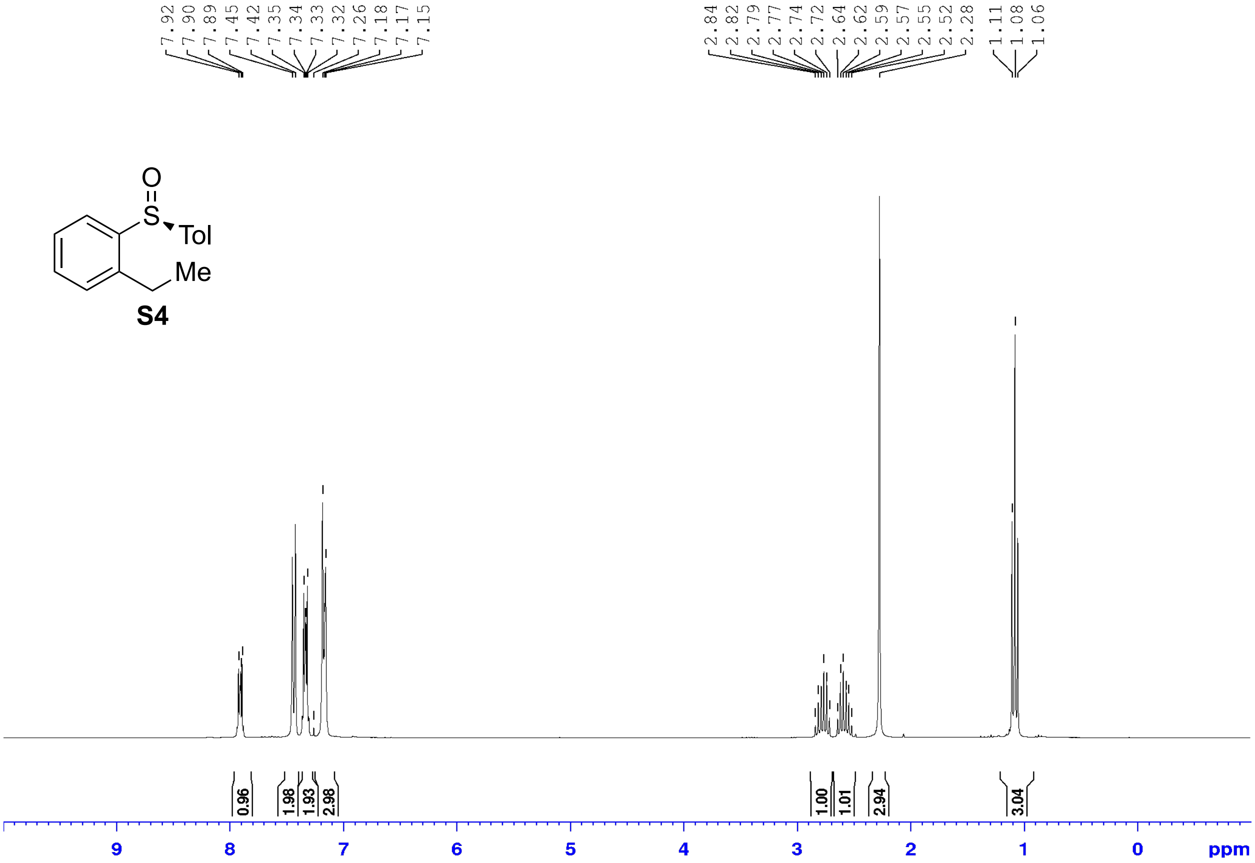
O
S
Tol
Me
7.92
7.90
7.89
7.45
7.42
7.35
7.34
7.33
7.32
7.26
7.18
7.17
7.15
2.84
2.82
2.79
2.77
2.74
2.72
2.64
2.62
2.59
2.57
2.55
2.52
2.28
1.11
1.08
1.06
0.96
1.98
1.93
2.98
1.00
1.01
2.94
3.04
9
8
7
6
5
4
3
2
1
0
ppm

S4

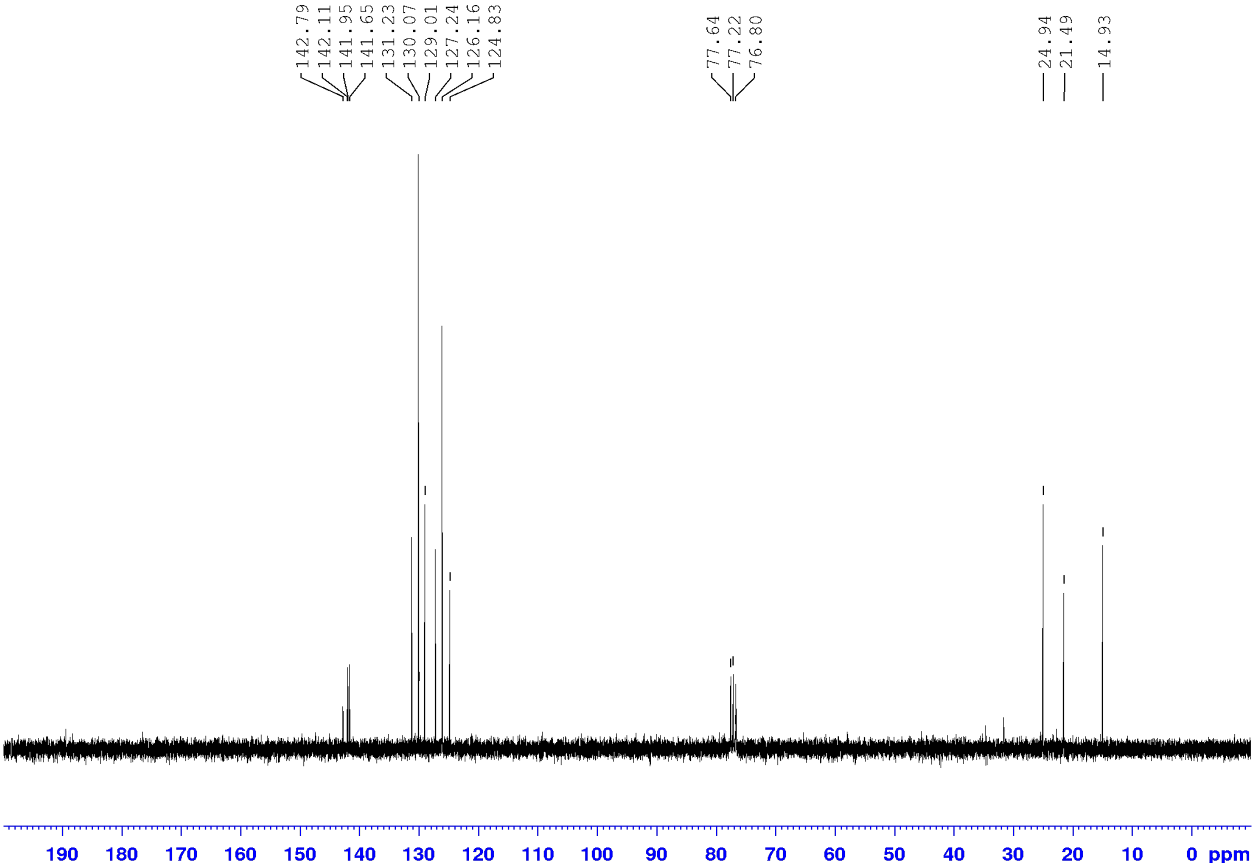
142.79
142.11
141.95
141.65
131.23
130.07
129.01
127.24
126.16
124.83
77.64
77.22
76.80
24.94
21.49
14.93
190
180
170
160
150
140
130
120
110
100
90
80
70
60
50
40
30
20
10
0 ppm

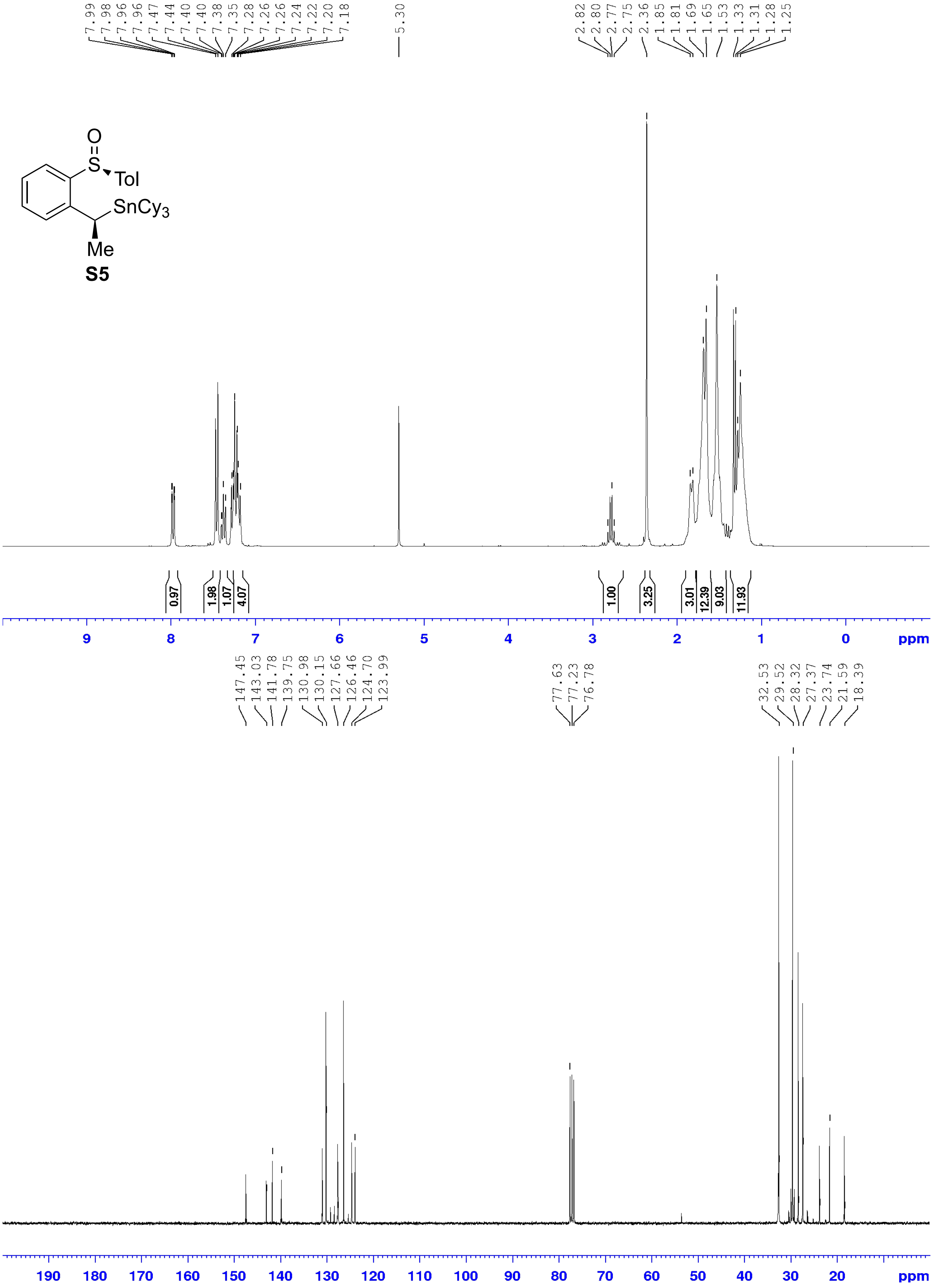

7.99 7.98 7.96 7.96 7.47 7.44 7.40 7.40 7.38 7.35 7.28 7.26 7.26 7.24 7.22 7.20 7.18
5.30
2.82 2.80 2.77 2.75 2.36 1.85 1.81 1.69 1.65 1.53 1.33 1.31 1.28 1.25
O
S
Tol
SnCy3
Me
S5
0.97 1.98 1.07 4.07 1.00 3.25 3.01 12.39 9.03 11.93
9 8 7 6 5 4 3 2 1 0 ppm
147.45 143.03 141.78 139.75 130.98 130.15 127.66 126.46 124.70 123.99
77.63 77.23 76.78
32.53 29.52 28.32 27.37 23.74 21.59 18.39
190 180 170 160 150 140 130 120 110 100 90 80 70 60 50 40 30 20 ppm

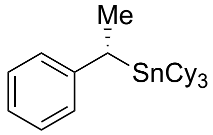
Me
SnCy3

3b

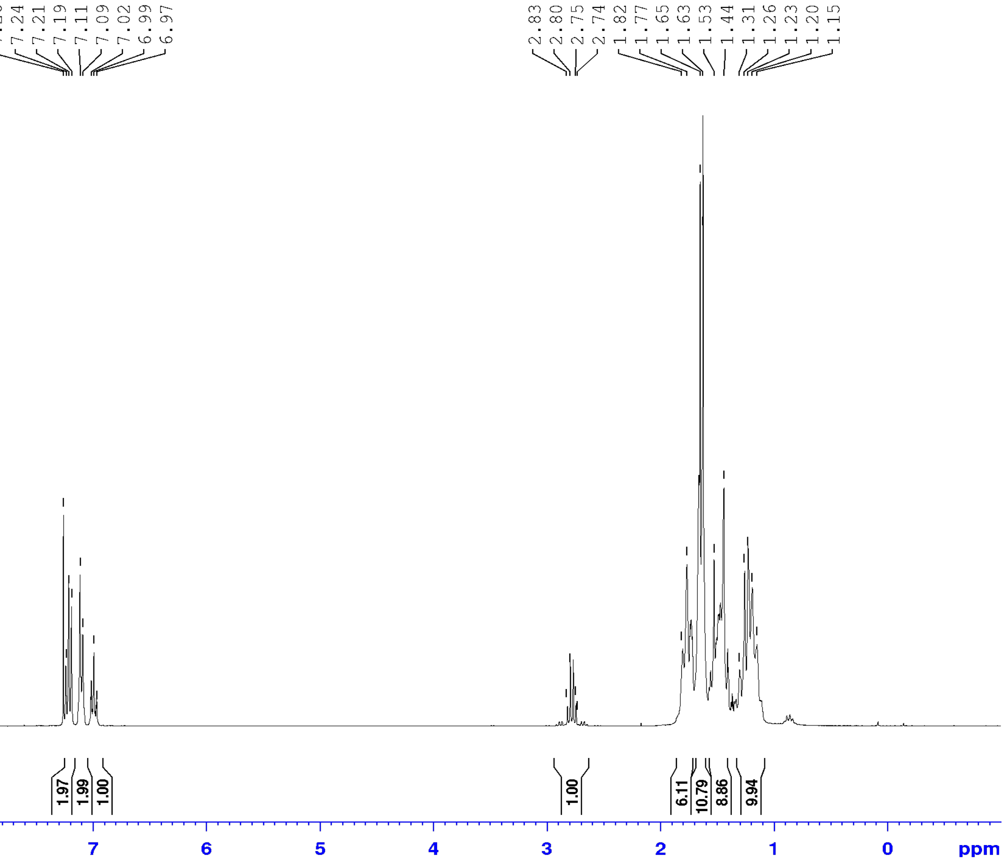
7.26
7.24
7.21
7.19
7.11
7.09
7.02
6.99
6.97
2.83
2.80
2.75
2.74
1.82
1.77
1.65
1.63
1.53
1.44
1.31
1.26
1.23
1.20
1.15
1.97
1.99
1.00
1.00
6.11
10.79
8.86
9.94
9
8
7
6
5
4
3
2
1
0
ppm

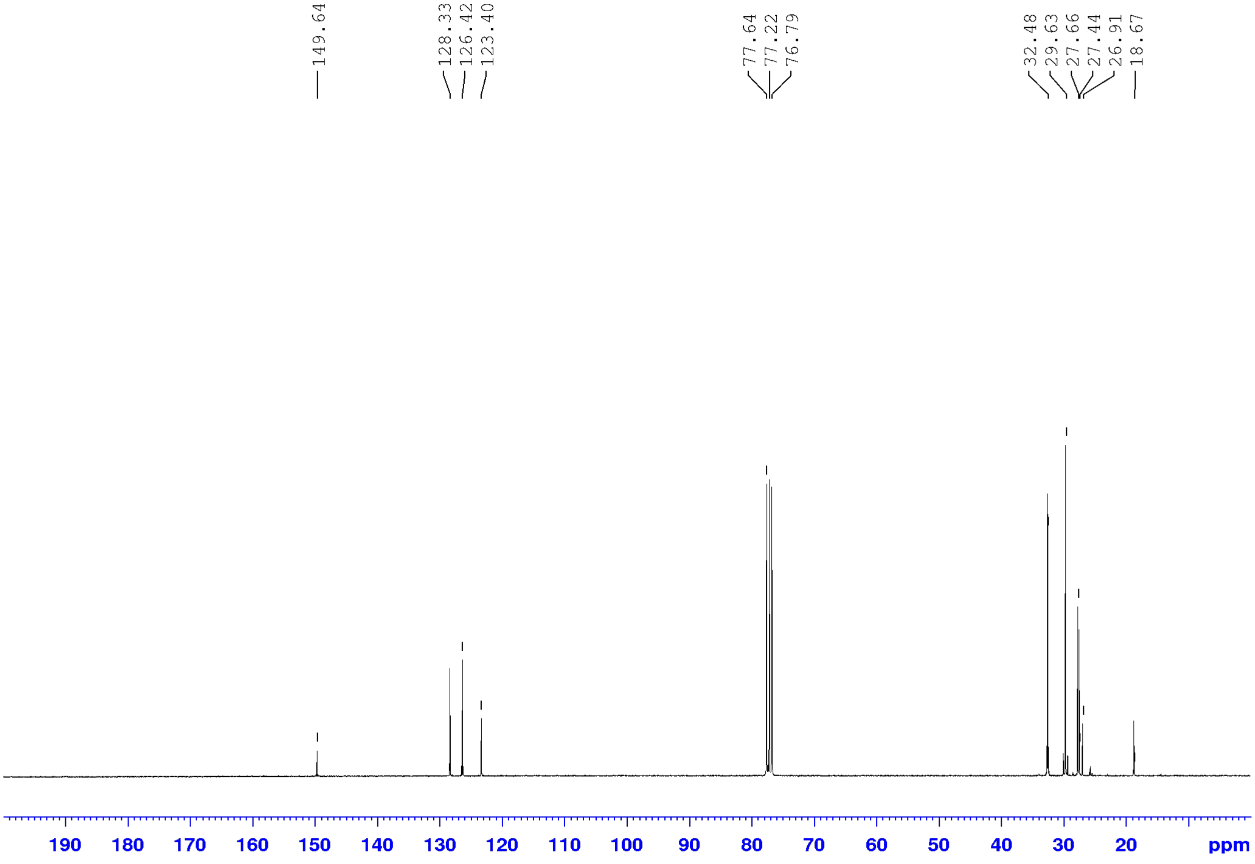
149.64
128.33
126.42
123.40
77.64
77.22
76.79
32.48
29.63
27.66
27.44
26.91
18.67
190
180
170
160
150
140
130
120
110
100
90
80
70
60
50
40
30
20
ppm

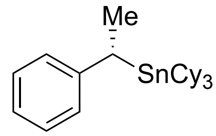

3b

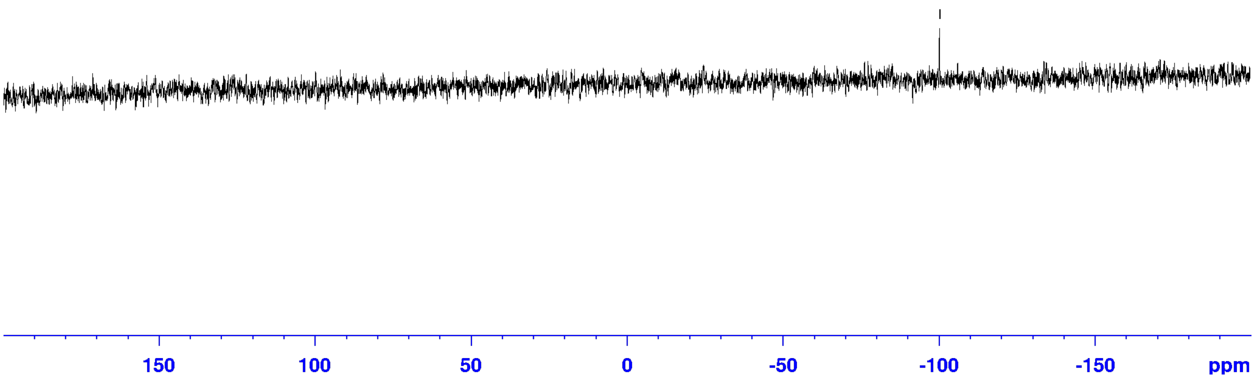

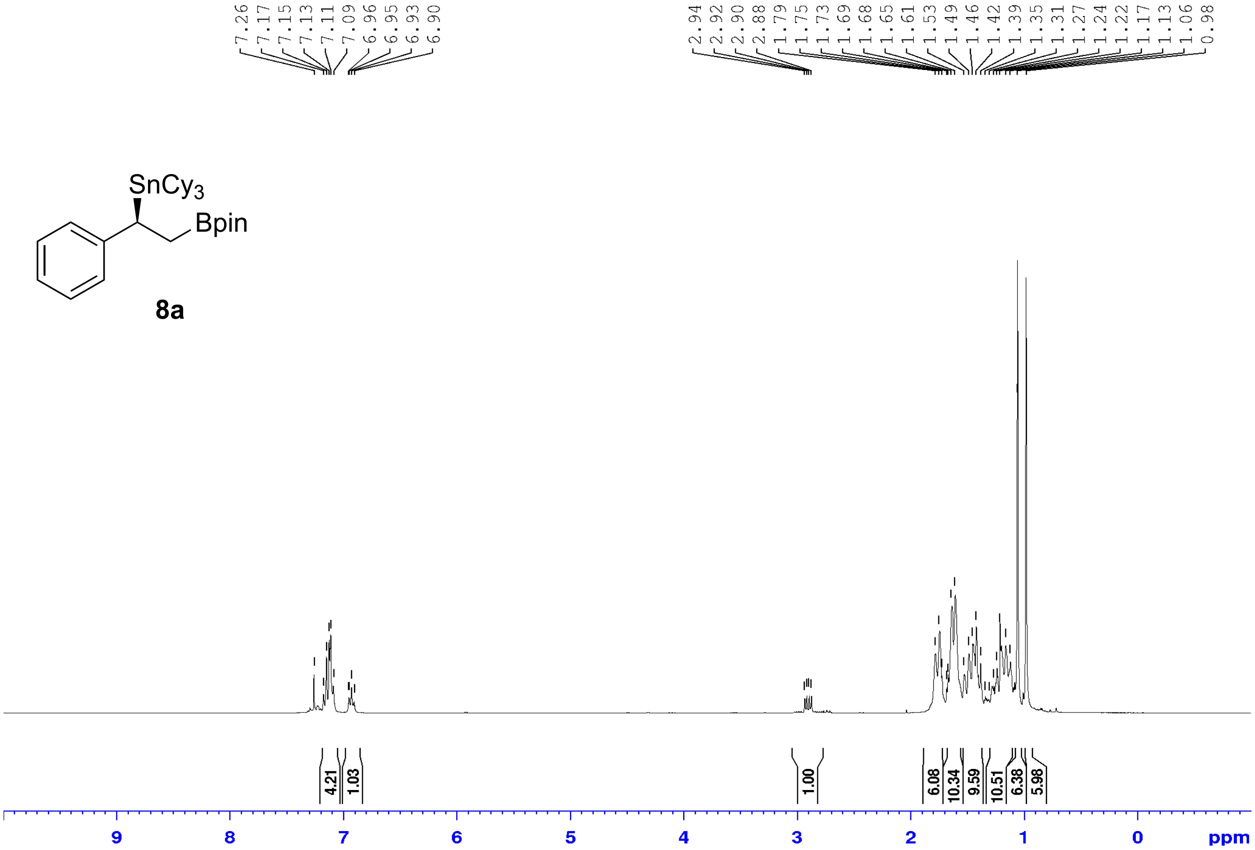

7.26 7.17 7.15 7.13 7.11 7.09 6.96 6.95 6.93 6.90
2.94 2.92 2.90 2.88 1.79 1.75 1.73 1.69 1.68 1.65 1.61 1.53 1.49 1.46 1.42 1.39 1.35 1.31 1.27 1.24 1.22 1.17 1.13 1.06 0.98
SnCy$_3$
Bpin
8a
4.21 1.03 1.00 6.08 10.34 9.59 10.51 6.38 5.98
9 8 7 6 5 4 3 2 1 0 ppm

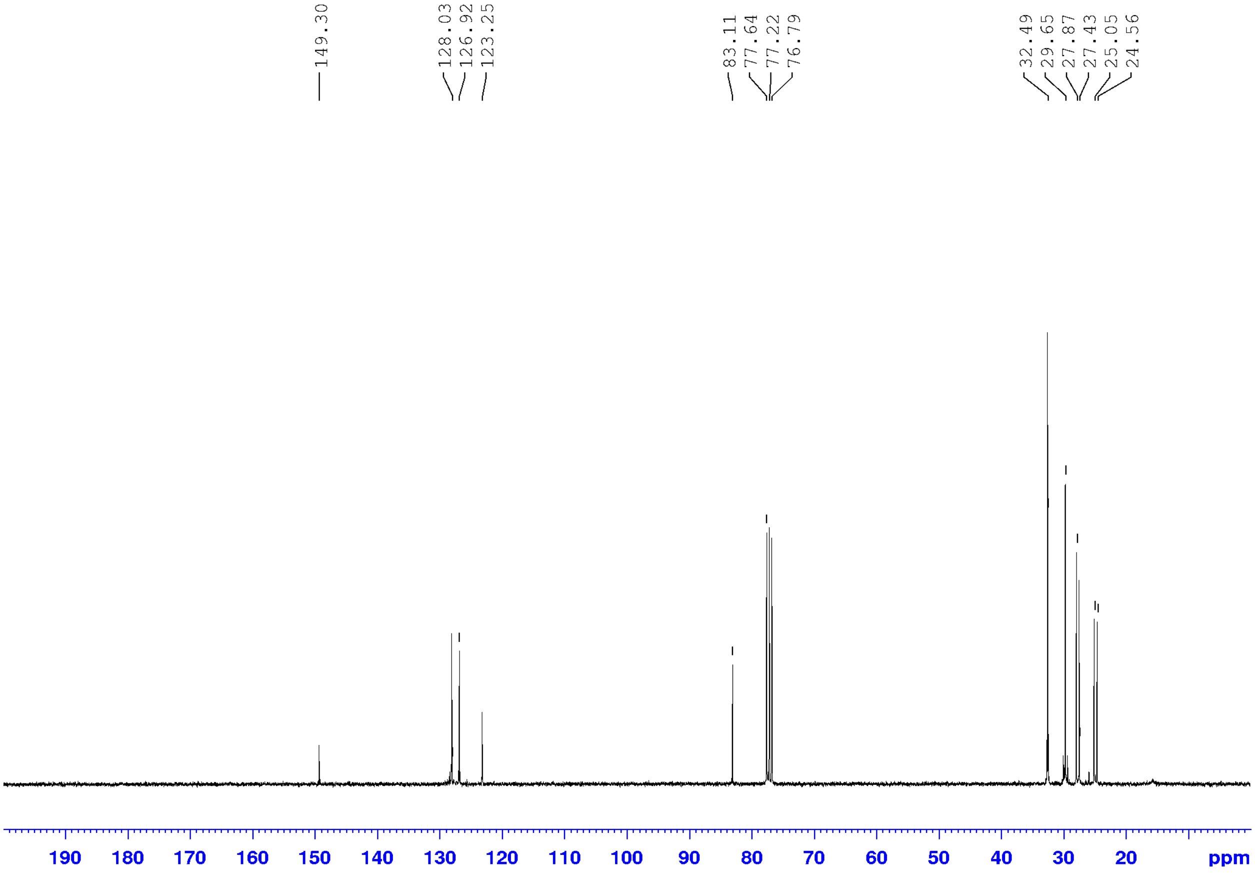

149.30 128.03 126.92 123.25 83.11 77.64 77.22 76.79 32.49 29.65 27.87 27.43 25.05 24.56
190 180 170 160 150 140 130 120 110 100 90 80 70 60 50 40 30 20 ppm

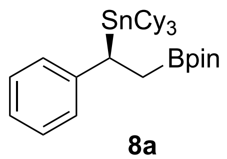

8a

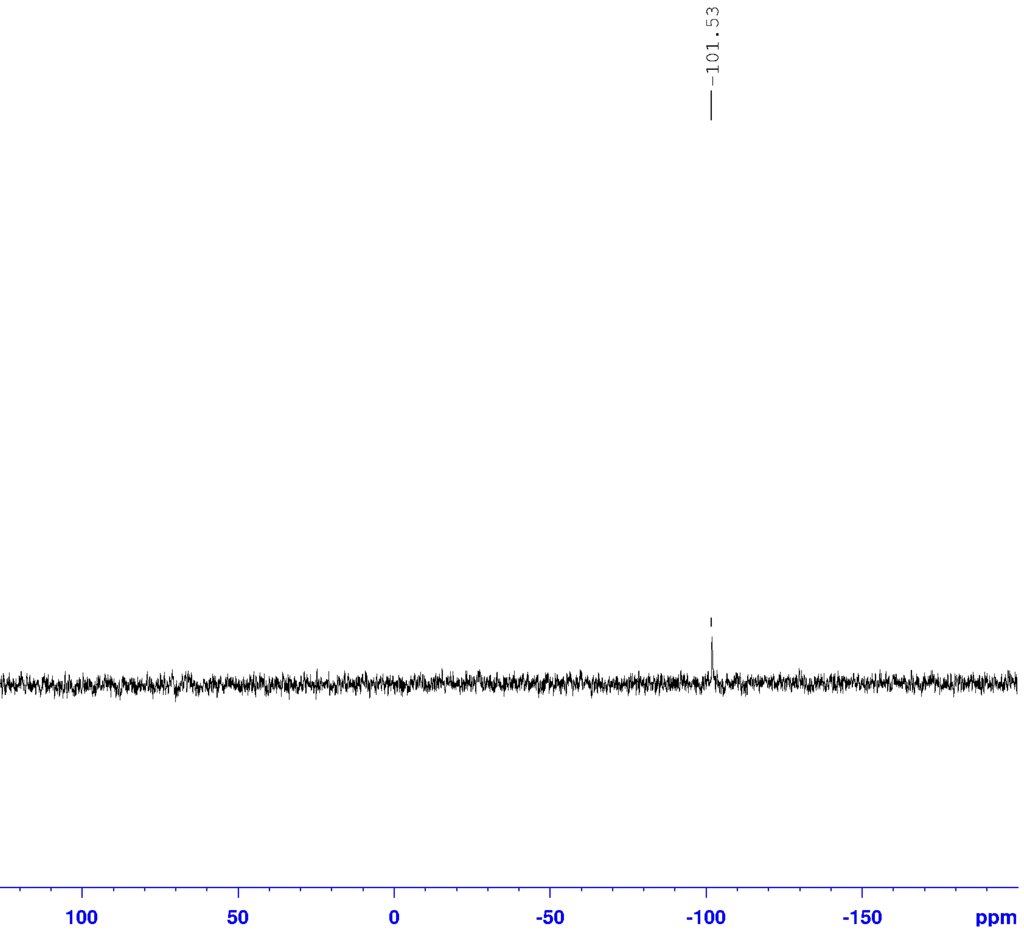

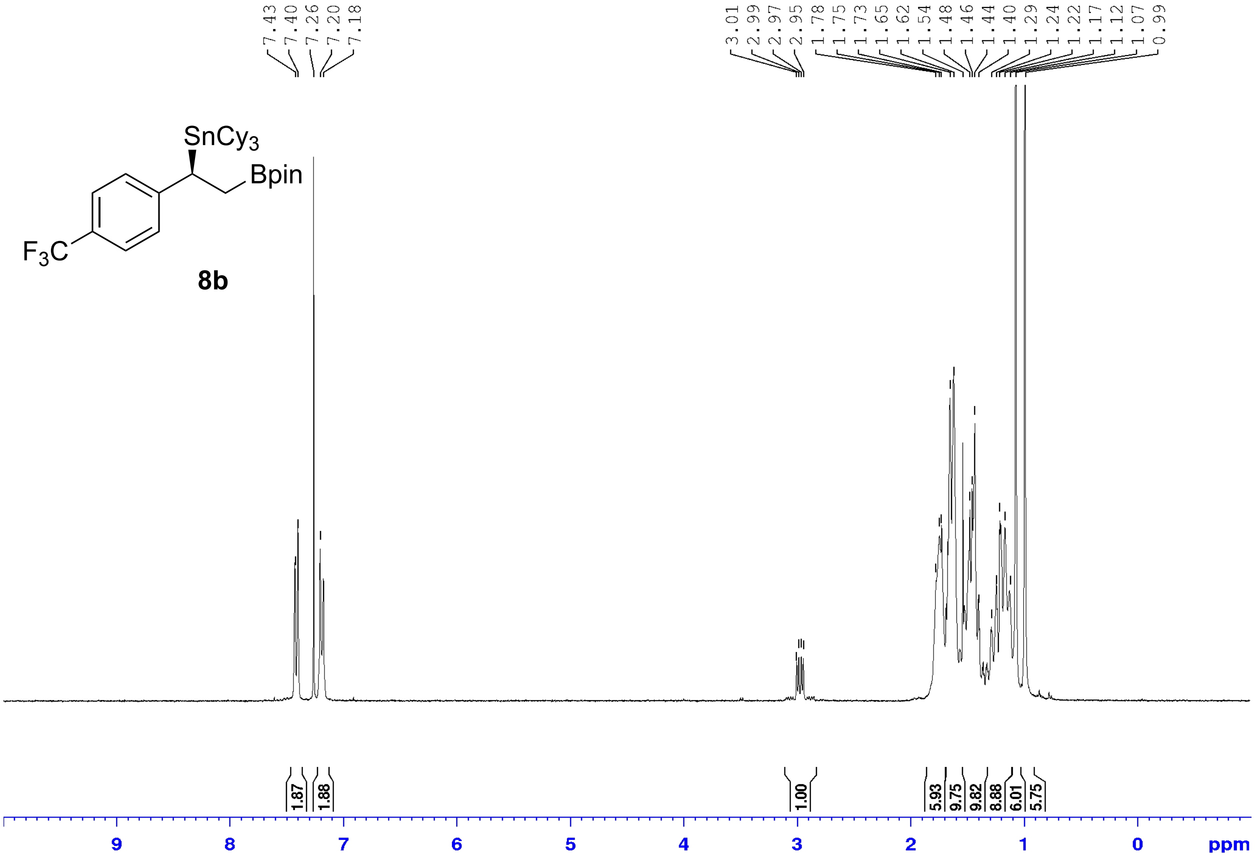
SnCy3
Bpin
F3C
8b
7.43 7.40 7.26 7.20 7.18
3.01 2.99 2.97 2.95 1.78 1.75 1.73 1.65 1.62 1.54 1.48 1.46 1.44 1.40 1.29 1.24 1.22 1.17 1.12 1.07 0.99
1.87 1.88 1.00 5.93 9.75 9.82 8.88 6.01 5.75
9 8 7 6 5 4 3 2 1 0 ppm

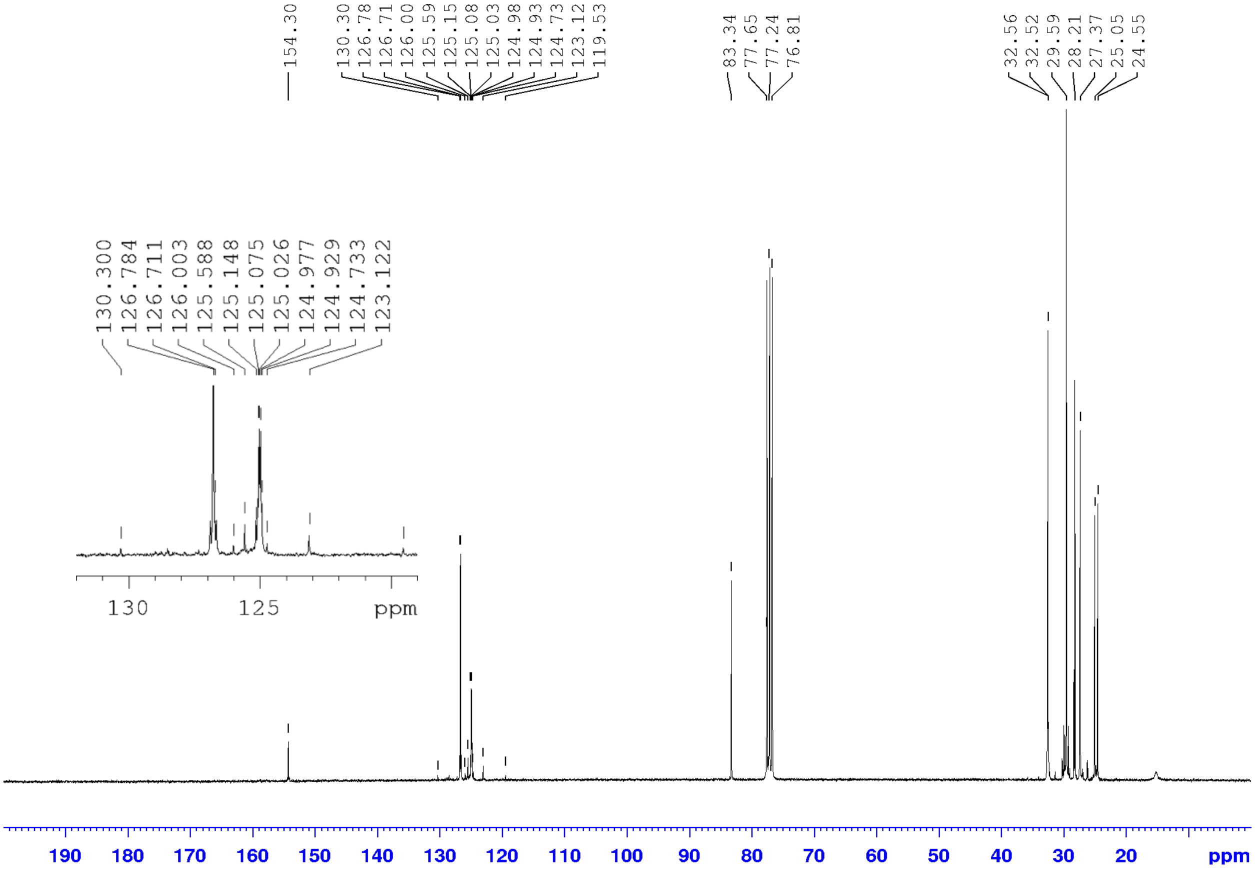
154.30
130.30 126.78 126.71 126.00 125.59 125.15 125.08 125.03 124.98 124.93 124.73 123.12 119.53
83.34 77.65 77.24 76.81
32.56 32.52 29.59 28.21 27.37 25.05 24.55
130.300 126.784 126.711 126.003 125.588 125.148 125.075 125.026 124.977 124.929 124.733 123.122
130 125 ppm
190 180 170 160 150 140 130 120 110 100 90 80 70 60 50 40 30 20 ppm

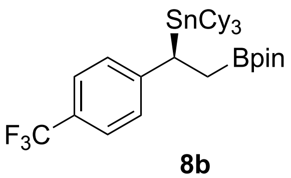

8b

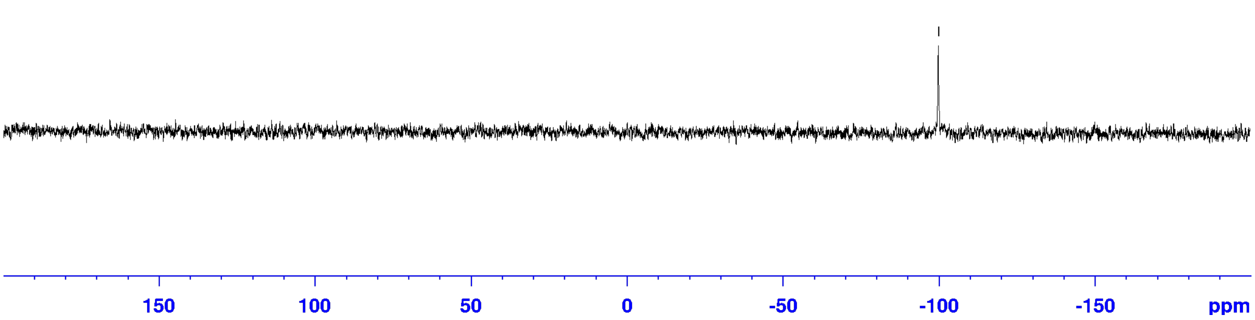

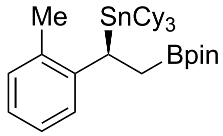
Me
SnCy3
Bpin

8c

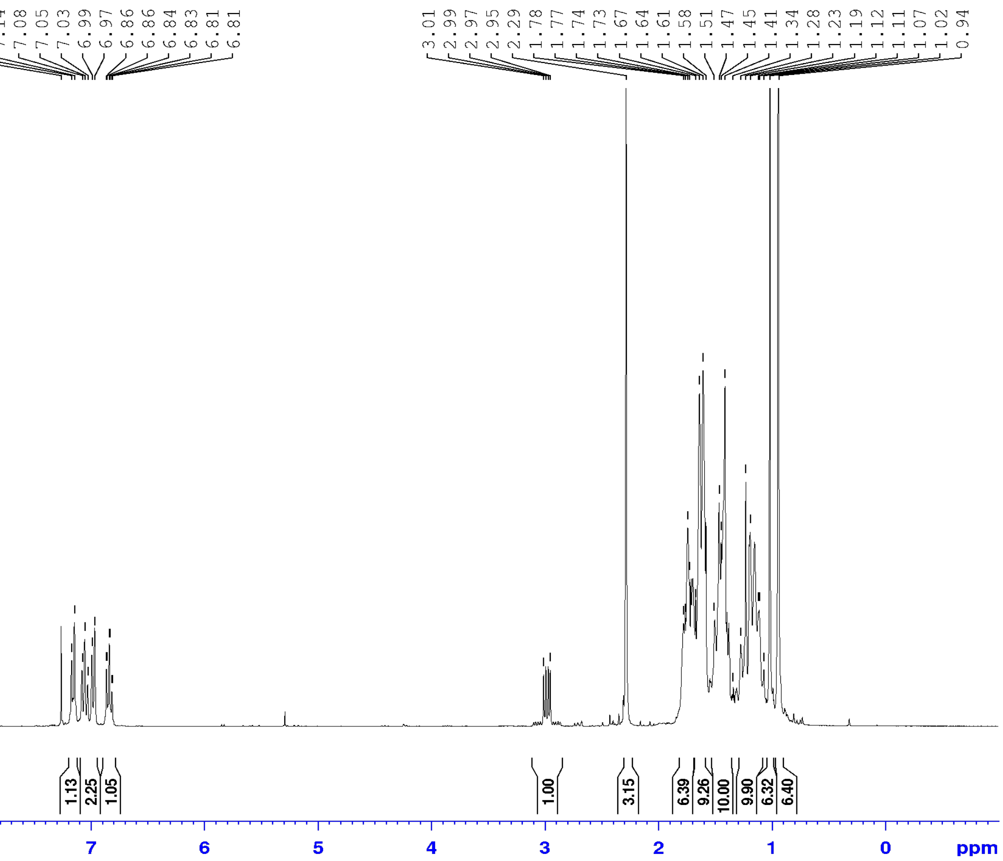
7.26 7.17 7.14 7.08 7.05 7.03 6.99 6.97 6.86 6.84 6.83 6.81 6.81
3.01 2.99 2.97 2.95 2.29 1.78 1.77 1.74 1.73 1.67 1.64 1.61 1.58 1.51 1.47 1.45 1.41 1.34 1.28 1.23 1.19 1.12 1.11 1.07 1.02 0.94
1.13 2.25 1.05 1.00 3.15 6.39 9.26 10.00 9.90 6.32 6.40
9 8 7 6 5 4 3 2 1 0 ppm

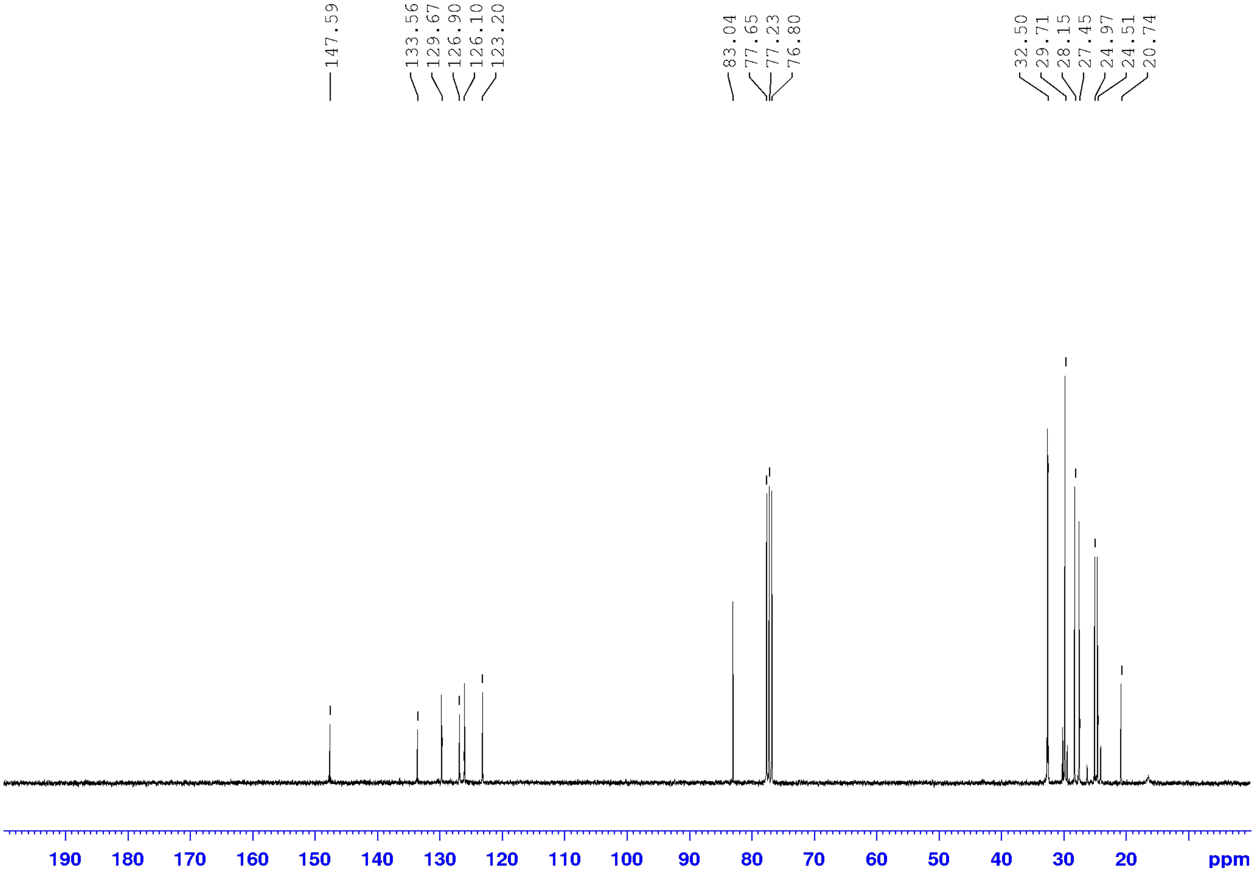
147.59
133.56 129.67 126.90 126.10 123.20
83.04 77.65 77.23 76.80
32.50 29.71 28.15 27.45 24.97 24.51 20.74
190 180 170 160 150 140 130 120 110 100 90 80 70 60 50 40 30 20 ppm

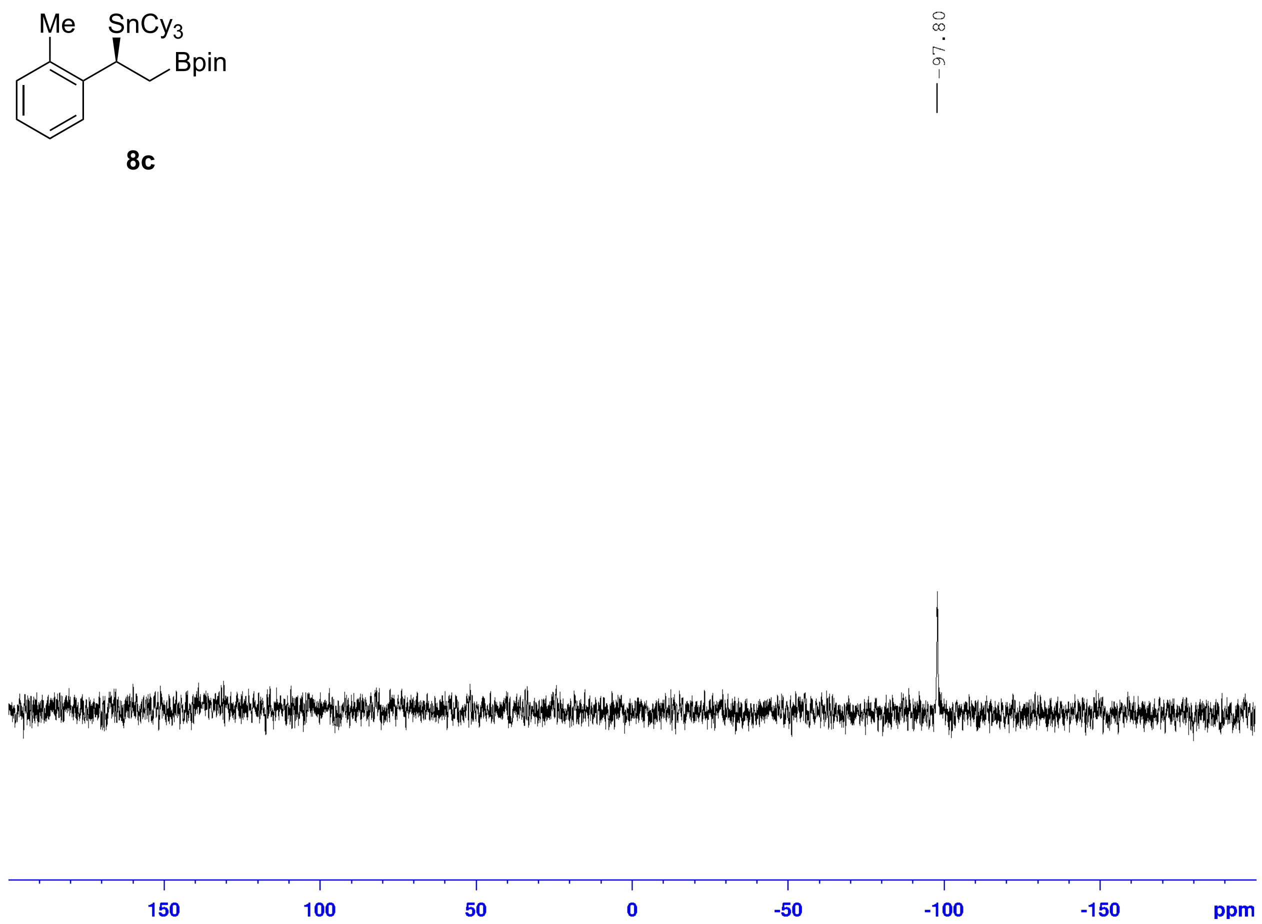

8c

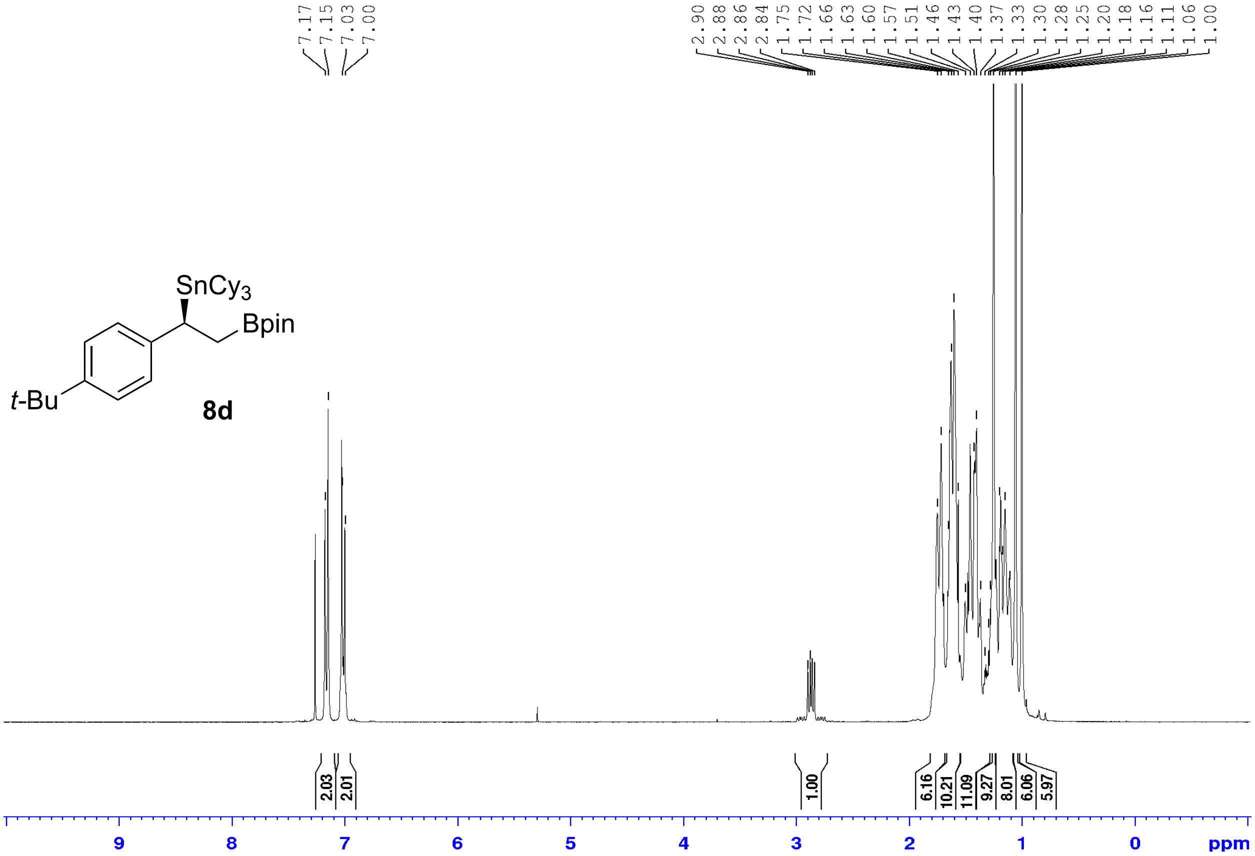
SnCy3
Bpin
t-Bu
7.17
7.15
7.03
7.00
2.90
2.88
2.86
2.84
1.75
1.72
1.66
1.63
1.60
1.57
1.51
1.46
1.43
1.40
1.37
1.33
1.30
1.28
1.25
1.20
1.18
1.16
1.11
1.06
1.00
2.03
2.01
1.00
6.16
10.21
11.09
9.27
8.01
6.06
5.97
9
8
7
6
5
4
3
2
1
0
ppm

8d

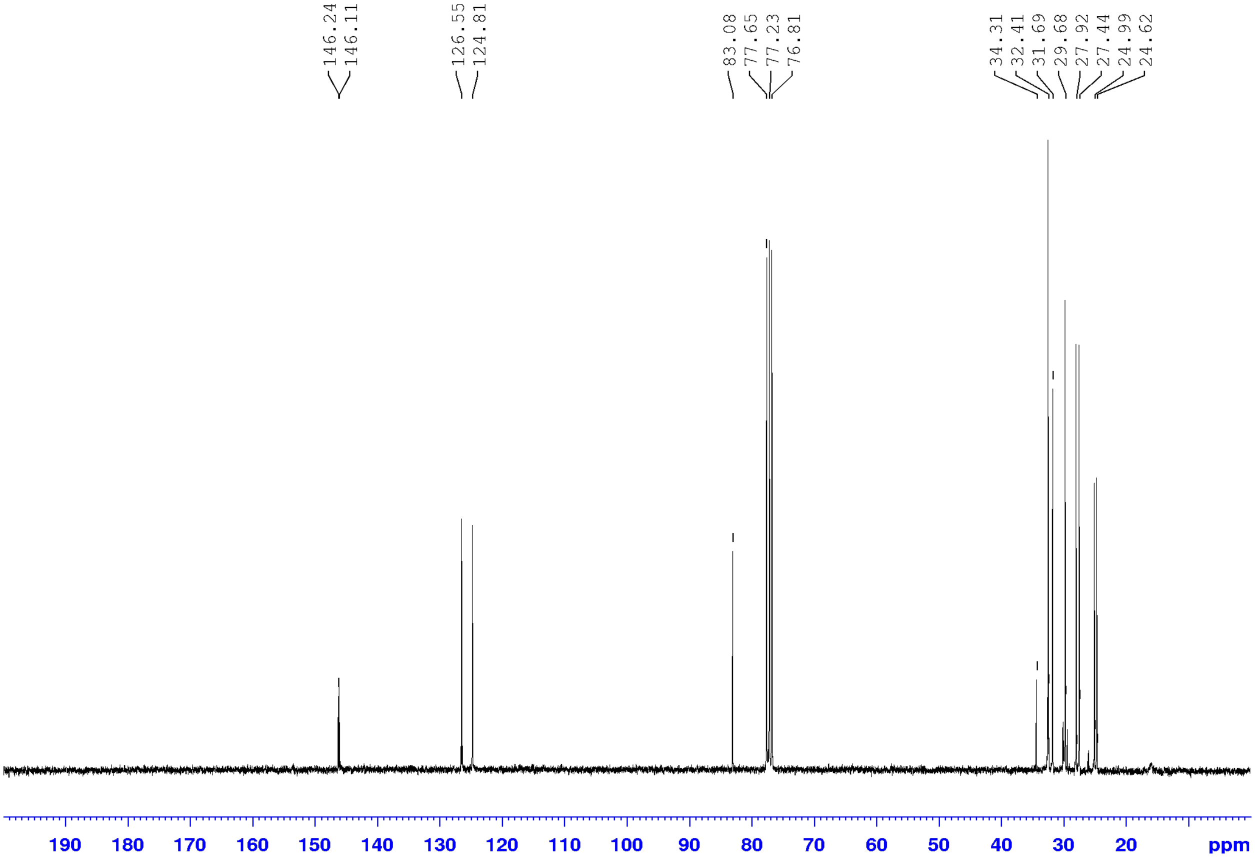
146.24
146.11
126.55
124.81
83.08
77.65
77.23
76.81
34.31
32.41
31.69
29.68
27.92
27.44
24.99
24.62
190
180
170
160
150
140
130
120
110
100
90
80
70
60
50
40
30
20
ppm

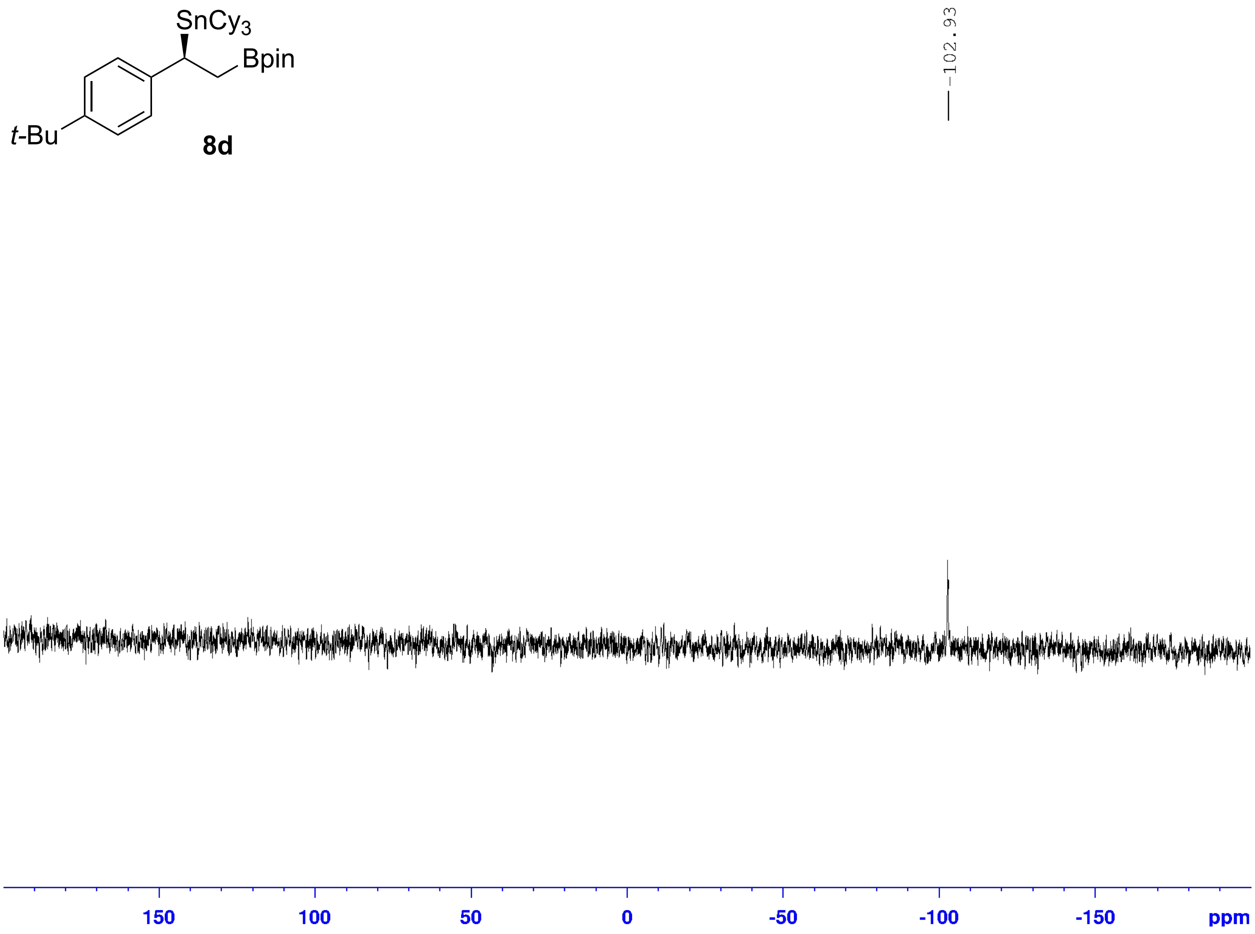
SnCy3
Bpin
t-Bu
8d
-102.93
150
100
50
0
-50
-100
-150
ppm

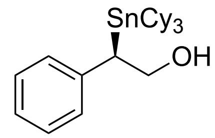
SnCy3
OH

11c

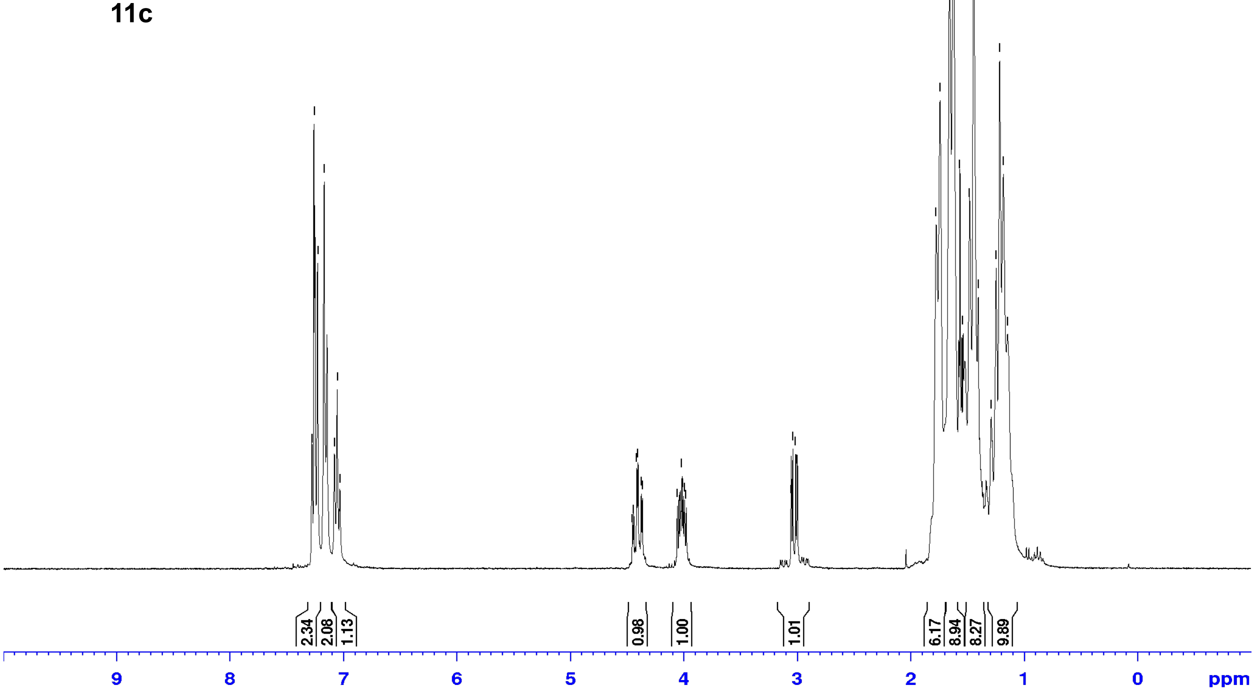
7.28 7.26 7.25 7.23 7.17 7.14 7.08 7.05 7.03
4.45 4.45 4.42 4.41 4.38 4.37 4.06 4.03 4.02 4.01 4.00 3.98 3.06 3.04 3.02 3.00 1.78 1.74 1.66 1.63 1.57 1.54 1.48 1.45 1.41 1.29 1.25 1.22 1.19 1.15
2.34 2.08 1.13 0.98 1.00 1.01 6.17 8.94 8.27 9.89
9 8 7 6 5 4 3 2 1 0 ppm

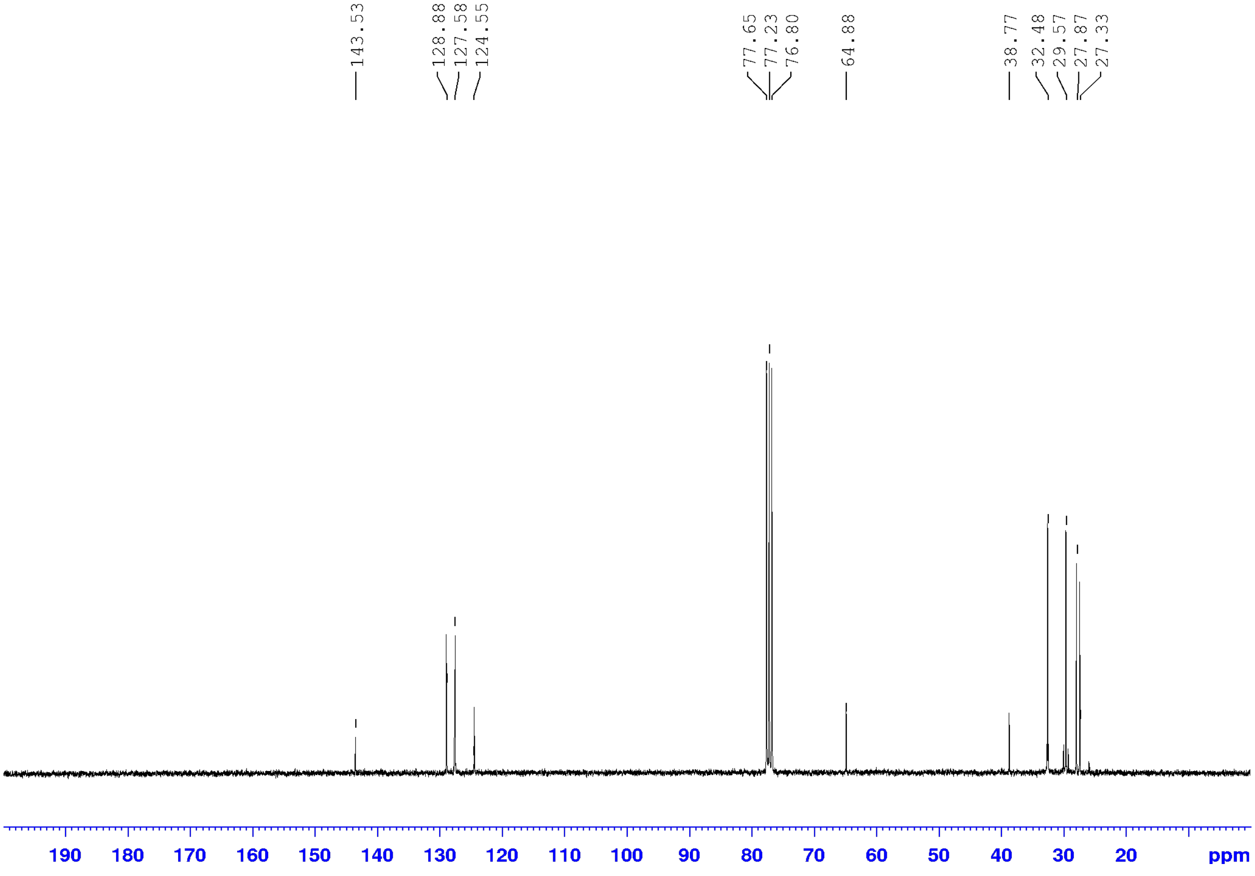
143.53
128.88 127.58 124.55
77.65 77.23 76.80
64.88
38.77 32.48 29.57 27.87 27.33
190 180 170 160 150 140 130 120 110 100 90 80 70 60 50 40 30 20 ppm

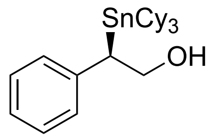

11c

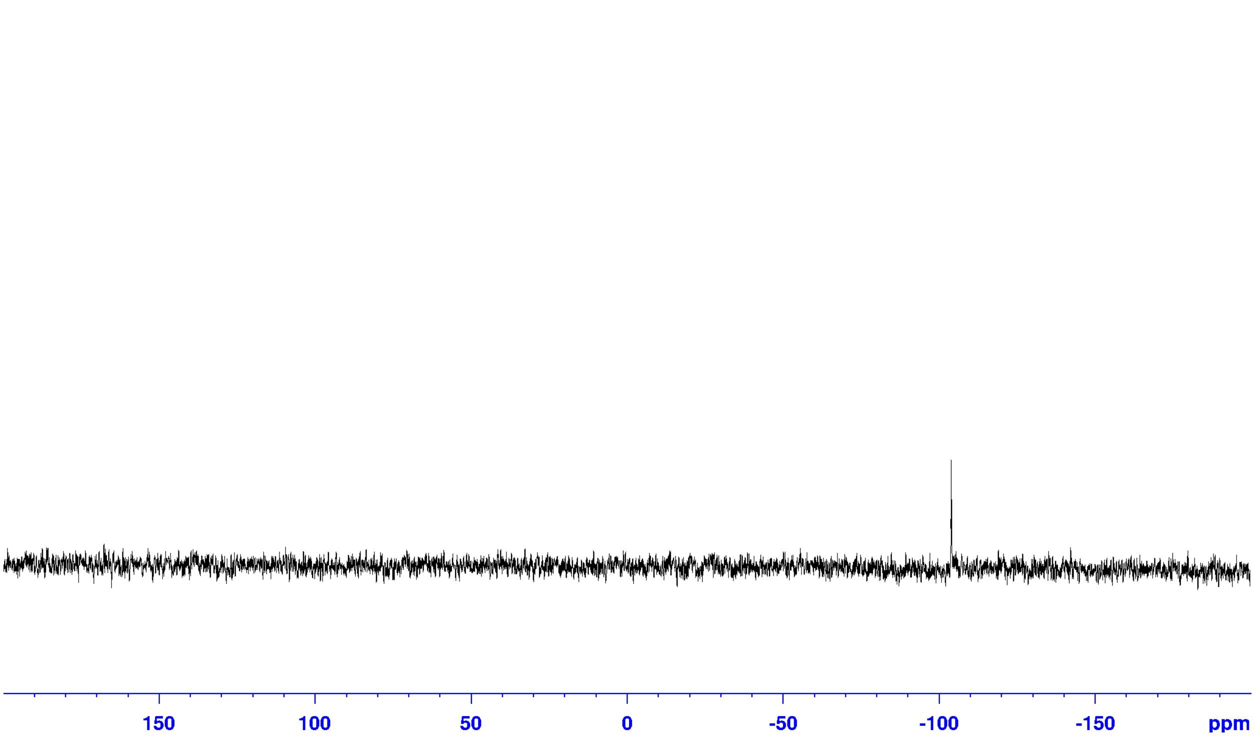

S6

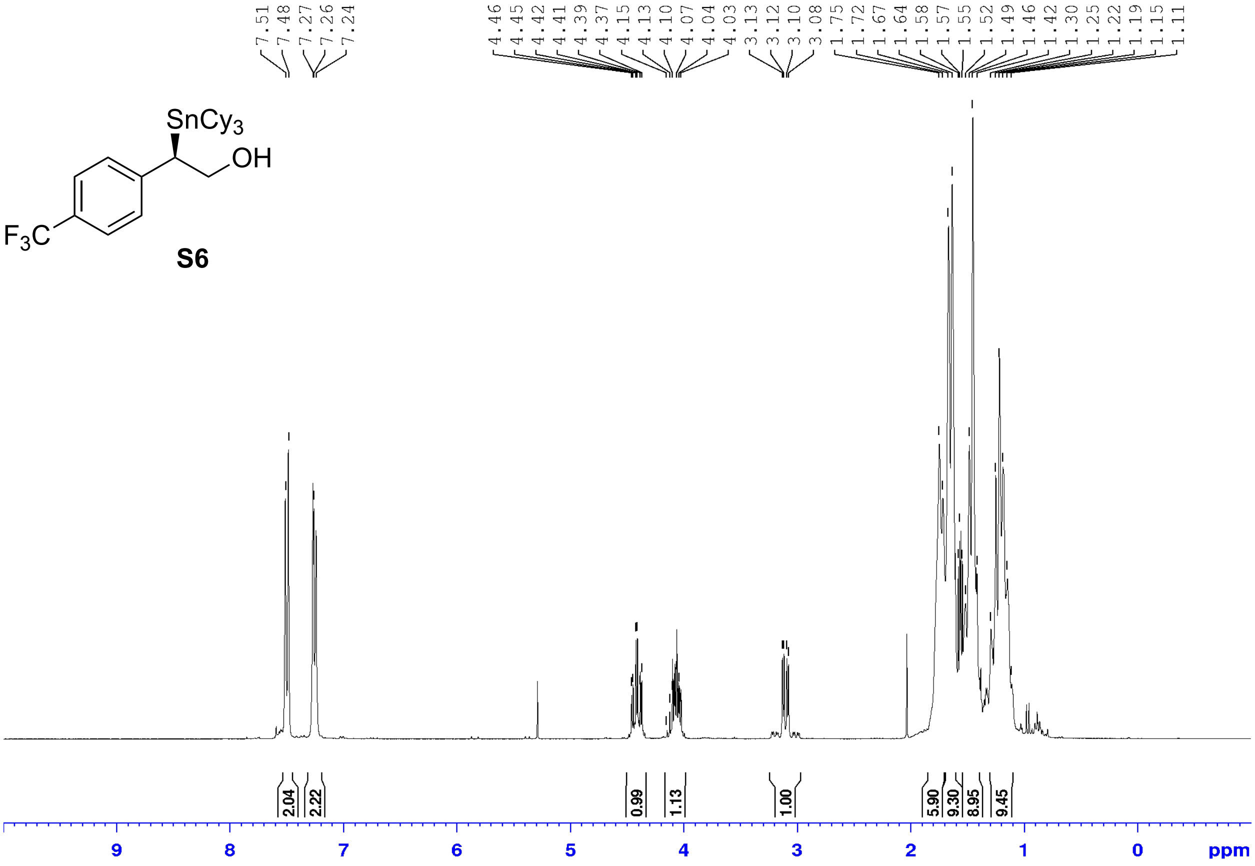
SnCy3
OH
F3C
7.51
7.48
7.27
7.26
7.24
4.46
4.45
4.42
4.41
4.39
4.37
4.15
4.13
4.10
4.07
4.04
4.03
3.13
3.12
3.10
3.08
1.75
1.72
1.67
1.64
1.58
1.57
1.55
1.52
1.49
1.46
1.42
1.30
1.25
1.22
1.19
1.15
1.11
2.04
2.22
0.99
1.13
1.00
5.90
9.30
8.95
9.45
9
8
7
6
5
4
3
2
1
0
ppm

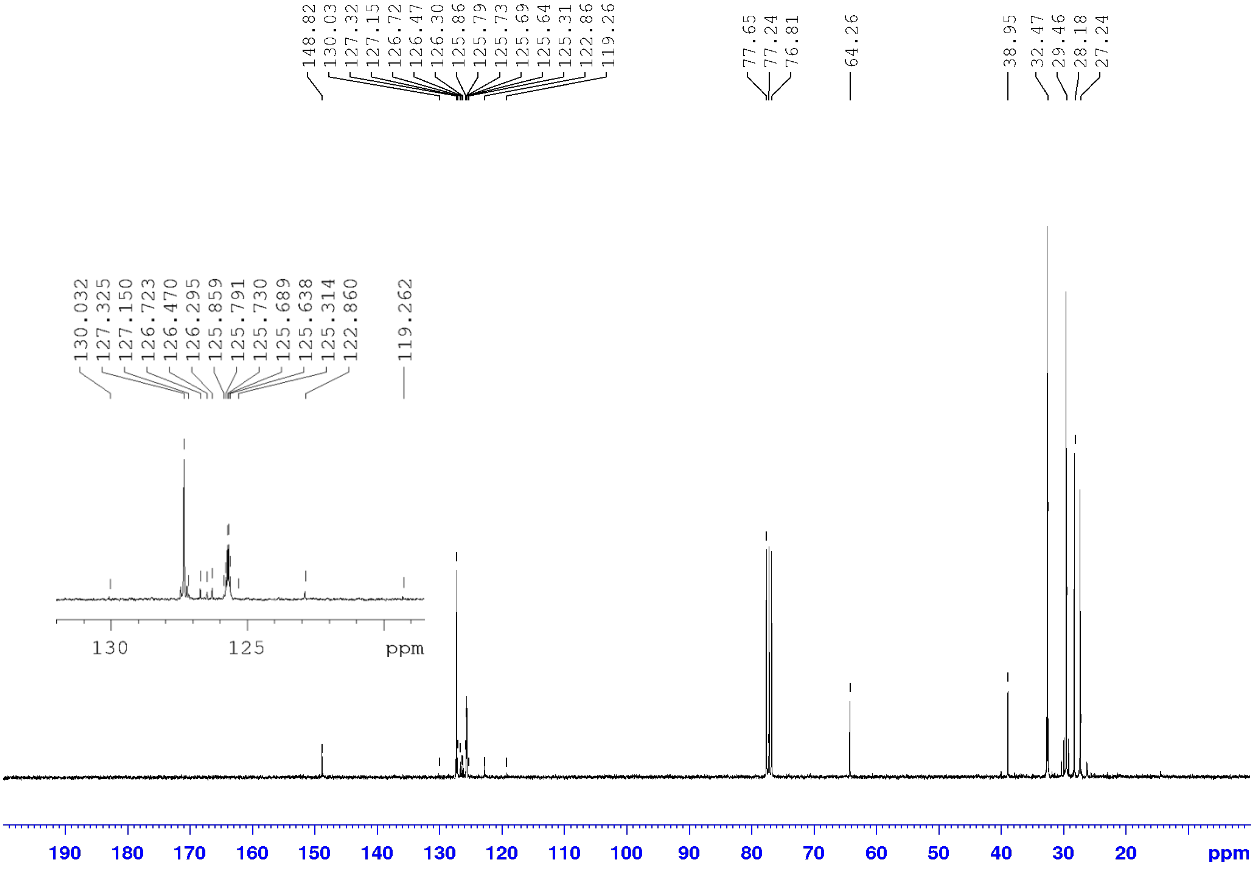
148.82
130.03
127.32
127.15
126.72
126.47
126.30
125.86
125.79
125.73
125.69
125.64
125.31
122.86
119.26
77.65
77.24
76.81
64.26
38.95
32.47
29.46
28.18
27.24
130.032
127.325
127.150
126.723
126.470
126.295
125.859
125.791
125.730
125.689
125.638
125.314
122.860
119.262
130
125
ppm
190
180
170
160
150
140
130
120
110
100
90
80
70
60
50
40
30
20
ppm

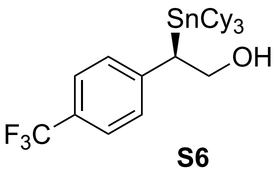

S6

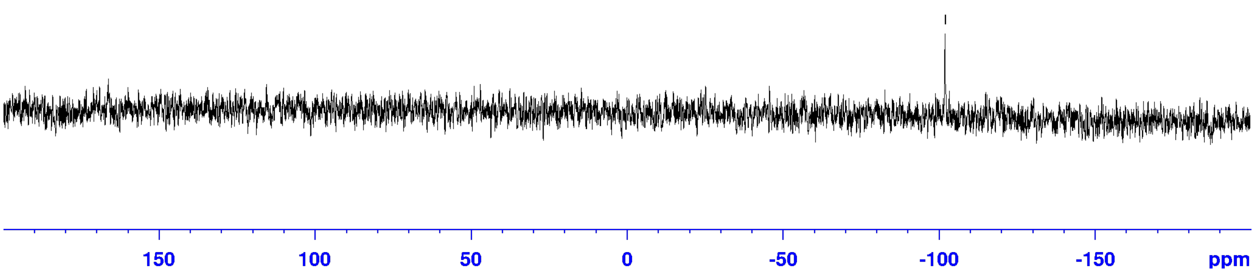

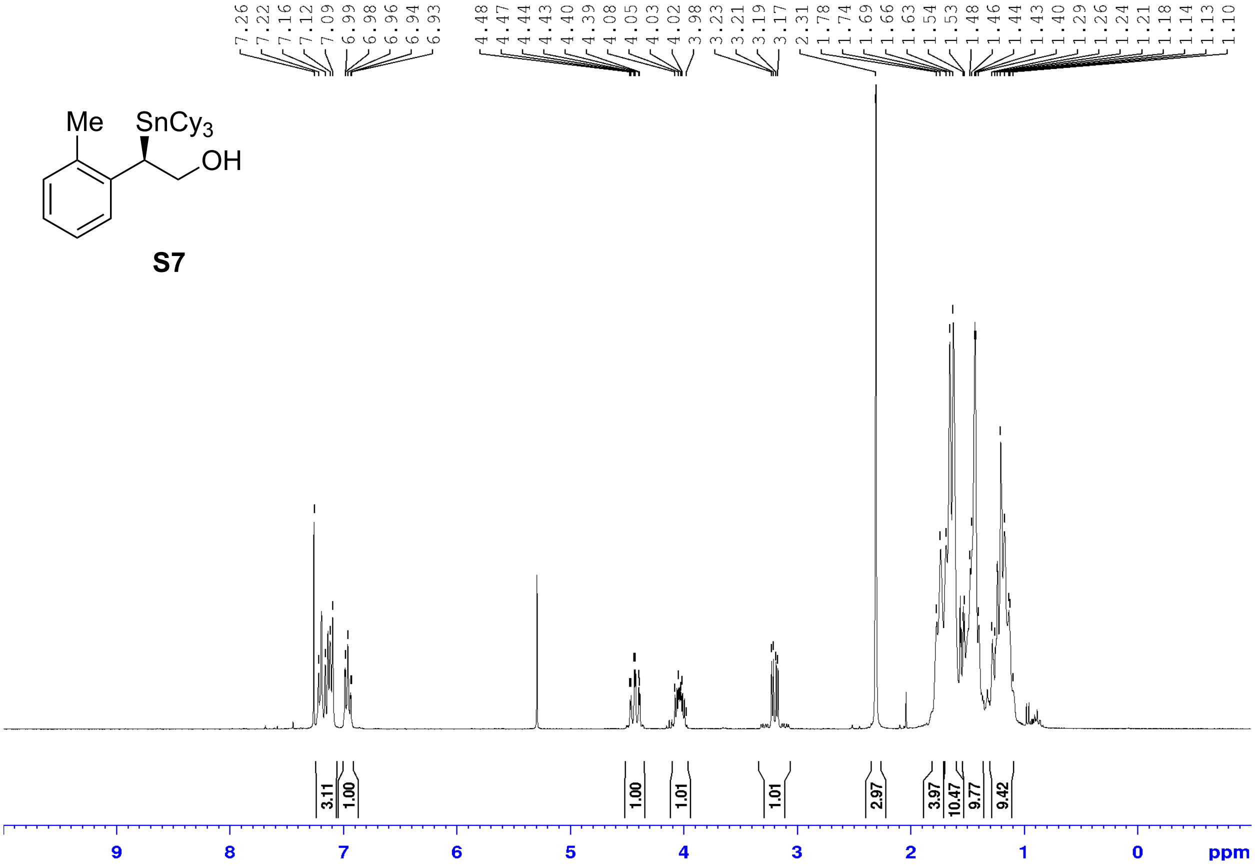

Me
SnCy3
OH
S7
7.26 7.22 7.16 7.12 7.09 6.99 6.98 6.96 6.94 6.93
4.48 4.47 4.44 4.43 4.40 4.39 4.08 4.05 4.03 4.02 3.98 3.23 3.21 3.19 3.17
2.31 1.78 1.74 1.69 1.66 1.63 1.54 1.53 1.48 1.46 1.44 1.43 1.40 1.29 1.26 1.24 1.21 1.18 1.14 1.13 1.10
3.11 1.00 1.00 1.01 1.01 2.97 3.97 10.47 9.77 9.42
9 8 7 6 5 4 3 2 1 0 ppm

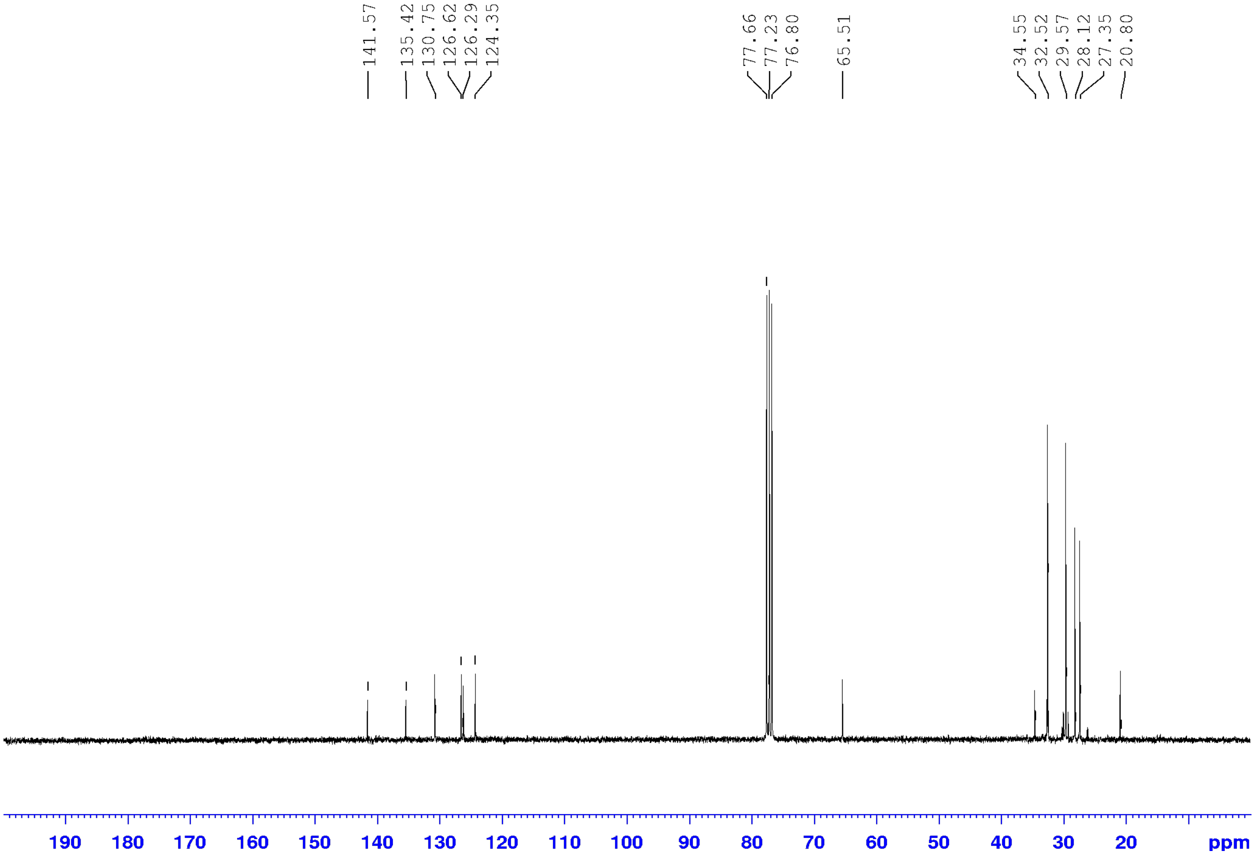

141.57 135.42 130.75 126.62 126.29 124.35
77.66 77.23 76.80 65.51
34.55 32.52 29.57 28.12 27.35 20.80
190 180 170 160 150 140 130 120 110 100 90 80 70 60 50 40 30 20 ppm

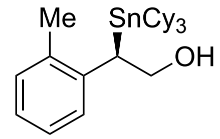

S7

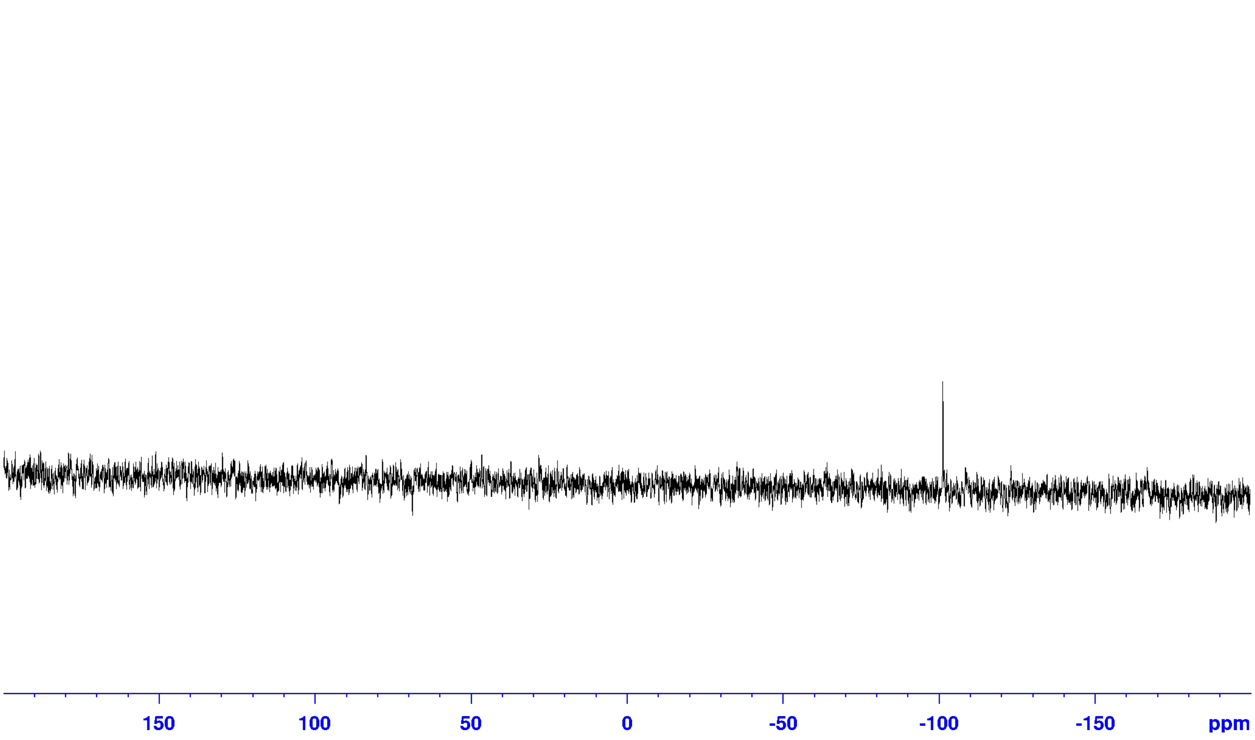

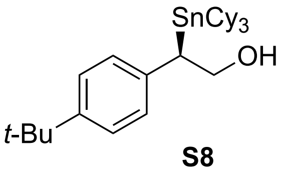

S8

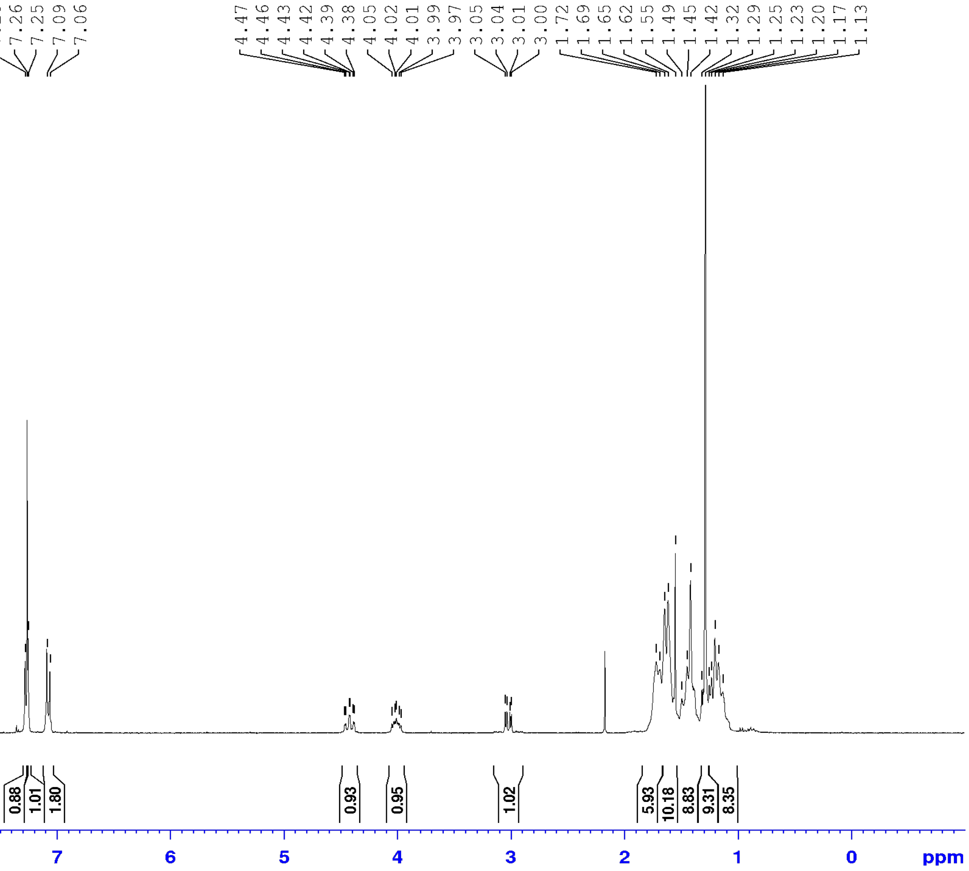

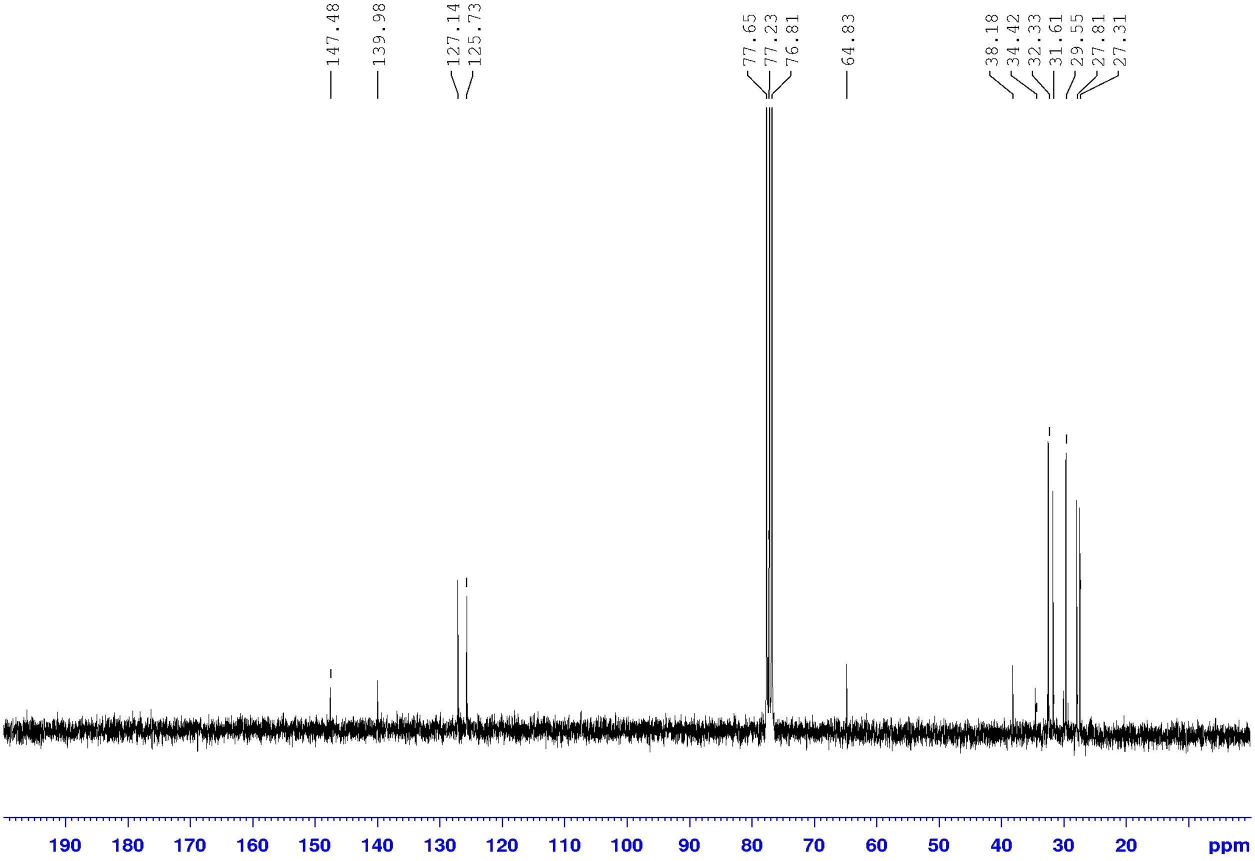

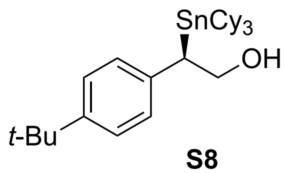

S8

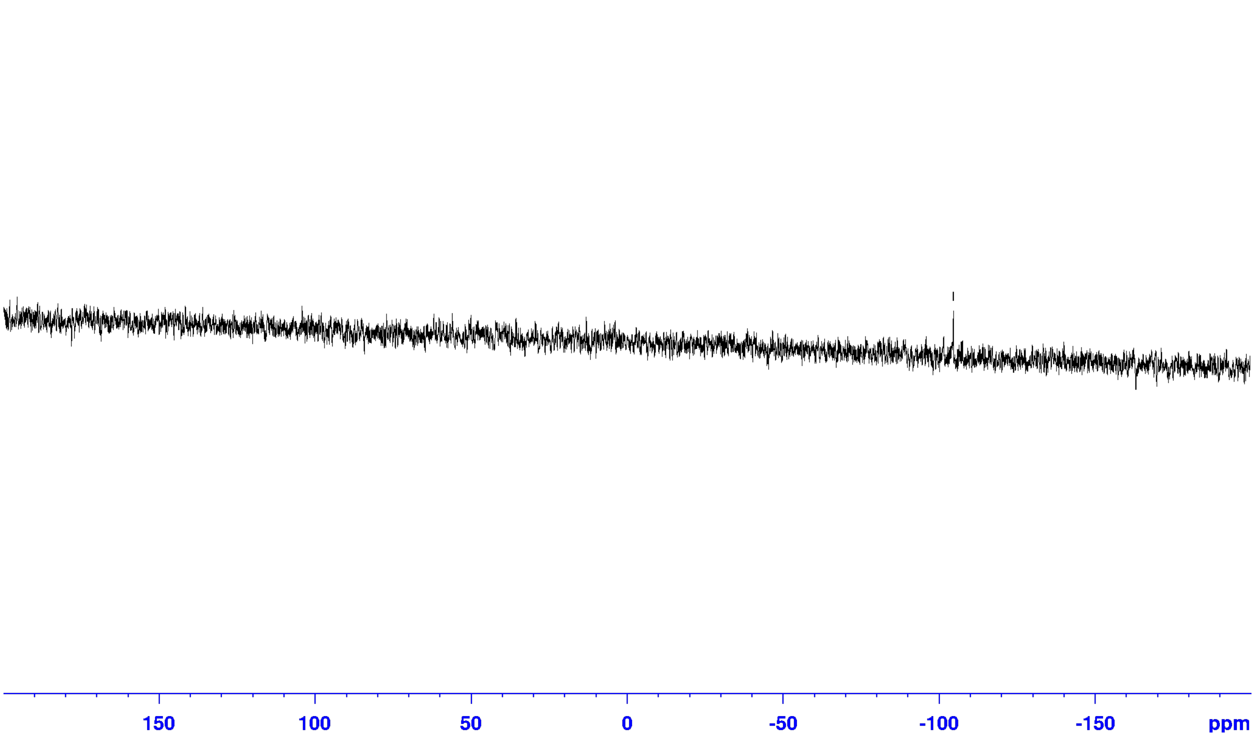

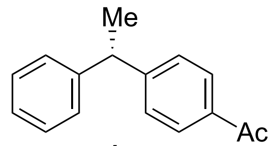
Me
Ac

4a

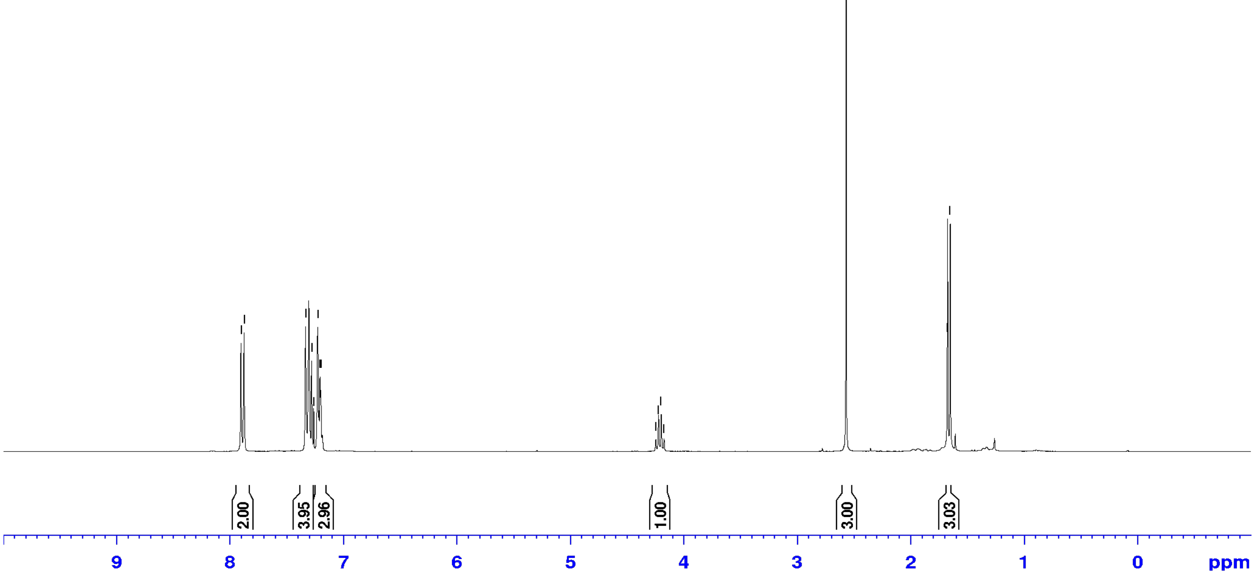
7.90
7.87
7.33
7.30
7.28
7.26
7.22
7.21
7.20
4.25
4.23
4.20
4.18
2.57
1.68
1.66
2.00
3.95
2.96
1.00
3.00
3.03
9
8
7
6
5
4
3
2
1
0
ppm

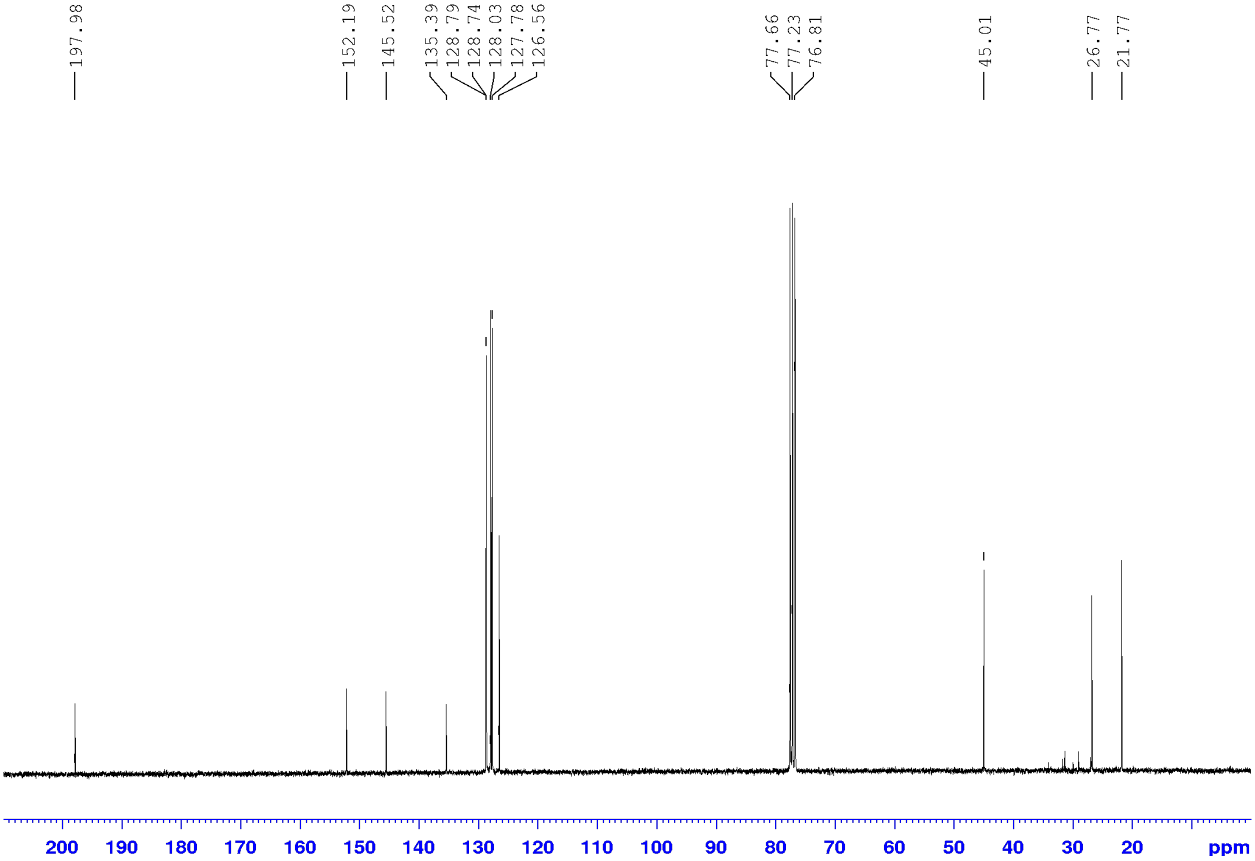
197.98
152.19
145.52
135.39
128.79
128.74
128.03
127.78
126.56
77.66
77.23
76.81
45.01
26.77
21.77
200
190
180
170
160
150
140
130
120
110
100
90
80
70
60
50
40
30
20
ppm

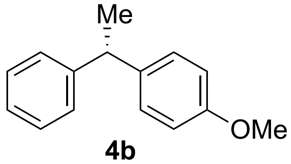
Me
OMe

4b

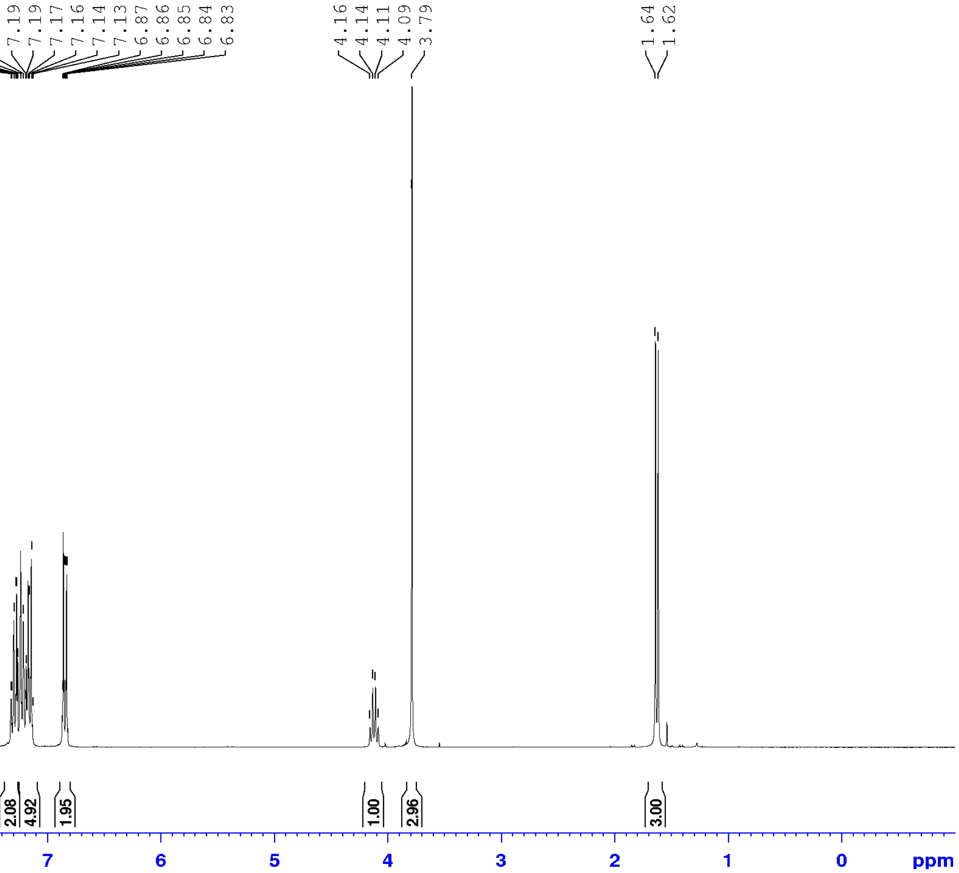
7.32
7.31
7.29
7.28
7.27
7.26
7.23
7.21
7.19
7.19
7.17
7.16
7.14
7.13
6.87
6.86
6.85
6.84
6.83
4.16
4.14
4.11
4.09
3.79
1.64
1.62
2.08
4.92
1.95
1.00
2.96
3.00
9
8
7
6
5
4
3
2
1
0
ppm

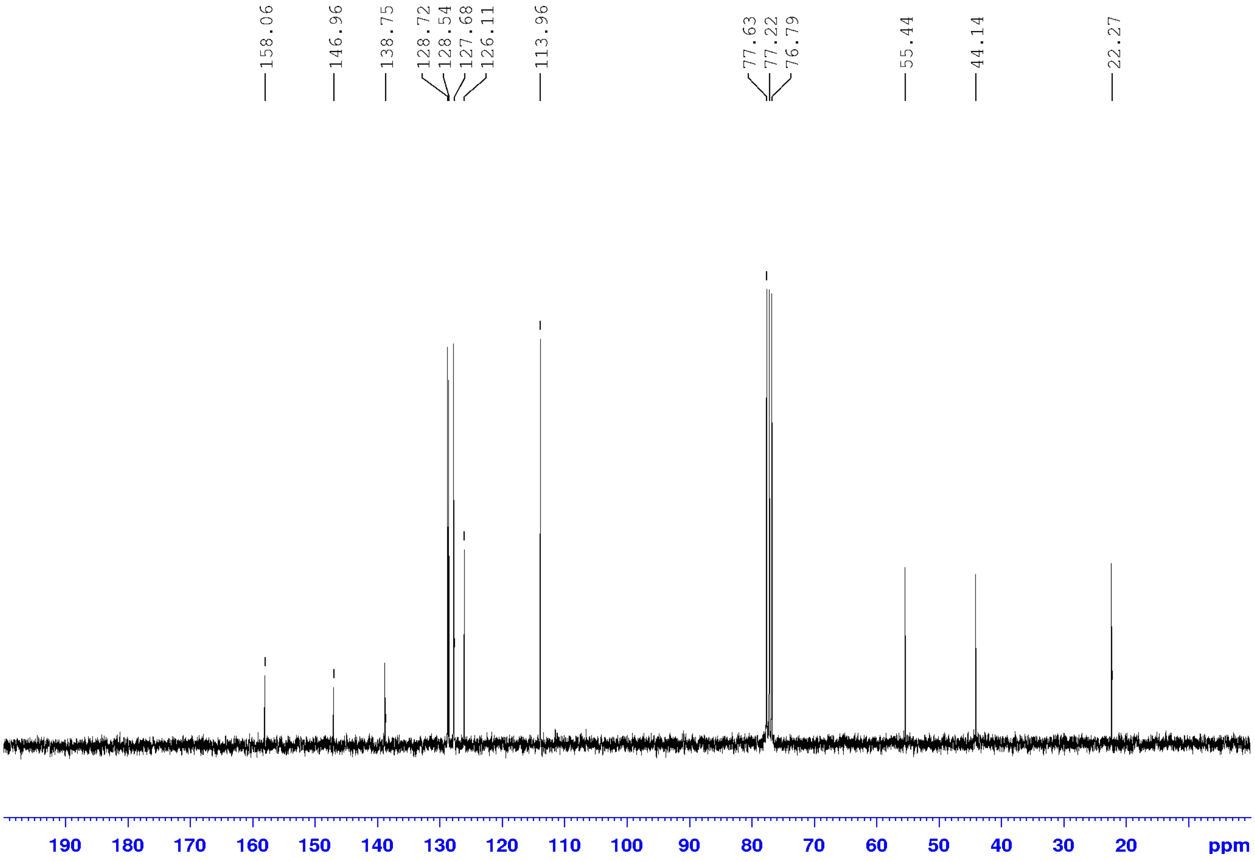
158.06
146.96
138.75
128.72
128.54
127.68
126.11
113.96
77.63
77.22
76.79
55.44
44.14
22.27
190
180
170
160
150
140
130
120
110
100
90
80
70
60
50
40
30
20
ppm

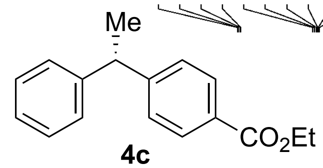
Me
CO_2Et

4c

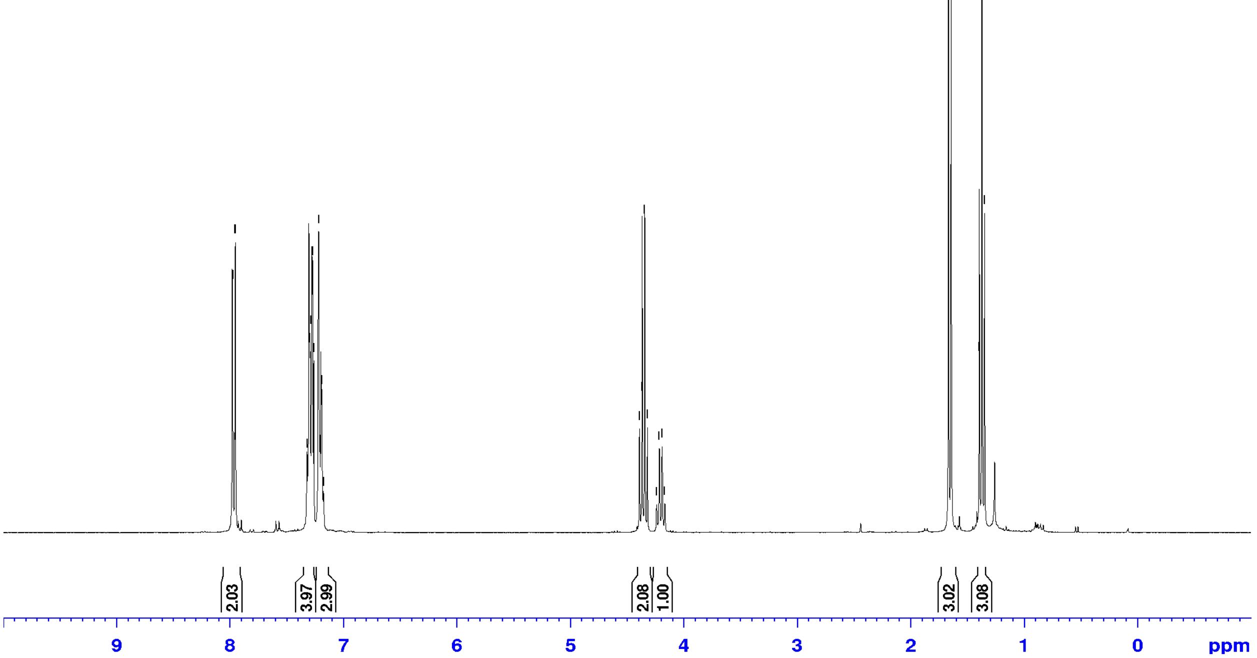
7.98 7.97 7.96 7.95 7.32 7.30 7.29 7.28 7.27 7.26 7.22 7.20 7.19 7.18
4.40 4.37 4.35 4.32 4.24 4.22 4.19 4.17
1.67 1.65 1.40 1.38 1.35
2.03 3.97 2.99 2.08 1.00 3.02 3.08
9 8 7 6 5 4 3 2 1 0 ppm

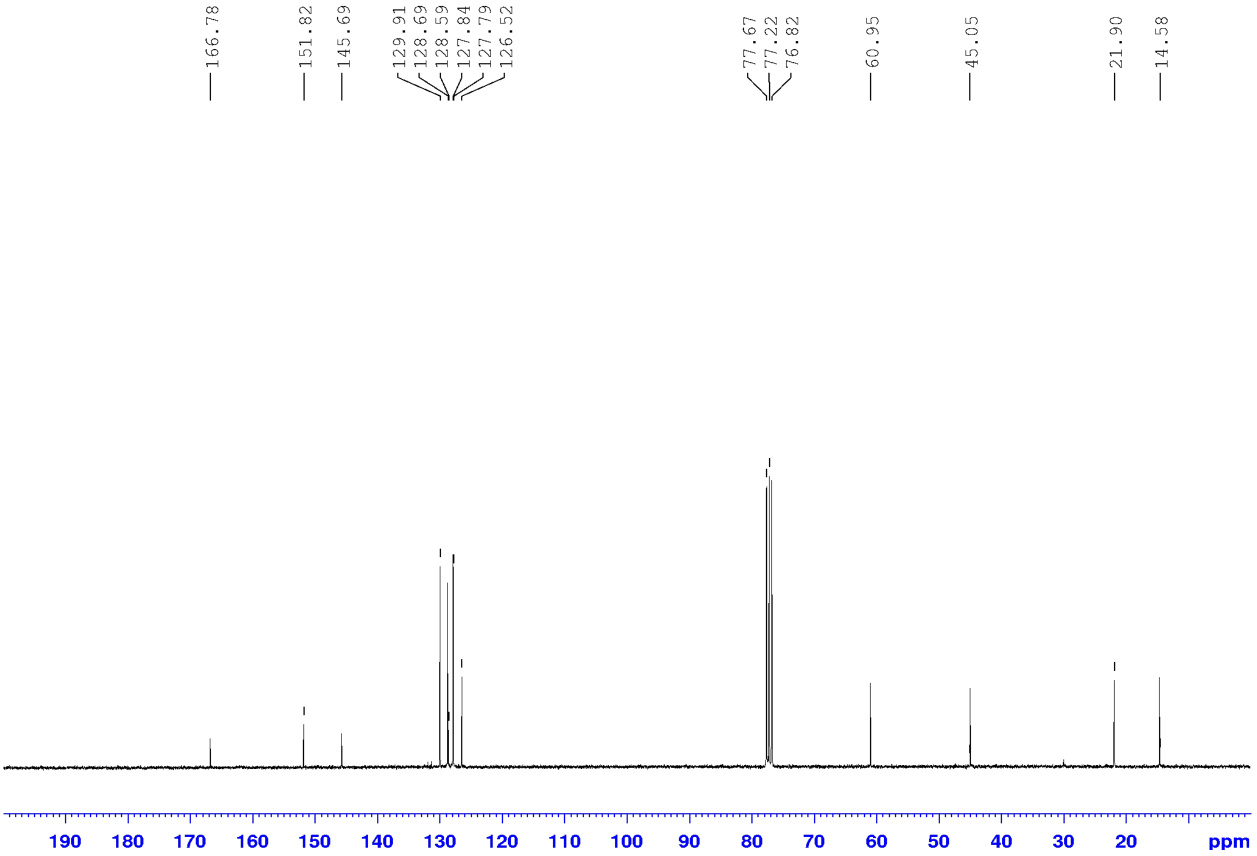
166.78
151.82
145.69
129.91 128.69 128.59 127.84 127.79 126.52
77.67 77.22 76.82
60.95
45.05
21.90
14.58
190 180 170 160 150 140 130 120 110 100 90 80 70 60 50 40 30 20 ppm

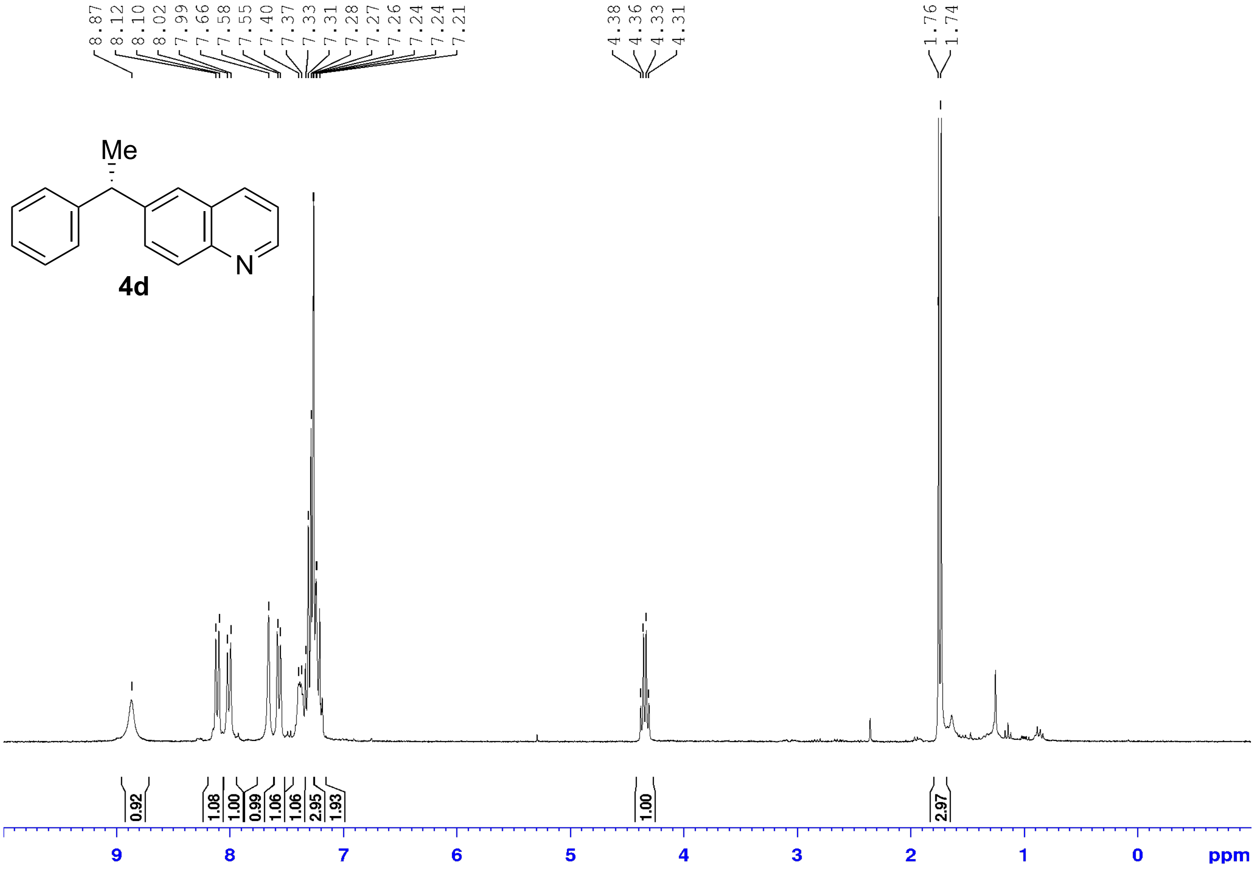
8.87
8.12
8.10
8.02
7.99
7.66
7.58
7.55
7.40
7.37
7.33
7.31
7.28
7.27
7.26
7.24
7.24
7.21
4.38
4.36
4.33
4.31
1.76
1.74
Me
N
4d
0.92
1.08
1.00
0.99
1.06
1.06
2.95
1.93
1.00
2.97
9
8
7
6
5
4
3
2
1
0
ppm

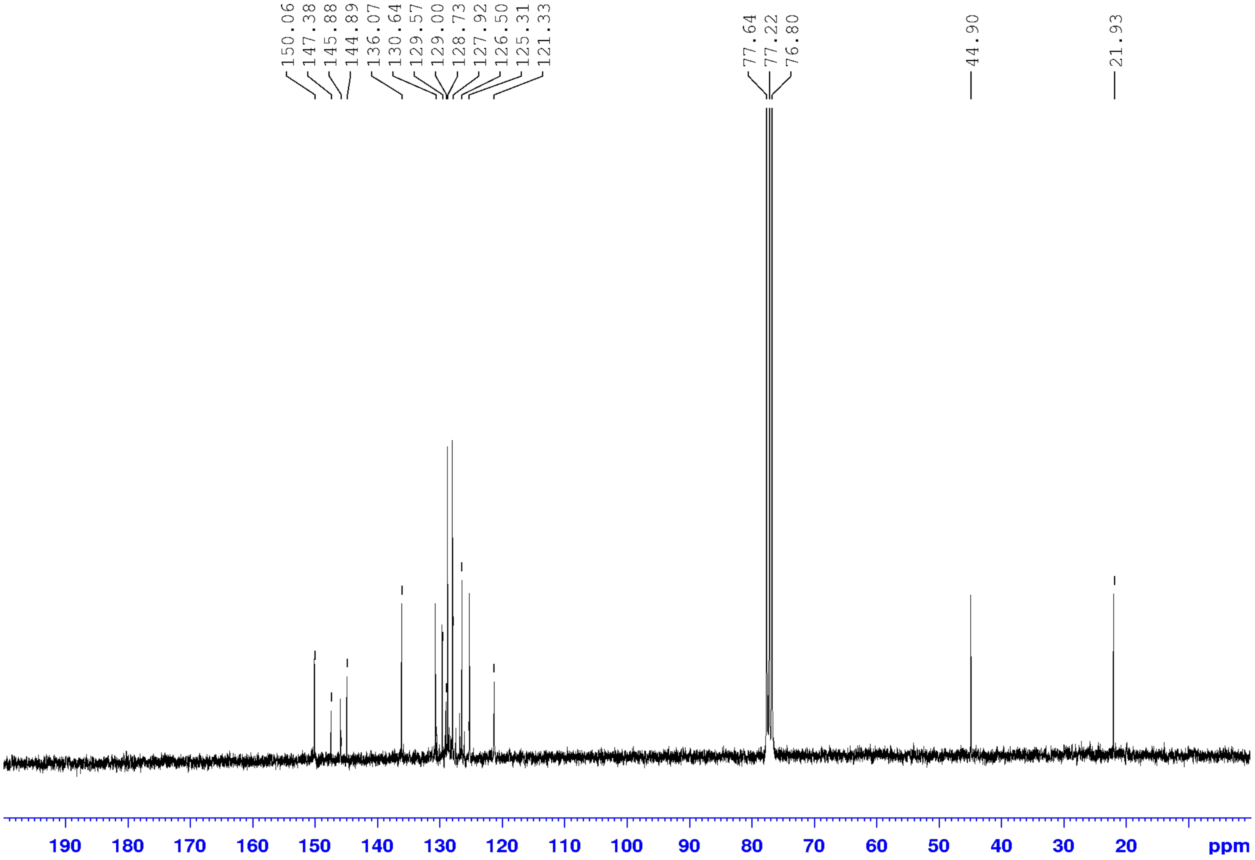
150.06
147.38
145.88
144.89
136.07
130.64
129.57
129.00
128.73
127.92
126.50
125.31
121.33
77.64
77.22
76.80
44.90
21.93
190
180
170
160
150
140
130
120
110
100
90
80
70
60
50
40
30
20
ppm

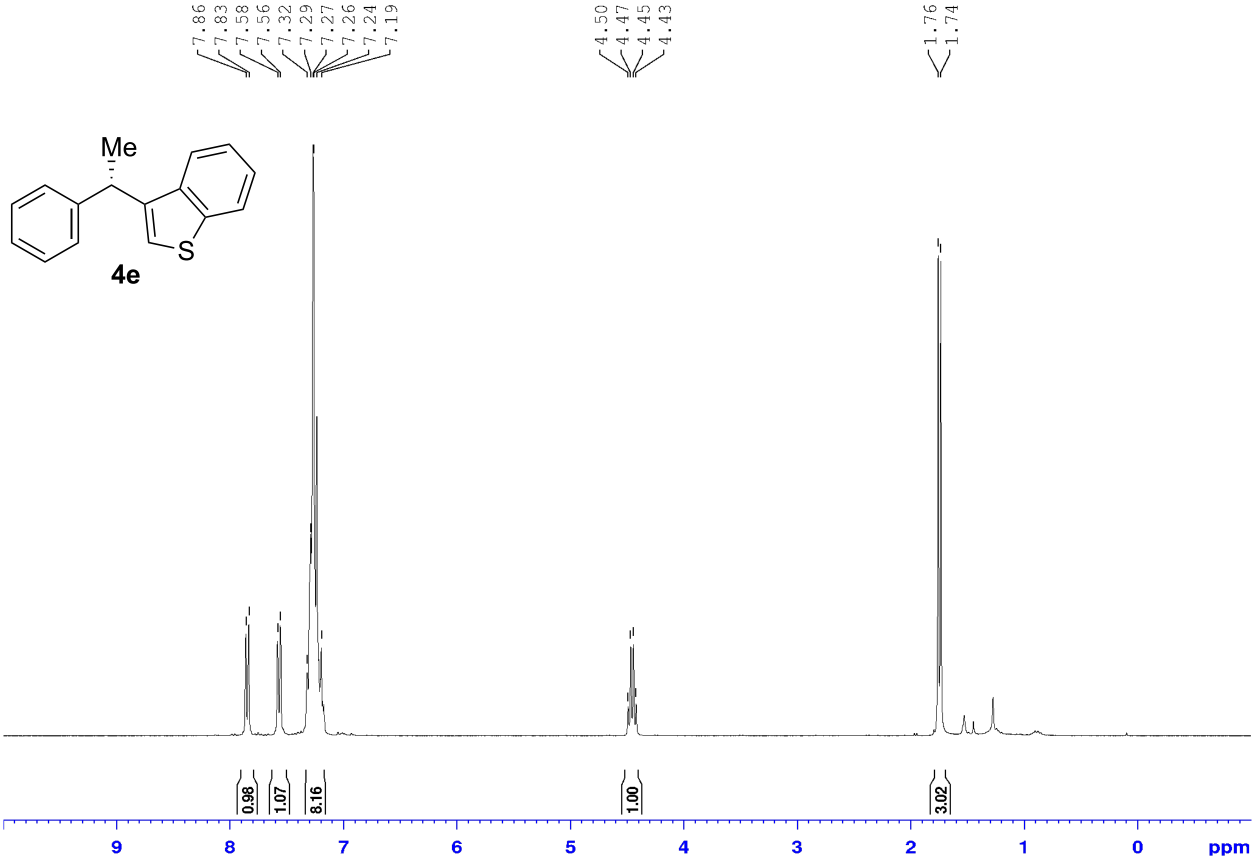
Me
S
7.86
7.83
7.58
7.56
7.32
7.29
7.27
7.26
7.24
7.19
4.50
4.47
4.45
4.43
1.76
1.74
0.98
1.07
8.16
1.00
3.02
9
8
7
6
5
4
3
2
1
0
ppm

4e

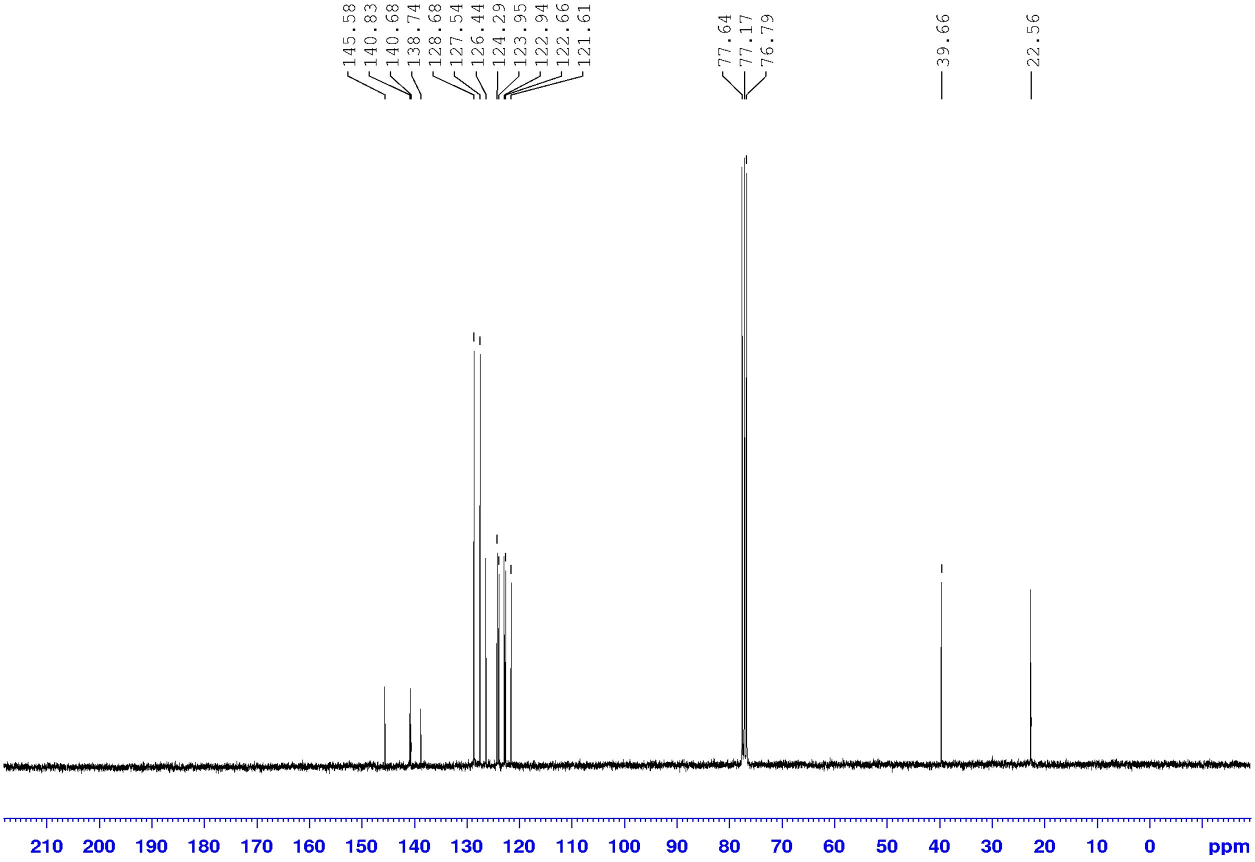
145.58
140.83
140.68
138.74
128.68
127.54
126.44
124.29
123.95
122.94
122.66
121.61
77.64
77.17
76.79
39.66
22.56
210
200
190
180
170
160
150
140
130
120
110
100
90
80
70
60
50
40
30
20
10
0
ppm

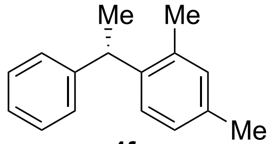

Me
Me
Me

4f

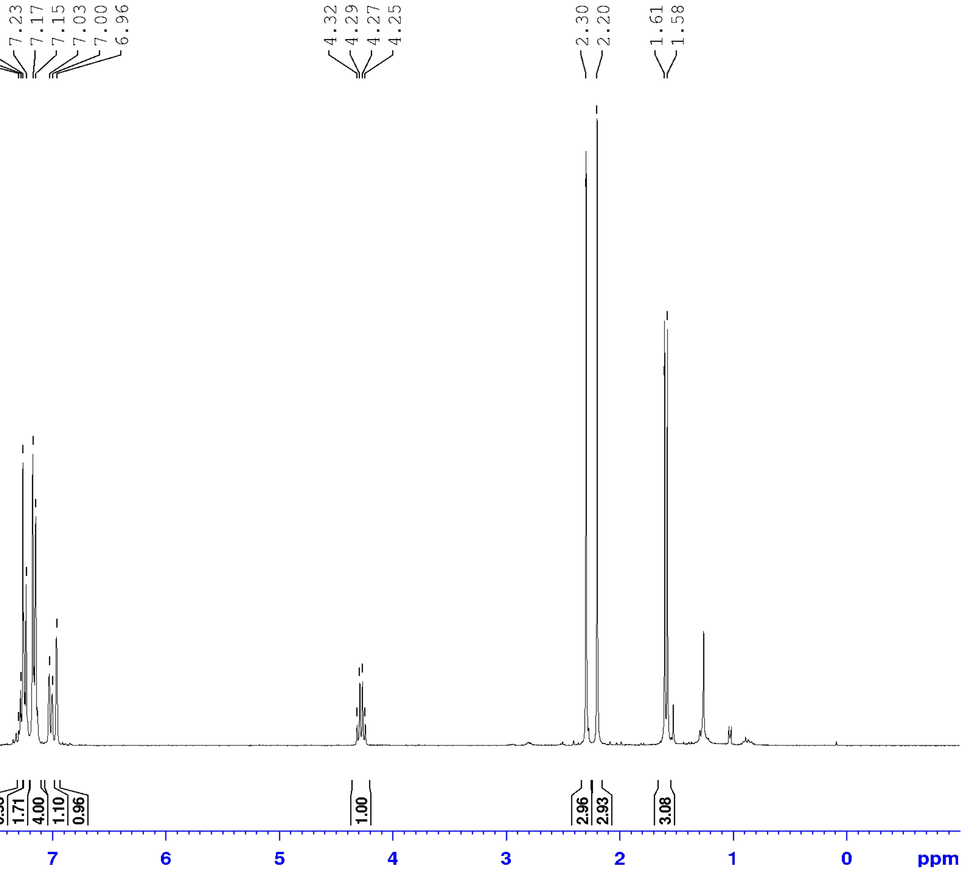

7.30 7.28 7.26 7.23 7.17 7.15 7.03 7.00 6.96
4.32 4.29 4.27 4.25
2.30 2.20
1.61 1.58
0.58 1.71 4.00 1.10 0.96
1.00
2.96 2.93
3.08
9 8 7 6 5 4 3 2 1 0 ppm

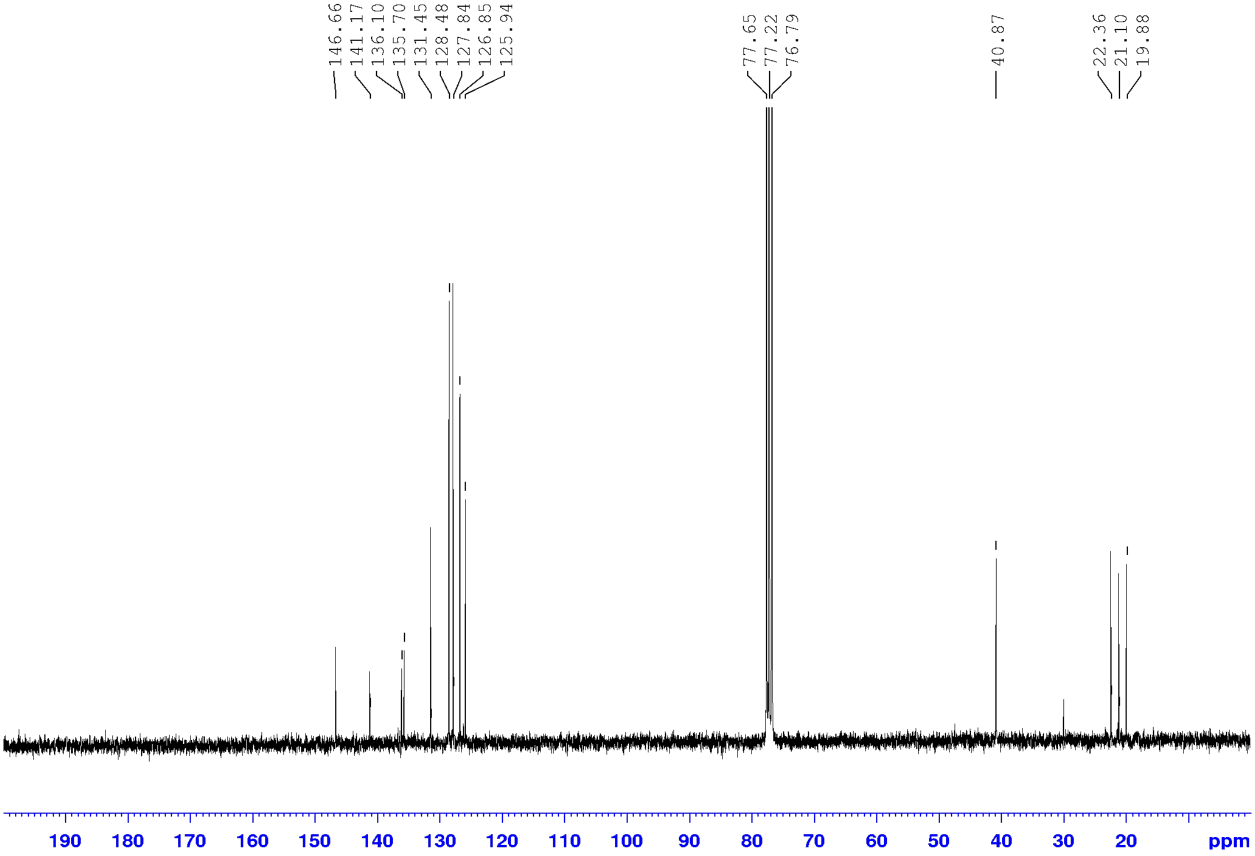

146.66 141.17 136.10 135.70 131.45 128.48 127.84 126.85 125.94
77.65 77.22 76.79
40.87
22.36 21.10 19.88
190 180 170 160 150 140 130 120 110 100 90 80 70 60 50 40 30 20 ppm

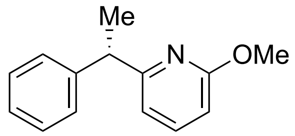
Me
N
OMe

4g

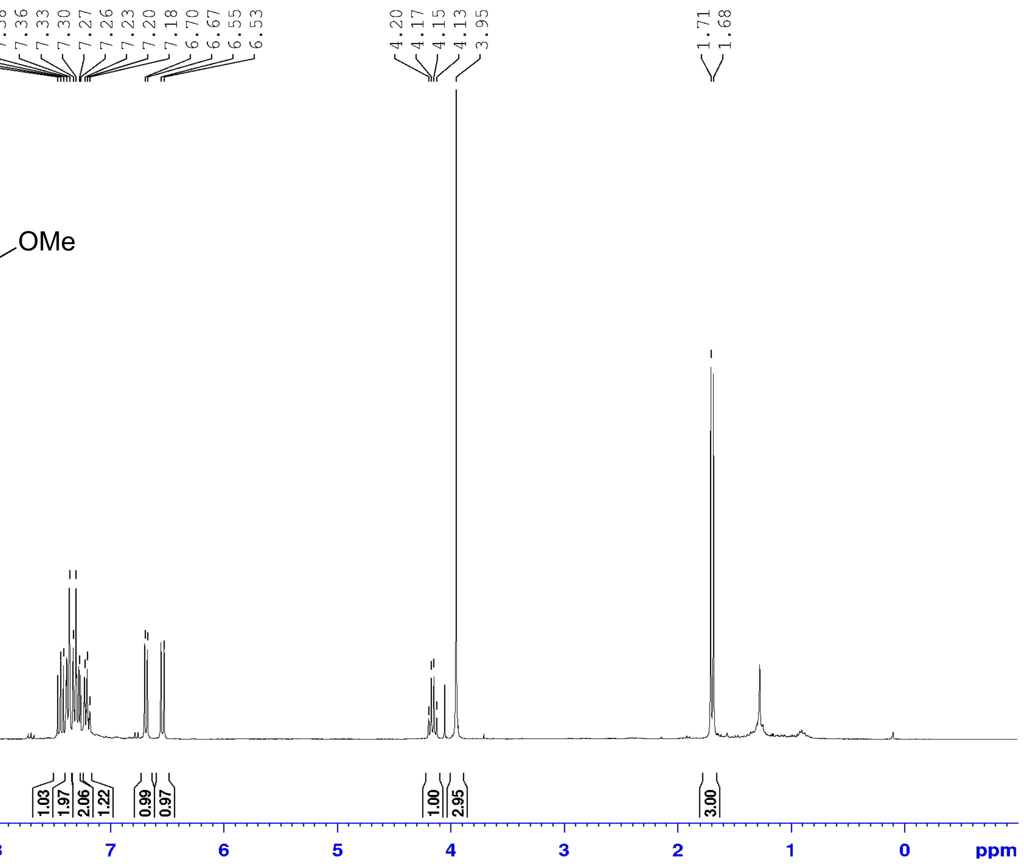
7.46 7.44 7.41 7.38 7.36 7.33 7.30 7.27 7.26 7.23 7.20 7.18 6.70 6.67 6.55 6.53
4.20 4.17 4.15 4.13 3.95
1.71 1.68
1.03 1.97 2.06 1.22 0.99 0.97 1.00 2.95 3.00
9 8 7 6 5 4 3 2 1 0 ppm

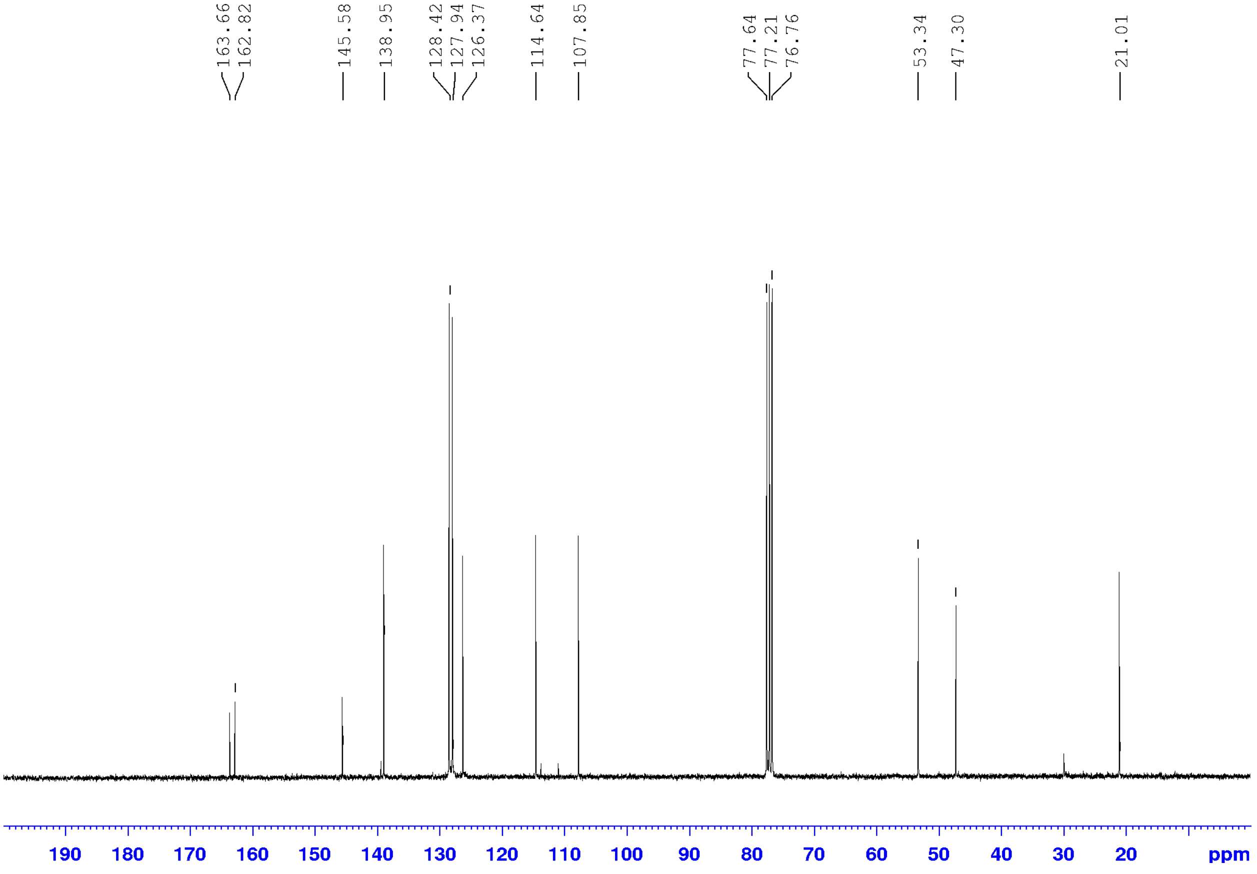
163.66 162.82 145.58 138.95 128.42 127.94 126.37 114.64 107.85 77.64 77.21 76.76 53.34 47.30 21.01
190 180 170 160 150 140 130 120 110 100 90 80 70 60 50 40 30 20 ppm

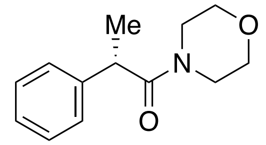
Me
N
O
O

6a

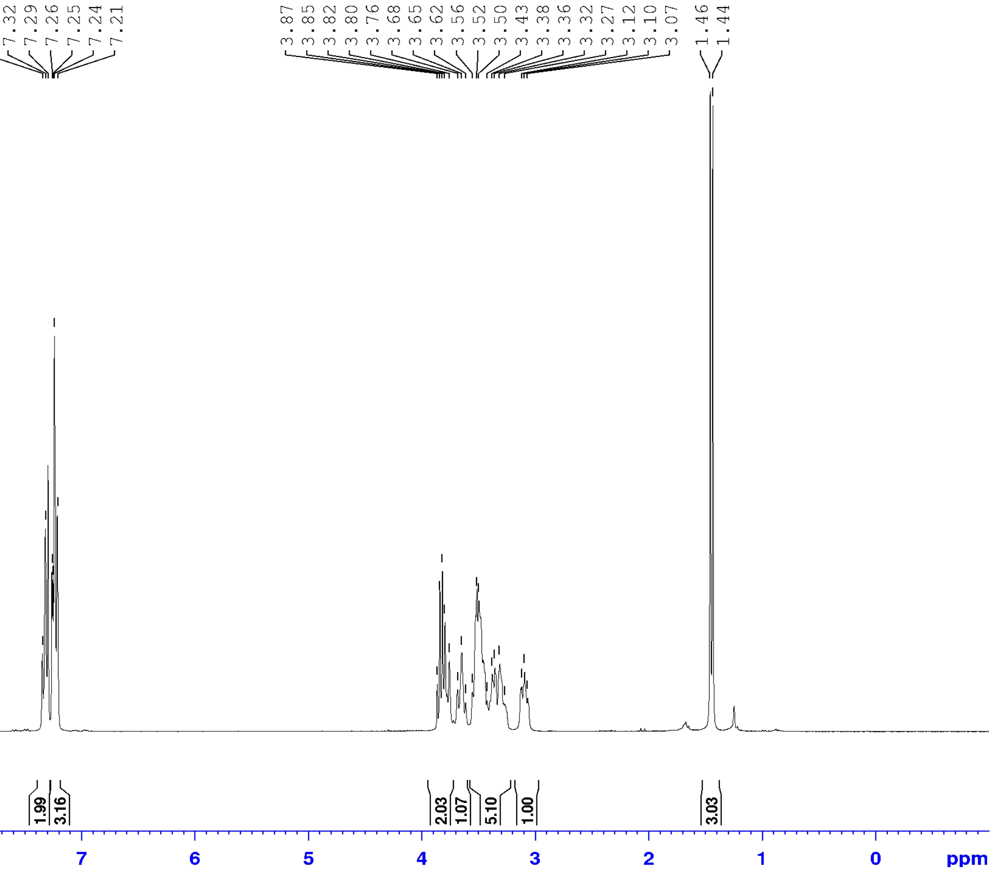
7.34
7.32
7.29
7.26
7.25
7.24
7.21
3.87
3.85
3.82
3.80
3.76
3.68
3.65
3.62
3.56
3.52
3.50
3.43
3.38
3.36
3.32
3.27
3.12
3.10
3.07
1.46
1.44
1.99
3.16
2.03
1.07
5.10
1.00
3.03
9
8
7
6
5
4
3
2
1
0
ppm

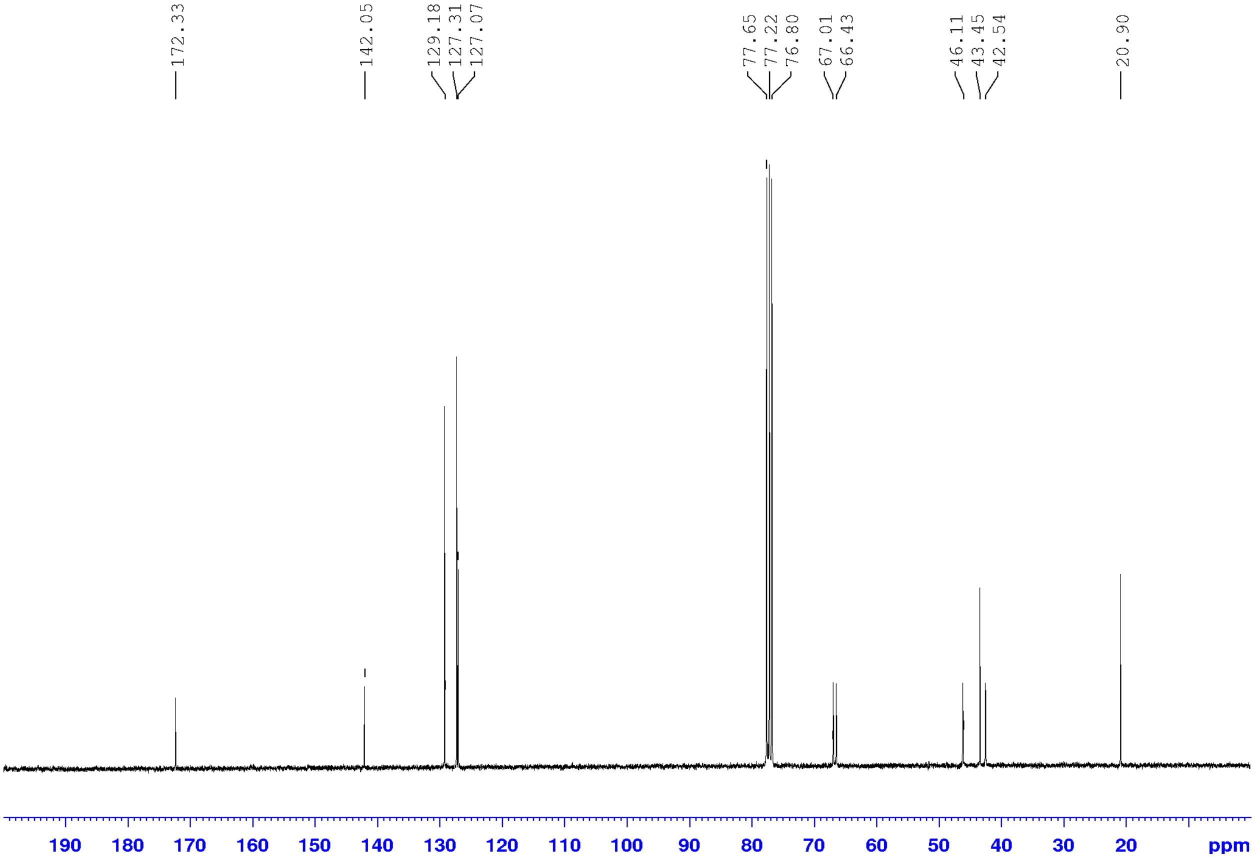
172.33
142.05
129.18
127.31
127.07
77.65
77.22
76.80
67.01
66.43
46.11
43.45
42.54
20.90
190
180
170
160
150
140
130
120
110
100
90
80
70
60
50
40
30
20
ppm

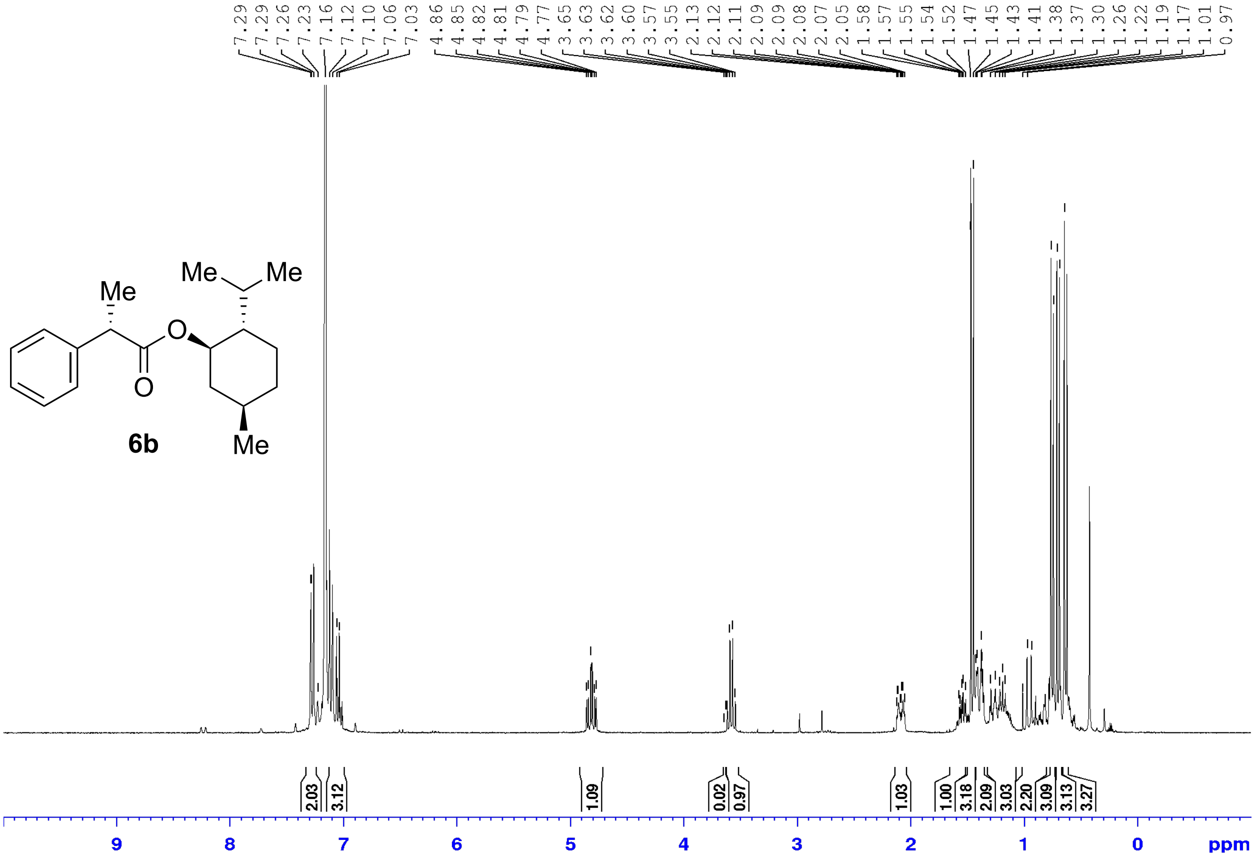
Me
Me
Me
O
O
Me
6b
7.29
7.29
7.26
7.23
7.16
7.12
7.10
7.06
7.03
4.86
4.85
4.82
4.81
4.79
4.77
3.65
3.63
3.62
3.60
3.57
3.55
2.13
2.12
2.11
2.09
2.09
2.08
2.07
2.05
1.58
1.57
1.55
1.54
1.52
1.47
1.45
1.43
1.41
1.38
1.37
1.30
1.26
1.22
1.19
1.17
1.01
0.97
2.03
3.12
1.09
0.02
0.97
1.03
1.00
3.18
2.09
3.03
2.20
3.09
3.13
3.27
9
8
7
6
5
4
3
2
1
0
ppm

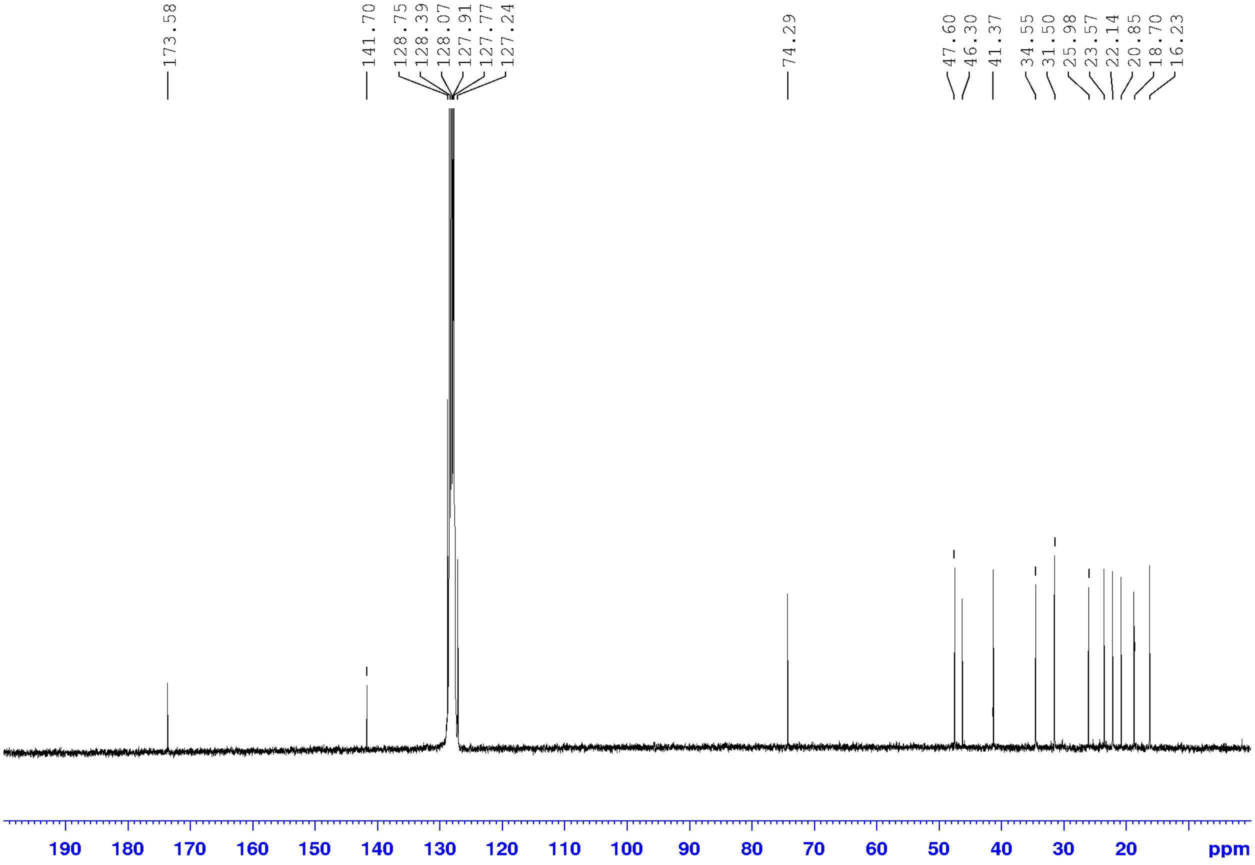
173.58
141.70
128.75
128.39
128.07
127.91
127.77
127.24
74.29
47.60
46.30
41.37
34.55
31.50
25.98
23.57
22.14
20.85
18.70
16.23
190
180
170
160
150
140
130
120
110
100
90
80
70
60
50
40
30
20
ppm

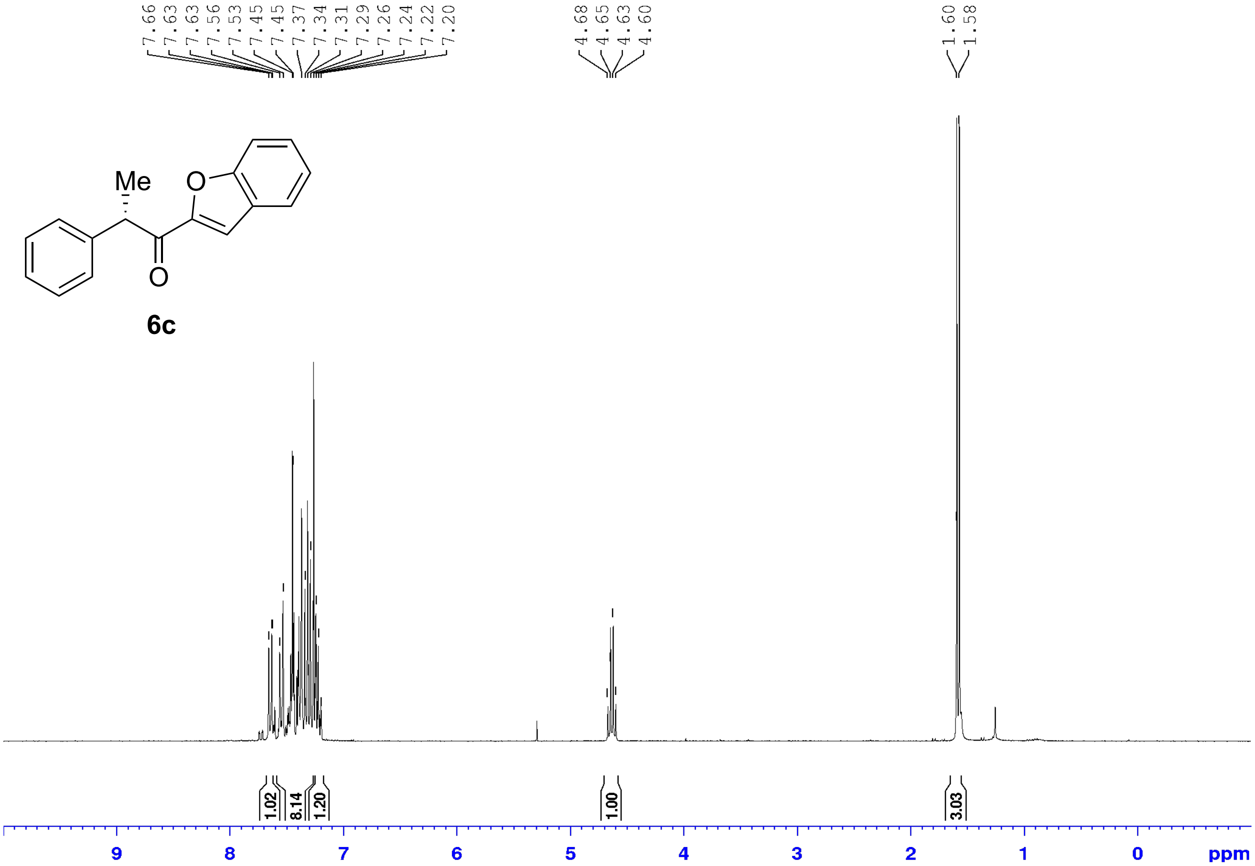
7.66
7.63
7.63
7.56
7.53
7.45
7.45
7.37
7.34
7.31
7.29
7.26
7.24
7.22
7.20
4.68
4.65
4.63
4.60
1.60
1.58
Me
O
O
6c
1.02
8.14
1.20
1.00
3.03
9
8
7
6
5
4
3
2
1
0
ppm

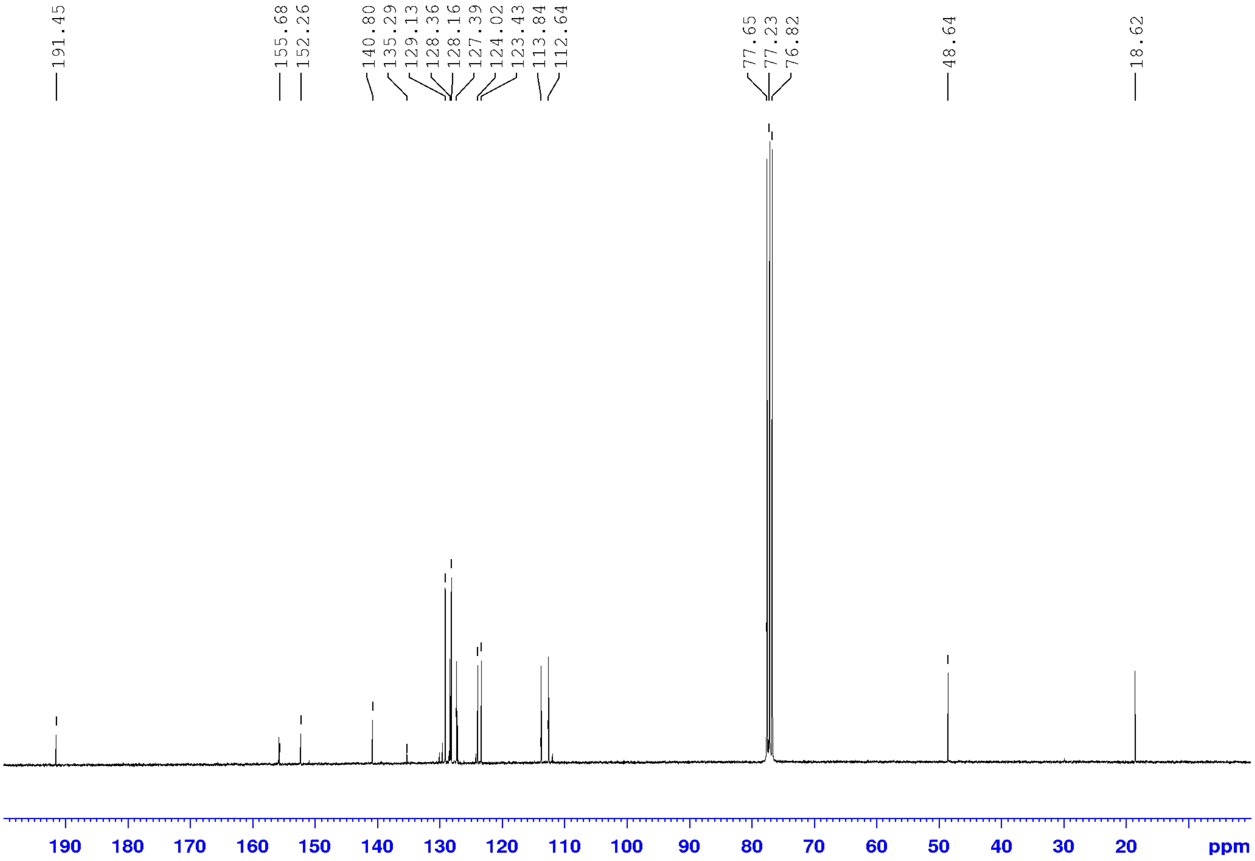
191.45
155.68
152.26
140.80
135.29
129.13
128.36
128.16
127.39
124.02
123.43
113.84
112.64
77.65
77.23
76.82
48.64
18.62
190
180
170
160
150
140
130
120
110
100
90
80
70
60
50
40
30
20
ppm

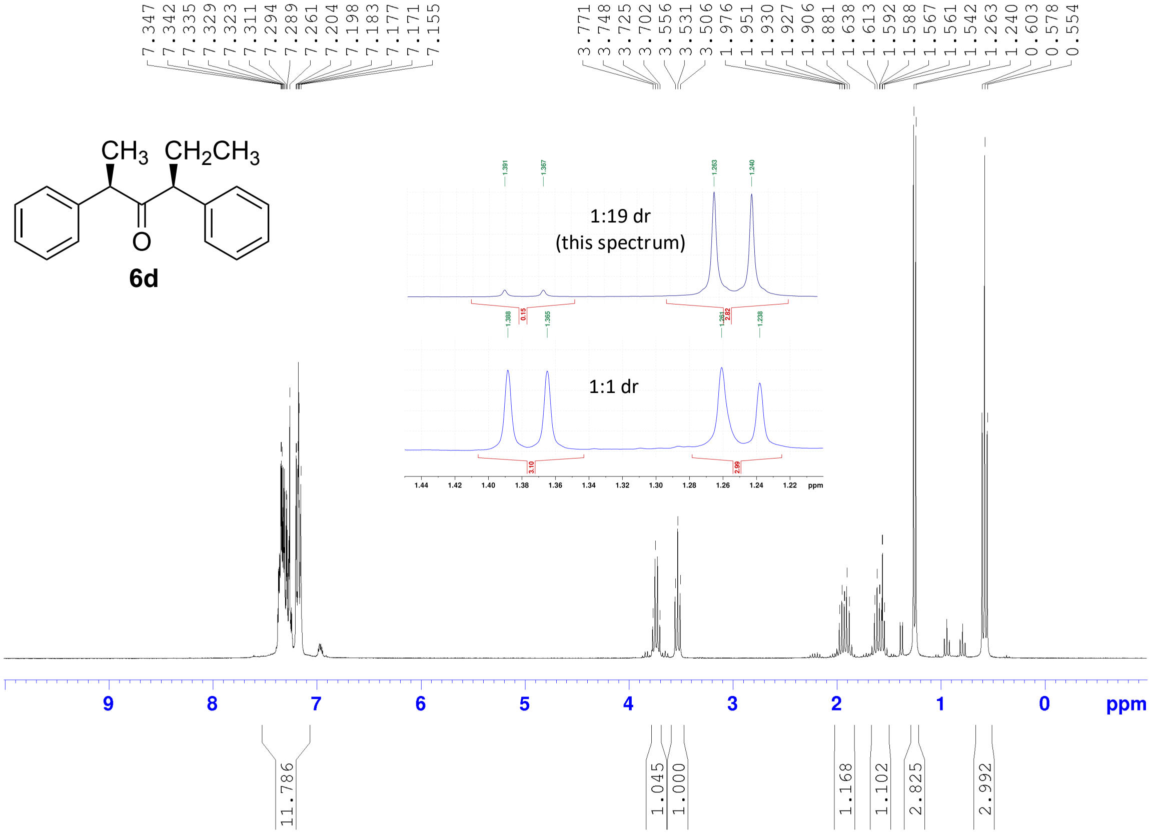

6d

7.347 7.342 7.335 7.329 7.323 7.311 7.294 7.289 7.261 7.204 7.198 7.183 7.177 7.171 7.155
3.771 3.748 3.725 3.702 3.556 3.531 3.506 1.976 1.951 1.930 1.927 1.906 1.881 1.638 1.613 1.592 1.588 1.567 1.561 1.542 1.263 1.240 0.603 0.578 0.554
1:19 dr
(this spectrum)
1:1 dr
11.786
1.045
1.000
1.168
1.102
2.825
2.992
ppm

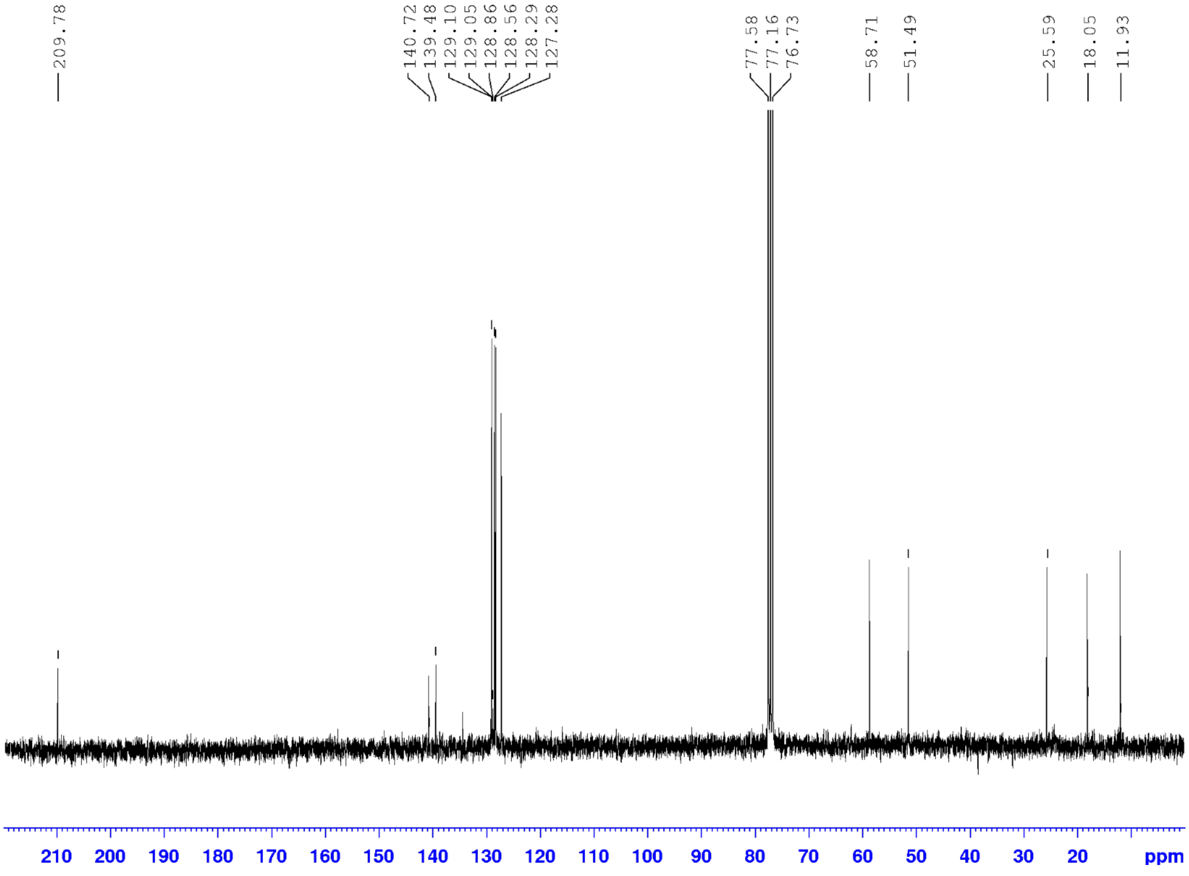
209.78
140.72 139.48 129.10 129.05 128.86 128.56 128.29 127.28
77.58 77.16 76.73
58.71
51.49
25.59
18.05
11.93
ppm

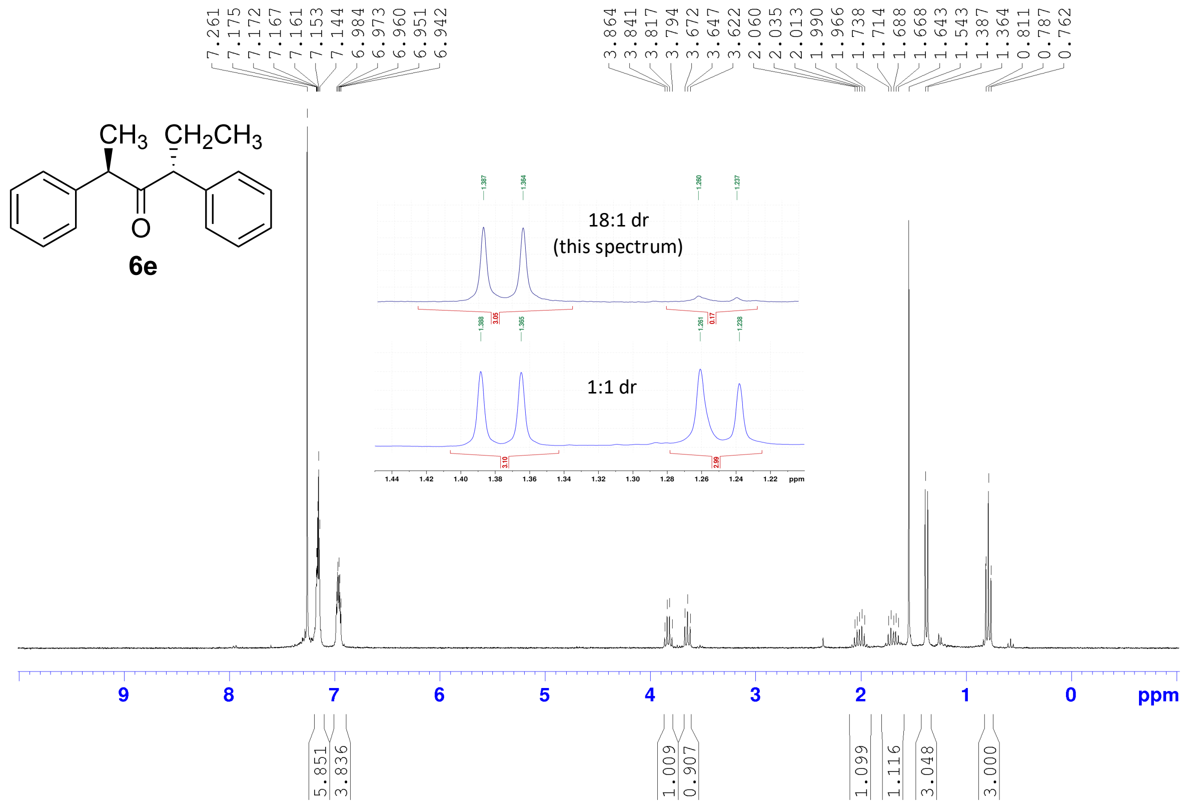

CH_3 CH_2CH_3
O
6e
18:1 dr
(this spectrum)
1:1 dr
7.261 7.175 7.172 7.167 7.161 7.153 7.144 6.984 6.973 6.960 6.951 6.942
3.864 3.841 3.817 3.794 3.672 3.647 3.622 2.060 2.035 2.013 1.990 1.966 1.738 1.714 1.688 1.668 1.643 1.543 1.387 1.364 0.811 0.787 0.762
5.851 3.836 1.009 0.907 1.099 1.116 3.048 3.000
9 8 7 6 5 4 3 2 1 0 ppm

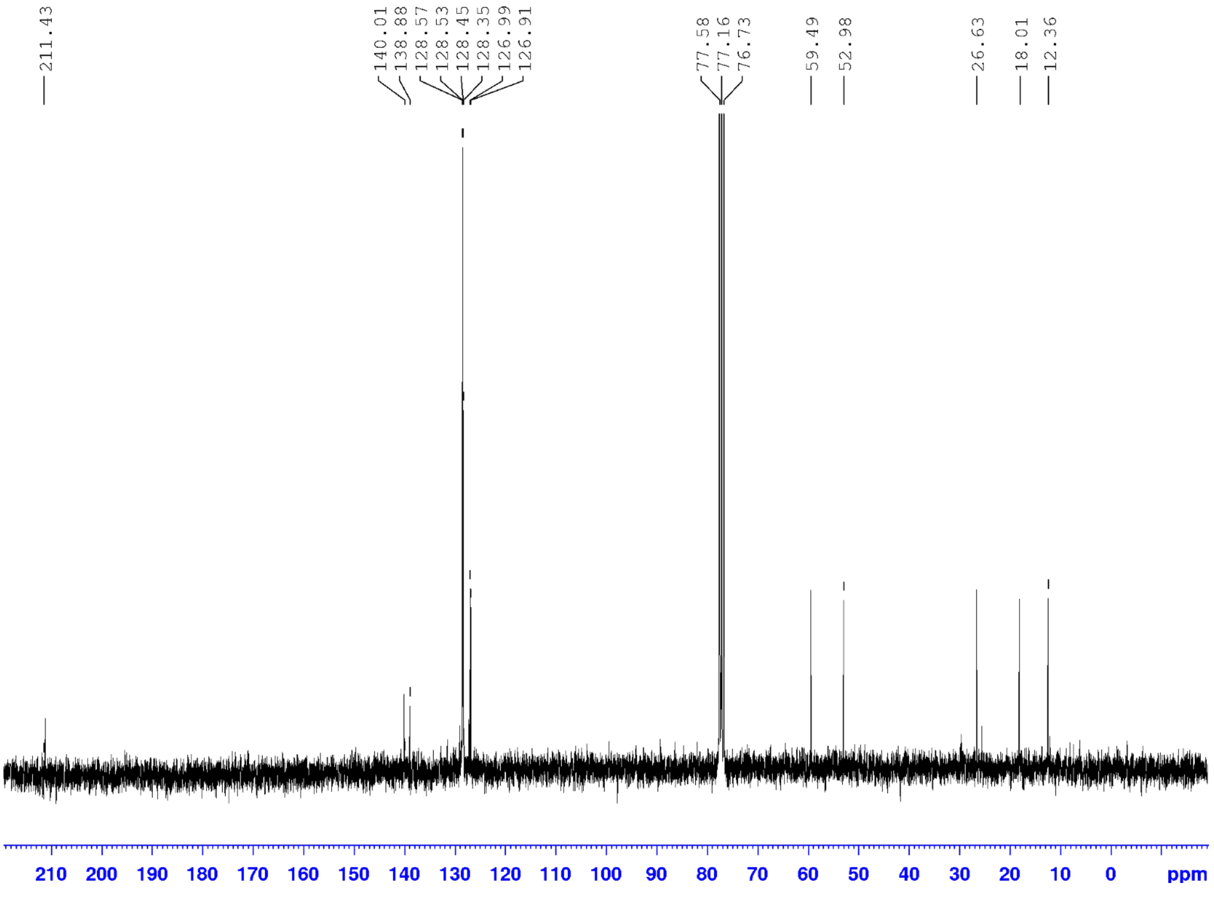

211.43
140.01 138.88 128.57 128.53 128.45 128.35 126.99 126.91
77.58 77.16 76.73
59.49 52.98
26.63 18.01 12.36
210 200 190 180 170 160 150 140 130 120 110 100 90 80 70 60 50 40 30 20 10 0 ppm

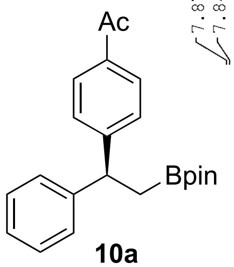
Ac
Bpin

10a

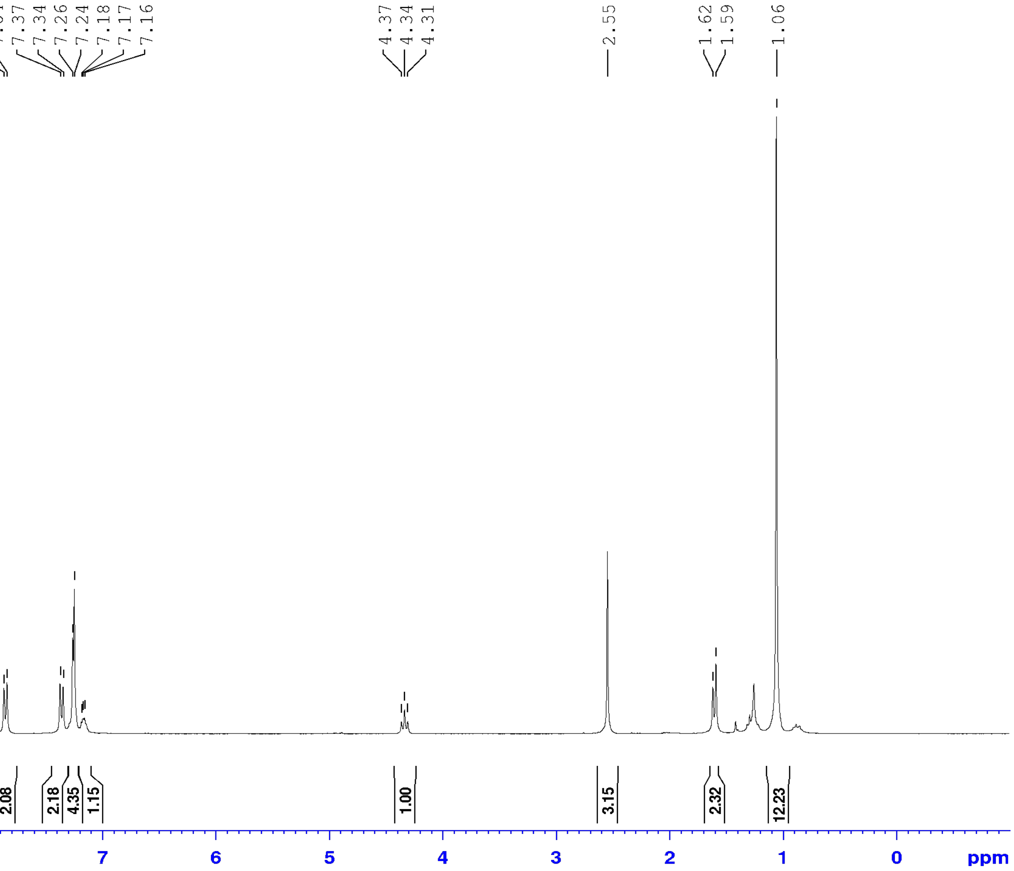
7.87
7.84
7.37
7.34
7.26
7.24
7.18
7.17
7.16
4.37
4.34
4.31
2.55
1.62
1.59
1.06
2.08
2.18
4.35
1.15
1.00
3.15
2.32
12.23
9
8
7
6
5
4
3
2
1
0
ppm

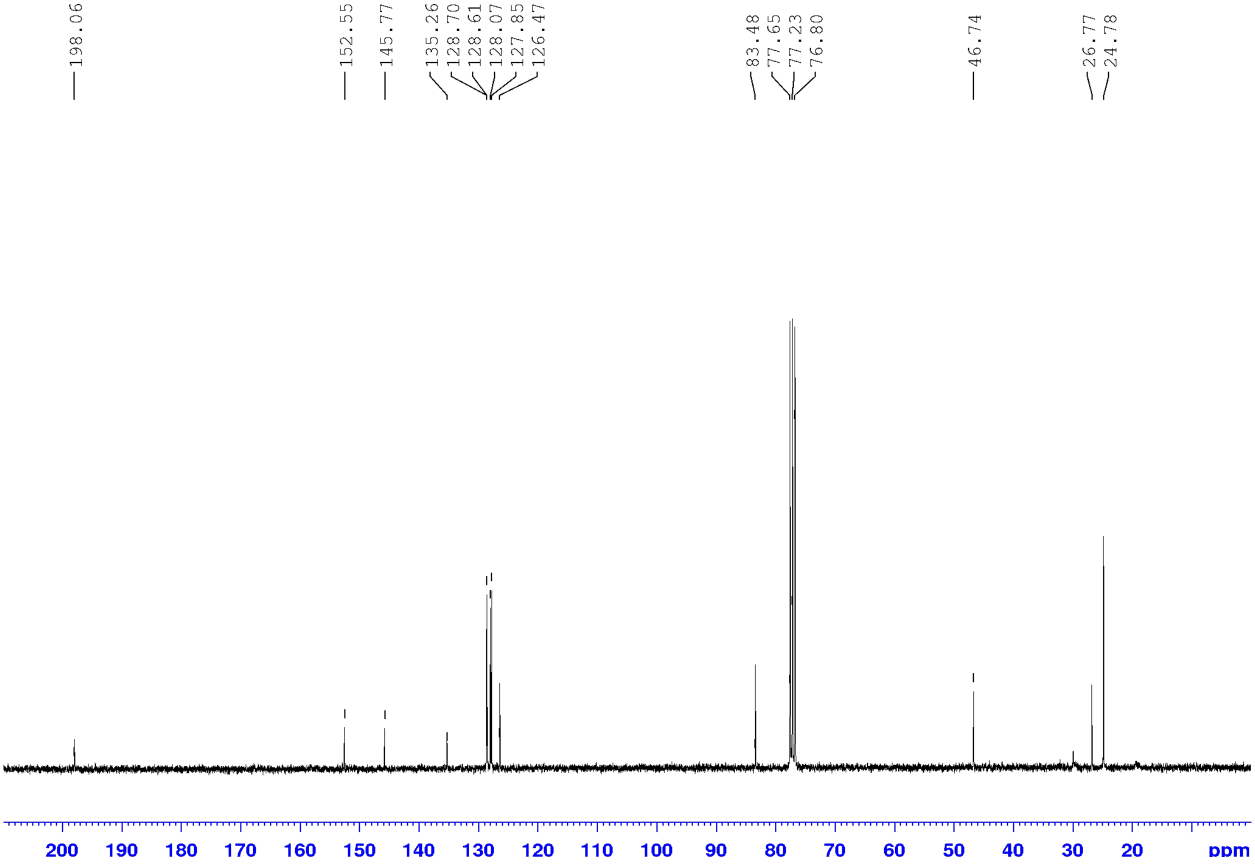
198.06
152.55
145.77
135.26
128.70
128.61
128.07
127.85
126.47
83.48
77.65
77.23
76.80
46.74
26.77
24.78
200
190
180
170
160
150
140
130
120
110
100
90
80
70
60
50
40
30
20
ppm

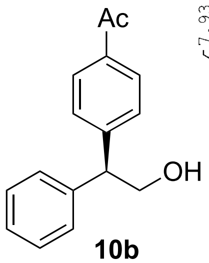

10b

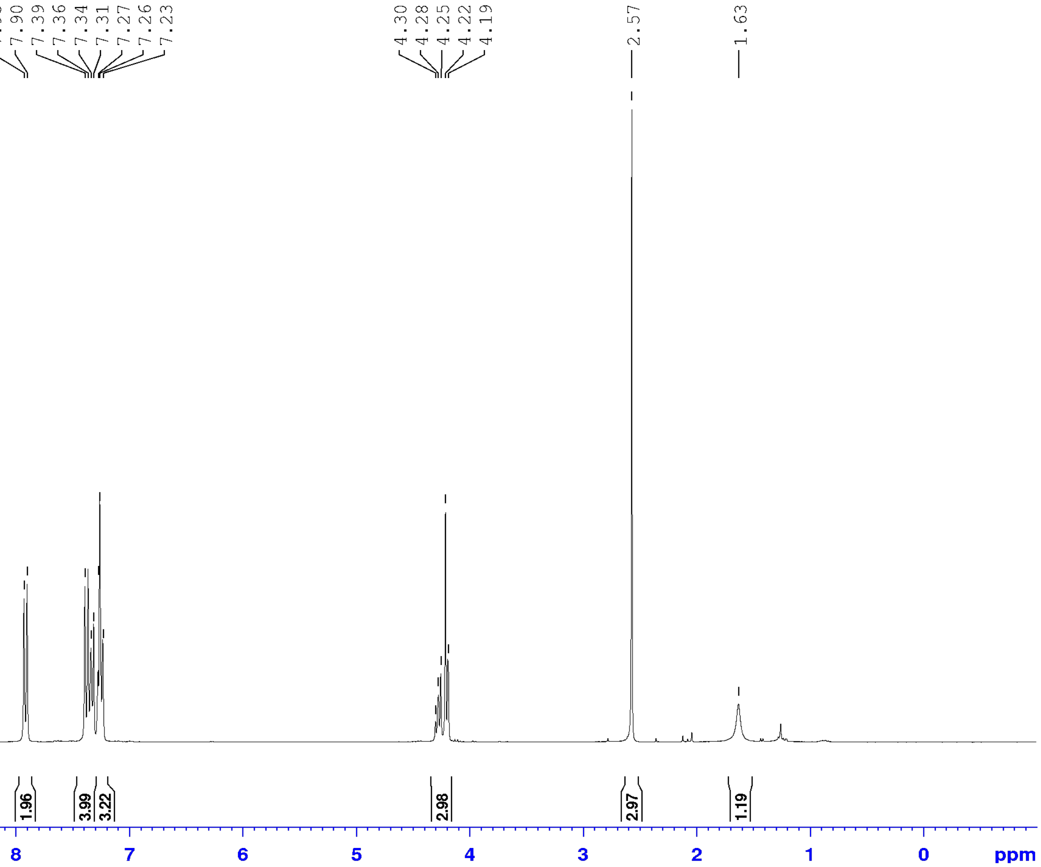
7.93
7.90
7.39
7.36
7.34
7.31
7.27
7.26
7.23
4.30
4.28
4.25
4.22
4.19
2.57
1.63
1.96
3.99
3.22
2.98
2.97
1.19
9
8
7
6
5
4
3
2
1
0
ppm

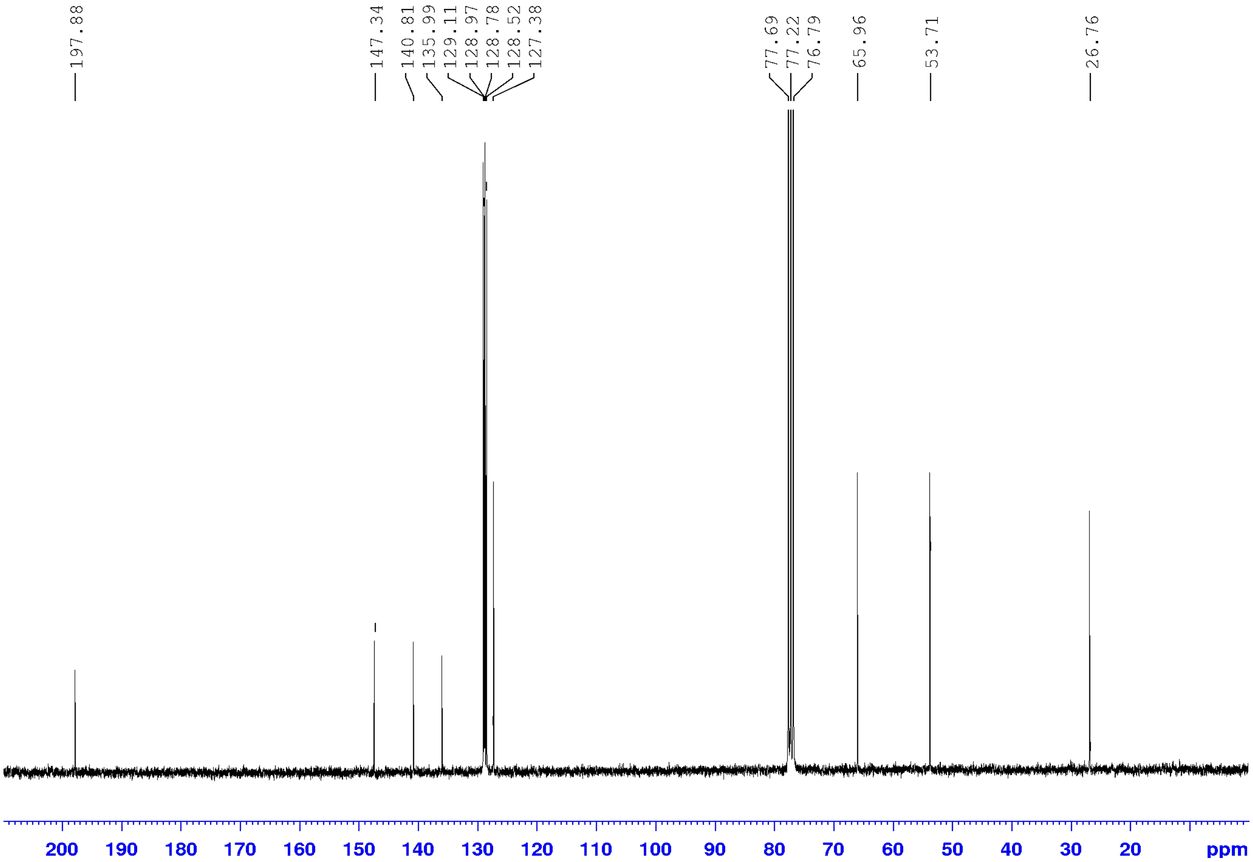
197.88
147.34
140.81
135.99
129.11
128.97
128.78
128.52
127.38
77.69
77.22
76.79
65.96
53.71
26.76
200
190
180
170
160
150
140
130
120
110
100
90
80
70
60
50
40
30
20
ppm

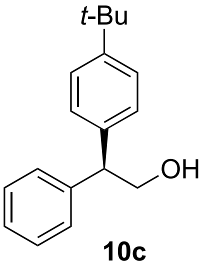
t-Bu
OH

10c

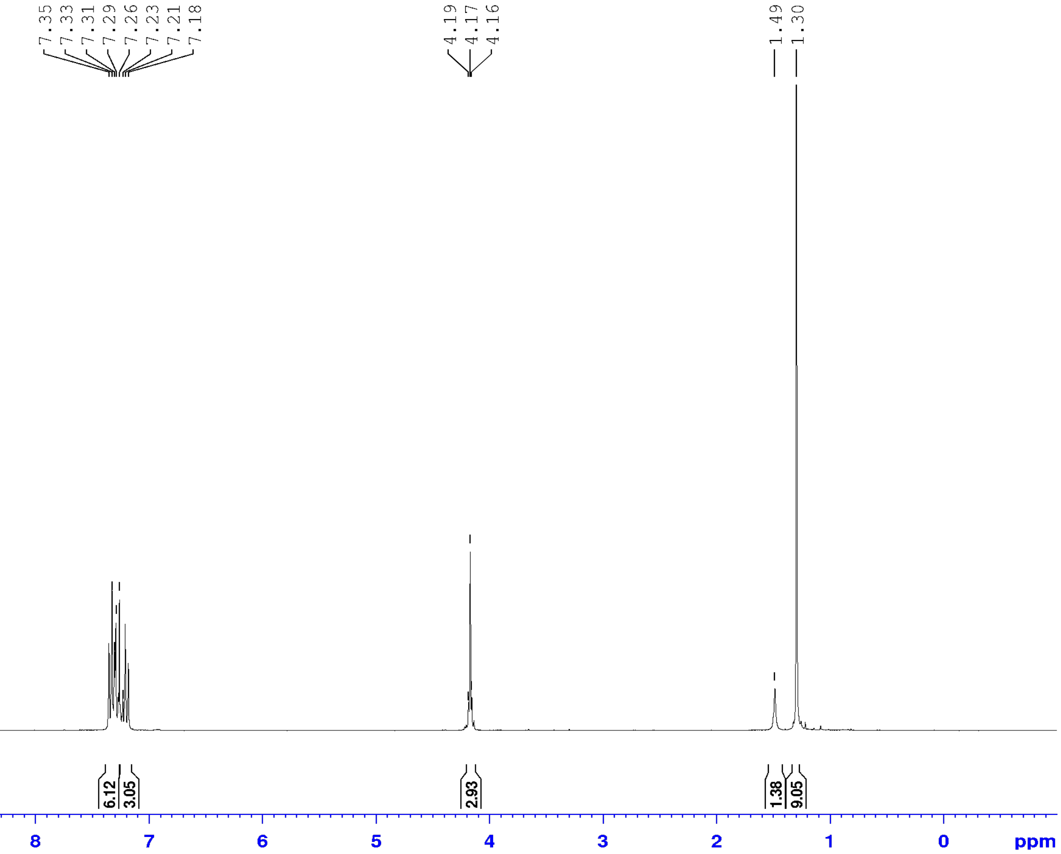
7.35 7.33 7.31 7.29 7.26 7.23 7.21 7.18
4.19 4.17 4.16
1.49 1.30
6.12 3.05 2.93 1.38 9.05
9 8 7 6 5 4 3 2 1 0 ppm

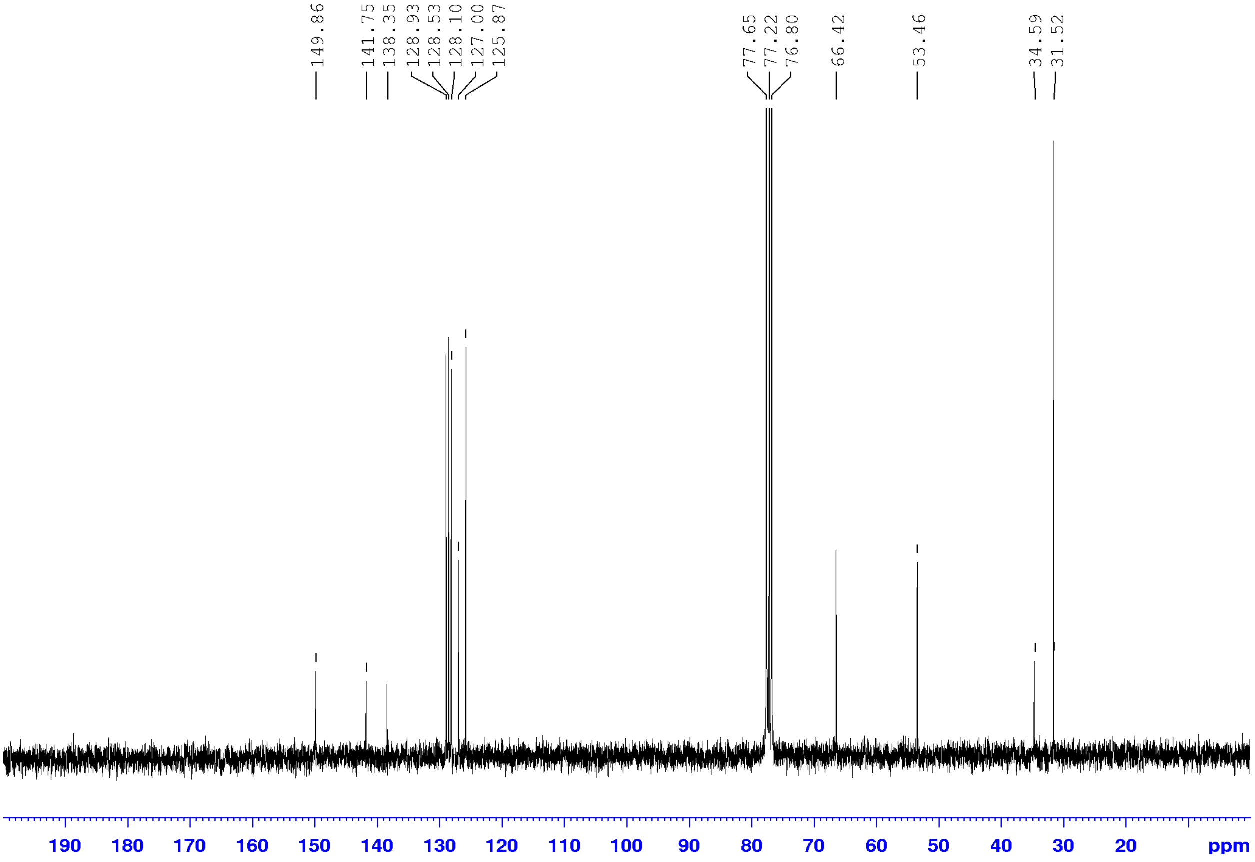
149.86
141.75 138.35 128.93 128.53 128.10 127.00 125.87
77.65 77.22 76.80
66.42
53.46
34.59 31.52
190 180 170 160 150 140 130 120 110 100 90 80 70 60 50 40 30 20 ppm

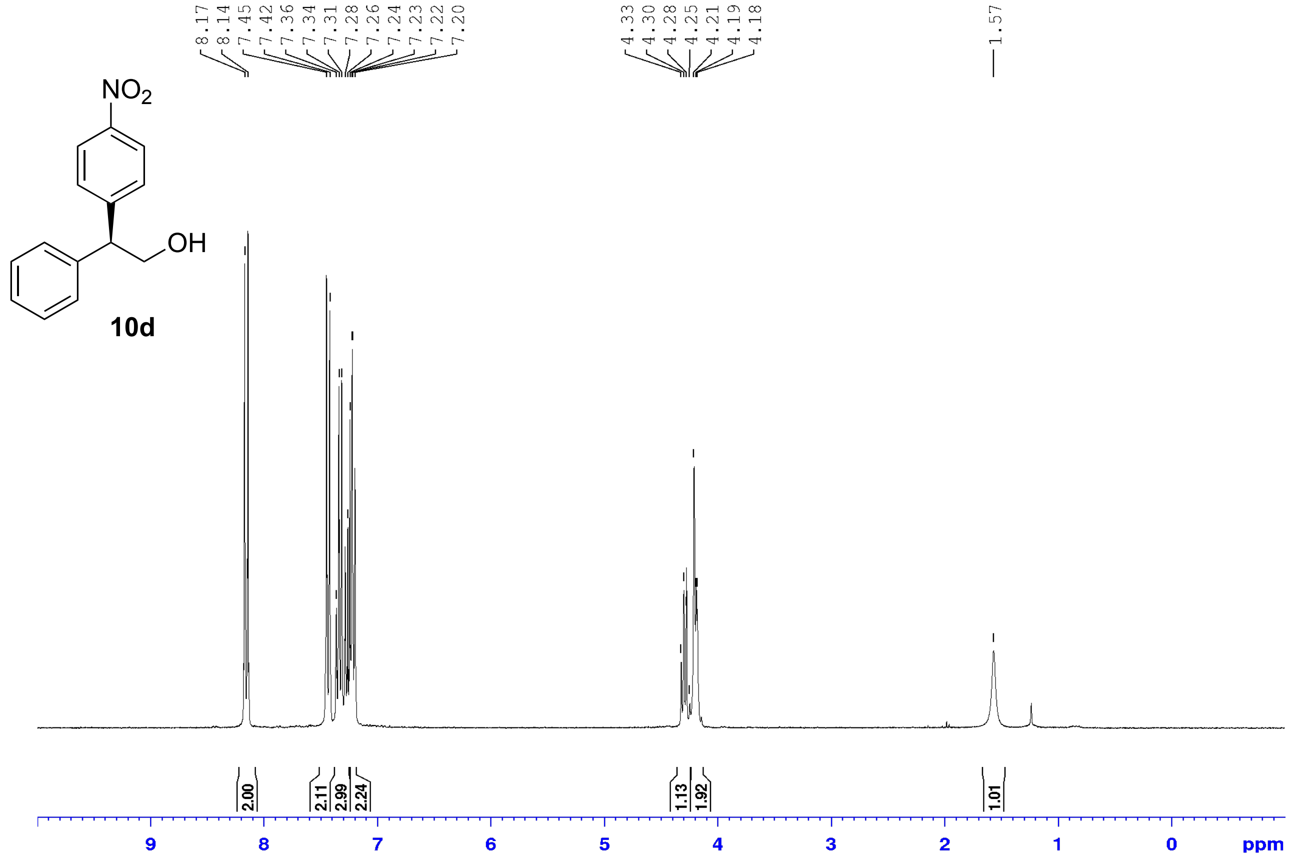
NO2
OH
10d
8.17
8.14
7.45
7.42
7.36
7.34
7.31
7.28
7.26
7.24
7.23
7.22
7.20
4.33
4.30
4.28
4.25
4.21
4.19
4.18
1.57
2.00
2.11
2.99
2.24
1.13
1.92
1.01
9
8
7
6
5
4
3
2
1
0
ppm

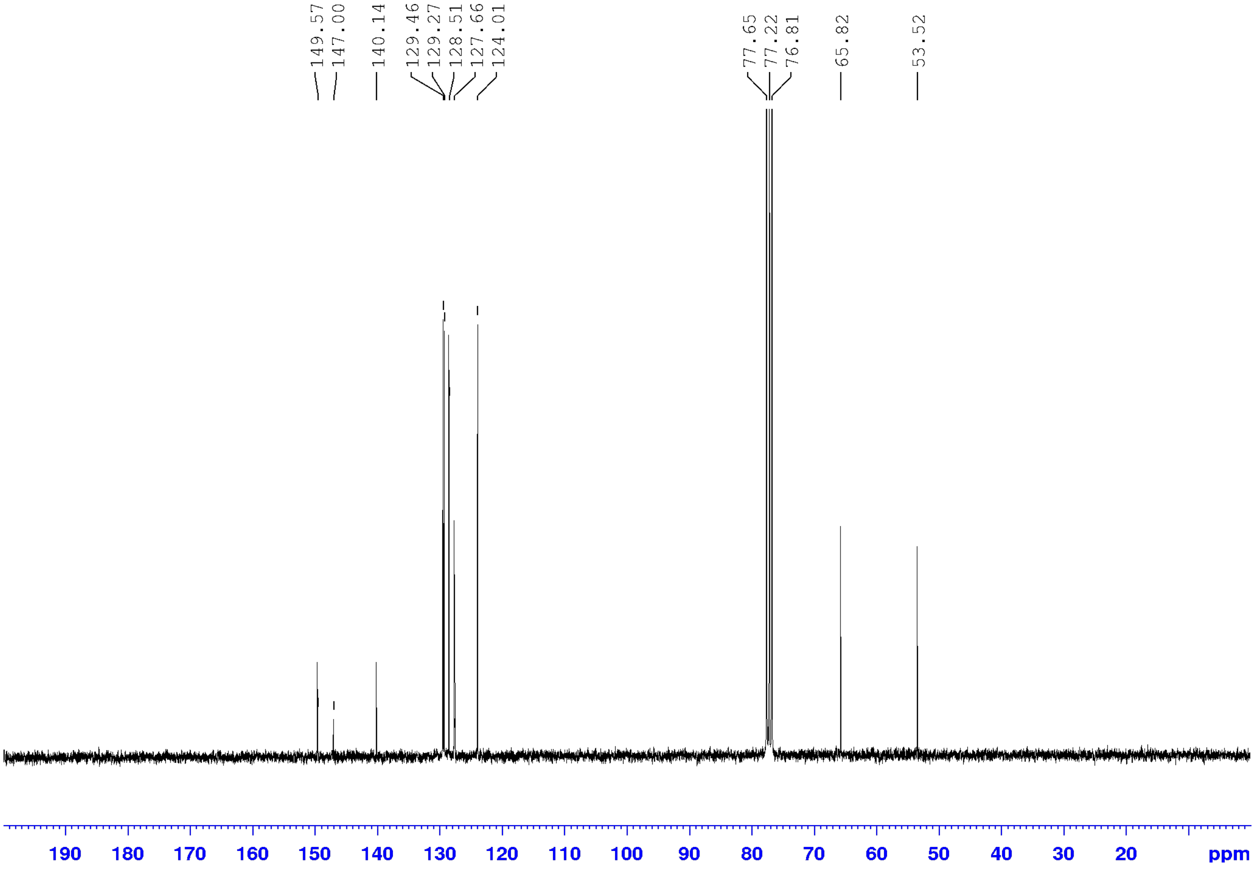
149.57
147.00
140.14
129.46
129.27
128.51
127.66
124.01
77.65
77.22
76.81
65.82
53.52
190
180
170
160
150
140
130
120
110
100
90
80
70
60
50
40
30
20
ppm

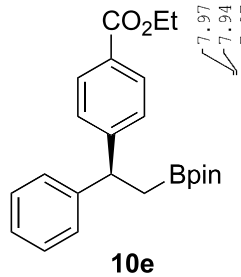

CO_2Et
Bpin

10e

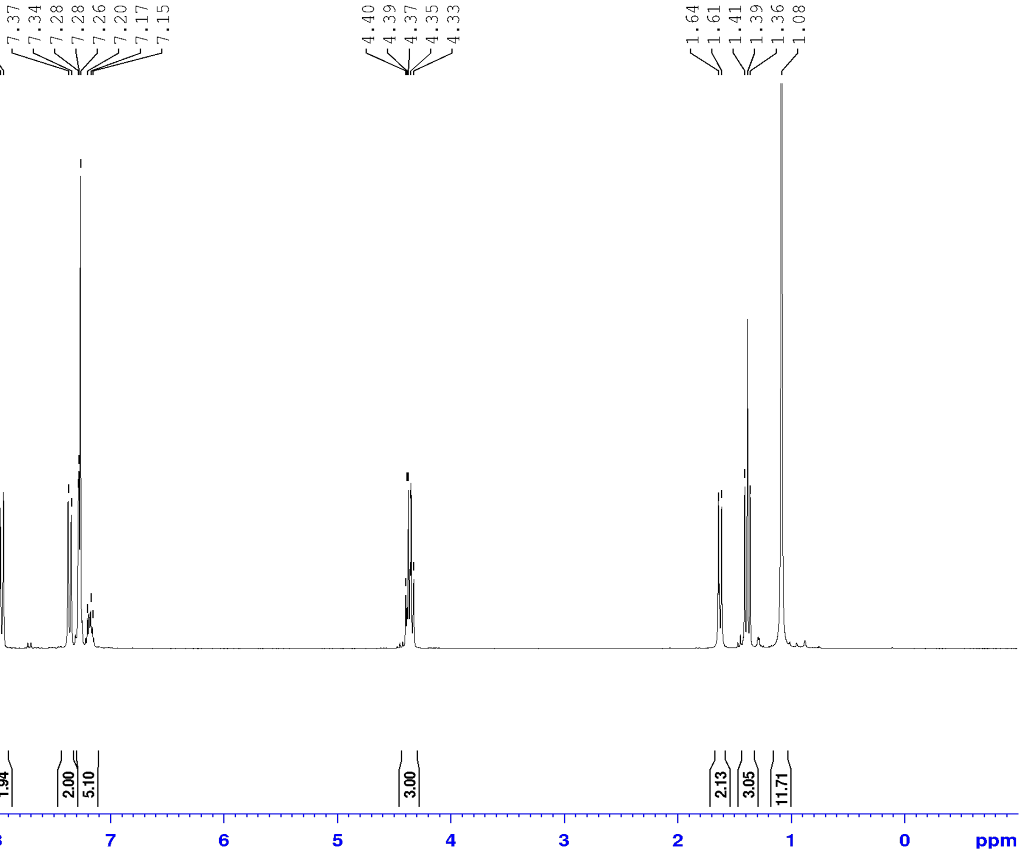

7.97 7.94 7.37 7.34 7.28 7.28 7.26 7.20 7.17 7.15
4.40 4.39 4.37 4.35 4.33
1.64 1.61 1.41 1.39 1.36 1.08
1.94 2.00 5.10 3.00 2.13 3.05 11.71
9 8 7 6 5 4 3 2 1 0 ppm

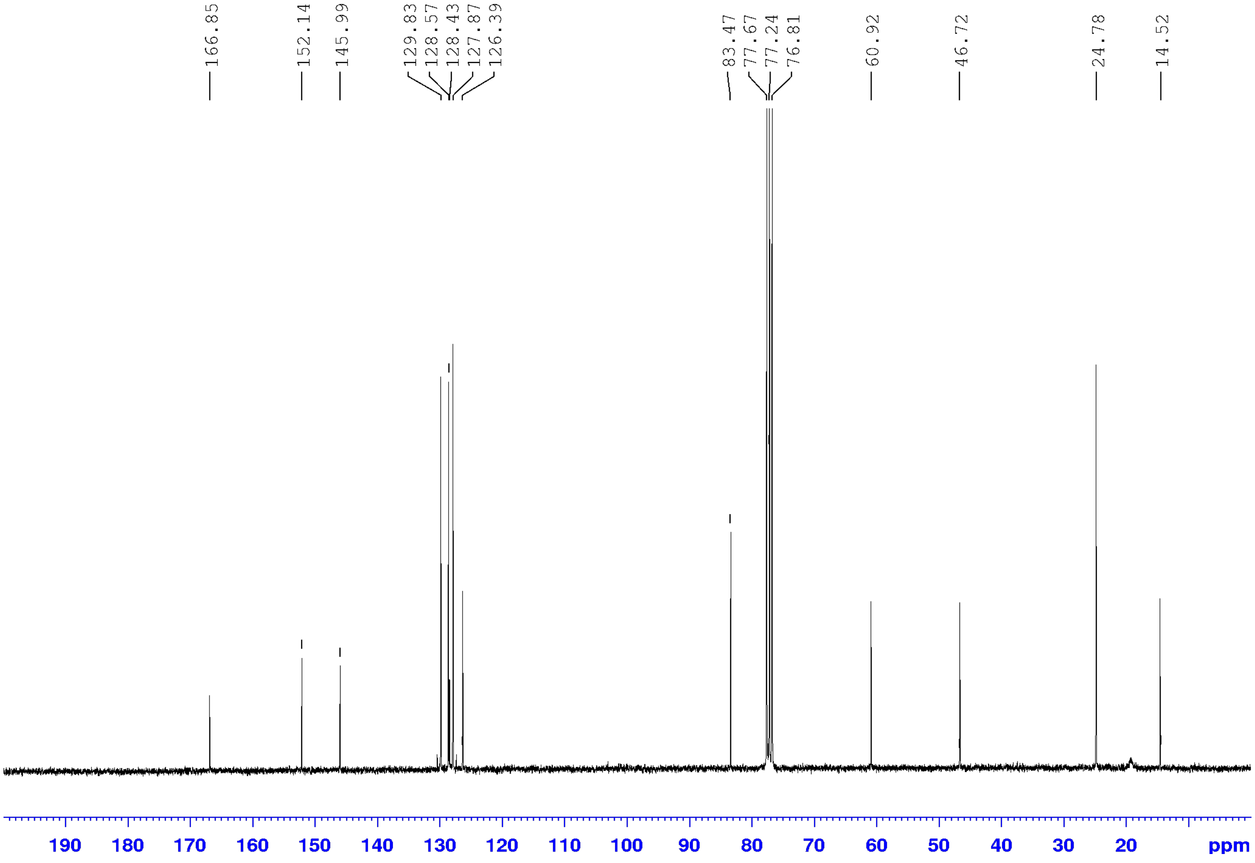

166.85
152.14
145.99
129.83 128.57 128.43 127.87 126.39
83.47 77.67 77.24 76.81
60.92
46.72
24.78
14.52
190 180 170 160 150 140 130 120 110 100 90 80 70 60 50 40 30 20 ppm

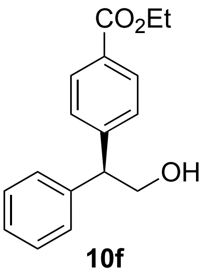
CO2Et
OH

10f

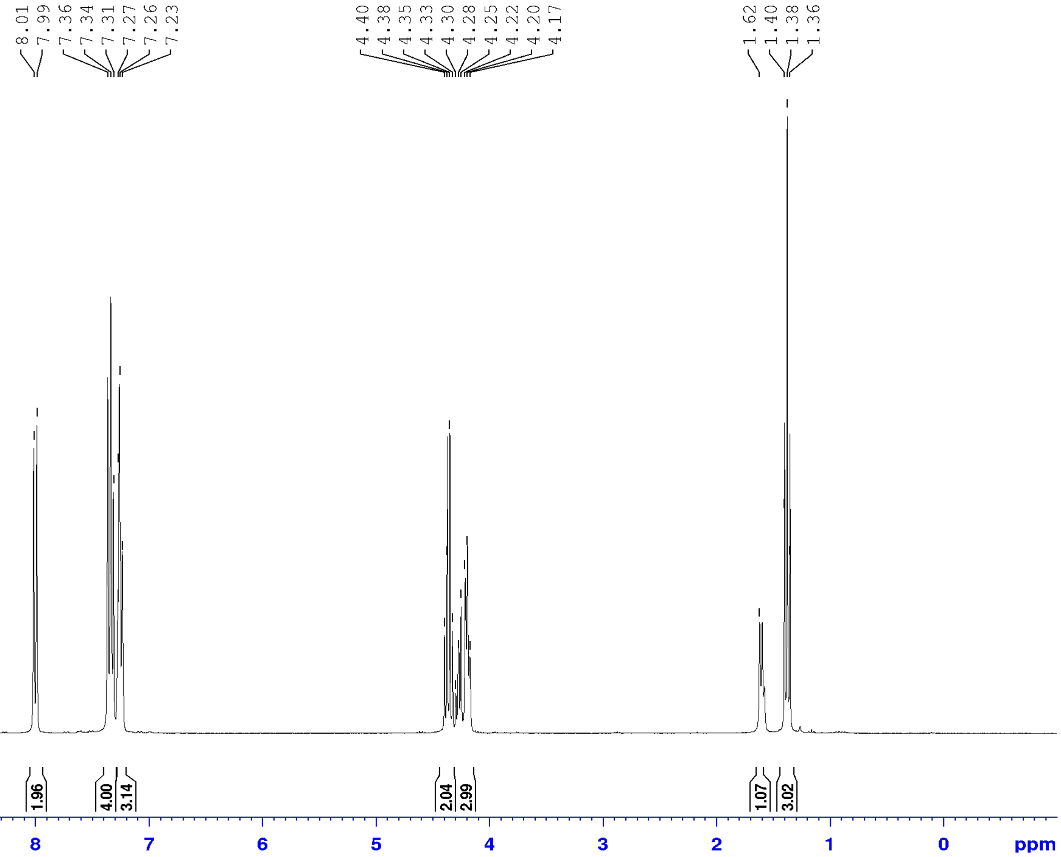
8.01
7.99
7.36
7.34
7.31
7.27
7.26
7.23
4.40
4.38
4.35
4.33
4.30
4.28
4.25
4.22
4.20
4.17
1.62
1.40
1.38
1.36
1.96
4.00
3.14
2.04
2.99
1.07
3.02
ppm

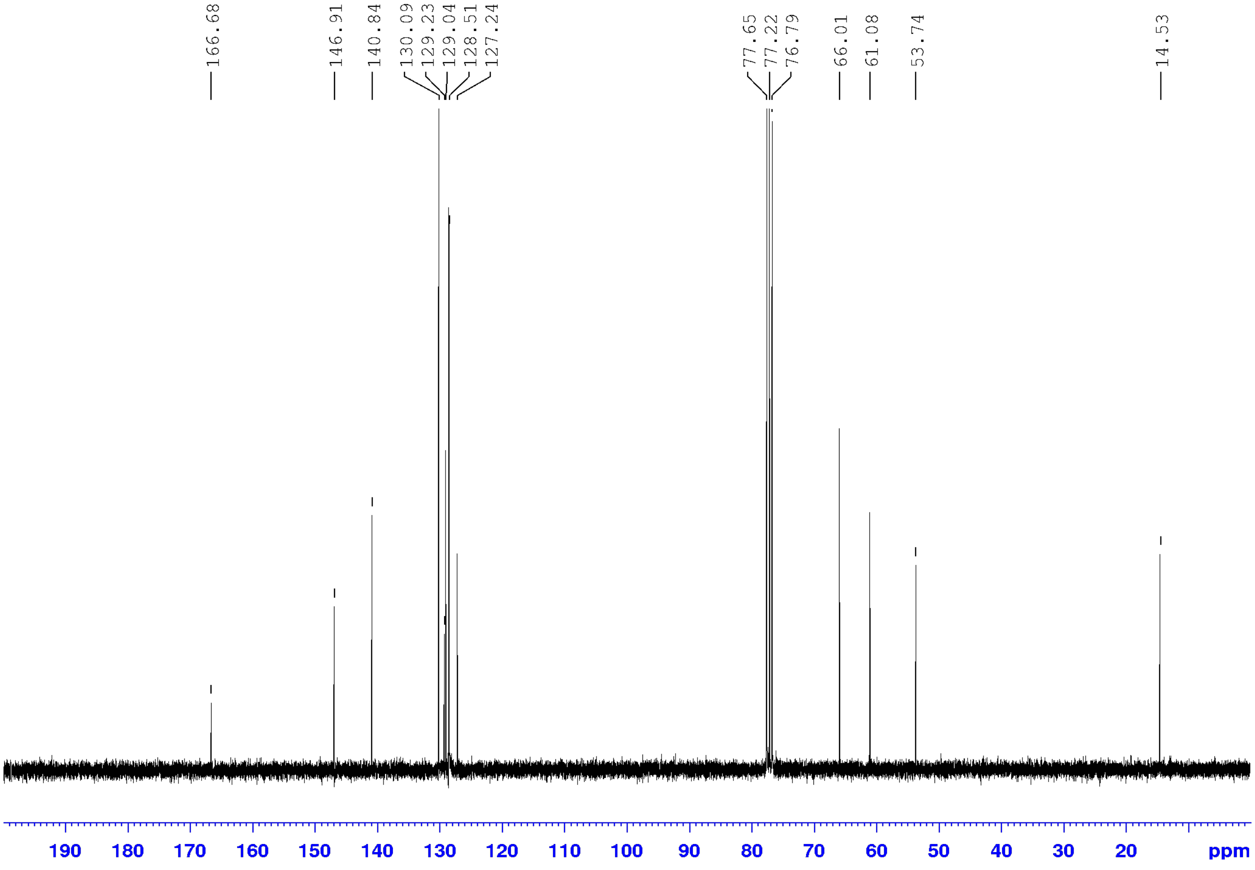
166.68
146.91
140.84
130.09
129.23
129.04
128.51
127.24
77.65
77.22
76.79
66.01
61.08
53.74
14.53
ppm

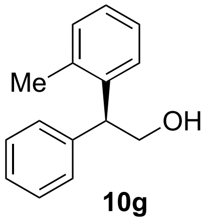

Me
OH

10g

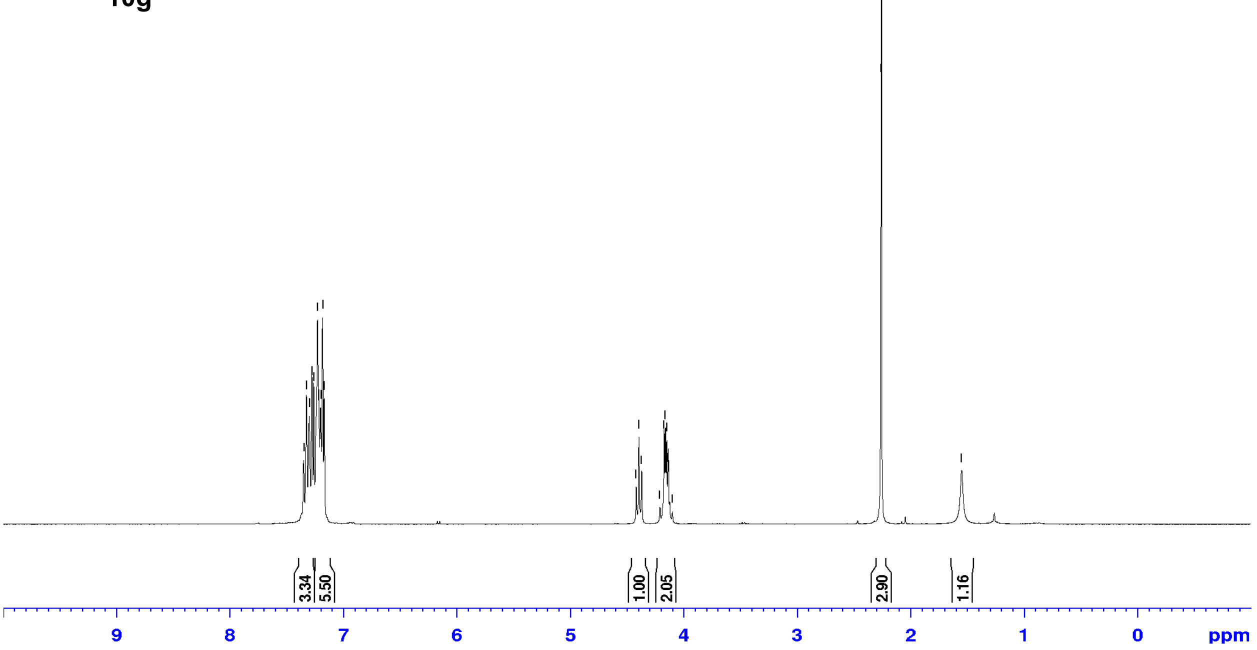

7.35
7.33
7.30
7.28
7.26
7.23
7.20
7.18
7.17
4.42
4.40
4.38
4.22
4.18
4.17
4.15
4.14
4.10
2.26
1.56
3.34
5.50
1.00
2.05
2.90
1.16
9 8 7 6 5 4 3 2 1 0 ppm

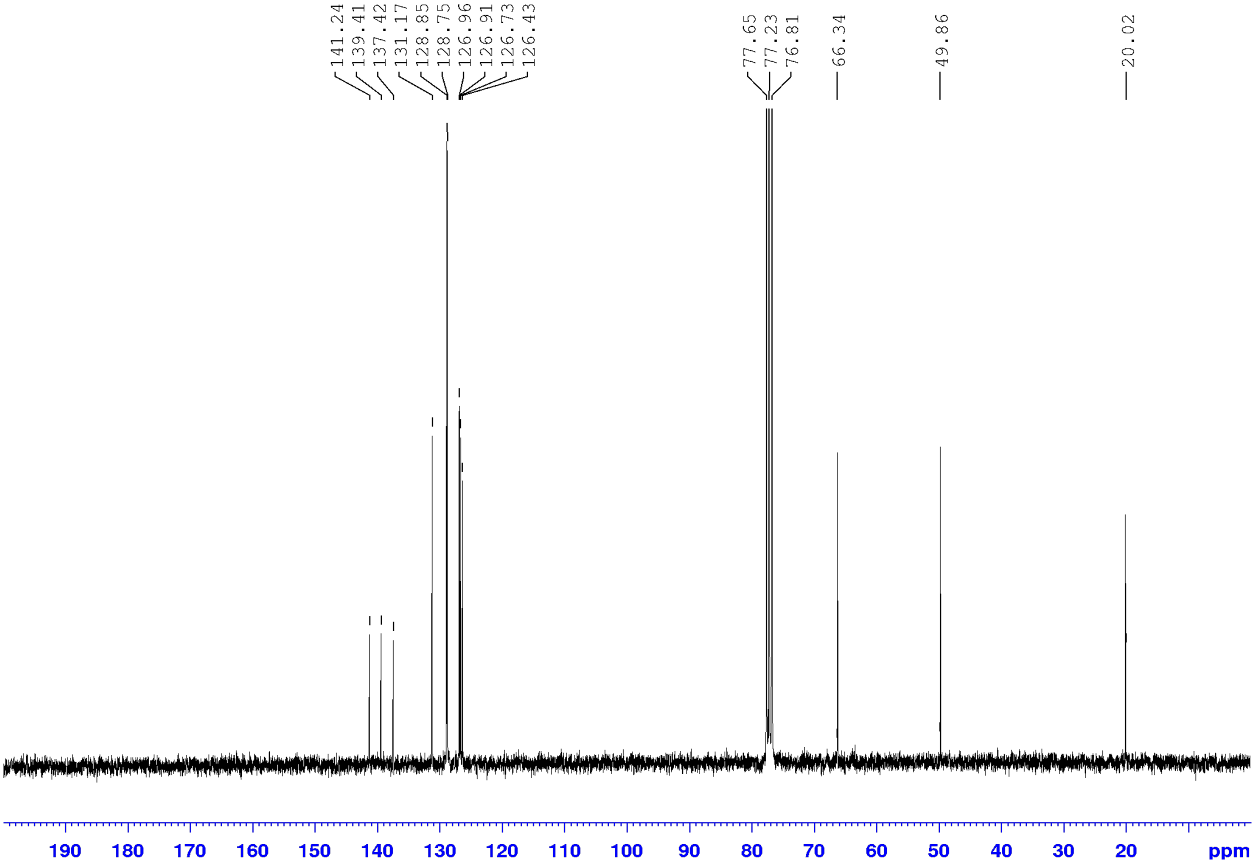

141.24
139.41
137.42
131.17
128.85
128.75
126.96
126.91
126.73
126.43
77.65
77.23
76.81
66.34
49.86
20.02
190 180 170 160 150 140 130 120 110 100 90 80 70 60 50 40 30 20 ppm

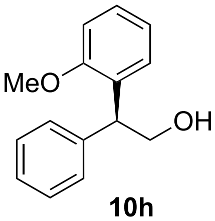
MeO
OH

10h

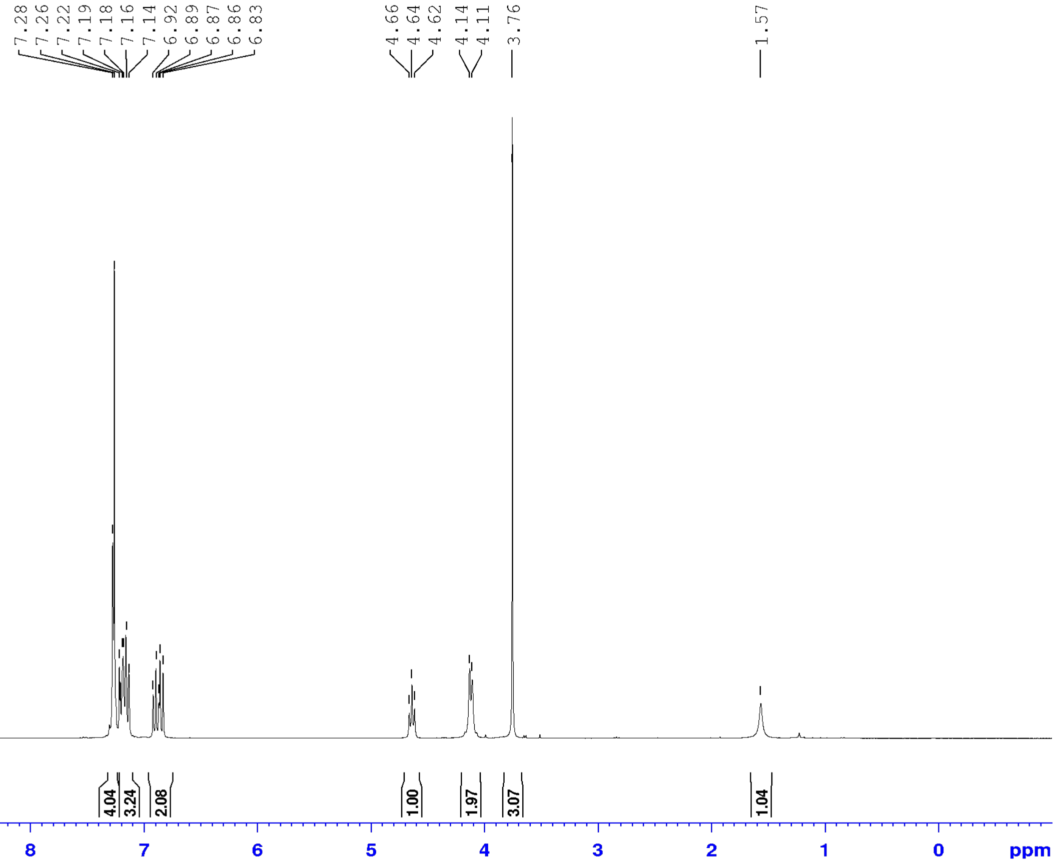
7.28
7.26
7.22
7.19
7.18
7.16
7.14
6.92
6.89
6.87
6.86
6.83
4.66
4.64
4.62
4.14
4.11
3.76
1.57
4.04
3.24
2.08
1.00
1.97
3.07
1.04
9
8
7
6
5
4
3
2
1
0
ppm

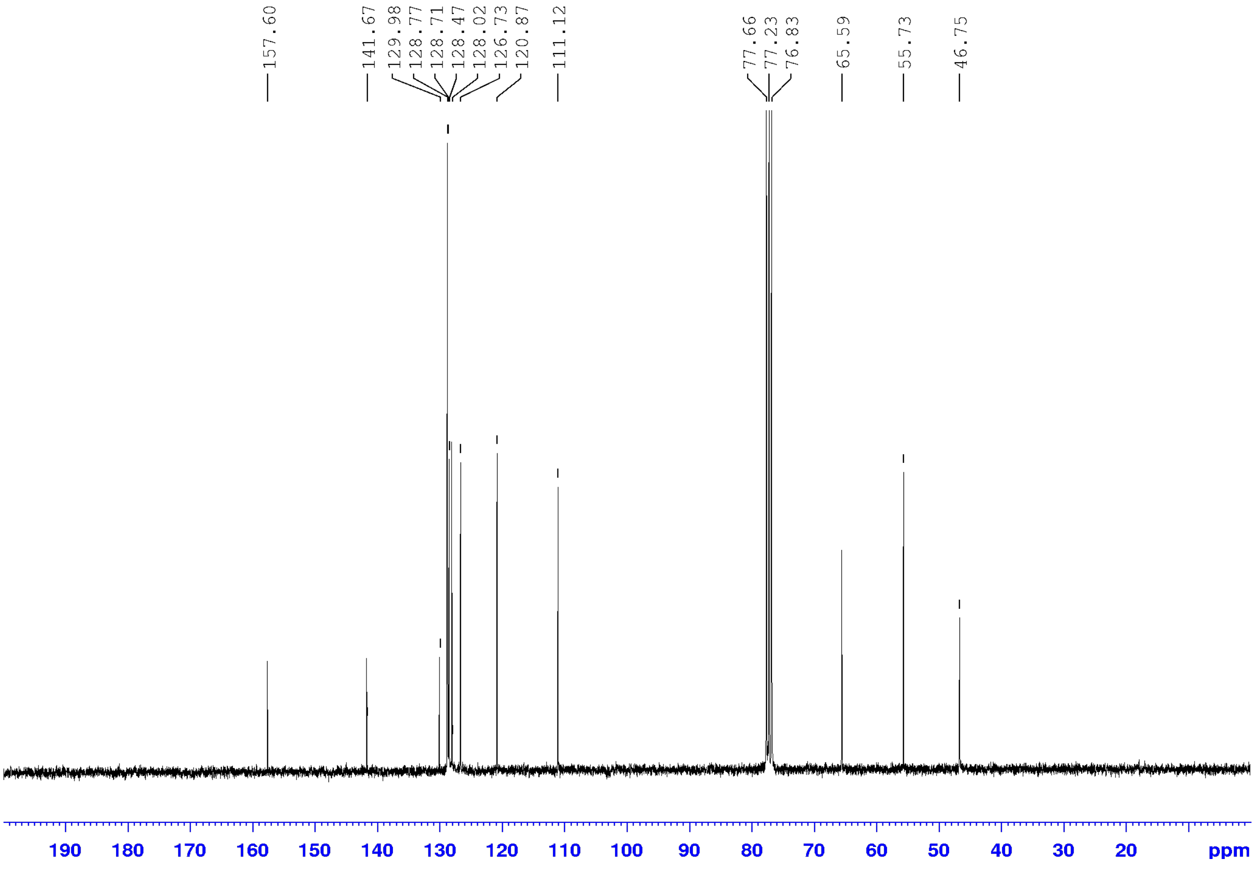
157.60
141.67
129.98
128.77
128.71
128.47
128.02
126.73
120.87
111.12
77.66
77.23
76.83
65.59
55.73
46.75
190
180
170
160
150
140
130
120
110
100
90
80
70
60
50
40
30
20
ppm

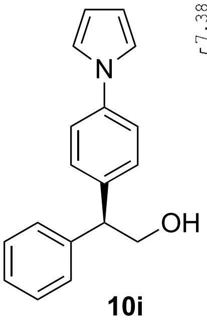
N
OH

10i

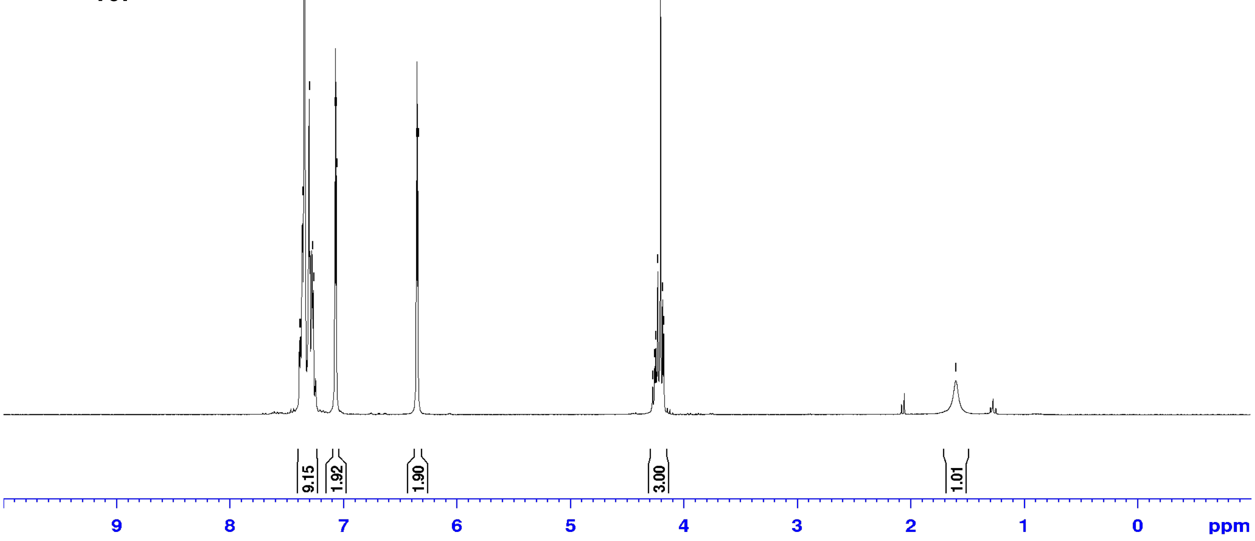
7.38
7.38
7.36
7.35
7.34
7.30
7.29
7.28
7.26
7.07
7.07
7.06
6.35
6.35
6.34
4.28
4.26
4.25
4.23
4.21
4.19
4.18
1.60
9.15
1.92
1.90
3.00
1.01
9
8
7
6
5
4
3
2
1
0
ppm

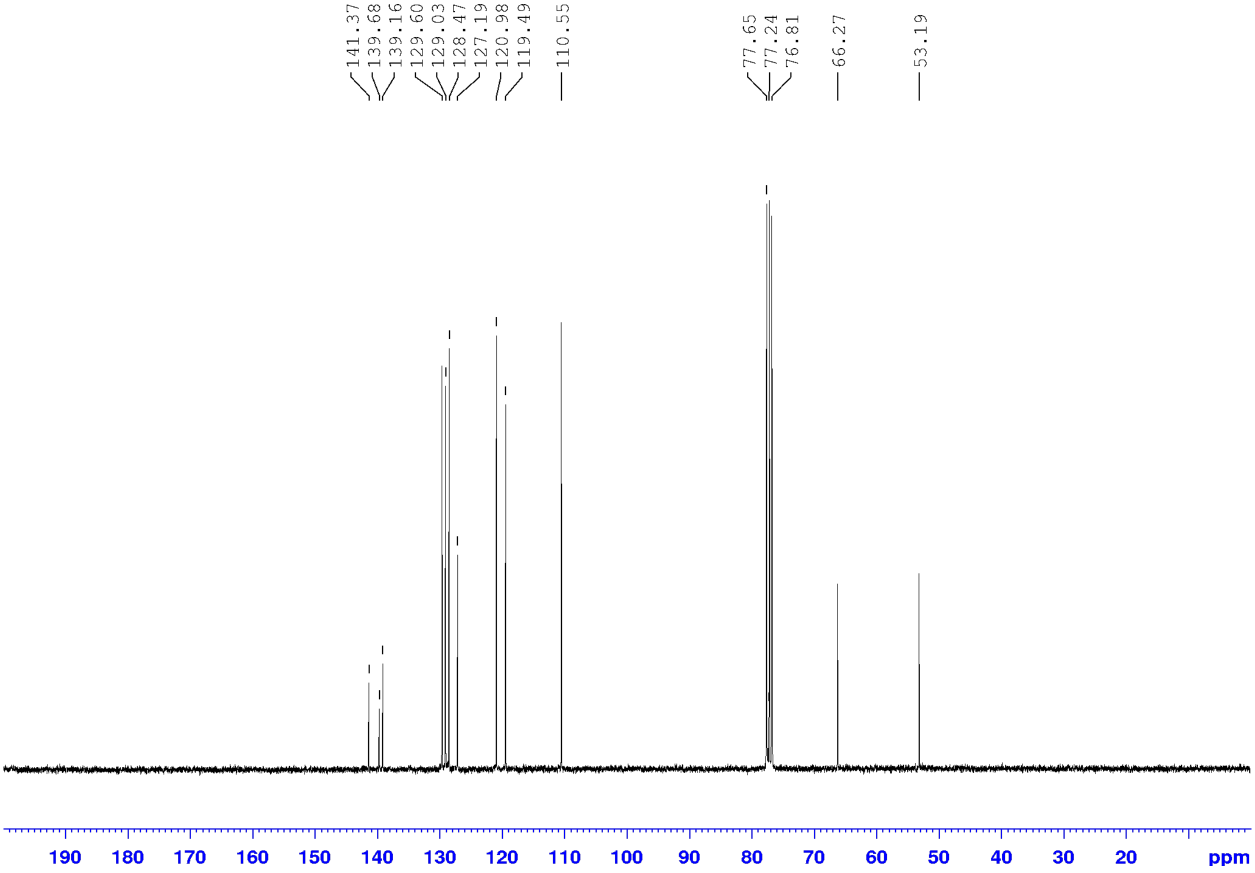
141.37
139.68
139.16
129.60
129.03
128.47
127.19
120.98
119.49
110.55
77.65
77.24
76.81
66.27
53.19
190
180
170
160
150
140
130
120
110
100
90
80
70
60
50
40
30
20
ppm

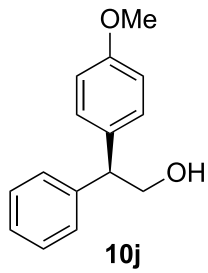
OMe
OH

10j

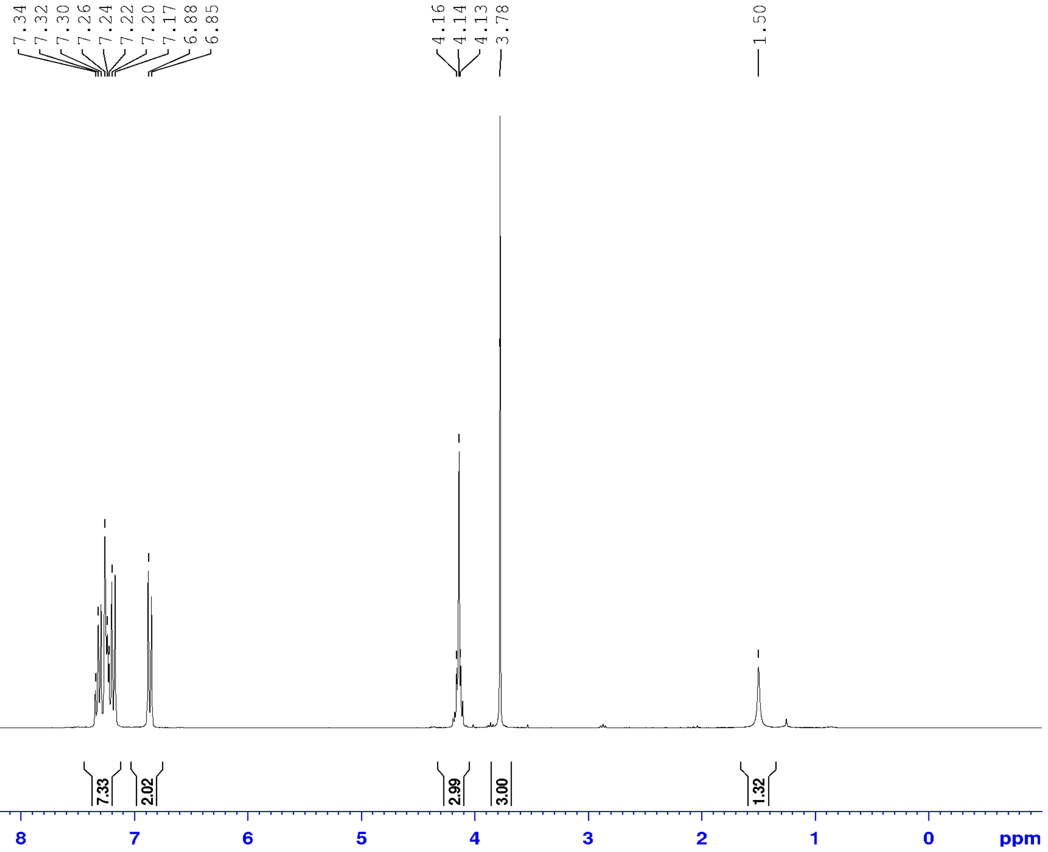
7.34
7.32
7.30
7.26
7.24
7.22
7.20
7.17
6.88
6.85
4.16
4.14
4.13
3.78
1.50
7.33
2.02
2.99
3.00
1.32
9
8
7
6
5
4
3
2
1
0
ppm

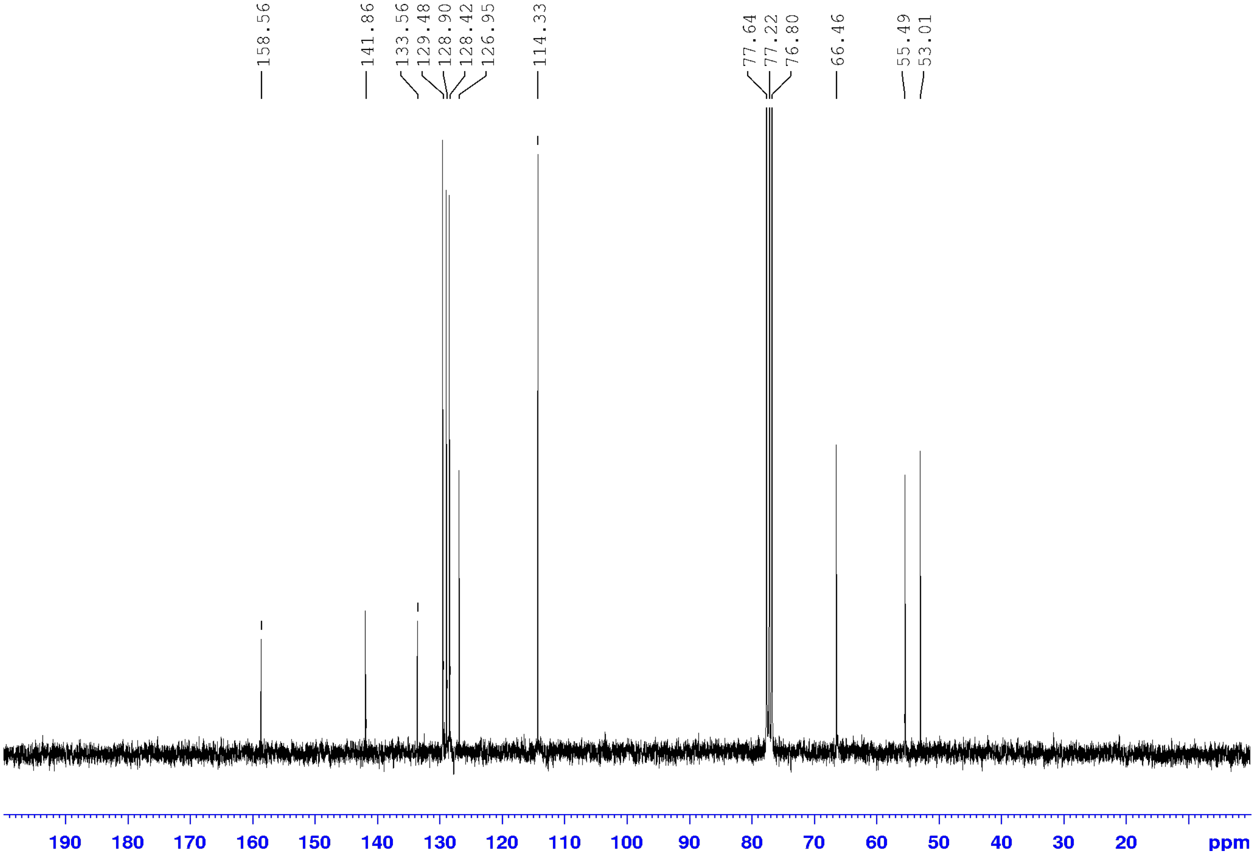
158.56
141.86
133.56
129.48
128.90
128.42
126.95
114.33
77.64
77.22
76.80
66.46
55.49
53.01
190
180
170
160
150
140
130
120
110
100
90
80
70
60
50
40
30
20
ppm

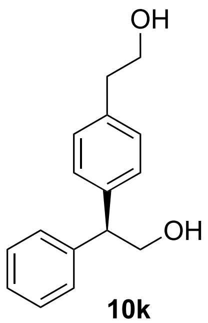
OH
OH

10k

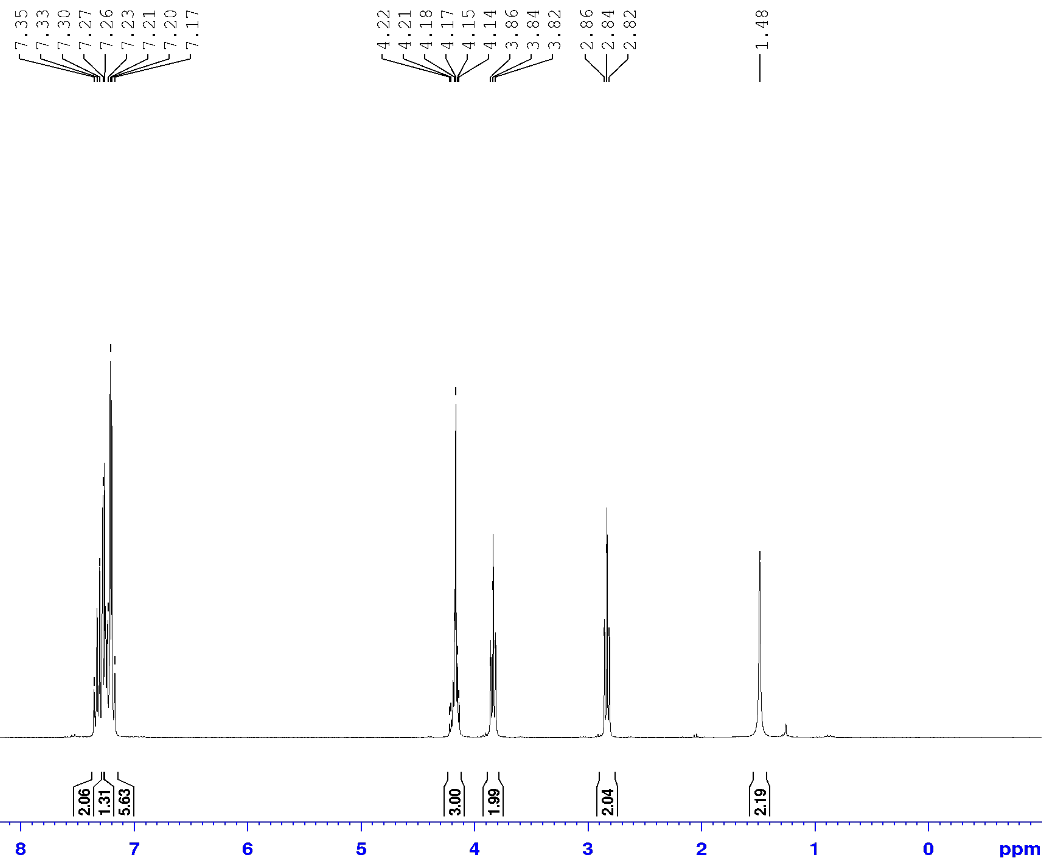
7.35
7.33
7.30
7.27
7.26
7.23
7.21
7.20
7.17
4.22
4.21
4.18
4.17
4.15
4.14
3.86
3.84
3.82
2.86
2.84
2.82
1.48
2.06
1.31
5.63
3.00
1.99
2.04
2.19
9
8
7
6
5
4
3
2
1
0
ppm

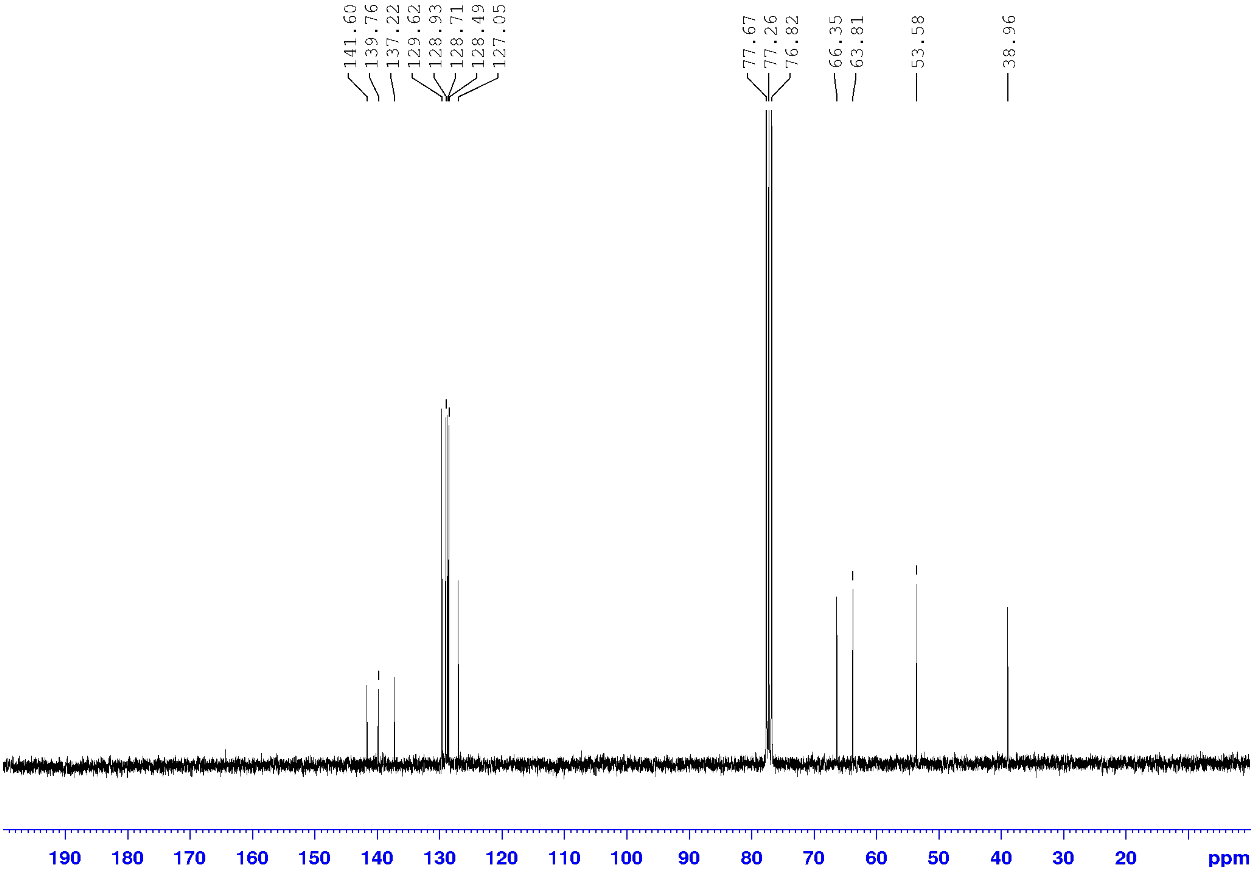
141.60
139.76
137.22
129.62
128.93
128.71
128.49
127.05
77.67
77.26
76.82
66.35
63.81
53.58
38.96
190
180
170
160
150
140
130
120
110
100
90
80
70
60
50
40
30
20
ppm

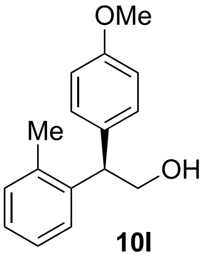
OMe
Me
OH

10l

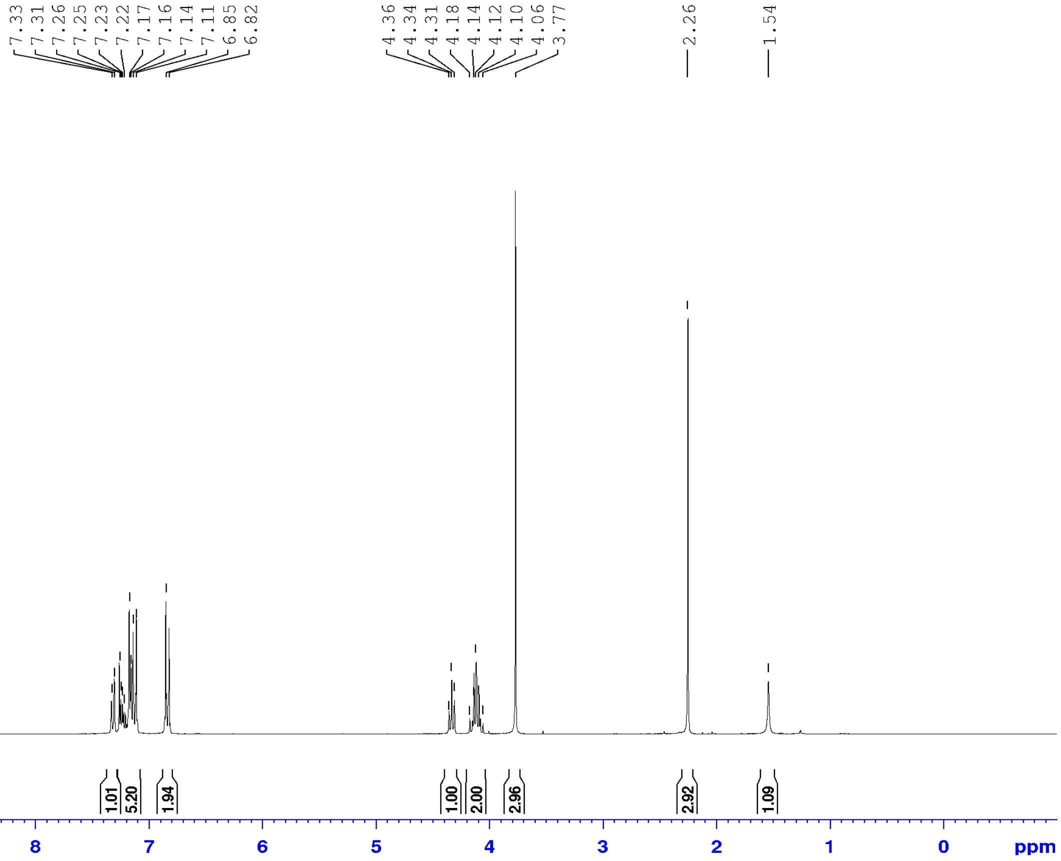
7.33
7.31
7.26
7.25
7.23
7.22
7.17
7.16
7.14
7.11
6.85
6.82
4.36
4.34
4.31
4.18
4.14
4.12
4.10
4.06
3.77
2.26
1.54
1.01
5.20
1.94
1.00
2.00
2.96
2.92
1.09
9
8
7
6
5
4
3
2
1
0
ppm

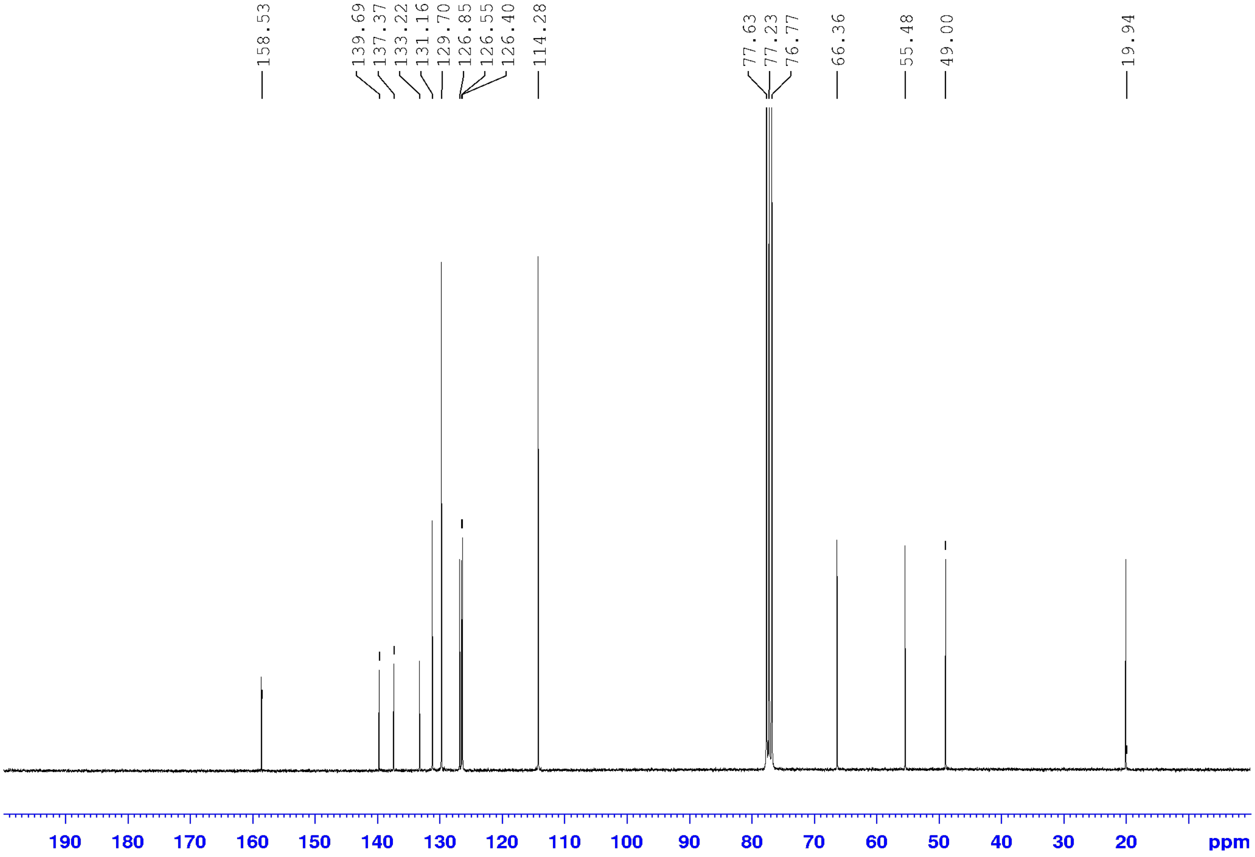
158.53
139.69
137.37
133.22
131.16
129.70
126.85
126.55
126.40
114.28
77.63
77.23
76.77
66.36
55.48
49.00
19.94
190
180
170
160
150
140
130
120
110
100
90
80
70
60
50
40
30
20
ppm

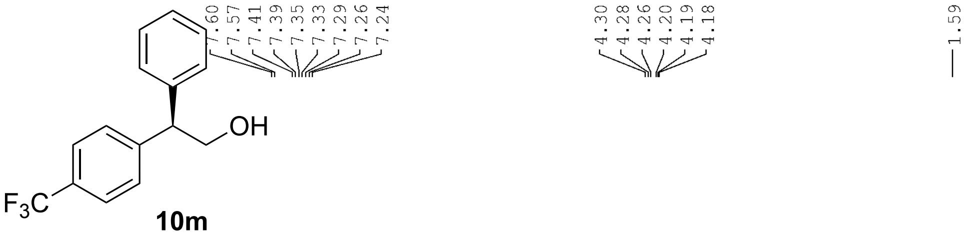
F3C
OH

10m

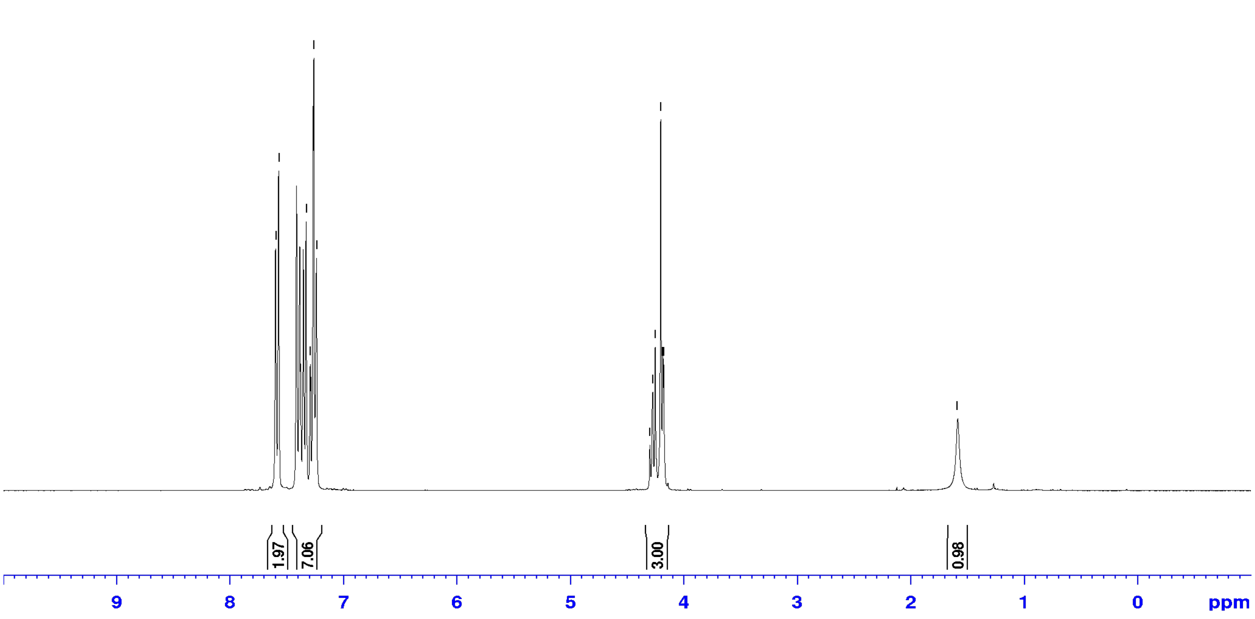
7.60
7.57
7.41
7.39
7.35
7.33
7.29
7.26
7.24
4.30
4.28
4.26
4.20
4.19
4.18
1.59
1.97
7.06
3.00
0.98
9
8
7
6
5
4
3
2
1
0
ppm

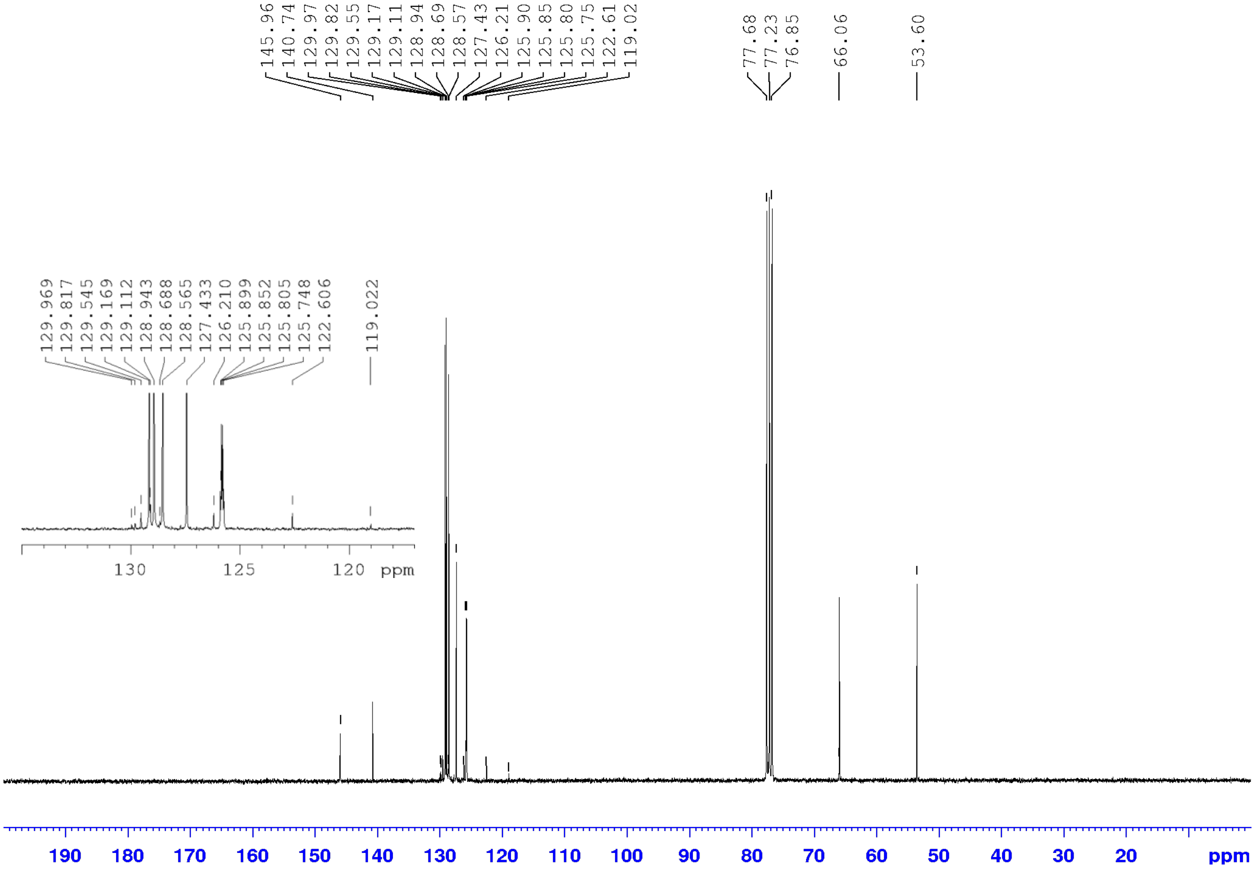
145.96
140.74
129.97
129.82
129.55
129.17
129.11
128.94
128.69
128.57
127.43
126.21
125.90
125.85
125.80
125.75
122.61
119.02
77.68
77.23
76.85
66.06
53.60
129.969
129.817
129.545
129.169
129.112
128.943
128.688
128.565
127.433
126.210
125.899
125.852
125.805
125.748
122.606
119.022
130
125
120
ppm
190
180
170
160
150
140
130
120
110
100
90
80
70
60
50
40
30
20
ppm

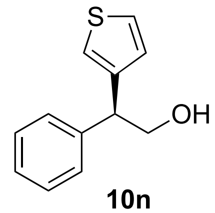
S
OH

10n

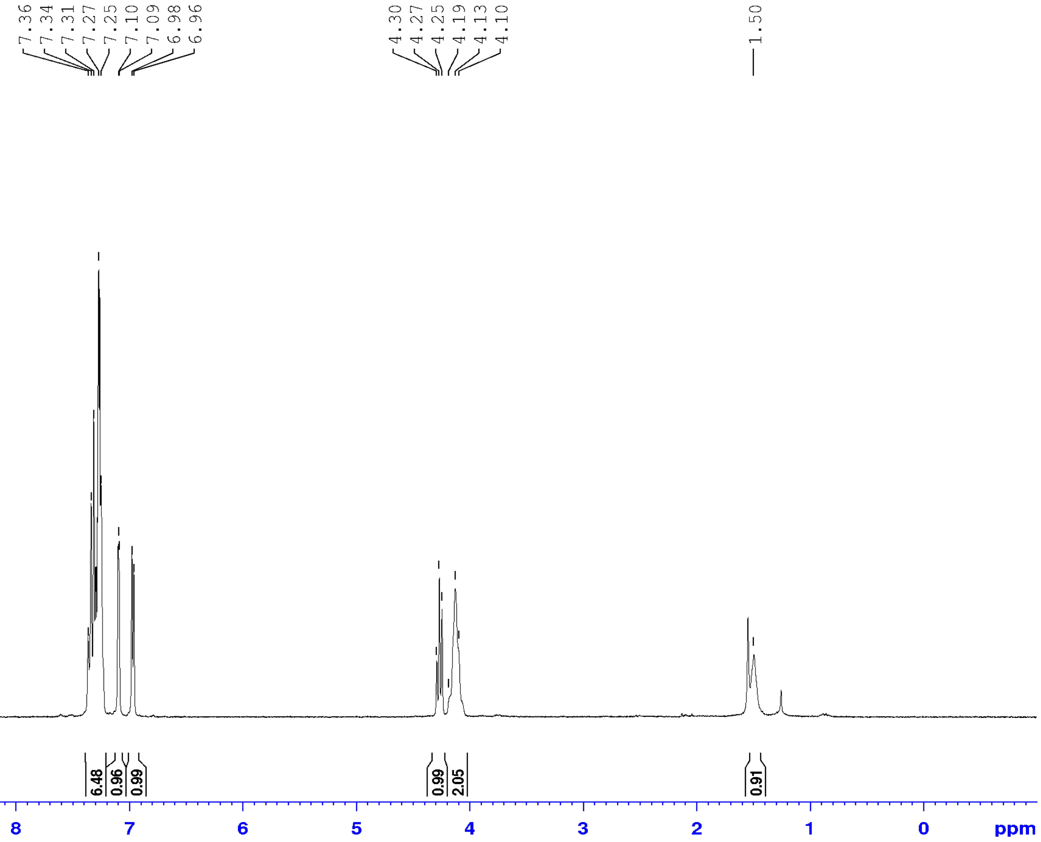
7.36
7.34
7.31
7.27
7.25
7.10
7.09
6.98
6.96
4.30
4.27
4.25
4.19
4.13
4.10
1.50
6.48
0.96
0.99
0.99
2.05
0.91
9
8
7
6
5
4
3
2
1
0
ppm

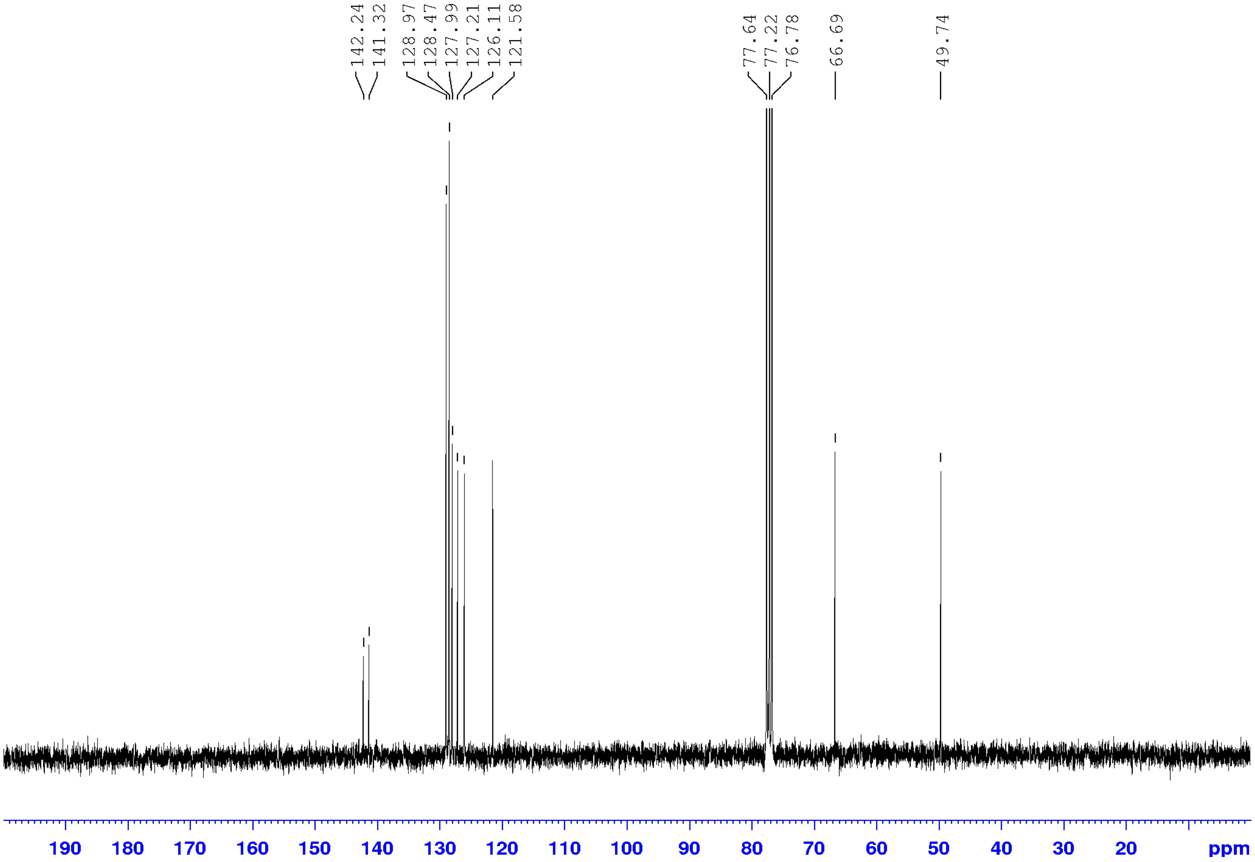
142.24
141.32
128.97
128.47
127.99
127.21
126.11
121.58
77.64
77.22
76.78
66.69
49.74
190
180
170
160
150
140
130
120
110
100
90
80
70
60
50
40
30
20
ppm

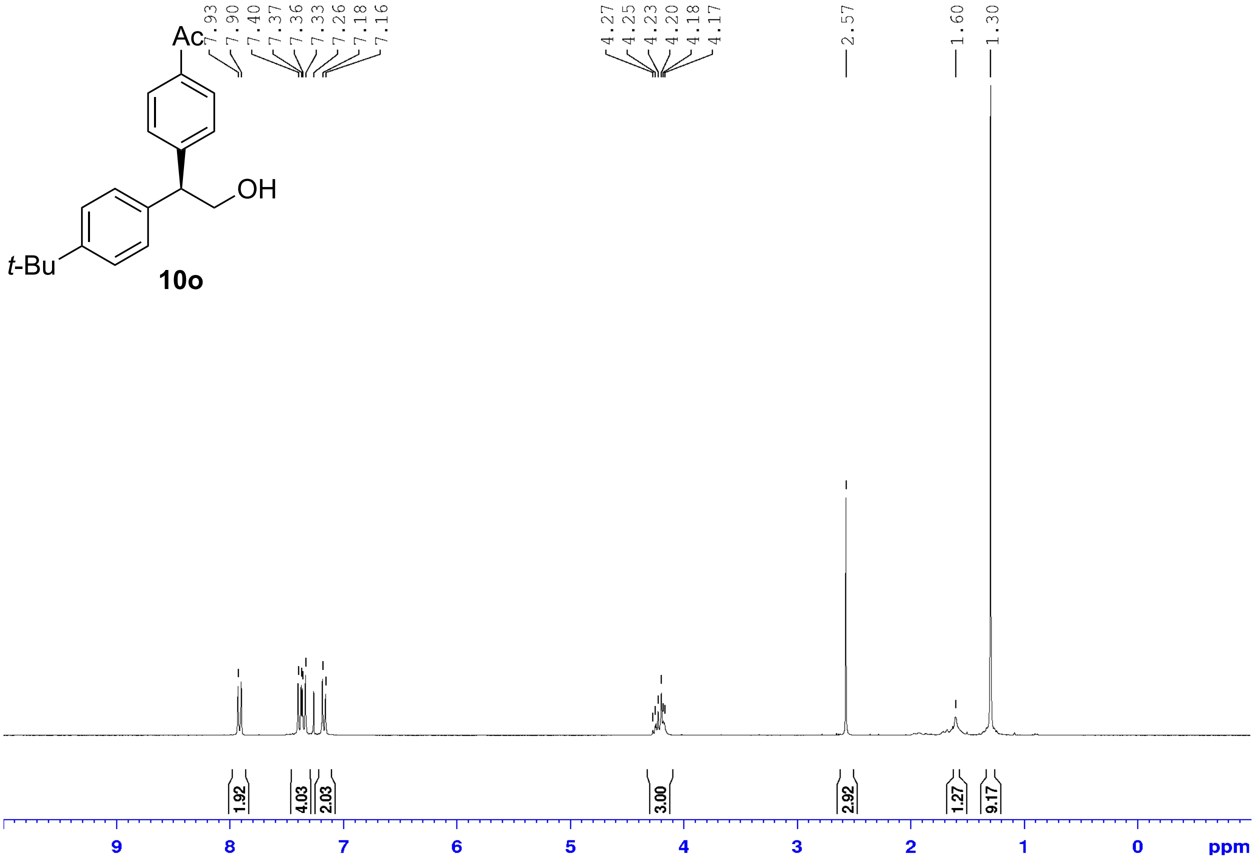
Ac
OH
t-Bu
10o
7.93
7.90
7.40
7.37
7.36
7.33
7.26
7.18
7.16
4.27
4.25
4.23
4.20
4.18
4.17
2.57
1.60
1.30
1.92
4.03
2.03
3.00
2.92
1.27
9.17
9
8
7
6
5
4
3
2
1
0
ppm

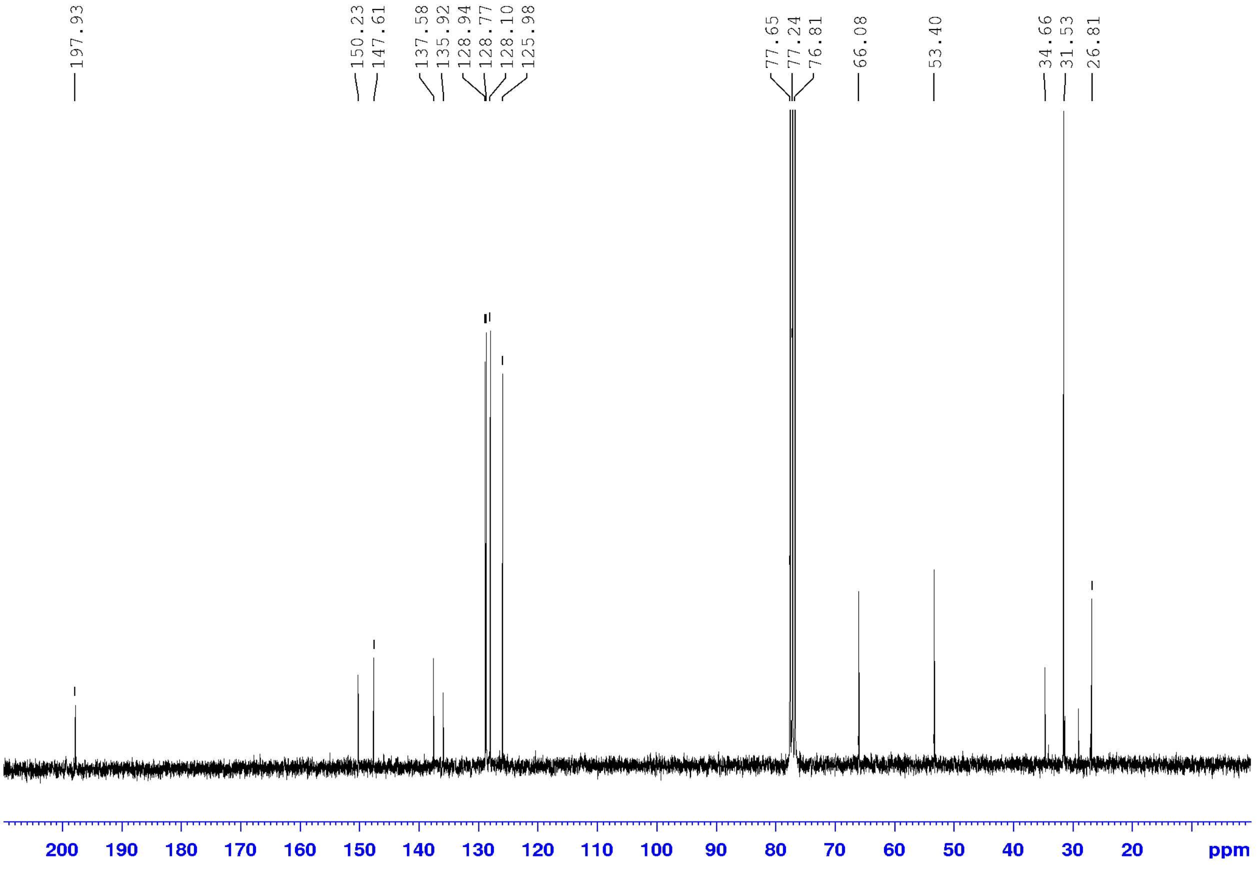
197.93
150.23
147.61
137.58
135.92
128.94
128.77
128.10
125.98
77.65
77.24
76.81
66.08
53.40
34.66
31.53
26.81
200
190
180
170
160
150
140
130
120
110
100
90
80
70
60
50
40
30
20
ppm

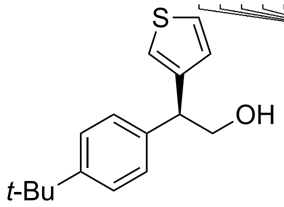
S
OH
t-Bu

10p

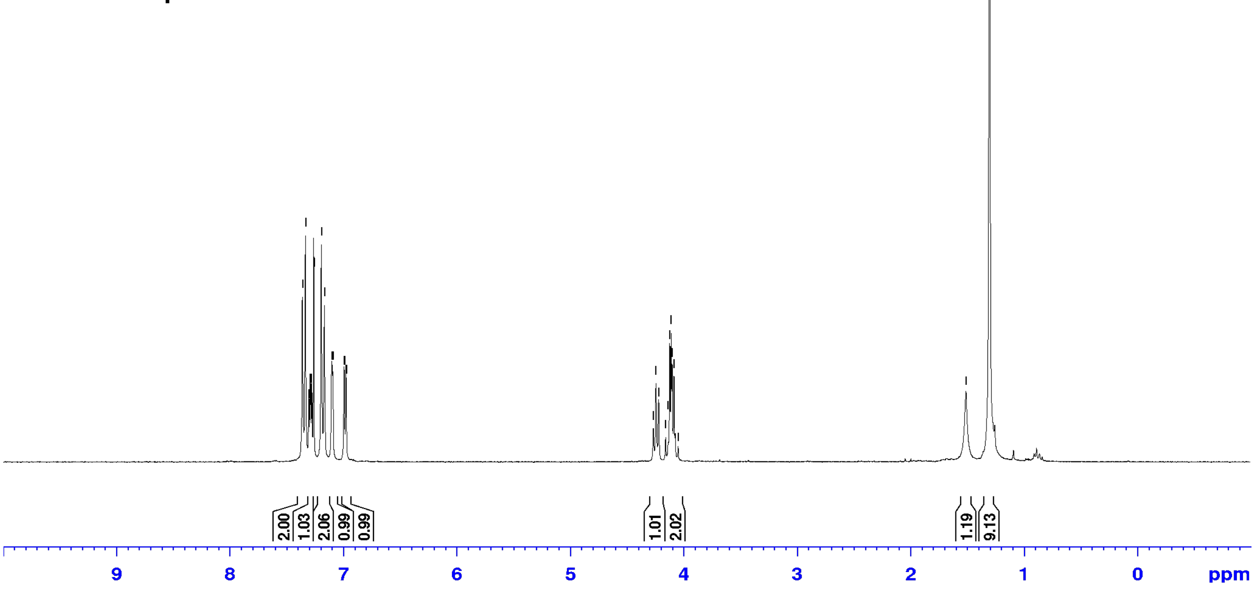
7.36
7.33
7.30
7.29
7.29
7.28
7.26
7.19
7.16
7.10
7.09
6.99
6.99
6.98
6.97
4.27
4.25
4.22
4.16
4.14
4.13
4.11
4.10
4.09
4.05
1.51
1.31
2.00
1.03
2.06
0.99
0.99
1.01
2.02
1.19
9.13
9
8
7
6
5
4
3
2
1
0
ppm

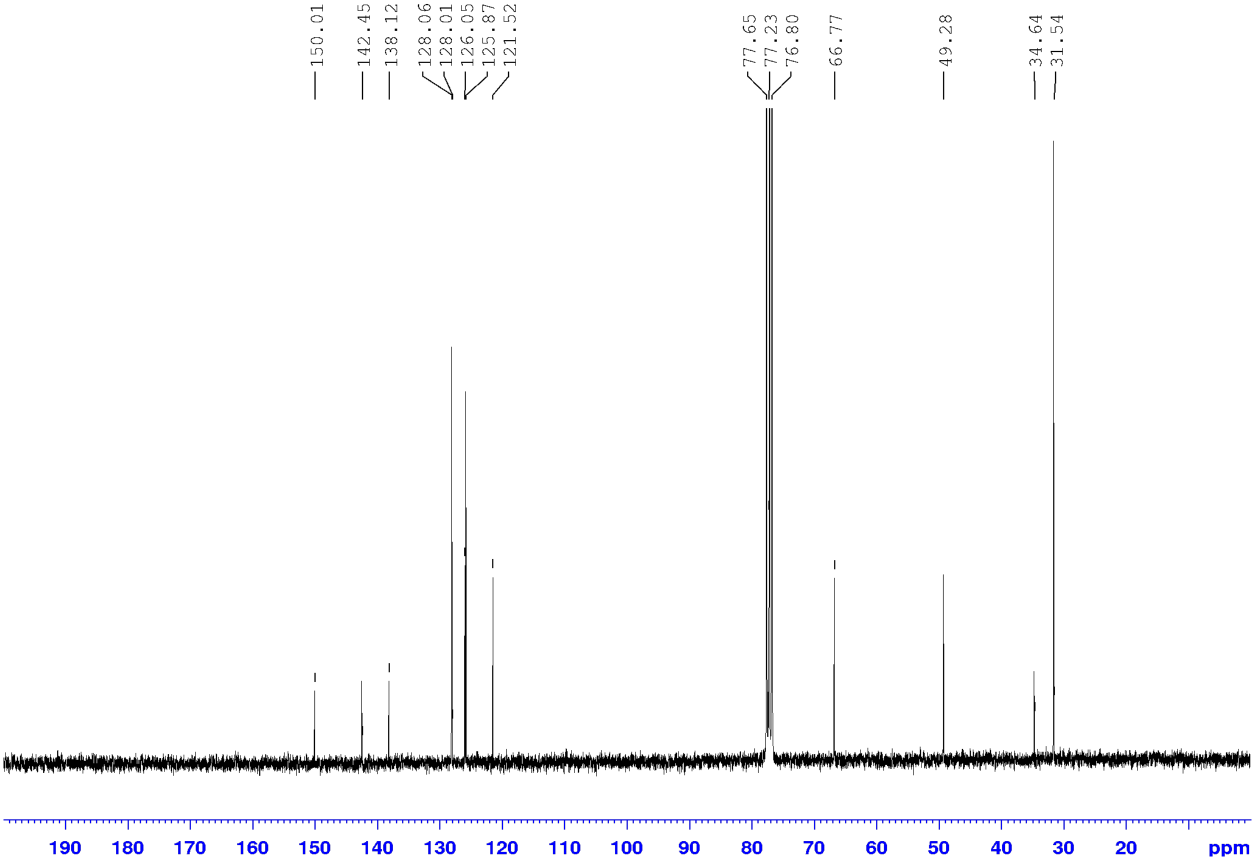
150.01
142.45
138.12
128.06
128.01
126.05
125.87
121.52
77.65
77.23
76.80
66.77
49.28
34.64
31.54
190
180
170
160
150
140
130
120
110
100
90
80
70
60
50
40
30
20
ppm

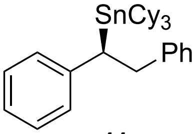
SnCy3
Ph

11a

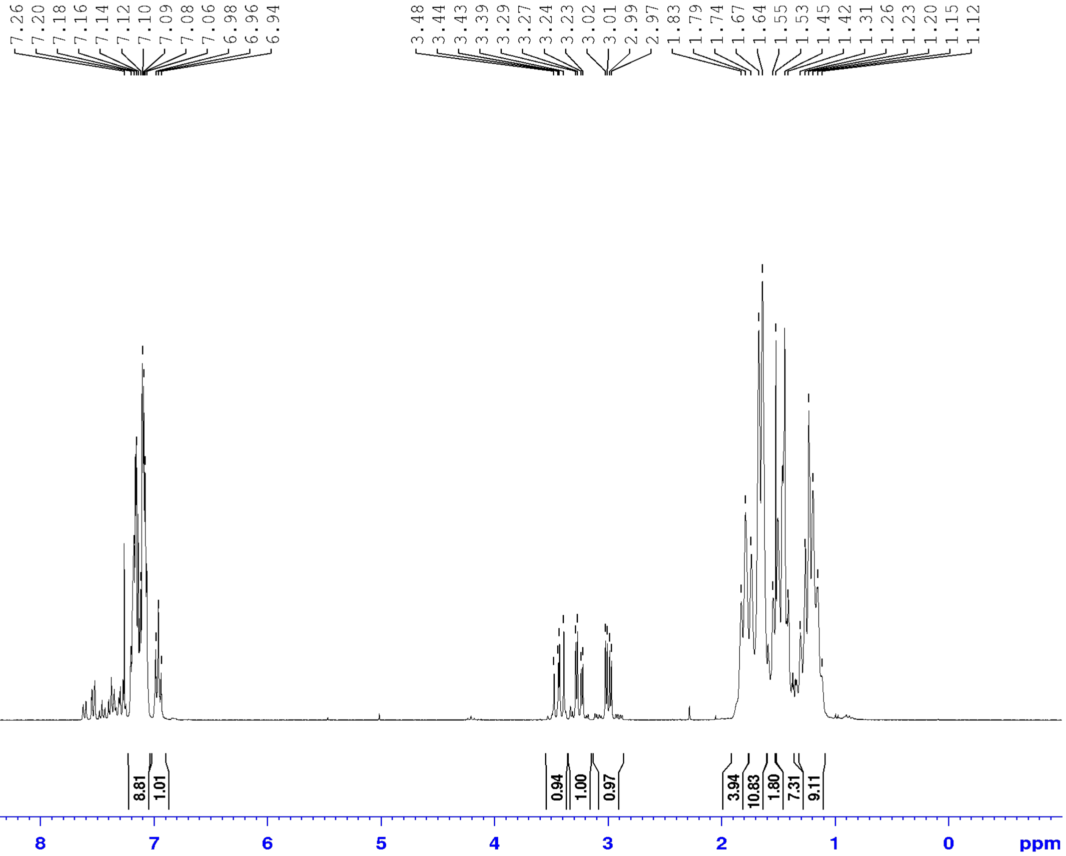
7.26 7.20 7.18 7.16 7.14 7.12 7.10 7.09 7.08 7.06 6.98 6.96 6.94
3.48 3.44 3.43 3.39 3.29 3.27 3.24 3.23 3.02 3.01 2.99 2.97 1.83 1.79 1.74 1.67 1.64 1.55 1.53 1.45 1.42 1.31 1.26 1.23 1.20 1.15 1.12
8.81 1.01 0.94 1.00 0.97 3.94 10.83 1.80 7.31 9.11
9 8 7 6 5 4 3 2 1 0 ppm

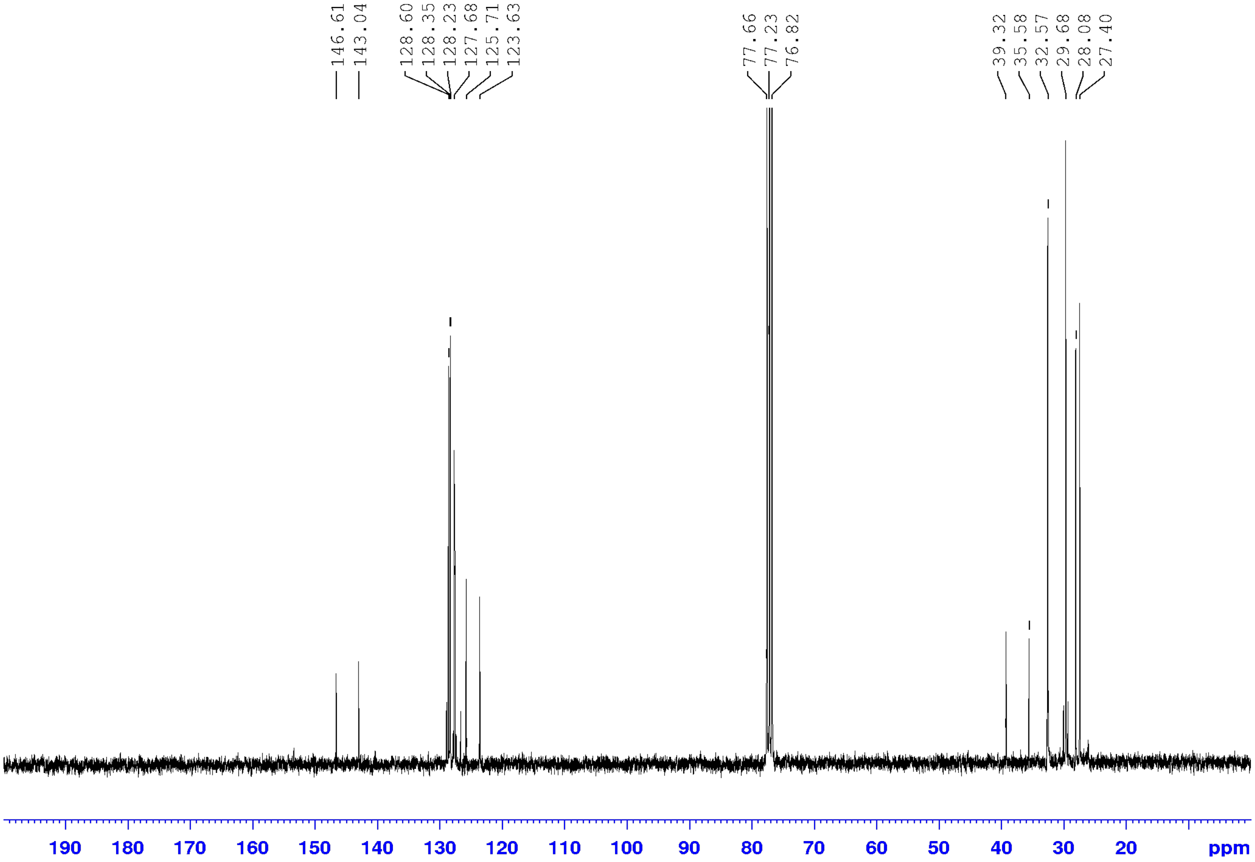
146.61 143.04 128.60 128.35 128.23 127.68 125.71 123.63 77.66 77.23 76.82 39.32 35.58 32.57 29.68 28.08 27.40
190 180 170 160 150 140 130 120 110 100 90 80 70 60 50 40 30 20 ppm

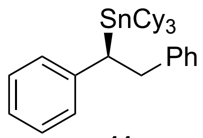

11a

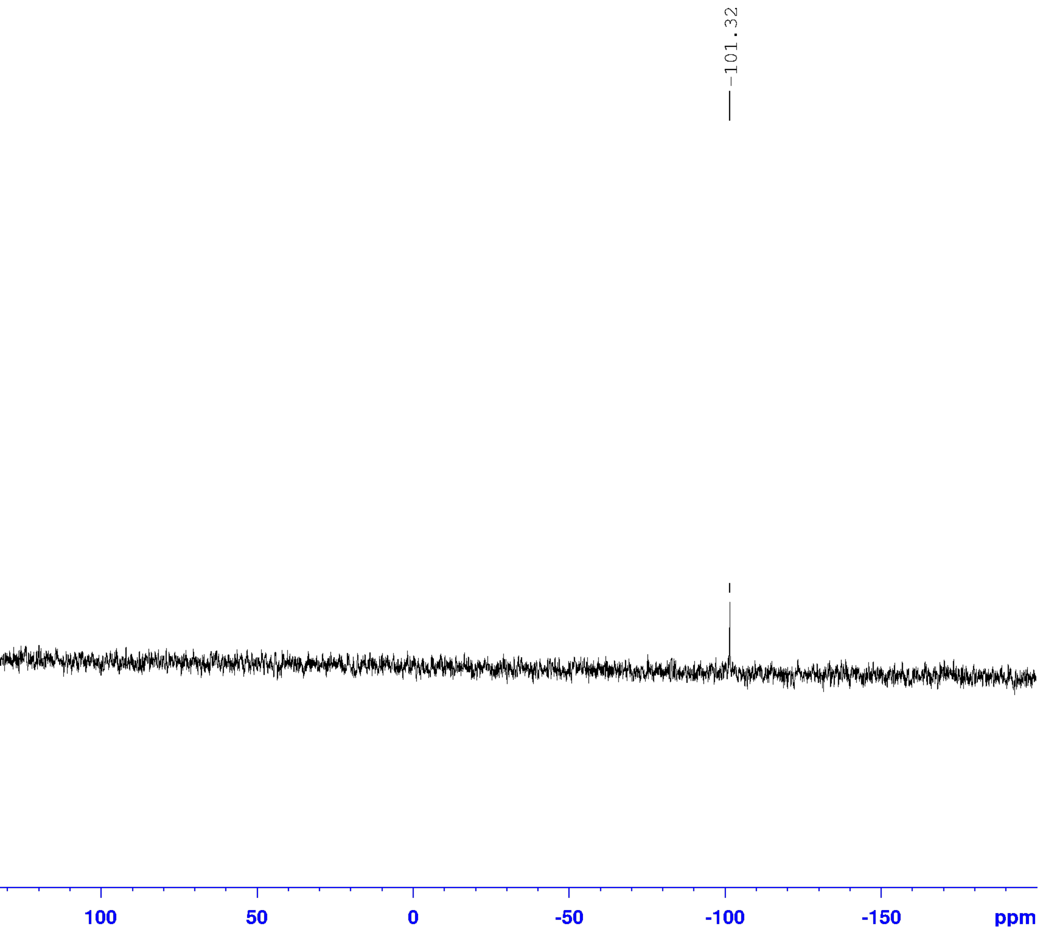

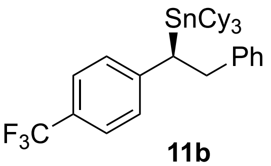

11b

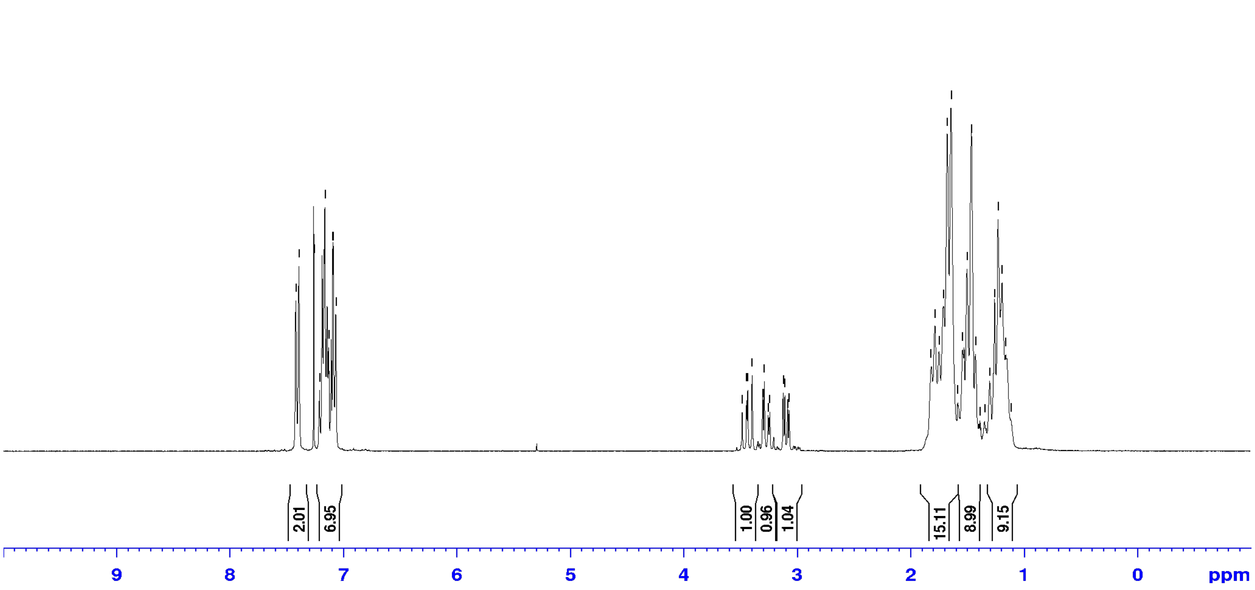

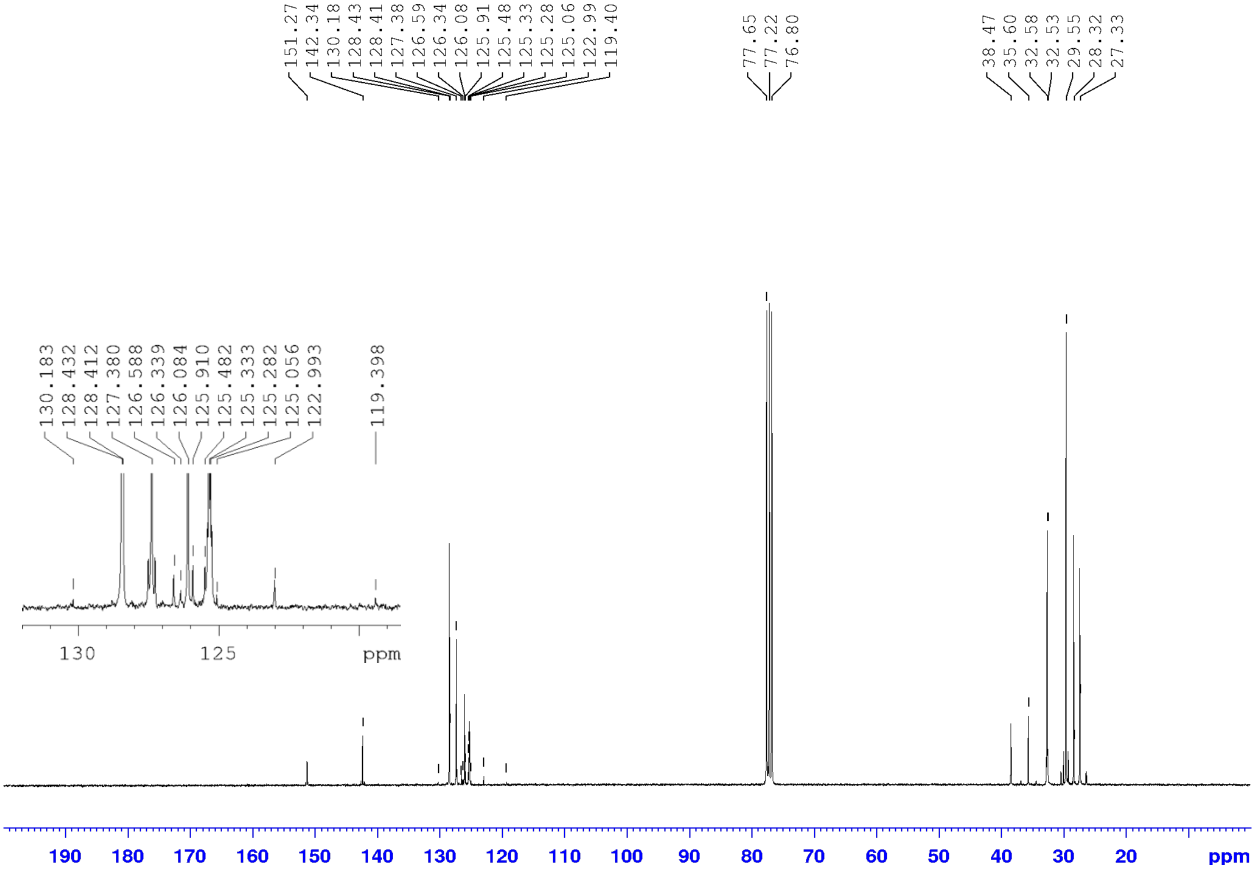

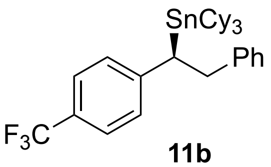

11b

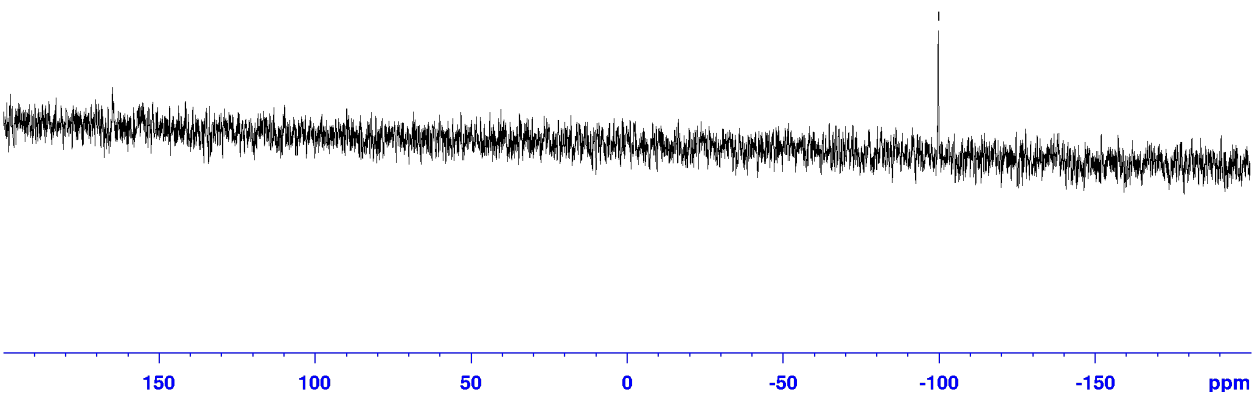

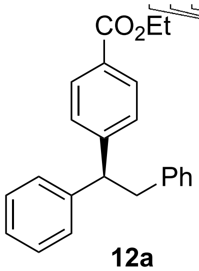

CO2Et
Ph

12a

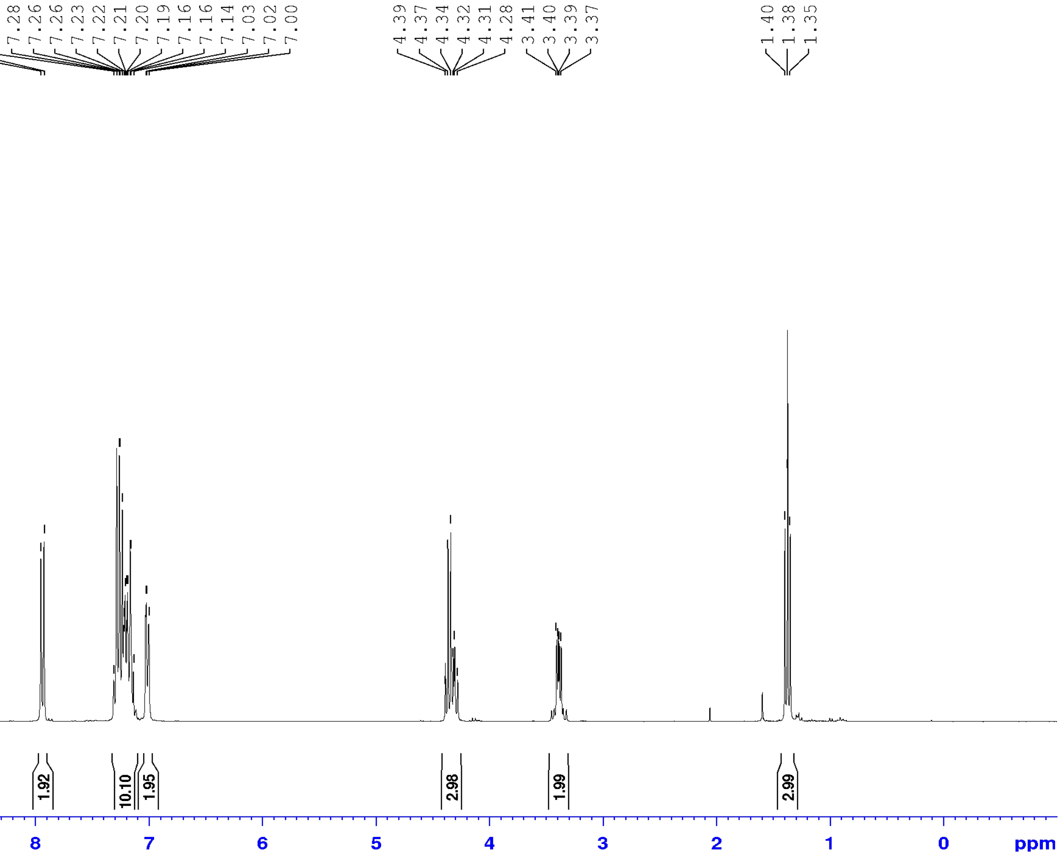

7.95
7.92
7.31
7.28
7.26
7.26
7.23
7.22
7.21
7.20
7.19
7.16
7.14
7.03
7.02
7.00
4.39
4.37
4.34
4.32
4.31
4.28
3.41
3.40
3.39
3.37
1.40
1.38
1.35
1.92
10.10
1.95
2.98
1.99
2.99
9
8
7
6
5
4
3
2
1
0
ppm

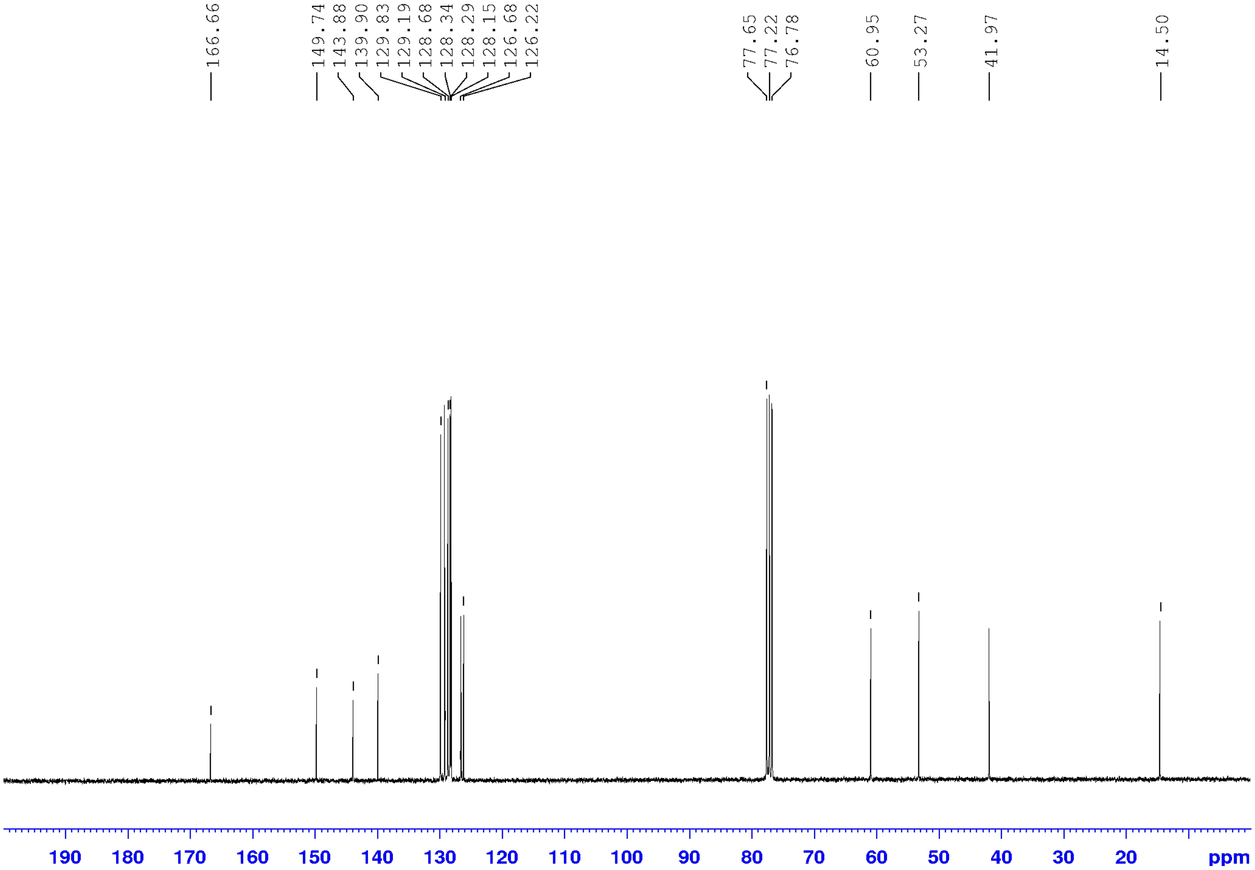

166.66
149.74
143.88
139.90
129.83
129.19
128.68
128.34
128.29
128.15
126.68
126.22
77.65
77.22
76.78
60.95
53.27
41.97
14.50
190
180
170
160
150
140
130
120
110
100
90
80
70
60
50
40
30
20
ppm

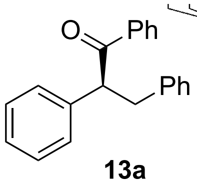
O
Ph
Ph

13a

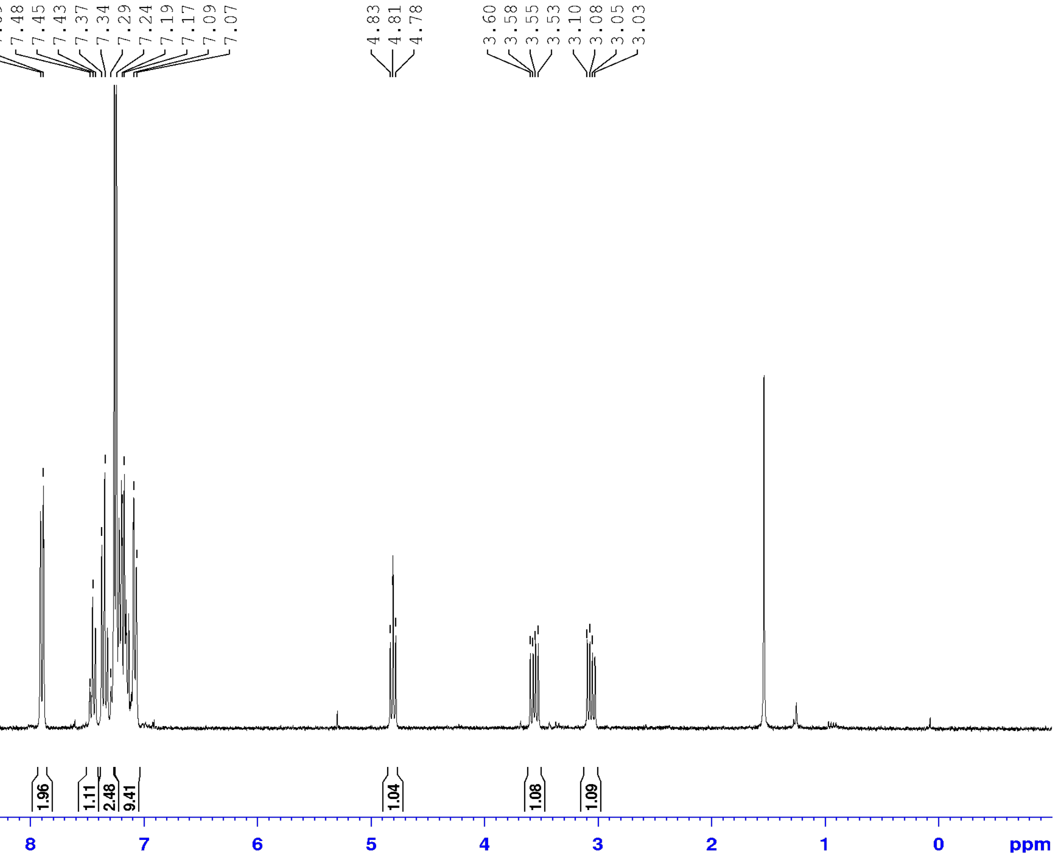
7.91
7.89
7.48
7.45
7.43
7.37
7.34
7.29
7.24
7.19
7.17
7.09
7.07
4.83
4.81
4.78
3.60
3.58
3.55
3.53
3.10
3.08
3.05
3.03
1.96
1.11
2.48
9.41
1.04
1.08
1.09
9
8
7
6
5
4
3
2
1
0
ppm

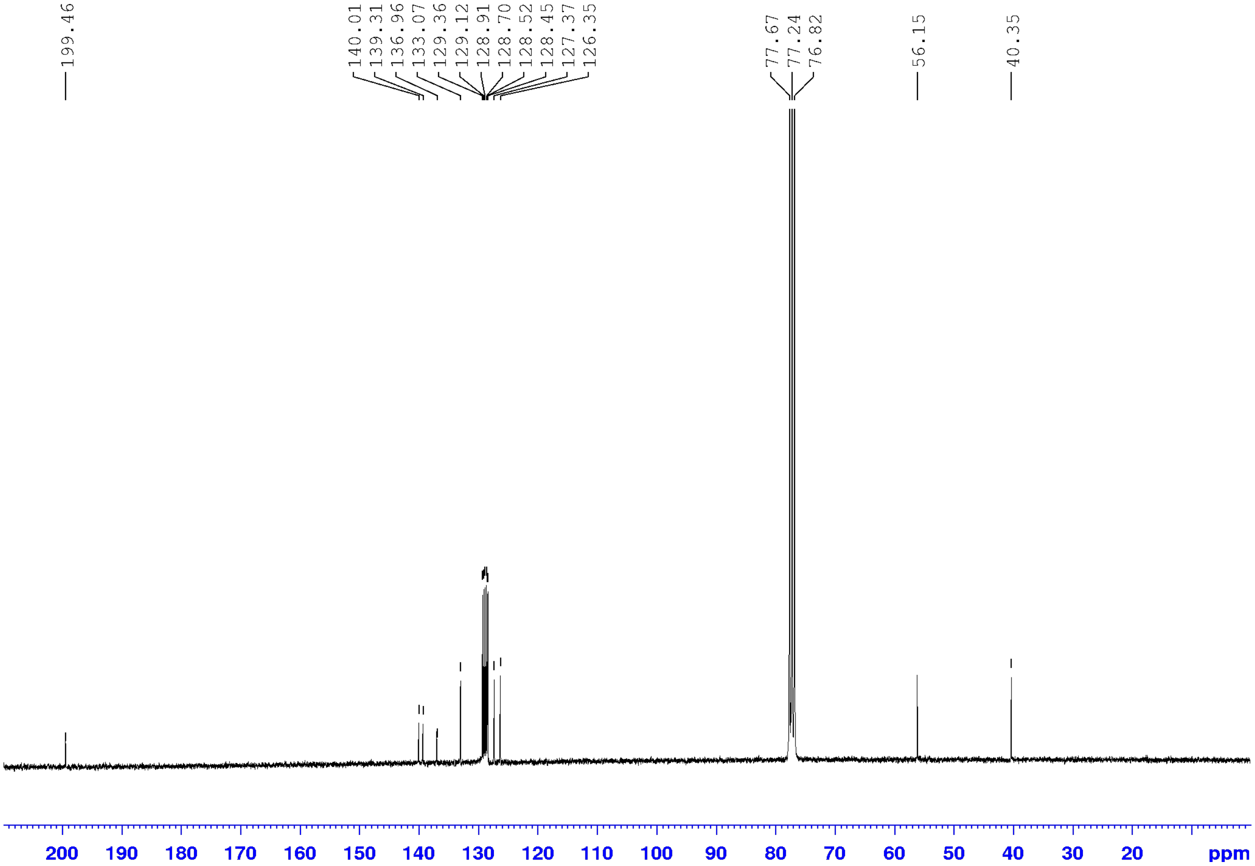
199.46
140.01
139.31
136.96
133.07
129.36
129.12
128.91
128.70
128.52
128.45
127.37
126.35
77.67
77.24
76.82
56.15
40.35
200
190
180
170
160
150
140
130
120
110
100
90
80
70
60
50
40
30
20
ppm

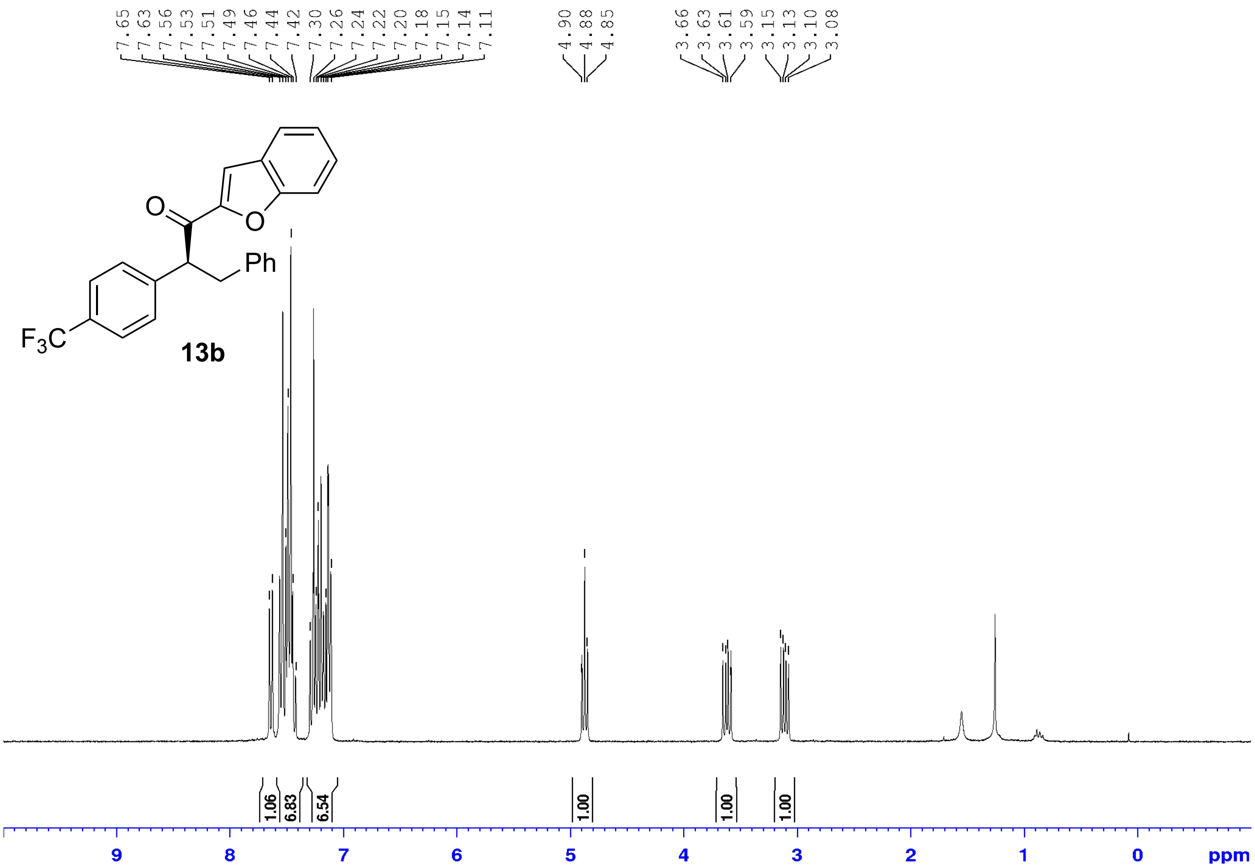
7.65 7.63 7.56 7.53 7.51 7.49 7.46 7.44 7.42 7.30 7.26 7.24 7.22 7.20 7.18 7.15 7.14 7.11
4.90 4.88 4.85
3.66 3.63 3.61 3.59 3.15 3.13 3.10 3.08
O
O
Ph
F_3C
13b
1.06 6.83 6.54 1.00 1.00 1.00
9 8 7 6 5 4 3 2 1 0 ppm

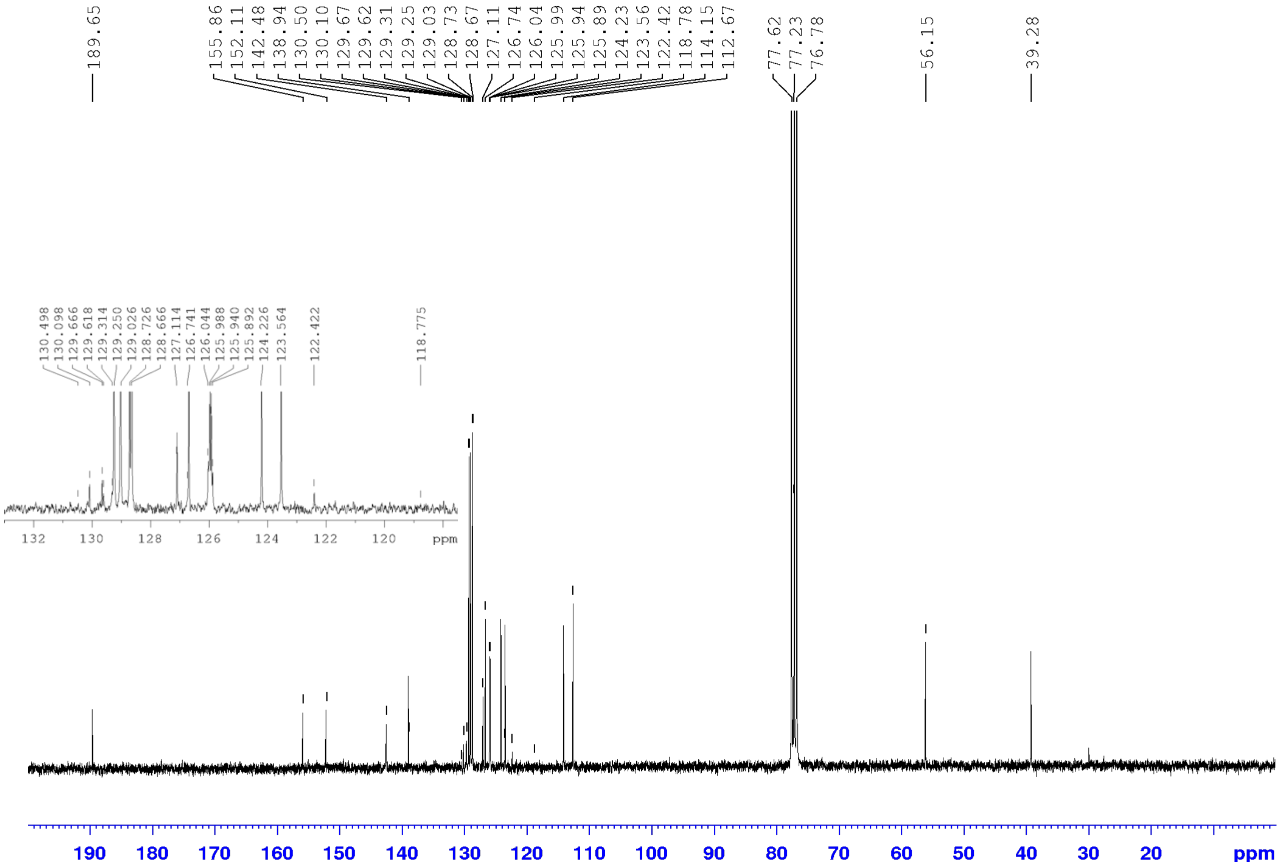
189.65
155.86 152.11 142.48 138.94 130.50 130.10 129.67 129.62 129.31 129.25 129.03 128.73 128.67 127.11 126.74 126.04 125.99 125.94 125.89 124.23 123.56 122.42 118.78 114.15 112.67
77.62 77.23 76.78
56.15
39.28
130.498 130.098 129.666 129.618 129.314 129.250 129.026 128.726 128.666 127.114 126.741 126.044 125.988 125.940 125.892 124.226 123.564 122.422 118.775
132 130 128 126 124 122 120 ppm
190 180 170 160 150 140 130 120 110 100 90 80 70 60 50 40 30 20 ppm
